# The regulatory pattern of target gene expression by aberrant enhancer methylation in glioblastoma

**DOI:** 10.1186/s12859-021-04345-8

**Published:** 2021-09-05

**Authors:** Xiaoxiao Zhao, Jianghuai Ji, Shijia Wang, Rendong Wang, Qiuhong Yu, Dongguo Li

**Affiliations:** 1 School of Biomedical Engineering, Capital Medical University, 10 You An Men Wai, Xi Tou Tiao, Beijing, 100069 People’s Republic of China; 2 Beijing Key Laboratory of Fundamental Research on Biomechanics in Clinical, Capital Medical University, Beijing, 100069 People’s Republic of China; 3 Department of Hyperbaric Oxygen, Beijing Tiantan Hospital, Capital Medical University, 119 Nansihuan Xi Lu, Fengtai District, Beijing, 100070 People’s Republic of China; 4 Department of Radiation Physics, Zhejiang Cancer Hospital, Hangzhou, 310022 People’s Republic of China; 5 Zhejiang Key Laboratory of Radiation Oncology, Hangzhou, 310022 People’s Republic of China

**Keywords:** Glioblastoma multiforme, Enhancer region, DNA methylation, Epigenetic regulation, Biomarker

## Abstract

**Background:**

Glioblastoma multiforme (GBM) is the most common and aggressive primary malignant brain tumor with grim prognosis. Aberrant DNA methylation is an epigenetic mechanism that promotes GBM carcinogenesis, while the function of DNA methylation at enhancer regions in GBM remains poorly described.

**Results:**

We integrated multi-omics data to identify differential methylation enhancer region (DMER)-genes and revealed global enhancer hypomethylation in GBM. In addition, a DMER-mediated target genes regulatory network and functional enrichment analysis of target genes that might be regulated by hypomethylation enhancer regions showed that aberrant enhancer regions could contribute to tumorigenesis and progression in GBM. Further, we identified 22 modules in which lncRNAs and mRNAs synergistically competed with each other. Finally, through the construction of drug-target association networks, our study identified potential small-molecule drugs for GBM treatment.

**Conclusions:**

Our study provides novel insights for understanding the regulation of aberrant enhancer region methylation and developing methylation-based biomarkers for the diagnosis and treatment of GBM.

**Supplementary Information:**

The online version contains supplementary material available at 10.1186/s12859-021-04345-8.

## Background

Glioblastoma multiforme (GBM) is the most common and deadly brain tumor and is classified as a grade IV glioma [1]. It is a highly invasive cancer that is characterized by changes in cerebral vessels and the gradual invasion of surrounding tissues along the perivascular space [2, 3]. GBM cells typically invade up to several centimeters away from the tumor mass and can even cross into the contralateral hemisphere [4, 5]. Although the current most advanced therapeutic treatment combining surgical resection, radiotherapy and chemotherapy [6], due to the radiotherapy resistance of GBM stem cells (GSCs) to traditional treatment, GBM patients are prone to relapse after treatment, and the median survival time is only 14.6 months [7, 8]. Therefore, there is an urgent need to make new progress in the study of accurate molecular mechanisms and reliable therapeutic targets of GBM.

In recent years, targeted therapies have made great progress in many types of cancer. Aberrant gene expression can be used as a target to develop new biomarkers for disease monitoring and prognosis or treatment response. The extensively studied mRNA in GBM is O6‐methylguanine‐DNA methyltransferase (MGMT). The expression of MGMT is highly regulated by a variety of transcription factors, which activate the MGMT promoter and induce more expression of MGMT [9]. Besides, biomarkers are not limited to protein-coding genes, and lncRNAs have become a hotspot of current research. For instance, overexpression of TP73-AS1 predicts poor prognosis in primary GBM cohorts and that this lncRNA promotes tumor aggressiveness and TMZ resistance in GSCs [10]. However, most of the current studies are limited to the expression of target genes in GBM, and few studies focus on the regulation mechanism of gene expression, particularly the regulation of DNA methylation on the expression of target genes in GBM.

DNA methylation is one of the most common epigenetic events in the mammalian genome. It is well known that aberration of DNA methylation contributes to carcinogenesis and it frequently occurs in the promoter region of genes [11]. In addition to promoters, enhancers have important roles in gene regulation that bind tissue-specific transcription factors and can regulate transcription at distant loci through chromosome looping [12]. Increasing evidence demonstrates that the methylation status of enhancer regions correlates better with target gene expression than promoters [13]. Aberrant methylation patterns in enhancers contribute to aberrant gene expression in multiple diseases, including many kinds of cancers [14–16]. For instance, Ying et al. [17] found that histone variants and different histone modifications interact with aberrant DNA methylation and cause perturbed enhancer activity in cytogenetically normal acute myeloid leukemia that contributes to a leukemic transcriptome. Recently, several studies have revealed aberrant DNA methylation in GBM, particularly methylation dynamics in gene promoters [18–20]. Nevertheless, few studies focused on enhancer regions, and the genome-wide enhancer methylation patterns in GBM remain unclear.

**Fig. 1:**
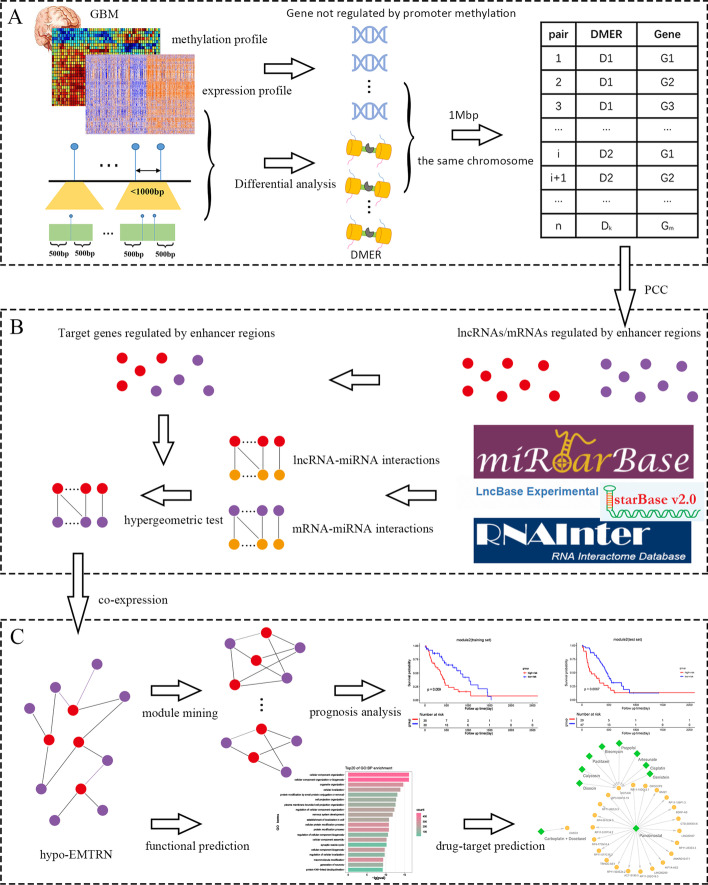
Workflow of our study. **A** Identification of genes regulated by DMERs through integrating multi-omics data. *DMERs* differential methylation enhancer regions. **B** Construction of lncRNA-mRNA co-expression networks. **C** Module mining and biomarker identification. *hypo-EMTRN* enhancer hypomethylation-mediated regulatory network

With the advancement of high-throughput sequencing technology, large-scale Illumina Infinium Human Methylation 450 BeadChip (Illumina HM450k) has been applied to cancer analysis. It contains 485,577 probes, which can target 99% of RefSeq genes and several other locations on the genome [21]. In this study, we developed an integrated model combining multi-omics data for identifying genes that might be regulated by differential methylation enhancer regions (DMERs) that might lead to tumorigenesis. The workflow of our study was shown in Fig. 1. Our study identified 191 lncRNAs and 1052 mRNAs whose expression might be regulated by hypomethylated enhancer regions. Then, an enhancer region hypomethylation-mediated regulatory network (hypo-EMTRN) was used to elucidate the regulatory mechanism of enhancer region and predict the biological function of target genes. Also, we used the biclique algorithm to identify 2651 synergistic, competitive modules, and then performed survival analysis to obtain 22 modules that might have clinical prognostic value. In particular, a higher degree of gene in the two modules might have better diagnostic and prognostic functions. Finally, through the construction of drug-target association networks, we identified potential small-molecule drugs for GBM treatment. This study shed light on the relationship between aberrant enhancer methylation and gene expression in GBM, and it might be of great help to the study of how methylation of enhancer regulates gene expression.

## Results

### Characterization of DMERs-driven target genes in GBM

**Fig. 2:**
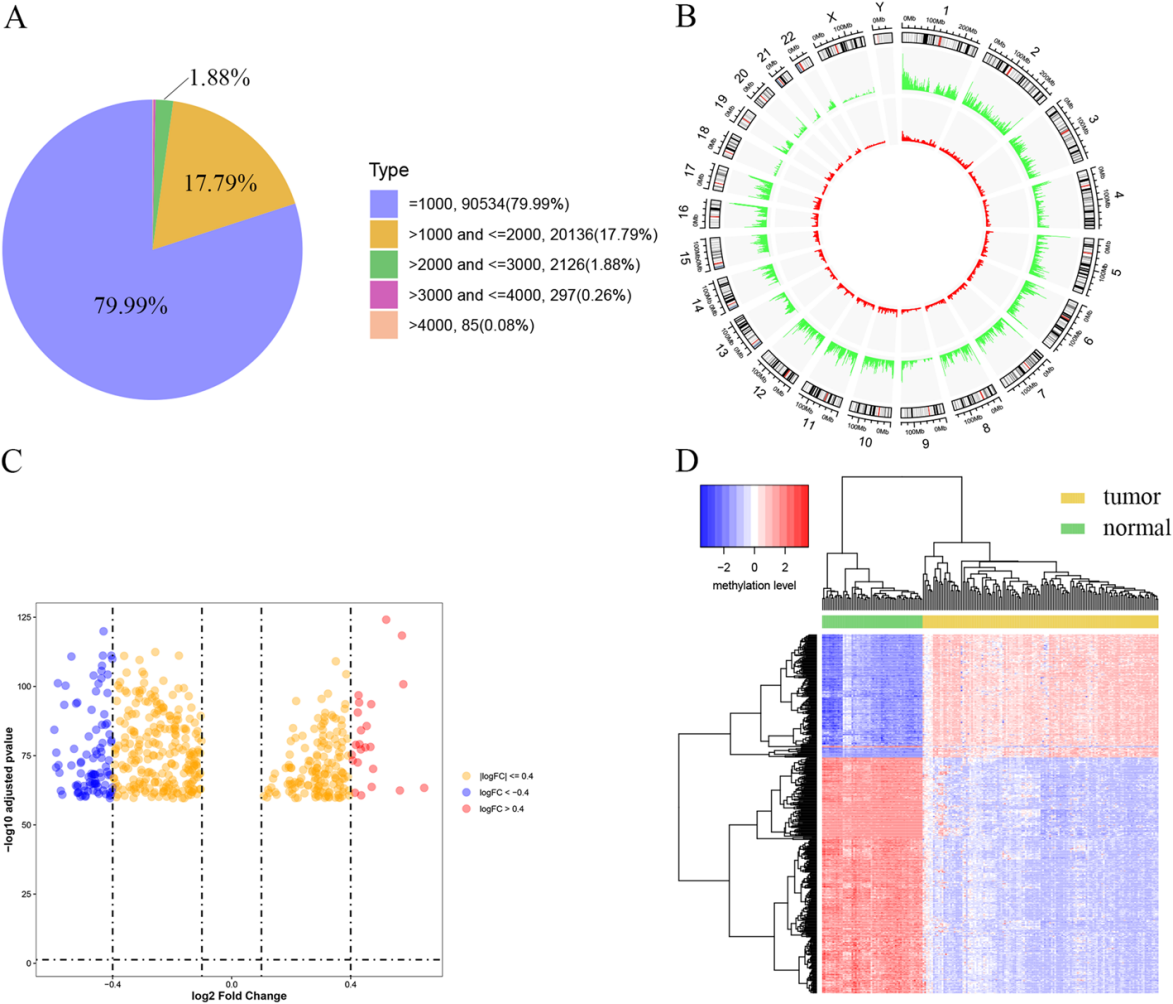
The DNA methylation pattern of enhancer region in GBM. **A** The pie chart shows the proportions of DMERs length. *DMERs* differential methylation enhancer regions. **B** A circos plot showing genomic regions that are significantly hypomethylated (37,818 regions, in green) or hypermethylated (7024 regions, in red) in the tumor group as compared to non-tumor group. **C** The volcano map shows the top 500 DMERs of significant difference. *DMERs* differential methylation enhancer regions. **D** Unsupervised hierarchical clustering analysis of the top five hundred DMERs with significant difference in GBM. On the x-axis, yellow represents the GBM samples and green represents the normal controls. The y-axis represents DMER. *DMERs* differential methylation enhancer regions

To characterize the DNA methylation pattern of enhancers in GBM, we applied a computational strategy to map the enhancer probes to the Infinium 450k array, thereby constructing the enhancer region methylation profile in GBM (described in the methods for details). In this study, we obtained overall CpG probes localized in enhancer regions from the GPL13534 comment file and the supplemental file of a previously published study [22]. 113,178 non-overlapping enhancer regions were constructed by using the calculation strategy. 79.99% of the regions had a length of 1000 bp, 17.79% a length > 1000 bp and < 2000 bp, and other regions accounted for 2.22% (Fig. 2A). Similarly, we used a computational method in the previous study to reannotate Infinium 450K arrays into promoter region of the gene [23]. In the present study, 54,477 probes were located in 20,386 gene promoter regions. Although each gene had several probes mapping to the corresponding promoter region, only the average value of DNA methylation probes in promoter of the was calculated as the DNA methylation level of the gene.

After preprocessing the methylation profiles, DMERs and differential promoter methylation genes (DPMGs) were recognized respectively between 136 tumor samples and 58 normal samples. In total, 44,841 DMERs were identified, including 7024 hypermethylated DMERs and 37,817 hypomethylated DMERs. Similarly, we obtained 4889 DPMGs. Globally, a clear hypomethylation pattern in the tumor group was observed when compared with the non-tumor group (Fig. 2B). 144 hypomethylated genes and 23 hypermethylated genes that might be regulated by promoter were identified by calculating the Pearson correlation coefficient (PCC) between the methylation value of each DPMG and the corresponding expression value. In order to further analyze the methylation pattern of the enhancer region, we used the DNA methylation data between the tumor and the normal control sample to explore the changes in its methylation level. Figure 2C showed the volcano map of the two elements (log_2_(FC) and adjusted *p* value) in the differential analysis of the DMERs (the top 500 rank of adjusted *p* value). The result showed that the number of hypomethylated enhancer regions in the top 500 rank of adjusted *p* value was much more than that of hypermethylated enhancer regions. Furthermore, the methylation levels of these DMERs with significant difference are shown in a heatmap (Fig. 2D). It was noted that these DMERs markedly differed between the tumor and normal tissues, and the number of hypomethylated enhancer regions was much greater than that of hypermethylated enhancer regions.

Aberrant enhancer methylation is common in many cancer types, is more closely related to target gene expression changes than promoter methylation, and might occur even when the promoter is constantly unmethylated [13]. To identify genes which might be regulated by the DMERs, we combined multi-omics data to build a model for associating enhancers with their target genes (DMERs-associated genes) (described in the methods and Additional file 1: Fig. S1A for details). Since it is known that there is an inverse correlation between methylation level of enhancer and chromatin activity [24], we retained only inversely correlated DMER-gene pairs. Finally, we obtained 5429 DMER-lncRNA pairs of which there were 4613 DMERs and 2819 lncRNAs, and 8909 DMER-mRNA pairs of which there were 6657 DMERs and 4440 mRNAs.

### The identification of cancer-related hallmarks in the regulatory network

**Fig. 3:**
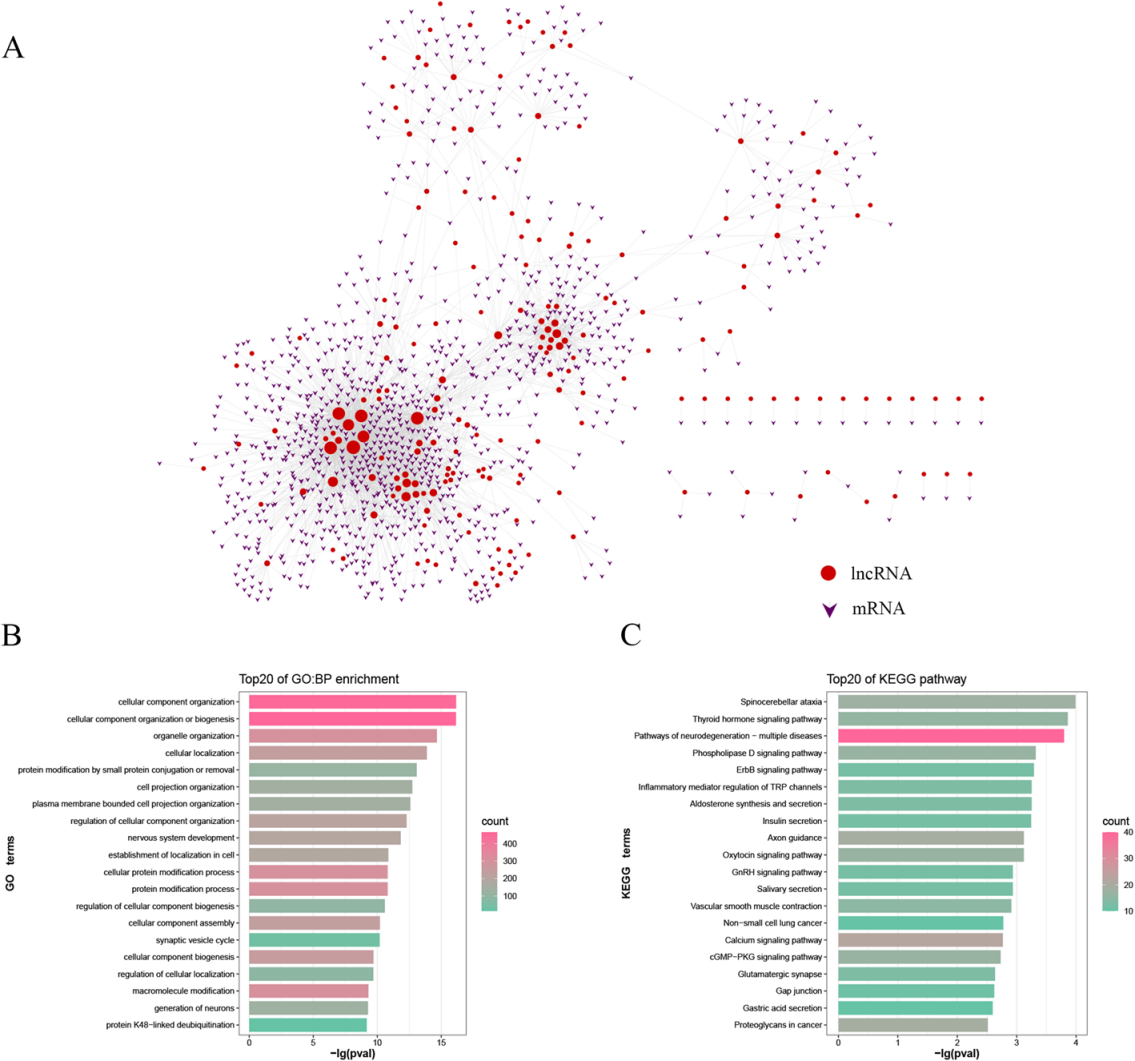
Construction of cancer-related target genes in regulatory network and Functional enrichment analysis. **A** lncRNA-mRNA co-expression network regulated by hypomethylated enhancer regions. The node degree is indicated by the node size. *lncRNA* long non-coding RNA. **B** The top 20 enriched BP items of upregulated genes. *BP* biological processes. **C** The top 20 enriched KEGG items of upregulated genes. *KEGG* Kyoto Encyclopedia of Genes and Genomes

Previous studies have demonstrated that enhancers dysregulate the expression of target genes through methylation-mediated epigenetic regulation and cause human disease [13, 17, 25]. Increasing evidence has shown that lncRNAs are involved in tumor growth, cell-cycle, and apoptosis through interactions with mRNAs [26, 27]. Hence, we constructed a lncRNA-mRNA co-expression regulatory network to study how these target genes that might be regulated by DMERs act synergistically to regulate the process of GBM, and found some biomarkers closely related to the occurrence and development of GBM. These target genes were matched with background network genes to obtain lncRNA-mRNA pairs. Then, the PCC of each lncRNA-mRNA pair was calculated based on the lncRNA and mRNA expression profiles. We used PCC > 0.5 and *p* value ≤ 0.05 as thresholds to screen out 3271 lncRNA-mRNA interactions. Finally, we constructed the enhancer region methylation-mediated target gene regulatory network (EMTRN) based on these relationship pairs (described in the methods and Additional file 1: Fig. S1B for details). The EMTRN contained 220 lncRNAs, 1173 mRNAs, and 3271 lncRNA-mRNA pairs. Since the lncRNAs and mRNAs in these relationship pairs were regulated by the hypermethylated enhancer regions or the hypomethylated enhancer regions, we divided the EMTRN into the hypermethylation-mediated regulatory network (hyper-EMTRN) and hypo-EMTRN. There were 29 lncRNAs, 121 mRNAs, 176 lncRNA-mRNA pairs in the hyper-EMTRN (Additional file 2: Fig. S2A and Additional file 4: Table S1) and 191 lncRNAs, 1052 mRNAs, 3095 lncRNA-mRNA pairs in the hypo-EMTRN (Fig. 3A and Additional file 5: Table S2). Obviously, we obtained many more target genes which might be regulated by hypomethylated enhancer than target genes which that might be regulated by hypermethylated enhancer in GBM. In general, inactive enhancers display higher levels of DNA methylation, whereas hypomethylation of enhancer is associated with transcription factor binding and subsequent transcriptional activation [28–30]. As can be seen from Fig. 2B, there was an overall hypomethylation pattern in GBM, and we identified far more genes that might be regulated by the hypomethylation enhancer regions than those that might be regulated by the hypermethylation enhancer regions. Besides, Studies have shown that a variety of cancers are identified many more hypomethylated enhancers than hypermethylated enhancers [22, 25]. Therefore, we focused on hypomethylated enhancer regions and conducted further research on them.

Despite tumorigenesis is a complicated dynamic process, recent studies proved that the dysregulation of target genes plays critical and complex roles during the development of tumors [31]. To evaluate the biological characteristics of these target genes that might be regulated by hypomethylated enhancer regions, the mRNAs in the hypo-EMTRN were taken to implement the function enrichment analysis. In this study, the top 20 Gene Ontology (GO) functional terms and pathways of enrichment results were displayed according to *p* value and gene enrichment ratio (Fig. 3B, C, Additional file 2: Fig. S2B and S2C). A sum of 680 GO terms and 34 Kyoto Encyclopedia of Genes and Genomes (KEGG) pathways were identified to be associated with these target genes (Additional file 6: Table S3). The results showed that the enriched GO terms were involved in cellular process (such as GO:0048522), cell migration (such as GO:2001224), cell adhesion (such as GO:007045), cell differentiation (such as GO:0030182 and GO:0030099), cellular metabolic process (GO:0031324 and GO:0051253), and other biological processes (BP), which were deeply correlated with the progression of GBM development [32, 33]. Epigenetic modifications to the genome, especially DNA methylation and histone modifications, affect gene expression causing increased risk for cancers and other diseases. Previous study [34] have shown that methylation of lysine occurs on two different histones (H3 and H4), and exists at six different sites between the two histones (H3K4, H3K9, H3K27, H3K36, H3K79 and H4K20). This study found that the significantly enriched GO terms of these target genes were related to histone H3-K4 demethylation (GO: 0034720) and H3-K79 methylation (GO: 2001160). The results suggested that the changes in the histone methyltransferase activity might regulate the expression of related target genes in GBM. Interestingly, we also found that the significantly enriched GO terms in these target genes included some biological processes of demethylation (such as GO:1901537 and GO:0070076), which might be related to the regulation of hypomethylated enhancer regions.

For the KEGG pathway analysis, we found that these target genes that might be regulated by hypomethylated enhancer regions were enriched in some GBM-related KEGG pathways. As the significantly enriched pathway (hsa04020, hsa04724) in this study, Afshari et al. [35] showed that calcium signaling pathway is involved in the processes of cell proliferation, metastasis, angiogenesis, migration, and invasiveness. Moreover, glutamatergic and calcium signaling may promote glioblastoma formation by metabolic reprogramming and genetic switching or upregulate the levels intracellular Ca^2+^ to increase glutamate release [36]. The role of ErbB [epidermal growth factor receptor (EGFR)] in GBM and glioma has also been extensively studied. EGFR could encourage tumor progression by promoting angiogenesis and cell invasion in GBM, and EGFR amplification could be a marker that played a role in prognostication, treatment, clinical trial eligibility [37, 38]. In this study, ErbB signaling pathway (hsa04012) was also an important pathway enriched by us.

### Identification of highly synergistic, competitive modules and prognostic related biomarkers

To further investigate the modularity feature of hypo-EMTRN and how lncRNAs and mRNAs synergized with each other. In this study, we used a novel maximal biclique enumeration algorithm to extract synergetic lncRNA–mRNA competitive modules. The maximal biclique enumeration algorithm is to find all maximal bicliques in a bipartite graph and generate both edge maximum and vertex maximum bicliques [39]. A biclique module is a complete bipartite graph in which an edge is realized from every vertex of a miRNA set to every vertex of a target gene set. In total, 2651 synergistic, competitive modules were identified from hypo-EMTRN. Subsequently, to evaluate whether these modules were prognostic factors for GBM, we performed survival analysis on these 2651 modules. Finally, 22 modules could significantly classify GBM patients into high and low risk groups in both the training set and the test set (Additional file 7: Table S4).

**Fig. 4:**
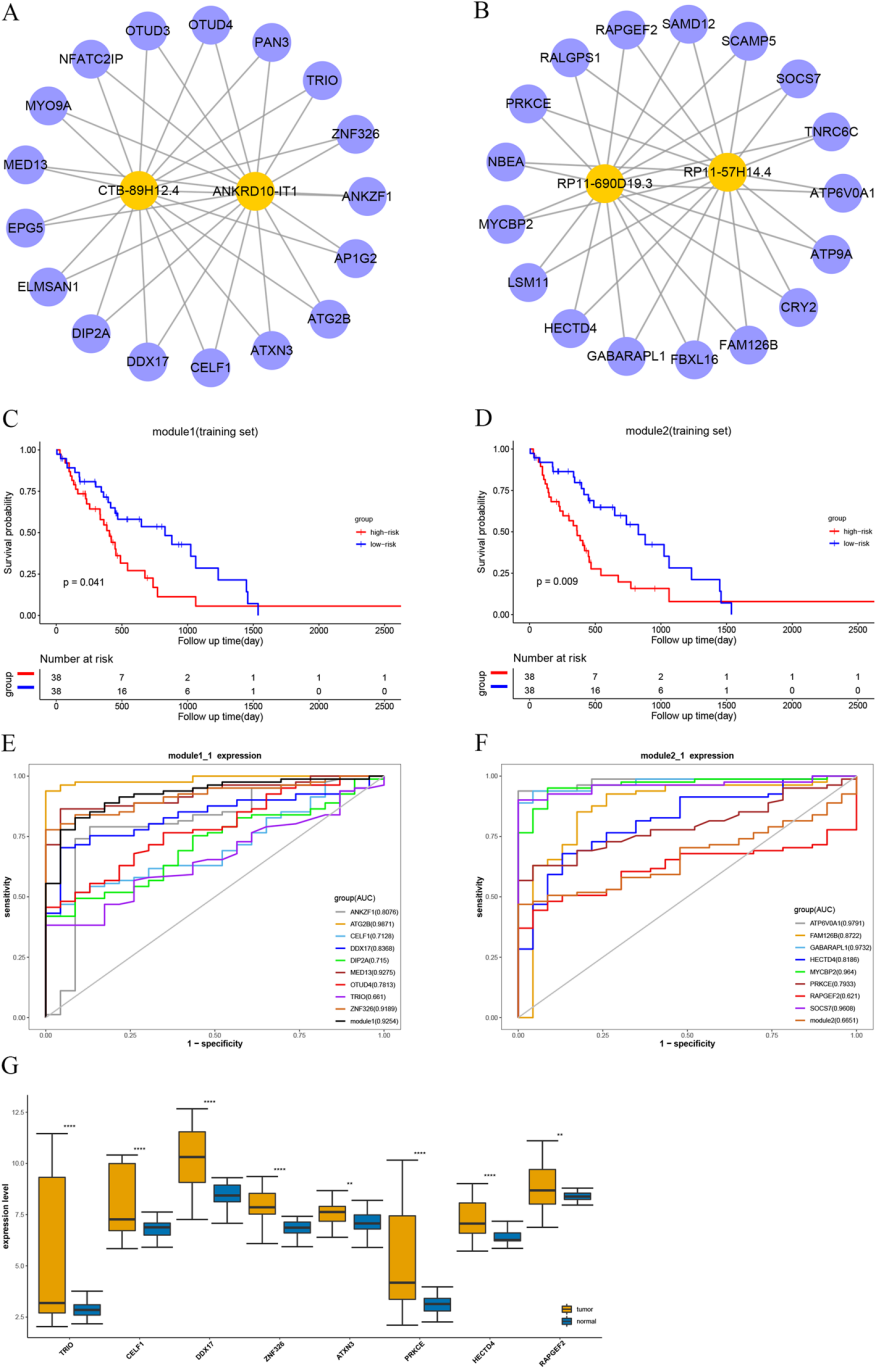
Gene co-expression modules associated with GBM. **A** Visualization of the lncRNA-mRNA co-expression network of module 1. **B** Visualization of the lncRNA-mRNA co-expression network of module 2. **C** Survival analysis curves of the module 1 in the training set. **D** Survival analysis curves of the module 2 in the training set. **E** Receiver operating characteristic analysis of some genes in module 1. **F** Receiver operating characteristic analysis of some genes in module 2. **G** Boxplots are presented with comparisons of expression levels between GBM and normal samples of hallmarks

Previous research discovered that hub genes play essential roles in networks, and the degree of the node in the top 10–20% of the network are usually defined as hub nodes [40]. Our results showed that most of the genes in module 1 (Fig. 4A) and module 2 (Fig. 4B) had high degrees in the hypo-EMTRN. As shown in Fig. 4C, D, patients in the low‐risk group had significantly longer overall survival time than those in the high‐risk group. The diagnostic value of the modules was further appraised to see whether they could be used as cancer biomarkers for early diagnosis of GBM. In order to ensure the accuracy of the research results, we collected an independent dataset of genome-wide lncRNA and mRNA expression level (GSE4290) in GBM to perform Receiver Operating Characteristic (ROC) curve analysis for the two modules by using the “ROC” function in the pROC package [41]. The overall area under the ROC curve of the diagnostic potential of module 1 and module 2 in GBM was 0.9286 and 0.6651, respectively (Fig. 4E, F). The result indicated that modules might effectively discriminate tumor samples from normal ones, and genes in modules might be potential diagnostic cancer hallmarks for GBM. Salhia et al. [42] reported that the expression of TRIO was upregulated in GBM tissues, and depletion of TRIO markedly suppressed cell migration and invasion. The present study found that TRIO was also up-regulated in module 1, and its increased expression in GBM might be affected by the corresponding hypomethylated enhancer regions. Some studies have reported that CELF1, DDX17 and ZNF326 are overexpressed in glioma [43–45]. Additionally, ATXN3 was highly expressed in breast cancer and it promotes tumor tissue metastasis by deubiquitinating and stabilizing KLF4 [46]. These genes were also highly expressed in module 1 in our study, but their expression status in GBM has not been reported. PRKCE kinase is involved in many different cellular functions, such as neuron channel activation, apoptosis, cardioprotection from ischemia, heat shock response, and insulin exocytosis [47]. Moreover, PRKCE is associated with prognosis of GBM. Wan et al. [48] found that HECTD4 was prominently elevated in CHOL tissues. Zhang et al. [49] used bioinformatics analysis to identify RAPGEF2 as potential target genes in the Wnt and MAPK signaling pathways of Medulloblastoma. These genes were also highly expressed in our study, but their expression status in GBM has not been reported. The expression of these genes had a synergistic effect with lncRNAs, and their expression might also be influenced by the high expression of lncRNA. More importantly, their expression might be simultaneously regulated by several hypomethylated enhancer regions. As shown in the boxplot, this study validated the expression states of these genes were also upregulated in GBM tissues compared to normal brain tissues (Fig. 4G). The result suggested that their upregulation could be used as a reliable clinical predictor for tumor diagnosis and to predict survival in patients with GBM. Hence, CELF1, DDX17, ZNF326, ATXN3, HECTD4, RAPGEF2 are promising diagnostic biomarkers specific for GBM.

### Prediction of small molecule drugs for GBM treatment

**Fig. 5:**
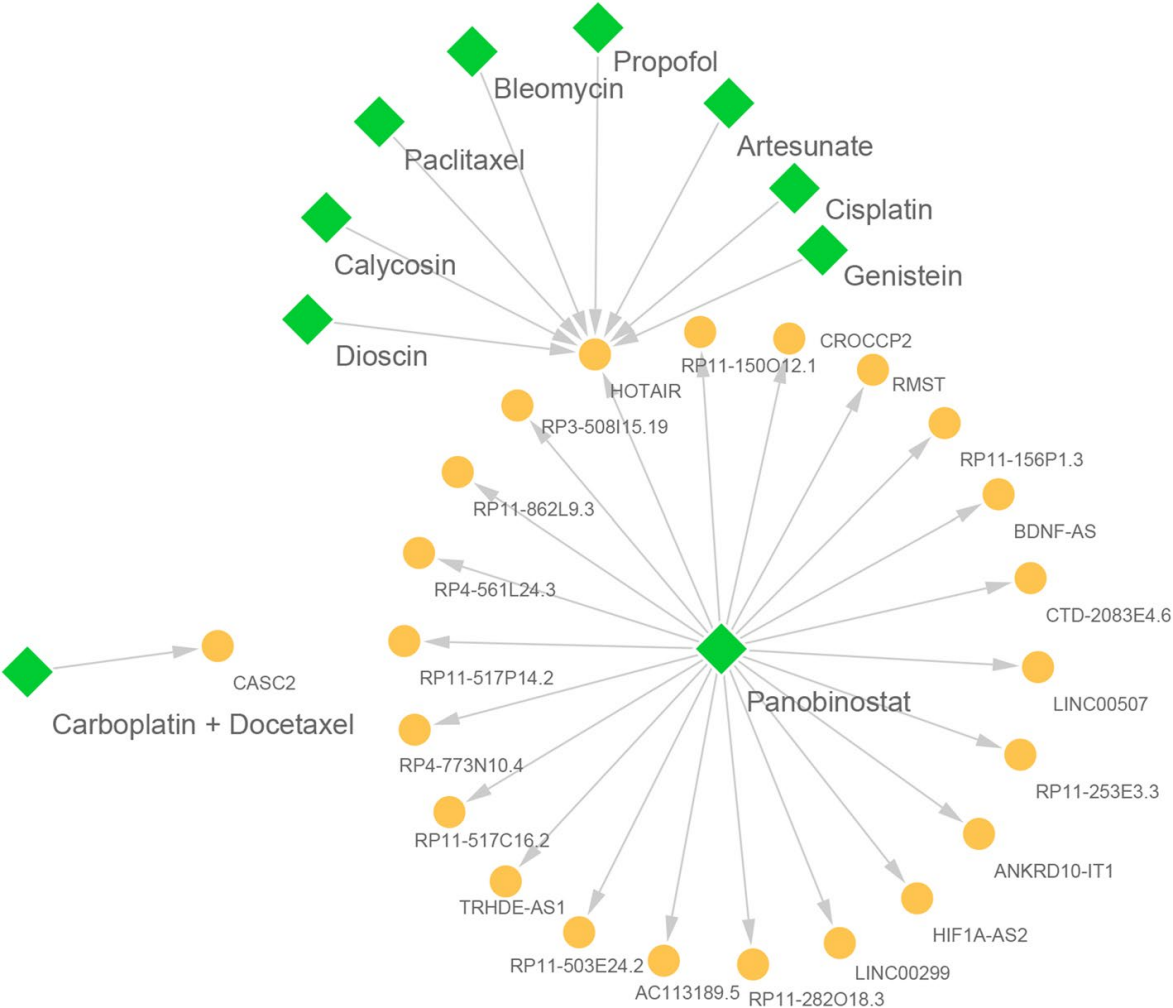
Construction of the drug-target network based on the lncRNAs regulated by hypomethylated enhancer regions

As precision medicine becomes increasingly relevant in healthcare, the field of pharmacogenomic also continues to gain prominence in the clinical setting [50]. Meanwhile, multiple studies have demonstrated that small molecule drugs can modify lncRNA expression, which suggests a promising therapy for human diseases [51, 52]. Thus, based on the hypo-EMTRN and the information in D-lnc, we inferred that some potential drugs could be used for the treatment of GBM patients by constructing the drug-target association network targeting lncRNAs (Fig. 5, described in the methods for details). Totally, we obtained 11 candidate drugs and 23 lncRNAs in the drug-target association network. In this network, these potential drugs could achieve the purpose of treatment by inhibiting the expression of the corresponding lncRNA. For example, Panobinostat can down-regulate the expression of multiple lncRNAs (down-regulated HIF1A-AS2, ANKRD10-IT1, BDNF-AS), genistein and Propofol can down-regulate the expression of HOTAIR. Previous studies have shown that panobinostat exposure induces aneugenicity, clastogenicity, oxidative DNA damage, DNA hypomethylation, and down-regulation of repair gene expression [53]. Javier De La Rosa et al. [54] found that Panobinostat could be used in combination with other drugs to reduces clonogenicity and induces apoptosis in glioblastoma cells. Besides, experiments have demonstrated that treatment of breast cancer MCF-7 cells with genistein resulted in decreased phosphorylation of Akt, and decreased expression of HOTAIR [55]. It was deduced that Propofol might be a novel potential small-molecule treatment for GBM. In this study, we identified some biomarkers of GBM. HOTAIR is a biomarker for multiple cancers and is highly expressed in cancer tissues compared to normal tissues [55, 56]. Ting Ma et al. revealed that Diosgenin inhibits gastric tumor proliferation through regulating the high expression of lncRNA HOTAIR [55]. There was also a study that showed that HOTAIR levels in serum samples from GBM patients was significantly higher than in the corresponding controls and it could be a novel diagnostic and prognostic biomarker in GBM [57]. In addition, Su et al. [58] elucidated that overexpression of BDNF-AS inhibited the proliferation, migration, and invasion, as well as induced the apoptosis of glioblastoma cells. The expression level of BDNF-AS in GBM was consistent with our study. We inferred that the high expression of these genes in GBM is regulated by hypomethylated enhancer regions. The expression of HIF1A-AS2, LINC00507, LINC00299 and RMST in GBM was not reported in any study and these genes could be Clinical diagnosis and prognostic biomarker in GBM.

## Discussion

With the advancement of high-throughput sequencing technology, the amount of genome-wide methylation data in public databases has increased exponentially, providing sufficient data for screening ideal diagnostic biomarkers. Meanwhile, epigenetic regulation has become a hotspot in biomedical research, especially DNA methylation. Abnormal changes in DNA methylation are considered to be one of the most powerful means for the development of tumor diagnosis, prognosis and predictive biomarkers [59]. Generally, DNA methylation-based biomarker studies has mainly been focused on the effects of hypermethylation of promoter in tumor suppressor genes, and there are few studies on enhancer methylation [60]. To our knowledge, the methylation dynamic in enhancers is still unclear so far. Thus, we systematically analyzed the methylation dynamics in enhancers in GBM.

In this study, we characterized genome-wide aberrant enhancer region methylation patterns in GBM based on multi-omics data. 7024 hypermethylated DMERs and 37,817 hypomethylated DMERs were identified by differential methylation analysis. From this result, we can see that the hypomethylated enhancer might be the main regulator of gene expression and they more often correlates with gene expression than hypermethylation. Activated enhancers are characterized by increased H3K27ac levels surrounding the enhancers and will lead to elevated transcription of their target genes. The activated K-M enhancer appears to overcome the promoter hypermethylation and drives the MGMT expression in GBM. Moreover, deletion of the K-M enhancer reduces MGMT and Ki67 expression, decreases cell proliferation, and sensitizes cells to TMZ to a clinically relevant level [61]. Therefore, it is critical to study the interplay between DNA methylation of enhancers and their effects on the regulation of target gene expression. Moreover, the change of DNA methylation pattern is one of the first detectable tumor-specific changes associated with tumorigenesis [62]. It is suggested that our results are valuable for the identification of tissue-specific biomarkers in GBM.

Next, we constructed a co-expression network regulated by the hypomethylated DMERs, in which there were 3095 lncRNA-mRNA relationship pairs. It can be clearly seen from hypo-EMTRN that most lncRNAs are hub nodes, indicating that lncRNAs play an important role in gene expression regulation. For example, the high expression of LINC01094 in the hub node of this network has been reported to promote the growth and invasion of GBM cells [63]. The results suggested that the lncRNAs screened in the present study might be biomarkers of GBM prognosis. And our analysis on the co-expression of genes would give insight into additional layers of regulation of the lncRNA-mRNA association network. Genes play a regulatory role through different biological functions and signaling pathway networks. We wondered how important roles these target genes whose expression might be affected by hypomethylated enhancer regions played in the pathogenesis of GBM. Through enrichment analysis, we found that the target genes in the hypo-EMTRN participated in many biological processes and pathways related to tumorigenesis and progression in GBM, such as thyroid hormone signaling pathway (hsa04919), insulin secretion (hsa04911), Wnt signaling pathway (hsa04310). Previous study has shown that the disturbances in the thyroid hormone signaling may activate growth and proliferation of neoplastic cells, and would inhibit processes of differentiation and apoptosis [64]. In addition, thyroid hormones directly and indirectly stimulate the process of angiogenesis in GBM [65]. Gong et al. [66] has suggested that insulin may promotes survival and proliferation of glioblastoma by activating the downstream Akt signaling the and the InsR/IGF1R pathway in tumor cells. Lee et al. [67] suggested that Wnt signaling is aberrantly activated in GBM and that it promotes GBM growth and invasion via the maintenance of stem cell properties. These results help to understand the occurrence and development of glioblastoma to some extent.

Following, we used a maximal biclique enumeration algorithm to identify synergistically competitive modules. 22 modules can significantly classify patients into high-risk and low-risk groups in both the test set (Additional file 3: Fig. S3 A and B). In addition to the genes in module 1 and module 2 mentioned in the results, the remaining genes in these two modules were also likely to become novel biomarkers. As we can see, the overall area under the ROC curve of these genes in GBM greater than 0.9, such as ANKRD10-IT1, AP1G2, CRY2, SAMD12 (Additional file 3: Fig. S3C and D). Finally, the drug-target association network was constructed to provide potential small molecule drugs and targets for the precise treatment of GBM. We found that HOTAIR expression is regulated by a variety of small molecule drugs in the drug-target network. Cisplatin is a chemotherapeutic drug used for treating numerous human cancers, such as prostate cancer, ovarian cancer and bladder cancer [68–70]. In recent years, studies have found that Cisplatin has been shown to be effective in combination with other drugs in treating patients with GBM [71–73]. It was inferred that cisplatin might downregulate the expression of HOTAIR. Additionally, as a well-known antimalarial drug, artesunate has clear side effects, and recently it has been reported to have antitumor effects. Although studies have shown that Artesunate can inhibit the overexpression of HOTAIR and thereby reduce the metastasis of cervical cancer cells [74]. At the same time, artesunate could significantly reduce the clonal formation ability and proliferation of glioblastoma cells by arresting cell cycle [75]. However, no studies have shown which gene expression can be regulated by artesunate, and our study found that artesunate may be a drug to inhibit high expression of HOTAIR.

Unfortunately, this study has some limitations that need to be highlighted. Since GBM is different from general tumors, sample acquisition is a problem, and this deficiency is likely to have affected the final results to some degree. Moreover, the mechanistic results from the current study were based on bioinformatics analysis. Meanwhile, the lack of analyzing the hypermethylated enhancer regions was a limitation to the present study. Due to technical and time constraints, we did not validate our results in animal models of GBM and brain tissues from patients with GBM. Future functional investigations and molecular experiment are still required to explore the mechanisms underlying the roles of novel biomarkers.

## Conclusion

In summary, we successfully constructed the lncRNA-mRNA regulatory network identified by analysis of DMERs in multi-omics data and confirmed that the deregulation of enhancers might lead to tumorigenesis. Furthermore, we identified survival prognostic modules by analyzing the genome‐wide lncRNA and mRNA expression profiles. The modules could serve as potential prognostic indicators alone or in combination with other clinicopathological for patients with GBM. Besides, the identified genes could be further evaluated for use as cancer biomarkers. Meanwhile, our study provides an insight into the discovery of potential drug targets for GBM treatment.

## Methods

### Data source and pre-processing

The DNA methylation data (level 3) generated from HM450K platform was downloaded from GEO database for all samples, with 136 tumor samples (GSE36278) and 58 normal samples (GSE42861). The methylation level of each probe was represented by the β-value (from 0 to 1). $Beta\, value = I_{meth} /I_{meth} + I_{unmeth}$, where $I_{meth}$ is the intensity of methylation and ${I}_{unmeth}$ is the intensity of unmethylation. To ensure the accuracy of methylation level, we removed CpG sites with missing value > 30% of samples. Then, we used the k-nearest neighbors method [76] with the knnImputation function in the ‘DMwR’ package for filling the missing value of methylation data. Clinical data of patients and expression data of 136 tumor samples were downloaded from GDC Data Portal. The expression level was quantified as fragments per kilobase per million reads mapped (FPKM). The expression profile of the validation dataset (GSE4290) was downloaded from GEO database, with 81 tumor samples and 23 normal samples.

The annotation file for lncRNAs and mRNAs was derived from GENCODE database [77]. The experimental interactions between lncRNAs and miRNAs were collected from the starBase v2.0 [78], LncBase v2 [79] and RNAInter database [80]. Human miRNAs and their targets were downloaded from starBase v2.0, miRTarBase (release 8.0) [81] and RNAInter database. These databases store manually curated collections of experimentally supported miRNA targets. The drug target information was downloaded from D-lnc [82].

### Construction of enhancer region and promoter region

In order to obtain a more comprehensive probes located in the enhancer region, the probes were derived from two parts: first, the GPL13534 (the platform file for the HM450K data we used) Comment file in GEO database contains the enhancer probe information, and a total of 102,559 enhancer probes were obtained; second, in the previously published literature, a total of 102,499 enhancer probes were annotated from the HM450K probe by Yao et al., and these probes have been used in enhancer-related research [83]. We merged the two parts of the probes and then removed the duplicated probes. Finally, we obtained 161,708 enhancer probes for subsequent analysis. Previous studies have suggested that the median size of the typical enhancer is around 1000 bp [84, 85]. Therefore, we constructed intervals using a window of 500 bp directly upstream of and downstream from the CpG coordinate. Overlapping intervals were joined, and extended into a larger interval. Then, the average value of enhancer probes in enhancer region was calculated as the DNA methylation level of enhancer region.

Since genes are regulated not only by enhancer methylation but also by promoter methylation. To obtain genes regulated by aberrant enhancer methylation, we needed to reannotate the promoter region. Since the regulatory mechanism of lncRNA was similar to the mRNA in promoter, the region 2 kb upstream from TSS of genes was regarded as the promoter region and the DNA methylation probes in promoter regions were obtained [86, 87]. Then we deleted the enhancer probes in promoter region and probes which mapped to more than one gene. Next, the DNA methylation level of a gene was defined as the average -values of probes that mapped to its promoter region.

### Identification of genes regulated by promoter methylation

We used the R package ‘limma’ [88] designed based on the generalized linear model to identify DPMGs between the tumor and normal samples with an adjusted *p* value ≤ 0.05 and the difference of median DNA methylation level between the tumor and normal samples ≥ 0.1. The *p* value was adjusted using the Benjamini–Hochberg (BH) method [89]. Similarly, we also identified DMERs for further analysis. We selected the enhancer regions with log_2_(FC) ≥ 0.01 and adjusted *p* value ≤ 0.05 as hypermethylated enhancer regions, and those with log_2_(FC) ≤ − 0.01 as hypomethylated enhancer regions. Meanwhile, PCC was calculated for each DPMG between the methylation value and the corresponding expression value. We obtained target genes that might be regulated by differential promoter methylation by retaining genes that were significantly negatively correlated (PCC < 0 and *p* value ≤ 0.05).

### Identification of target genes regulated by DMERs

To obtain genes that are only regulated by enhancer methylation, we removed genes whose expression is regulated by promoter methylation. We calculated the distance between the central site of DMER and TSS of lncRNA or mRNA. Previous research suggested that the greatest known distance between an enhancer and a gene was about 1 Mbp [90]. In addition, it is difficult to know which gene is regulated by each enhancer, since enhancers can work remotely in any orientation and do not necessarily regulate the closest gene [22]. Therefore, we selected DMER-lncRNA and DMER-mRNA pairs located on the same chromosome, with a maximal linear distance of 1 Mbp between the center [91]. We used PCC to calculate the correlation between the DMER and gene expression. Gene expression is negatively regulated by enhancer methylation, and the hypermethylated enhancer region can downregulate or even silence gene expression, while the hypomethylated enhancer region tends to activate gene expression [15, 92]. So we retained only negatively correlated DMER-lncRNA pairs and DMER-mRNA pairs with *p* value ≤ 0.05.

### Regulatory network construction and visualization

We collected and integrated 69,622 non-redundant lncRNA-miRNA interactions and 795,761 miRNA-mRNA pairs from multiple databases. A total of 4,563,164 pairs between lncRNAs and mRNAs that shared with the same miRNA were obtained.

The EMTRN was constructed as follows: First, a lncRNA-mRNA pair which interacted with more than one same miRNA and whose hypergeometric test based on lncRNA-miRNA pair and mRNA-miRNA pair was significant (false discovery rate (FDR) ≤ 0.05) was considered as a candidate interaction pair. These candidate interaction pairs formed the background network needed for the research. Second, we matched the above relationship pairs with the lncRNA and mRNA pairs regulated by the differential enhancer methylation regions. 308,314 lncRNA-mRNA pairs were screened for further analysis. Third, the PCC of each lncRNA-mRNA pair was calculated based on the lncRNA and mRNA expression profiles. A previous study has indicated that increased lncRNA expression can enhance corresponding mRNA expression [93]. Therefore, PCC > 0.5 and *p* value ≤ 0.05 were used as thresholds to screen out 3271 lncRNA-mRNA pairs. Finally, the lncRNA-mRNA pairs were used to construct the lncRNA-mRNA network which was visualized through the software Cytoscape [94].

### Functional prediction of genes regulated by DMERs

As the functional prediction of lncRNAs is hampered by the shortage of annotated information, functional annotation analysis of lncRNAs has frequently been conducted based on the guilt by association principle [95]. The mRNAs co-expressed with lncRNAs in the regulatory network were used to perform functional enrichment analysis. GO and KEGG pathway enrichment analyses were performed to identify the significantly enriched GO terms and pathways, using the R package ‘clusterProfiler’ [96]. The BP, cell components (CC), molecular function (MF), and KEGG pathways of mRNAs were retrieved with a cut‐off criterion of *p* value ≤ 0.01 and visualized by the R packages ‘ggplot2’.

### Identification of modules associated with GBM prognosis

The regulatory network of lncRNA-mRNA in this study is a typical bipartite graph. By identifying the largest binary group in these regulatory relationships, we could find lncRNAs and mRNAs which are more closely and cooperatively regulated. So we adopted maximal biclique enumeration algorithm to identify synergistically competitive modules by using package ‘biclique’ [39]. The module was a complete bipartite graph in which all lncRNAs are connected with all mRNAs. We set the least number of nodes in the mRNA or lncRNA set to two.

To identify the clinical effect of modules, the patients were randomly divided into a training set and a test set, based on all the expression profile data (the sample sizes were the same in both groups). A cox proportional hazards regression model was fitted to evaluate the association between the expression profile of genes in the module and patient survival in GBM. The risk score was adopted to classify the risk groups, as follows: $risk score={\beta }_{1}{X}_{1}+{\beta }_{2}{X}_{2}+\cdots +{\beta }_{i}{X}_{i}$, where ${\beta }_{i}$ was the cox regression coefficient for ${gene}_{i}$ and ${X}_{i}$ was the expression level of ${gene}_{i}$ in a corresponding patient. The median risk score was used as a cut-off to divide patients in the training set into high- and low-risk groups. This risk score model and cut-off point were also applied to the test set to divide the patients into high- and low-risk groups. Kaplan–Meier survival analysis and the log-rank test (*p* ≤ 0.05) were performed to estimate the survival difference between the two patient groups.

### Prediction of small molecule drugs for GBM treatment

D-lnc is a comprehensive platform that can detect the modification of drugs on lncRNA expression [82]. It contained 4960 experimentally validated lncRNA-drug regulatory association for Homo sapiens. Since the expression of lncRNA in hypo-EMTRN might be up-regulated by the influence of hypomethylated enhancer regions, we screened out and integrated non-redundant 2366 lncRNA-drug associations and the drugs in these associations could downregulate the lncRNA expression. Then, we matched the 2366 lncRNA-drug associations in D-lnc with the lncRNAs in hypo-EMTRN to obtain the drug-target association network. Finally, the drug-target association network was constructed and illustrated by the Cytoscape software. All analyses were performed using R 4.0.2 software.

## Supplementary Information

**Additional file 1**. **Figure S1** Workflow of core process in the study. (A) Workflow of identifying target genes that might be regulated by DMERs. (B) Workflow of construction of EMTRN. EMTRN, enhancer region methylation-mediated target gene regulatory network.

**Additional file 2**. **Figure S2** Construction of cancer-related hallmarks in regulatory network and Functional enrichment analysis. (A) lncRNA-mRNA co-expression network regulated by hypermethylated enhancer regions. The node degree is indicated by the node size. lncRNA, long non-coding RNA. (B) The top 20 enriched CC items of downregulated genes. CC, cellular component. (C) The top 20 enriched MF items of downregulated genes. MF, molecular function.

**Additional file 3**. **Figure S3** Kaplan–Meier curves and receiver operating characteristics of modules 1 and 2. (A) Survival analysis curves of the module 1 in the testing set. (B) Survival analysis curves of the module 2 in the testing set. (C) Receiver operating characteristic analysis of some genes in module 1. (D) Receiver operating characteristic analysis of some genes in module 2.

**Additional file 4**. **Table S1**. lncRNA-mRNA regulated by the hypermethylation enhancer regions.

**Additional file 5**. **Table S2**. lncRNA-mRNA regulated by the hypomethylation enhancer regions.

**Additional file 6**. **Table S3**. GO terms and KEGG pathways.

**Additional file 7**. **Table S4**. Prognostic modules regulated by hypomethylation enhancer regions in GBM.

## Data Availability

The datasets used and/or analyzed during the present study are available from the corresponding author on reasonable request.

## Abbreviations

GBM: Glioblastoma multiforme
DMER: Differential methylation enhancer region
GSCs: GBM stem cells
FPKM: Fragments per kilobase per million reads mapped
TSS: Transcription start site
DPMGs: Differential promoter methylation genes
BH: Benjamini–Hochberg
PCC: Pearson correlation coefficient
GO: Gene ontology
KEGG: Kyoto encyclopedia of genes and genomes
BP: Biological processes
CC: Cell components
MF: Molecular function
EMTRN: Enhancer methylation-mediated target gene regulatory network
hypo-EMTRN: Enhancer hypomethylation-mediated regulatory network
hyper-EMTRN: Enhancer hypermethylation-mediated regulatory network
ROC: Receiver operating characteristic
