# Supplementary figures and images for: The regulatory pattern of target gene expression by aberrant enhancer methylation in glioblastoma
Source: BMC Bioinformatics. 2021 Sep 5;22:420. doi: 10.1186/s12859-021-04345-8 (PMC8420065; doi:10.1186/s12859-021-04345-8)

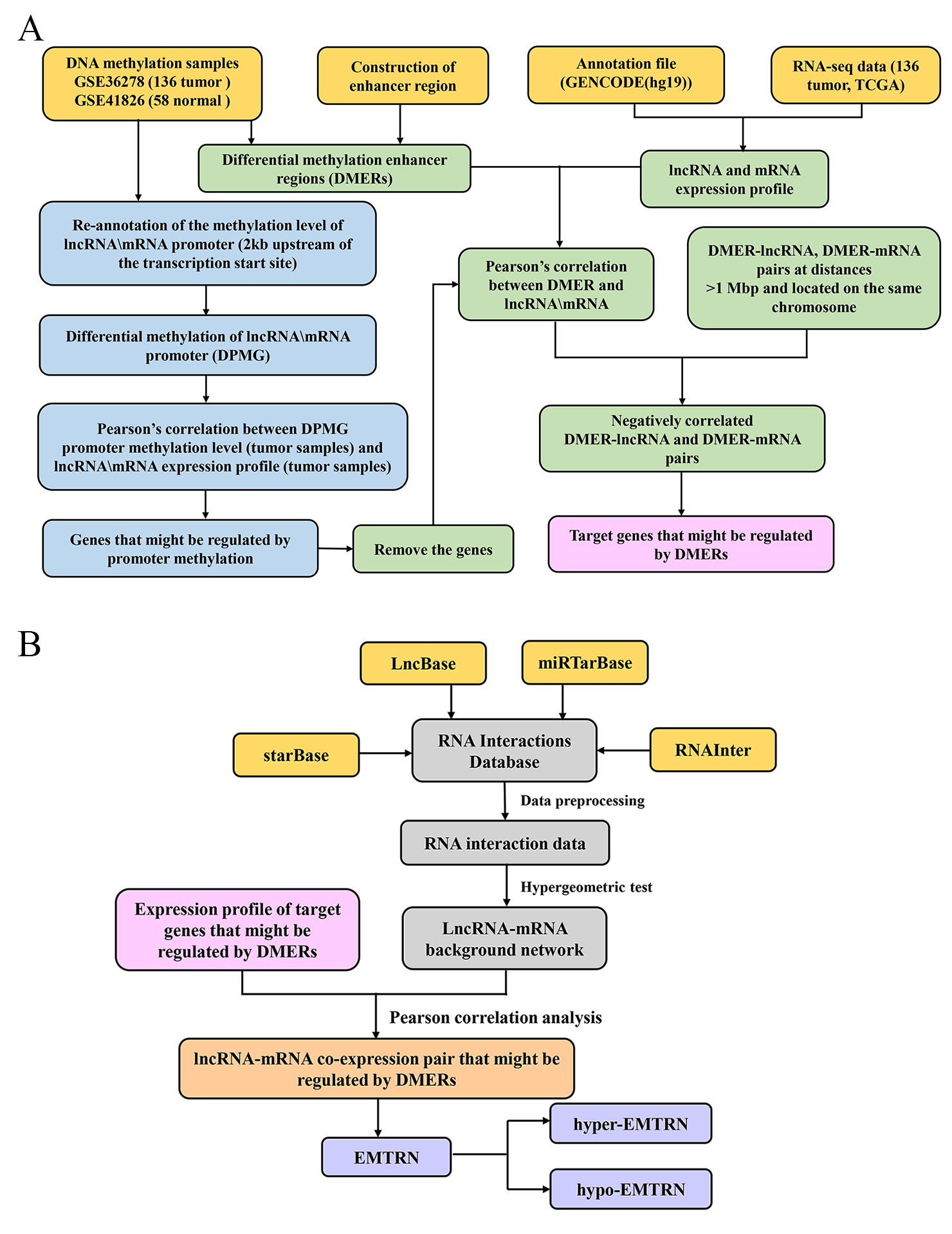

Supplement: Supplementary file 1 — Additional file 1. Figure S1 Workflow of core process in the study. (A) Workflow of identifying target genes that might be regulated by DMERs. (B) Workflow of construction of EMTRN. EMTRN, enhancer region methylation-mediated target gene regulatory network. [file 12859_2021_4345_MOESM1_ESM.tif]

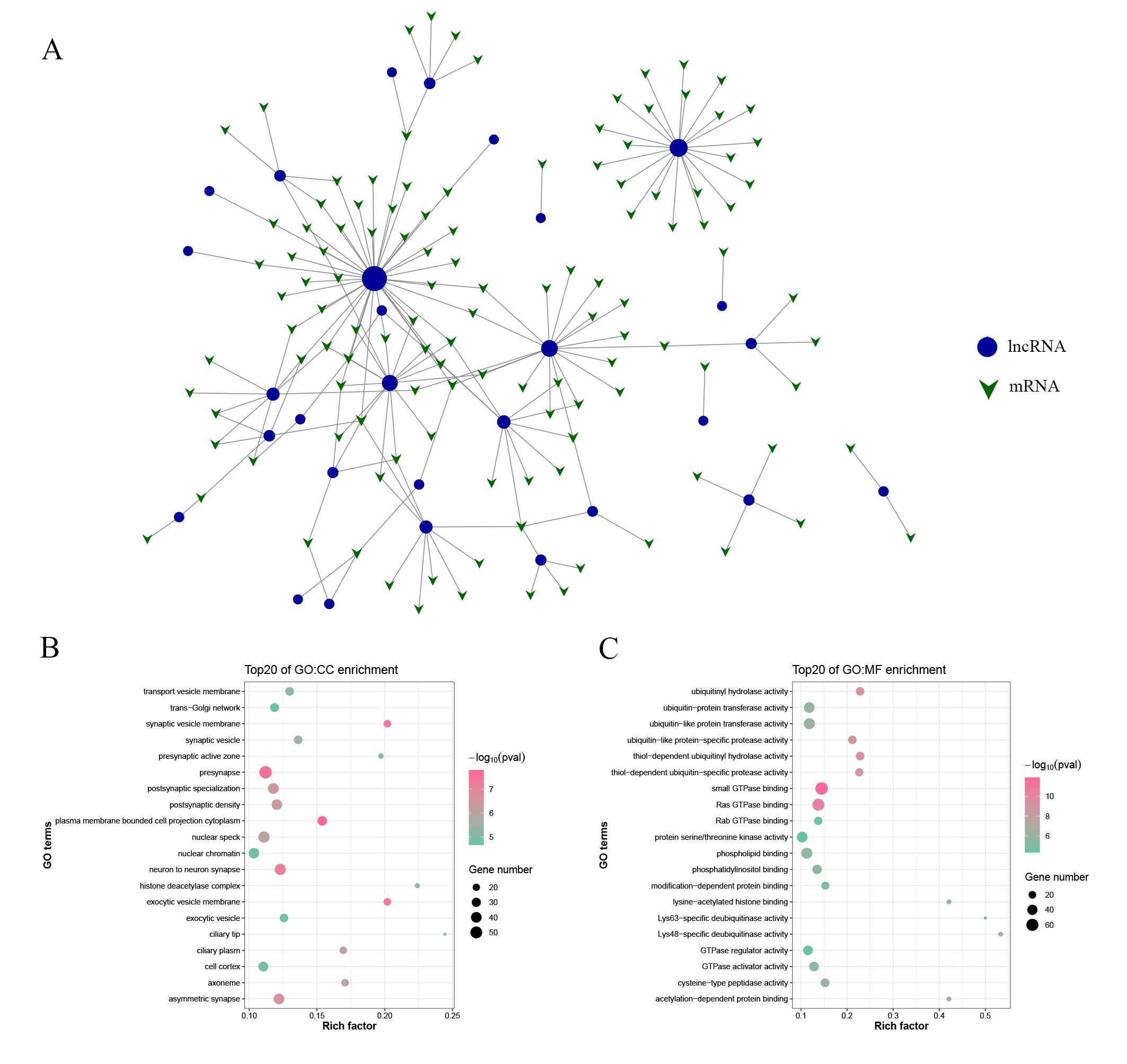

Supplement: Supplementary file 2 — Additional file 2. Figure S2 Construction of cancer-related hallmarks in regulatory network and Functional enrichment analysis. (A) lncRNA-mRNA co-expression network regulated by hypermethylated enhancer regions. The node degree is indicated by the node size. lncRNA, long non-coding RNA. (B) The top 20 enriched CC items of downregulated genes. CC, cellular component. (C) The top 20 enriched MF items of downregulated genes. MF, molecular function. [file 12859_2021_4345_MOESM2_ESM.tif]

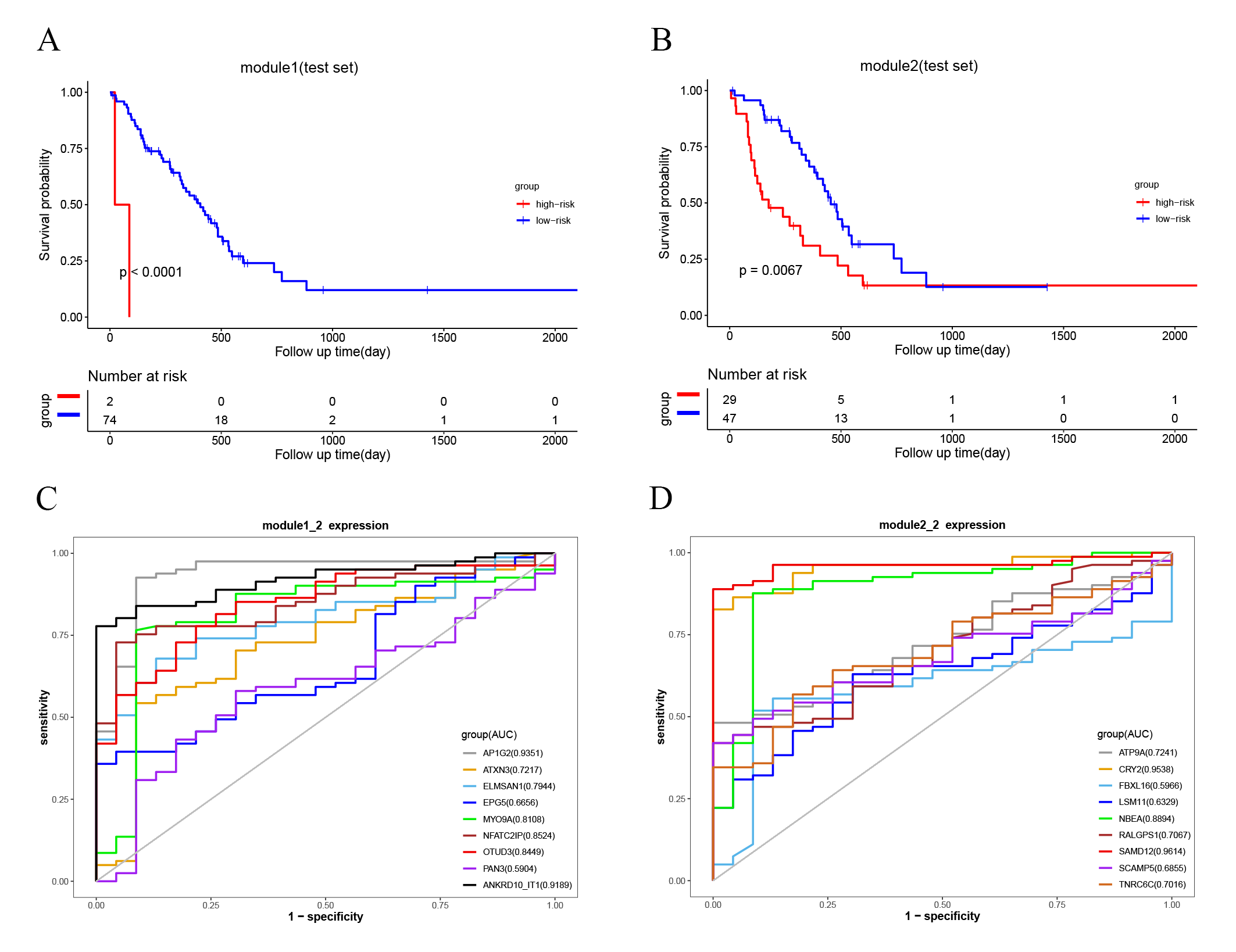

Supplement: Supplementary file 3 — Additional file 3. Figure S3 Kaplan–Meier curves and receiver operating characteristics of modules 1 and 2. (A) Survival analysis curves of the module 1 in the testing set. (B) Survival analysis curves of the module 2 in the testing set. (C) Receiver operating characteristic analysis of some genes in module 1. (D) Receiver operating characteristic analysis of some genes in module 2. [file 12859_2021_4345_MOESM3_ESM.tif]
