# Supplementary material for: The regulatory pattern of target gene expression by aberrant enhancer methylation in glioblastoma
Source: BMC Bioinformatics. 2021 Sep 5;22:420. doi: 10.1186/s12859-021-04345-8 (PMC8420065; doi:10.1186/s12859-021-04345-8)
Supplement: Supplementary file 4 — Additional file 4. Table S1. lncRNA-mRNA regulated by the hypermethylation enhancer regions. [file 12859_2021_4345_MOESM4_ESM.docx]

Table S1. lncRNA-mRNA regulated by the hypermethylation enhancer regions

| lncRNA | mRNA | PCC | PCC.pval |
| --- | --- | --- | --- |
| ENSG00000176659 | ENSG00000105383 | 0.705006603 | 9.76E-22 |
| ENSG00000176659 | ENSG00000161929 | 0.685210912 | 3.56E-20 |
| ENSG00000176659 | ENSG00000197471 | 0.532689487 | 2.47E-11 |
| ENSG00000176659 | ENSG00000115165 | 0.541956937 | 9.50E-12 |
| ENSG00000176659 | ENSG00000137462 | 0.685170943 | 3.58E-20 |
| ENSG00000176659 | ENSG00000185905 | 0.717429114 | 8.73E-23 |
| ENSG00000176659 | ENSG00000164674 | 0.520095205 | 8.63E-11 |
| ENSG00000176659 | ENSG00000105339 | 0.625359217 | 4.00E-16 |
| ENSG00000176659 | ENSG00000059377 | 0.637280702 | 7.35E-17 |
| ENSG00000176659 | ENSG00000130487 | 0.501010097 | 5.23E-10 |
| ENSG00000176659 | ENSG00000169385 | 0.544343206 | 7.40E-12 |
| ENSG00000176659 | ENSG00000146192 | 0.687740488 | 2.28E-20 |
| ENSG00000176659 | ENSG00000104894 | 0.676716447 | 1.53E-19 |
| ENSG00000176659 | ENSG00000105639 | 0.533870625 | 2.19E-11 |
| ENSG00000176659 | ENSG00000104974 | 0.782075429 | 2.64E-29 |
| ENSG00000176659 | ENSG00000129667 | 0.556504421 | 2.00E-12 |
| ENSG00000176659 | ENSG00000167895 | 0.6947077 | 6.57E-21 |
| ENSG00000176659 | ENSG00000186818 | 0.699071864 | 2.96E-21 |
| ENSG00000176659 | ENSG00000100365 | 0.64067439 | 4.47E-17 |
| ENSG00000176659 | ENSG00000168961 | 0.650866715 | 9.70E-18 |
| ENSG00000176659 | ENSG00000173372 | 0.57821724 | 1.69E-13 |
| ENSG00000176840 | ENSG00000107864 | 0.605878682 | 5.49E-15 |
| ENSG00000176840 | ENSG00000236279 | 0.739501023 | 8.58E-25 |
| ENSG00000176840 | ENSG00000177542 | 0.504174378 | 3.91E-10 |
| ENSG00000176840 | ENSG00000170231 | 0.585036269 | 7.48E-14 |
| ENSG00000176840 | ENSG00000152595 | 0.521976988 | 7.18E-11 |
| ENSG00000197182 | ENSG00000132522 | 0.514074276 | 1.54E-10 |
| ENSG00000197182 | ENSG00000104852 | 0.51209475 | 1.86E-10 |
| ENSG00000197182 | ENSG00000185958 | 0.580559943 | 1.28E-13 |
| ENSG00000213963 | ENSG00000131023 | 0.539993564 | 1.17E-11 |
| ENSG00000213963 | ENSG00000151006 | 0.539767957 | 1.19E-11 |
| ENSG00000213963 | ENSG00000020922 | 0.548091916 | 4.97E-12 |
| ENSG00000213963 | ENSG00000178252 | 0.592538977 | 2.99E-14 |
| ENSG00000213963 | ENSG00000135720 | 0.541219627 | 1.03E-11 |
| ENSG00000214106 | ENSG00000138496 | 0.558370077 | 1.63E-12 |
| ENSG00000224870 | ENSG00000107404 | 0.548793053 | 4.61E-12 |
| ENSG00000224870 | ENSG00000197785 | 0.544815934 | 7.04E-12 |
| ENSG00000224870 | ENSG00000198925 | 0.515435713 | 1.35E-10 |
| ENSG00000224870 | ENSG00000160075 | 0.506540494 | 3.14E-10 |
| ENSG00000226200 | ENSG00000107929 | 0.546754314 | 5.73E-12 |
| ENSG00000226200 | ENSG00000227345 | 0.597369048 | 1.63E-14 |
| ENSG00000226200 | ENSG00000171206 | 0.521613527 | 7.44E-11 |
| ENSG00000226200 | ENSG00000189319 | 0.558914432 | 1.53E-12 |
| ENSG00000226200 | ENSG00000165406 | 0.663016942 | 1.45E-18 |
| ENSG00000230454 | ENSG00000178252 | 0.585465016 | 7.10E-14 |
| ENSG00000230454 | ENSG00000132522 | 0.691203775 | 1.24E-20 |
| ENSG00000230454 | ENSG00000114735 | 0.766967517 | 1.35E-27 |
| ENSG00000230454 | ENSG00000108515 | 0.660409642 | 2.20E-18 |
| ENSG00000230454 | ENSG00000173540 | 0.577483175 | 1.84E-13 |
| ENSG00000230454 | ENSG00000168970 | 0.533568062 | 2.26E-11 |
| ENSG00000230454 | ENSG00000185829 | 0.542978682 | 8.54E-12 |
| ENSG00000230454 | ENSG00000176909 | 0.551534291 | 3.43E-12 |
| ENSG00000230454 | ENSG00000127804 | 0.528842342 | 3.64E-11 |
| ENSG00000232098 | ENSG00000007202 | 0.628151404 | 2.71E-16 |
| ENSG00000232098 | ENSG00000133026 | 0.514521894 | 1.48E-10 |
| ENSG00000232098 | ENSG00000104852 | 0.558385998 | 1.63E-12 |
| ENSG00000232098 | ENSG00000105443 | 0.506247499 | 3.23E-10 |
| ENSG00000232098 | ENSG00000198925 | 0.524803174 | 5.44E-11 |
| ENSG00000232098 | ENSG00000108839 | 0.521060781 | 7.85E-11 |
| ENSG00000232098 | ENSG00000275342 | 0.501968734 | 4.79E-10 |
| ENSG00000232098 | ENSG00000040199 | 0.593599058 | 2.62E-14 |
| ENSG00000232098 | ENSG00000156983 | 0.557655772 | 1.76E-12 |
| ENSG00000232098 | ENSG00000113716 | 0.51107423 | 2.05E-10 |
| ENSG00000232098 | ENSG00000099821 | 0.548016053 | 5.01E-12 |
| ENSG00000232098 | ENSG00000169221 | 0.558736325 | 1.56E-12 |
| ENSG00000232098 | ENSG00000065000 | 0.669448069 | 5.12E-19 |
| ENSG00000232098 | ENSG00000130816 | 0.630213077 | 2.03E-16 |
| ENSG00000232098 | ENSG00000198218 | 0.529522379 | 3.40E-11 |
| ENSG00000232098 | ENSG00000215883 | 0.543584587 | 8.01E-12 |
| ENSG00000232098 | ENSG00000161021 | 0.600715768 | 1.07E-14 |
| ENSG00000232098 | ENSG00000049239 | 0.519699115 | 8.97E-11 |
| ENSG00000233396 | ENSG00000104643 | 0.532221292 | 2.59E-11 |
| ENSG00000240288 | ENSG00000163660 | 0.548412355 | 4.80E-12 |
| ENSG00000244567 | ENSG00000198837 | 0.527097196 | 4.33E-11 |
| ENSG00000244567 | ENSG00000115041 | 0.529711051 | 3.33E-11 |
| ENSG00000244567 | ENSG00000040199 | 0.56253803 | 1.02E-12 |
| ENSG00000244567 | ENSG00000108587 | 0.542259539 | 9.21E-12 |
| ENSG00000244567 | ENSG00000076201 | 0.558184066 | 1.66E-12 |
| ENSG00000244567 | ENSG00000133026 | 0.577268374 | 1.89E-13 |
| ENSG00000244567 | ENSG00000214753 | 0.66062586 | 2.12E-18 |
| ENSG00000244567 | ENSG00000164715 | 0.651745276 | 8.48E-18 |
| ENSG00000244567 | ENSG00000173120 | 0.556726099 | 1.95E-12 |
| ENSG00000244567 | ENSG00000102870 | 0.550984311 | 3.64E-12 |
| ENSG00000244567 | ENSG00000185219 | 0.584520923 | 7.96E-14 |
| ENSG00000244567 | ENSG00000161202 | 0.557252459 | 1.84E-12 |
| ENSG00000244567 | ENSG00000184863 | 0.534209937 | 2.11E-11 |
| ENSG00000244567 | ENSG00000099381 | 0.583548612 | 8.95E-14 |
| ENSG00000244567 | ENSG00000178252 | 0.541657609 | 9.80E-12 |
| ENSG00000244567 | ENSG00000038219 | 0.643073312 | 3.14E-17 |
| ENSG00000244567 | ENSG00000100084 | 0.501836778 | 4.85E-10 |
| ENSG00000244567 | ENSG00000147548 | 0.606457053 | 5.10E-15 |
| ENSG00000244567 | ENSG00000140750 | 0.590553318 | 3.82E-14 |
| ENSG00000244567 | ENSG00000182944 | 0.557537665 | 1.78E-12 |
| ENSG00000244567 | ENSG00000100207 | 0.510450231 | 2.18E-10 |
| ENSG00000244567 | ENSG00000189319 | 0.59606297 | 1.92E-14 |
| ENSG00000244567 | ENSG00000131023 | 0.501623867 | 4.94E-10 |
| ENSG00000244567 | ENSG00000156983 | 0.551147153 | 3.58E-12 |
| ENSG00000244567 | ENSG00000151148 | 0.527726896 | 4.07E-11 |
| ENSG00000244567 | ENSG00000132694 | 0.545130189 | 6.81E-12 |
| ENSG00000244567 | ENSG00000094916 | 0.619491674 | 8.98E-16 |
| ENSG00000244567 | ENSG00000113319 | 0.550711983 | 3.75E-12 |
| ENSG00000244567 | ENSG00000170144 | 0.560019039 | 1.36E-12 |
| ENSG00000244567 | ENSG00000183723 | 0.580361829 | 1.31E-13 |
| ENSG00000244567 | ENSG00000104643 | 0.594740884 | 2.27E-14 |
| ENSG00000244567 | ENSG00000165406 | 0.524116352 | 5.82E-11 |
| ENSG00000244567 | ENSG00000079313 | 0.583806685 | 8.67E-14 |
| ENSG00000244567 | ENSG00000169926 | 0.638883066 | 5.82E-17 |
| ENSG00000244567 | ENSG00000116604 | 0.603358392 | 7.61E-15 |
| ENSG00000244567 | ENSG00000155111 | 0.572348369 | 3.35E-13 |
| ENSG00000244567 | ENSG00000107864 | 0.536602681 | 1.66E-11 |
| ENSG00000244567 | ENSG00000115904 | 0.614753593 | 1.70E-15 |
| ENSG00000244567 | ENSG00000107862 | 0.5920882 | 3.16E-14 |
| ENSG00000245060 | ENSG00000175348 | 0.545294445 | 6.69E-12 |
| ENSG00000245060 | ENSG00000110315 | 0.556364186 | 2.03E-12 |
| ENSG00000246477 | ENSG00000184863 | 0.517776518 | 1.08E-10 |
| ENSG00000246477 | ENSG00000147548 | 0.607735839 | 4.31E-15 |
| ENSG00000250366 | ENSG00000107864 | 0.629194673 | 2.34E-16 |
| ENSG00000253649 | ENSG00000137221 | 0.566431806 | 6.60E-13 |
| ENSG00000253649 | ENSG00000094916 | 0.548234022 | 4.89E-12 |
| ENSG00000253649 | ENSG00000020922 | 0.516673083 | 1.20E-10 |
| ENSG00000253649 | ENSG00000198218 | 0.543198292 | 8.34E-12 |
| ENSG00000253649 | ENSG00000102870 | 0.525549088 | 5.05E-11 |
| ENSG00000253649 | ENSG00000135837 | 0.505717196 | 3.39E-10 |
| ENSG00000253649 | ENSG00000009413 | 0.662176911 | 1.66E-18 |
| ENSG00000253649 | ENSG00000131023 | 0.578224473 | 1.69E-13 |
| ENSG00000253649 | ENSG00000151006 | 0.743256003 | 3.73E-25 |
| ENSG00000257702 | ENSG00000181481 | 0.536181534 | 1.73E-11 |
| ENSG00000257964 | ENSG00000184863 | 0.501320832 | 5.08E-10 |
| ENSG00000257964 | ENSG00000135837 | 0.515384099 | 1.36E-10 |
| ENSG00000259291 | ENSG00000183723 | 0.560085214 | 1.35E-12 |
| ENSG00000260267 | ENSG00000158158 | 0.504807032 | 3.69E-10 |
| ENSG00000260267 | ENSG00000108515 | 0.548394784 | 4.81E-12 |
| ENSG00000260267 | ENSG00000099381 | 0.645595877 | 2.15E-17 |
| ENSG00000260267 | ENSG00000186174 | 0.660493201 | 2.17E-18 |
| ENSG00000260267 | ENSG00000107862 | 0.553093573 | 2.90E-12 |
| ENSG00000260267 | ENSG00000184863 | 0.534404033 | 2.07E-11 |
| ENSG00000260267 | ENSG00000102870 | 0.523201245 | 6.37E-11 |
| ENSG00000260267 | ENSG00000076201 | 0.556055058 | 2.10E-12 |
| ENSG00000260267 | ENSG00000185219 | 0.560619562 | 1.27E-12 |
| ENSG00000260267 | ENSG00000168970 | 0.520116706 | 8.61E-11 |
| ENSG00000260267 | ENSG00000007202 | 0.528704677 | 3.69E-11 |
| ENSG00000260267 | ENSG00000107929 | 0.56226063 | 1.06E-12 |
| ENSG00000260267 | ENSG00000147548 | 0.575405451 | 2.35E-13 |
| ENSG00000260267 | ENSG00000198837 | 0.579290235 | 1.49E-13 |
| ENSG00000260267 | ENSG00000173120 | 0.558727666 | 1.57E-12 |
| ENSG00000260267 | ENSG00000178252 | 0.596956647 | 1.72E-14 |
| ENSG00000260841 | ENSG00000111252 | 0.598104847 | 1.49E-14 |
| ENSG00000260841 | ENSG00000167202 | 0.563860029 | 8.83E-13 |
| ENSG00000260841 | ENSG00000115594 | 0.517853067 | 1.07E-10 |
| ENSG00000260841 | ENSG00000072786 | 0.517706975 | 1.09E-10 |
| ENSG00000266088 | ENSG00000122224 | 0.518105127 | 1.05E-10 |
| ENSG00000267858 | ENSG00000106012 | 0.505873052 | 3.34E-10 |
| ENSG00000267858 | ENSG00000214021 | 0.573266705 | 3.01E-13 |
| ENSG00000267858 | ENSG00000105397 | 0.517583658 | 1.10E-10 |
| ENSG00000267858 | ENSG00000132522 | 0.571759387 | 3.59E-13 |
| ENSG00000272250 | ENSG00000129003 | 0.513093603 | 1.69E-10 |
| ENSG00000272250 | ENSG00000135720 | 0.511267297 | 2.01E-10 |
| ENSG00000272301 | ENSG00000178252 | 0.539292355 | 1.25E-11 |
| ENSG00000272301 | ENSG00000185219 | 0.519456184 | 9.18E-11 |
| ENSG00000272301 | ENSG00000168970 | 0.503746679 | 4.07E-10 |
| ENSG00000272301 | ENSG00000161547 | 0.516919412 | 1.17E-10 |
| ENSG00000272780 | ENSG00000113319 | 0.508880385 | 2.52E-10 |
| ENSG00000276248 | ENSG00000163660 | 0.527593036 | 4.12E-11 |
| ENSG00000276248 | ENSG00000040199 | 0.506851009 | 3.05E-10 |
| ENSG00000278133 | ENSG00000163660 | 0.588758369 | 4.76E-14 |
| ENSG00000278133 | ENSG00000161547 | 0.500298251 | 5.58E-10 |
| ENSG00000281207 | ENSG00000130749 | 0.508371844 | 2.64E-10 |
| ENSG00000281207 | ENSG00000198663 | 0.503891977 | 4.01E-10 |
| ENSG00000281207 | ENSG00000132522 | 0.597283169 | 1.65E-14 |
| ENSG00000281207 | ENSG00000104852 | 0.597479159 | 1.61E-14 |
| ENSG00000281207 | ENSG00000198837 | 0.552886732 | 2.97E-12 |
| ENSG00000281207 | ENSG00000104936 | 0.56665546 | 6.44E-13 |
| ENSG00000281207 | ENSG00000108839 | 0.631146646 | 1.77E-16 |
| ENSG00000281207 | ENSG00000063169 | 0.553349331 | 2.82E-12 |
| ENSG00000281207 | ENSG00000099381 | 0.5089159 | 2.51E-10 |
| ENSG00000281207 | ENSG00000147548 | 0.526298039 | 4.69E-11 |
