# Supplementary material for: The regulatory pattern of target gene expression by aberrant enhancer methylation in glioblastoma
Source: BMC Bioinformatics. 2021 Sep 5;22:420. doi: 10.1186/s12859-021-04345-8 (PMC8420065; doi:10.1186/s12859-021-04345-8)
Supplement: Supplementary file 5 — Additional file 5. Table S2. lncRNA-mRNA regulated by the hypomethylation enhancer regions. [file 12859_2021_4345_MOESM5_ESM.docx]

Table S2. lncRNA-mRNA regulated by the hypomethylation enhancer regions

| lncRNA | mRNA | PCC | PCC.pval |
| --- | --- | --- | --- |
| ENSG00000157306 | ENSG00000108669 | 0.536683601 | 1.64E-11 |
| ENSG00000157306 | ENSG00000167371 | 0.625477796 | 3.94E-16 |
| ENSG00000157306 | ENSG00000235194 | 0.709071328 | 4.49E-22 |
| ENSG00000157306 | ENSG00000184787 | 0.560271823 | 1.32E-12 |
| ENSG00000157306 | ENSG00000213983 | 0.60221972 | 8.81E-15 |
| ENSG00000157306 | ENSG00000266173 | 0.582649854 | 9.97E-14 |
| ENSG00000157306 | ENSG00000196391 | 0.571032751 | 3.90E-13 |
| ENSG00000157306 | ENSG00000164061 | 0.567210441 | 6.05E-13 |
| ENSG00000157306 | ENSG00000155744 | 0.550083836 | 4.02E-12 |
| ENSG00000157306 | ENSG00000167861 | 0.519274316 | 9.35E-11 |
| ENSG00000157306 | ENSG00000108352 | 0.721431341 | 3.90E-23 |
| ENSG00000177335 | ENSG00000108468 | 0.502539902 | 4.55E-10 |
| ENSG00000177335 | ENSG00000168014 | 0.500838186 | 5.31E-10 |
| ENSG00000177406 | ENSG00000021355 | 0.527046305 | 4.35E-11 |
| ENSG00000177406 | ENSG00000103381 | 0.513922151 | 1.56E-10 |
| ENSG00000177406 | ENSG00000111269 | 0.587243186 | 5.72E-14 |
| ENSG00000177406 | ENSG00000145779 | 0.521019458 | 7.89E-11 |
| ENSG00000177406 | ENSG00000122359 | 0.510933878 | 2.08E-10 |
| ENSG00000177406 | ENSG00000135838 | 0.502620193 | 4.51E-10 |
| ENSG00000177406 | ENSG00000184060 | 0.516189019 | 1.26E-10 |
| ENSG00000177406 | ENSG00000153071 | 0.535041873 | 1.94E-11 |
| ENSG00000177406 | ENSG00000100368 | 0.591620345 | 3.34E-14 |
| ENSG00000177406 | ENSG00000110324 | 0.519078871 | 9.53E-11 |
| ENSG00000177406 | ENSG00000138756 | 0.518887422 | 9.70E-11 |
| ENSG00000177406 | ENSG00000111885 | 0.509181736 | 2.45E-10 |
| ENSG00000177640 | ENSG00000149050 | 0.573814842 | 2.83E-13 |
| ENSG00000177640 | ENSG00000109501 | 0.521554775 | 7.48E-11 |
| ENSG00000177640 | ENSG00000129625 | 0.612601143 | 2.27E-15 |
| ENSG00000177640 | ENSG00000169379 | 0.562496435 | 1.03E-12 |
| ENSG00000177640 | ENSG00000118997 | 0.734182948 | 2.73E-24 |
| ENSG00000177640 | ENSG00000150667 | 0.628406691 | 2.61E-16 |
| ENSG00000177640 | ENSG00000119280 | 0.519933828 | 8.77E-11 |
| ENSG00000177640 | ENSG00000064652 | 0.559104064 | 1.50E-12 |
| ENSG00000177640 | ENSG00000163964 | 0.507384455 | 2.90E-10 |
| ENSG00000177640 | ENSG00000164114 | 0.566408313 | 6.62E-13 |
| ENSG00000177640 | ENSG00000188010 | 0.554033011 | 2.62E-12 |
| ENSG00000177640 | ENSG00000163521 | 0.600426195 | 1.11E-14 |
| ENSG00000177640 | ENSG00000142606 | 0.531505538 | 2.78E-11 |
| ENSG00000177640 | ENSG00000152078 | 0.511721174 | 1.93E-10 |
| ENSG00000177640 | ENSG00000197557 | 0.691352609 | 1.20E-20 |
| ENSG00000177640 | ENSG00000103599 | 0.663528024 | 1.34E-18 |
| ENSG00000177640 | ENSG00000145491 | 0.683842862 | 4.52E-20 |
| ENSG00000177640 | ENSG00000164953 | 0.560563364 | 1.28E-12 |
| ENSG00000177640 | ENSG00000141198 | 0.517690585 | 1.09E-10 |
| ENSG00000177640 | ENSG00000188316 | 0.705833743 | 8.34E-22 |
| ENSG00000177640 | ENSG00000138036 | 0.591151123 | 3.54E-14 |
| ENSG00000177640 | ENSG00000114698 | 0.571519795 | 3.69E-13 |
| ENSG00000178947 | ENSG00000139112 | 0.526577399 | 4.56E-11 |
| ENSG00000178947 | ENSG00000125814 | 0.553757064 | 2.70E-12 |
| ENSG00000178947 | ENSG00000151690 | 0.511886799 | 1.90E-10 |
| ENSG00000178947 | ENSG00000166159 | 0.555229892 | 2.30E-12 |
| ENSG00000178947 | ENSG00000162706 | 0.51779808 | 1.08E-10 |
| ENSG00000178947 | ENSG00000084764 | 0.565982156 | 6.95E-13 |
| ENSG00000178947 | ENSG00000006432 | 0.568756244 | 5.07E-13 |
| ENSG00000178947 | ENSG00000135423 | 0.521456226 | 7.56E-11 |
| ENSG00000178947 | ENSG00000088367 | 0.55944998 | 1.44E-12 |
| ENSG00000178947 | ENSG00000129990 | 0.535362469 | 1.88E-11 |
| ENSG00000178947 | ENSG00000136854 | 0.523669987 | 6.08E-11 |
| ENSG00000178947 | ENSG00000149927 | 0.526976798 | 4.38E-11 |
| ENSG00000178947 | ENSG00000100307 | 0.517489889 | 1.11E-10 |
| ENSG00000178947 | ENSG00000065325 | 0.521237181 | 7.72E-11 |
| ENSG00000178947 | ENSG00000053108 | 0.530369135 | 3.12E-11 |
| ENSG00000178947 | ENSG00000111344 | 0.560324279 | 1.31E-12 |
| ENSG00000178947 | ENSG00000174516 | 0.596822712 | 1.75E-14 |
| ENSG00000178947 | ENSG00000117016 | 0.578960789 | 1.55E-13 |
| ENSG00000178977 | ENSG00000120727 | 0.500404656 | 5.53E-10 |
| ENSG00000178977 | ENSG00000164088 | 0.510229671 | 2.22E-10 |
| ENSG00000179859 | ENSG00000144061 | 0.507631763 | 2.83E-10 |
| ENSG00000179859 | ENSG00000163040 | 0.519192334 | 9.42E-11 |
| ENSG00000179859 | ENSG00000179029 | 0.627004711 | 3.18E-16 |
| ENSG00000181908 | ENSG00000131899 | 0.501672842 | 4.92E-10 |
| ENSG00000182352 | ENSG00000158486 | 0.504613725 | 3.75E-10 |
| ENSG00000184608 | ENSG00000159239 | 0.539080376 | 1.28E-11 |
| ENSG00000184608 | ENSG00000146038 | 0.660363935 | 2.21E-18 |
| ENSG00000184608 | ENSG00000120051 | 0.690281513 | 1.46E-20 |
| ENSG00000184608 | ENSG00000132321 | 0.651111004 | 9.35E-18 |
| ENSG00000184608 | ENSG00000167646 | 0.530906041 | 2.96E-11 |
| ENSG00000184608 | ENSG00000166689 | 0.544484051 | 7.29E-12 |
| ENSG00000184608 | ENSG00000168491 | 0.593063215 | 2.80E-14 |
| ENSG00000184608 | ENSG00000142606 | 0.523754953 | 6.03E-11 |
| ENSG00000184608 | ENSG00000158486 | 0.523974173 | 5.90E-11 |
| ENSG00000184608 | ENSG00000205838 | 0.626195592 | 3.56E-16 |
| ENSG00000184608 | ENSG00000121270 | 0.501842881 | 4.85E-10 |
| ENSG00000184608 | ENSG00000118690 | 0.62201366 | 6.36E-16 |
| ENSG00000184608 | ENSG00000164185 | 0.639216405 | 5.54E-17 |
| ENSG00000184608 | ENSG00000111834 | 0.672765026 | 2.96E-19 |
| ENSG00000184608 | ENSG00000179813 | 0.537578217 | 1.50E-11 |
| ENSG00000184608 | ENSG00000186439 | 0.536745621 | 1.63E-11 |
| ENSG00000184608 | ENSG00000137473 | 0.663195576 | 1.41E-18 |
| ENSG00000184608 | ENSG00000166246 | 0.75676699 | 1.64E-26 |
| ENSG00000184608 | ENSG00000140057 | 0.590169785 | 4.00E-14 |
| ENSG00000184608 | ENSG00000173947 | 0.594432653 | 2.36E-14 |
| ENSG00000184608 | ENSG00000167858 | 0.657436141 | 3.51E-18 |
| ENSG00000184608 | ENSG00000156042 | 0.656875455 | 3.83E-18 |
| ENSG00000184608 | ENSG00000144031 | 0.60818055 | 4.07E-15 |
| ENSG00000184608 | ENSG00000270765 | 0.719437615 | 5.84E-23 |
| ENSG00000196972 | ENSG00000011347 | 0.646002469 | 2.03E-17 |
| ENSG00000196972 | ENSG00000172375 | 0.603933308 | 7.07E-15 |
| ENSG00000196972 | ENSG00000166501 | 0.540229887 | 1.14E-11 |
| ENSG00000196972 | ENSG00000184156 | 0.590857931 | 3.67E-14 |
| ENSG00000196972 | ENSG00000088367 | 0.5638457 | 8.84E-13 |
| ENSG00000196972 | ENSG00000177614 | 0.549981189 | 4.06E-12 |
| ENSG00000196972 | ENSG00000147676 | 0.715809686 | 1.21E-22 |
| ENSG00000196972 | ENSG00000121671 | 0.555429232 | 2.25E-12 |
| ENSG00000196972 | ENSG00000152078 | 0.541018935 | 1.05E-11 |
| ENSG00000196972 | ENSG00000157087 | 0.529383755 | 3.45E-11 |
| ENSG00000196972 | ENSG00000153933 | 0.578222007 | 1.69E-13 |
| ENSG00000196972 | ENSG00000053108 | 0.796503386 | 4.51E-31 |
| ENSG00000196972 | ENSG00000172350 | 0.724878481 | 1.93E-23 |
| ENSG00000196972 | ENSG00000137843 | 0.679657001 | 9.28E-20 |
| ENSG00000196972 | ENSG00000125814 | 0.765901288 | 1.77E-27 |
| ENSG00000196972 | ENSG00000129473 | 0.50293219 | 4.38E-10 |
| ENSG00000196972 | ENSG00000177570 | 0.677300334 | 1.39E-19 |
| ENSG00000196972 | ENSG00000180354 | 0.538762556 | 1.32E-11 |
| ENSG00000196972 | ENSG00000101298 | 0.617213324 | 1.22E-15 |
| ENSG00000196972 | ENSG00000140600 | 0.575390936 | 2.35E-13 |
| ENSG00000196972 | ENSG00000187189 | 0.517501499 | 1.11E-10 |
| ENSG00000196972 | ENSG00000060140 | 0.766790986 | 1.42E-27 |
| ENSG00000196972 | ENSG00000164506 | 0.59713461 | 1.68E-14 |
| ENSG00000196972 | ENSG00000154118 | 0.607055072 | 4.71E-15 |
| ENSG00000196972 | ENSG00000171368 | 0.613755942 | 1.95E-15 |
| ENSG00000196972 | ENSG00000117016 | 0.647348536 | 1.66E-17 |
| ENSG00000196972 | ENSG00000189241 | 0.599939228 | 1.18E-14 |
| ENSG00000196972 | ENSG00000006432 | 0.640572746 | 4.54E-17 |
| ENSG00000196972 | ENSG00000197106 | 0.775202425 | 1.64E-28 |
| ENSG00000196972 | ENSG00000006740 | 0.723054853 | 2.81E-23 |
| ENSG00000196972 | ENSG00000175264 | 0.525084678 | 5.29E-11 |
| ENSG00000196972 | ENSG00000078902 | 0.517230252 | 1.14E-10 |
| ENSG00000196972 | ENSG00000104888 | 0.707701308 | 5.84E-22 |
| ENSG00000196972 | ENSG00000023171 | 0.528396581 | 3.80E-11 |
| ENSG00000196972 | ENSG00000198626 | 0.778991137 | 6.04E-29 |
| ENSG00000196972 | ENSG00000084764 | 0.560909792 | 1.23E-12 |
| ENSG00000196972 | ENSG00000183780 | 0.595592134 | 2.04E-14 |
| ENSG00000196972 | ENSG00000115977 | 0.502862979 | 4.41E-10 |
| ENSG00000196972 | ENSG00000175352 | 0.565366102 | 7.45E-13 |
| ENSG00000196972 | ENSG00000108352 | 0.57118936 | 3.83E-13 |
| ENSG00000196972 | ENSG00000050748 | 0.563631049 | 9.06E-13 |
| ENSG00000196972 | ENSG00000165983 | 0.720833716 | 4.41E-23 |
| ENSG00000196972 | ENSG00000114757 | 0.588989783 | 4.62E-14 |
| ENSG00000196972 | ENSG00000131242 | 0.645920456 | 2.05E-17 |
| ENSG00000196972 | ENSG00000135750 | 0.635091471 | 1.01E-16 |
| ENSG00000196972 | ENSG00000187764 | 0.544440669 | 7.32E-12 |
| ENSG00000196972 | ENSG00000116254 | 0.809233274 | 9.41E-33 |
| ENSG00000196972 | ENSG00000100285 | 0.656816259 | 3.87E-18 |
| ENSG00000196972 | ENSG00000003987 | 0.506678692 | 3.10E-10 |
| ENSG00000196972 | ENSG00000158109 | 0.625042657 | 4.18E-16 |
| ENSG00000196972 | ENSG00000198794 | 0.5593519 | 1.46E-12 |
| ENSG00000203999 | ENSG00000110195 | 0.510935209 | 2.08E-10 |
| ENSG00000203999 | ENSG00000179029 | 0.550175612 | 3.98E-12 |
| ENSG00000204685 | ENSG00000116539 | 0.504524186 | 3.78E-10 |
| ENSG00000205885 | ENSG00000158711 | 0.503070893 | 4.33E-10 |
| ENSG00000205885 | ENSG00000197879 | 0.604041741 | 6.97E-15 |
| ENSG00000205885 | ENSG00000147324 | 0.507064224 | 2.99E-10 |
| ENSG00000205885 | ENSG00000178202 | 0.522485213 | 6.83E-11 |
| ENSG00000205885 | ENSG00000124782 | 0.503722336 | 4.08E-10 |
| ENSG00000206187 | ENSG00000120053 | 0.565860862 | 7.05E-13 |
| ENSG00000206417 | ENSG00000163516 | 0.690608044 | 1.37E-20 |
| ENSG00000206417 | ENSG00000109758 | 0.567260909 | 6.01E-13 |
| ENSG00000206417 | ENSG00000108963 | 0.564706986 | 8.03E-13 |
| ENSG00000206417 | ENSG00000197283 | 0.517480186 | 1.11E-10 |
| ENSG00000206417 | ENSG00000163945 | 0.58143689 | 1.15E-13 |
| ENSG00000206417 | ENSG00000156042 | 0.544075289 | 7.61E-12 |
| ENSG00000206417 | ENSG00000270379 | 0.537990507 | 1.43E-11 |
| ENSG00000206417 | ENSG00000115687 | 0.592581964 | 2.97E-14 |
| ENSG00000206417 | ENSG00000266173 | 0.514680084 | 1.46E-10 |
| ENSG00000206417 | ENSG00000197530 | 0.544879028 | 6.99E-12 |
| ENSG00000206417 | ENSG00000104731 | 0.53056552 | 3.06E-11 |
| ENSG00000206417 | ENSG00000167371 | 0.663622192 | 1.32E-18 |
| ENSG00000206417 | ENSG00000136828 | 0.534740929 | 2.00E-11 |
| ENSG00000206417 | ENSG00000169885 | 0.565814914 | 7.08E-13 |
| ENSG00000206417 | ENSG00000108352 | 0.507387364 | 2.90E-10 |
| ENSG00000215022 | ENSG00000108306 | 0.59681015 | 1.75E-14 |
| ENSG00000215022 | ENSG00000140948 | 0.569281521 | 4.77E-13 |
| ENSG00000215022 | ENSG00000111364 | 0.62159517 | 6.74E-16 |
| ENSG00000215022 | ENSG00000162408 | 0.506679842 | 3.10E-10 |
| ENSG00000215022 | ENSG00000112877 | 0.514318087 | 1.51E-10 |
| ENSG00000215022 | ENSG00000066427 | 0.574645254 | 2.57E-13 |
| ENSG00000215022 | ENSG00000196233 | 0.600854073 | 1.05E-14 |
| ENSG00000215022 | ENSG00000008083 | 0.52208041 | 7.11E-11 |
| ENSG00000215022 | ENSG00000077044 | 0.502338572 | 4.63E-10 |
| ENSG00000215022 | ENSG00000076513 | 0.529116739 | 3.54E-11 |
| ENSG00000215022 | ENSG00000099365 | 0.520774159 | 8.08E-11 |
| ENSG00000215022 | ENSG00000183337 | 0.535611158 | 1.83E-11 |
| ENSG00000215022 | ENSG00000266173 | 0.638135537 | 6.49E-17 |
| ENSG00000215022 | ENSG00000102606 | 0.546432421 | 5.93E-12 |
| ENSG00000215022 | ENSG00000075292 | 0.509698327 | 2.33E-10 |
| ENSG00000215022 | ENSG00000196391 | 0.596907117 | 1.73E-14 |
| ENSG00000215022 | ENSG00000160305 | 0.604078797 | 6.93E-15 |
| ENSG00000215022 | ENSG00000176953 | 0.575108353 | 2.43E-13 |
| ENSG00000215022 | ENSG00000170004 | 0.501691708 | 4.91E-10 |
| ENSG00000215022 | ENSG00000130338 | 0.611387287 | 2.67E-15 |
| ENSG00000215022 | ENSG00000163939 | 0.511509384 | 1.97E-10 |
| ENSG00000215022 | ENSG00000141068 | 0.548584669 | 4.71E-12 |
| ENSG00000215022 | ENSG00000172375 | 0.525501425 | 5.07E-11 |
| ENSG00000215022 | ENSG00000187801 | 0.553901223 | 2.66E-12 |
| ENSG00000215022 | ENSG00000132740 | 0.538950232 | 1.30E-11 |
| ENSG00000215022 | ENSG00000140332 | 0.529880728 | 3.28E-11 |
| ENSG00000215022 | ENSG00000198198 | 0.519490328 | 9.15E-11 |
| ENSG00000215022 | ENSG00000155744 | 0.532147919 | 2.61E-11 |
| ENSG00000215447 | ENSG00000115109 | 0.576396293 | 2.09E-13 |
| ENSG00000215447 | ENSG00000122299 | 0.516174086 | 1.26E-10 |
| ENSG00000215447 | ENSG00000157193 | 0.509660977 | 2.34E-10 |
| ENSG00000215447 | ENSG00000048028 | 0.555372833 | 2.26E-12 |
| ENSG00000215447 | ENSG00000110066 | 0.516932332 | 1.17E-10 |
| ENSG00000215447 | ENSG00000160218 | 0.810424959 | 6.45E-33 |
| ENSG00000215447 | ENSG00000160305 | 0.715301894 | 1.33E-22 |
| ENSG00000215447 | ENSG00000184677 | 0.690535472 | 1.39E-20 |
| ENSG00000215447 | ENSG00000149548 | 0.537841015 | 1.46E-11 |
| ENSG00000215447 | ENSG00000135913 | 0.702062947 | 1.70E-21 |
| ENSG00000215447 | ENSG00000133812 | 0.569401227 | 4.71E-13 |
| ENSG00000215447 | ENSG00000059145 | 0.61512403 | 1.62E-15 |
| ENSG00000215447 | ENSG00000124120 | 0.522139047 | 7.07E-11 |
| ENSG00000215447 | ENSG00000106479 | 0.558340172 | 1.63E-12 |
| ENSG00000215447 | ENSG00000006459 | 0.519683019 | 8.98E-11 |
| ENSG00000215447 | ENSG00000187189 | 0.502820569 | 4.43E-10 |
| ENSG00000215447 | ENSG00000065613 | 0.505067874 | 3.60E-10 |
| ENSG00000215447 | ENSG00000168488 | 0.588573804 | 4.86E-14 |
| ENSG00000215447 | ENSG00000122966 | 0.544486291 | 7.29E-12 |
| ENSG00000215447 | ENSG00000140992 | 0.673517749 | 2.61E-19 |
| ENSG00000215447 | ENSG00000114648 | 0.639919006 | 5.00E-17 |
| ENSG00000215447 | ENSG00000084676 | 0.514030786 | 1.55E-10 |
| ENSG00000215447 | ENSG00000135686 | 0.582597187 | 1.00E-13 |
| ENSG00000215447 | ENSG00000175216 | 0.567692853 | 5.72E-13 |
| ENSG00000215447 | ENSG00000138081 | 0.686612398 | 2.79E-20 |
| ENSG00000215447 | ENSG00000143970 | 0.55747616 | 1.80E-12 |
| ENSG00000215447 | ENSG00000169375 | 0.60941542 | 3.46E-15 |
| ENSG00000215447 | ENSG00000170871 | 0.625953149 | 3.69E-16 |
| ENSG00000215447 | ENSG00000126870 | 0.586410631 | 6.33E-14 |
| ENSG00000215447 | ENSG00000067596 | 0.599328323 | 1.27E-14 |
| ENSG00000215447 | ENSG00000134982 | 0.521501838 | 7.52E-11 |
| ENSG00000215447 | ENSG00000177169 | 0.618957413 | 9.66E-16 |
| ENSG00000215447 | ENSG00000197323 | 0.592774549 | 2.90E-14 |
| ENSG00000215447 | ENSG00000166783 | 0.696830814 | 4.46E-21 |
| ENSG00000215447 | ENSG00000166135 | 0.641461453 | 3.98E-17 |
| ENSG00000215447 | ENSG00000140320 | 0.53693993 | 1.60E-11 |
| ENSG00000215447 | ENSG00000037749 | 0.578901493 | 1.56E-13 |
| ENSG00000215447 | ENSG00000171634 | 0.688899474 | 1.86E-20 |
| ENSG00000215447 | ENSG00000149657 | 0.580360208 | 1.31E-13 |
| ENSG00000215447 | ENSG00000135392 | 0.534012659 | 2.16E-11 |
| ENSG00000215447 | ENSG00000197283 | 0.613731546 | 1.95E-15 |
| ENSG00000215447 | ENSG00000132953 | 0.50550449 | 3.46E-10 |
| ENSG00000215447 | ENSG00000184787 | 0.607934208 | 4.20E-15 |
| ENSG00000215447 | ENSG00000104517 | 0.579583246 | 1.44E-13 |
| ENSG00000215447 | ENSG00000067900 | 0.530715287 | 3.01E-11 |
| ENSG00000215447 | ENSG00000169554 | 0.503935117 | 4.00E-10 |
| ENSG00000215447 | ENSG00000008869 | 0.671261931 | 3.80E-19 |
| ENSG00000215447 | ENSG00000134698 | 0.602295347 | 8.72E-15 |
| ENSG00000215447 | ENSG00000076108 | 0.653059956 | 6.93E-18 |
| ENSG00000215447 | ENSG00000169914 | 0.591263735 | 3.50E-14 |
| ENSG00000215447 | ENSG00000025293 | 0.637923246 | 6.69E-17 |
| ENSG00000215447 | ENSG00000272886 | 0.526915838 | 4.41E-11 |
| ENSG00000215447 | ENSG00000071054 | 0.583394138 | 9.11E-14 |
| ENSG00000215447 | ENSG00000170242 | 0.509764697 | 2.32E-10 |
| ENSG00000215447 | ENSG00000065526 | 0.7486247 | 1.10E-25 |
| ENSG00000215447 | ENSG00000187605 | 0.659028436 | 2.73E-18 |
| ENSG00000215447 | ENSG00000123200 | 0.502989621 | 4.36E-10 |
| ENSG00000215447 | ENSG00000067248 | 0.518229769 | 1.03E-10 |
| ENSG00000215447 | ENSG00000170456 | 0.594022101 | 2.48E-14 |
| ENSG00000215447 | ENSG00000105778 | 0.567105604 | 6.12E-13 |
| ENSG00000215447 | ENSG00000165097 | 0.503684389 | 4.09E-10 |
| ENSG00000215447 | ENSG00000109756 | 0.547639768 | 5.22E-12 |
| ENSG00000215447 | ENSG00000204843 | 0.517844884 | 1.07E-10 |
| ENSG00000215447 | ENSG00000100815 | 0.518424376 | 1.01E-10 |
| ENSG00000215447 | ENSG00000134452 | 0.555863833 | 2.14E-12 |
| ENSG00000215447 | ENSG00000025800 | 0.571888926 | 3.54E-13 |
| ENSG00000215447 | ENSG00000170921 | 0.590495238 | 3.84E-14 |
| ENSG00000215447 | ENSG00000144535 | 0.52632246 | 4.68E-11 |
| ENSG00000215447 | ENSG00000196323 | 0.600148003 | 1.15E-14 |
| ENSG00000215447 | ENSG00000124486 | 0.523297133 | 6.31E-11 |
| ENSG00000215447 | ENSG00000185722 | 0.562893152 | 9.84E-13 |
| ENSG00000215447 | ENSG00000151461 | 0.562924055 | 9.81E-13 |
| ENSG00000215447 | ENSG00000180357 | 0.650335702 | 1.05E-17 |
| ENSG00000215447 | ENSG00000196914 | 0.591039609 | 3.59E-14 |
| ENSG00000215447 | ENSG00000151422 | 0.623687208 | 5.05E-16 |
| ENSG00000215447 | ENSG00000264522 | 0.528379536 | 3.81E-11 |
| ENSG00000215447 | ENSG00000197226 | 0.538002021 | 1.43E-11 |
| ENSG00000215447 | ENSG00000163625 | 0.572971906 | 3.12E-13 |
| ENSG00000215447 | ENSG00000153317 | 0.576178749 | 2.15E-13 |
| ENSG00000215447 | ENSG00000053254 | 0.664127598 | 1.21E-18 |
| ENSG00000215447 | ENSG00000157540 | 0.530560022 | 3.06E-11 |
| ENSG00000215447 | ENSG00000011021 | 0.58946283 | 4.36E-14 |
| ENSG00000215447 | ENSG00000187555 | 0.641020096 | 4.25E-17 |
| ENSG00000215447 | ENSG00000149187 | 0.650682461 | 9.98E-18 |
| ENSG00000215447 | ENSG00000069275 | 0.542466428 | 9.01E-12 |
| ENSG00000215447 | ENSG00000102921 | 0.57784762 | 1.76E-13 |
| ENSG00000215447 | ENSG00000116198 | 0.627413128 | 3.00E-16 |
| ENSG00000215447 | ENSG00000114867 | 0.584528765 | 7.95E-14 |
| ENSG00000215447 | ENSG00000125686 | 0.700174104 | 2.41E-21 |
| ENSG00000215447 | ENSG00000266173 | 0.528350635 | 3.82E-11 |
| ENSG00000215447 | ENSG00000139436 | 0.646090616 | 2.00E-17 |
| ENSG00000215447 | ENSG00000126581 | 0.50574952 | 3.38E-10 |
| ENSG00000215447 | ENSG00000176953 | 0.584965681 | 7.54E-14 |
| ENSG00000215447 | ENSG00000107560 | 0.504852469 | 3.67E-10 |
| ENSG00000215447 | ENSG00000140948 | 0.678394038 | 1.15E-19 |
| ENSG00000215447 | ENSG00000140382 | 0.509356743 | 2.41E-10 |
| ENSG00000215447 | ENSG00000153201 | 0.553170814 | 2.88E-12 |
| ENSG00000215447 | ENSG00000019995 | 0.711337492 | 2.90E-22 |
| ENSG00000215447 | ENSG00000149930 | 0.61660757 | 1.33E-15 |
| ENSG00000215447 | ENSG00000125447 | 0.572845315 | 3.17E-13 |
| ENSG00000215447 | ENSG00000115020 | 0.60987556 | 3.26E-15 |
| ENSG00000215447 | ENSG00000164190 | 0.668520526 | 5.96E-19 |
| ENSG00000215447 | ENSG00000167110 | 0.613957413 | 1.90E-15 |
| ENSG00000215447 | ENSG00000066135 | 0.603000883 | 7.97E-15 |
| ENSG00000215447 | ENSG00000078687 | 0.639712504 | 5.15E-17 |
| ENSG00000215447 | ENSG00000183495 | 0.698389468 | 3.35E-21 |
| ENSG00000215447 | ENSG00000008083 | 0.590819307 | 3.69E-14 |
| ENSG00000215447 | ENSG00000066739 | 0.57746703 | 1.84E-13 |
| ENSG00000215447 | ENSG00000066933 | 0.543389144 | 8.18E-12 |
| ENSG00000215447 | ENSG00000197217 | 0.504137821 | 3.92E-10 |
| ENSG00000215447 | ENSG00000146587 | 0.611802737 | 2.52E-15 |
| ENSG00000215447 | ENSG00000081026 | 0.560682399 | 1.26E-12 |
| ENSG00000215447 | ENSG00000175662 | 0.566541666 | 6.52E-13 |
| ENSG00000215447 | ENSG00000179981 | 0.504517963 | 3.79E-10 |
| ENSG00000215447 | ENSG00000108819 | 0.501687071 | 4.92E-10 |
| ENSG00000215447 | ENSG00000118900 | 0.722780386 | 2.97E-23 |
| ENSG00000215447 | ENSG00000108510 | 0.590565883 | 3.81E-14 |
| ENSG00000215447 | ENSG00000064999 | 0.55091728 | 3.67E-12 |
| ENSG00000215447 | ENSG00000180667 | 0.57350781 | 2.93E-13 |
| ENSG00000215447 | ENSG00000110497 | 0.562776685 | 9.97E-13 |
| ENSG00000215447 | ENSG00000079308 | 0.553990331 | 2.63E-12 |
| ENSG00000215447 | ENSG00000037637 | 0.585058483 | 7.46E-14 |
| ENSG00000215447 | ENSG00000186660 | 0.522954803 | 6.52E-11 |
| ENSG00000215447 | ENSG00000110237 | 0.572547354 | 3.28E-13 |
| ENSG00000215447 | ENSG00000103404 | 0.630769428 | 1.87E-16 |
| ENSG00000215447 | ENSG00000166439 | 0.710640896 | 3.32E-22 |
| ENSG00000215447 | ENSG00000115977 | 0.500050398 | 5.71E-10 |
| ENSG00000215447 | ENSG00000110851 | 0.607165556 | 4.65E-15 |
| ENSG00000215447 | ENSG00000005810 | 0.529454497 | 3.42E-11 |
| ENSG00000215447 | ENSG00000137776 | 0.548089814 | 4.97E-12 |
| ENSG00000215447 | ENSG00000152990 | 0.514416437 | 1.49E-10 |
| ENSG00000215447 | ENSG00000010322 | 0.567009368 | 6.19E-13 |
| ENSG00000215447 | ENSG00000126705 | 0.55051479 | 3.83E-12 |
| ENSG00000215447 | ENSG00000086758 | 0.748973306 | 1.02E-25 |
| ENSG00000215447 | ENSG00000139613 | 0.543664619 | 7.94E-12 |
| ENSG00000215447 | ENSG00000205726 | 0.56403893 | 8.65E-13 |
| ENSG00000215447 | ENSG00000151276 | 0.556023041 | 2.11E-12 |
| ENSG00000215447 | ENSG00000011275 | 0.534223544 | 2.11E-11 |
| ENSG00000215447 | ENSG00000275023 | 0.653104804 | 6.88E-18 |
| ENSG00000215447 | ENSG00000196547 | 0.508034455 | 2.73E-10 |
| ENSG00000215447 | ENSG00000164164 | 0.544344162 | 7.40E-12 |
| ENSG00000215447 | ENSG00000083168 | 0.708964294 | 4.59E-22 |
| ENSG00000215447 | ENSG00000123066 | 0.692726022 | 9.40E-21 |
| ENSG00000215447 | ENSG00000106144 | 0.506806861 | 3.06E-10 |
| ENSG00000215447 | ENSG00000152102 | 0.706958973 | 6.73E-22 |
| ENSG00000215447 | ENSG00000100354 | 0.553367212 | 2.82E-12 |
| ENSG00000215447 | ENSG00000074755 | 0.757085282 | 1.52E-26 |
| ENSG00000215447 | ENSG00000163939 | 0.667458783 | 7.09E-19 |
| ENSG00000215447 | ENSG00000204569 | 0.573023052 | 3.10E-13 |
| ENSG00000215447 | ENSG00000243232 | 0.508061884 | 2.72E-10 |
| ENSG00000215447 | ENSG00000138031 | 0.525753971 | 4.95E-11 |
| ENSG00000215447 | ENSG00000168763 | 0.515689968 | 1.32E-10 |
| ENSG00000215447 | ENSG00000139437 | 0.563907441 | 8.78E-13 |
| ENSG00000215447 | ENSG00000213380 | 0.549300369 | 4.37E-12 |
| ENSG00000215447 | ENSG00000137337 | 0.654686111 | 5.39E-18 |
| ENSG00000215447 | ENSG00000170832 | 0.659748309 | 2.44E-18 |
| ENSG00000215447 | ENSG00000055917 | 0.568747783 | 5.07E-13 |
| ENSG00000215447 | ENSG00000198836 | 0.548390748 | 4.81E-12 |
| ENSG00000215447 | ENSG00000084112 | 0.549850044 | 4.12E-12 |
| ENSG00000215447 | ENSG00000175727 | 0.523190638 | 6.37E-11 |
| ENSG00000215447 | ENSG00000166833 | 0.502683119 | 4.49E-10 |
| ENSG00000215447 | ENSG00000141027 | 0.501539881 | 4.98E-10 |
| ENSG00000215447 | ENSG00000100813 | 0.57523534 | 2.40E-13 |
| ENSG00000215447 | ENSG00000102858 | 0.555902609 | 2.14E-12 |
| ENSG00000215447 | ENSG00000257093 | 0.531979512 | 2.65E-11 |
| ENSG00000215447 | ENSG00000090686 | 0.590313131 | 3.93E-14 |
| ENSG00000215447 | ENSG00000154114 | 0.540950651 | 1.06E-11 |
| ENSG00000215447 | ENSG00000166197 | 0.728612452 | 8.88E-24 |
| ENSG00000215447 | ENSG00000070047 | 0.571627467 | 3.64E-13 |
| ENSG00000215447 | ENSG00000182473 | 0.632694295 | 1.42E-16 |
| ENSG00000215447 | ENSG00000118007 | 0.621302116 | 7.01E-16 |
| ENSG00000215447 | ENSG00000113194 | 0.601803699 | 9.29E-15 |
| ENSG00000215447 | ENSG00000182095 | 0.615041305 | 1.64E-15 |
| ENSG00000215447 | ENSG00000151693 | 0.637189308 | 7.45E-17 |
| ENSG00000215447 | ENSG00000132604 | 0.50030901 | 5.58E-10 |
| ENSG00000215447 | ENSG00000033327 | 0.538265672 | 1.39E-11 |
| ENSG00000215447 | ENSG00000130227 | 0.592267324 | 3.09E-14 |
| ENSG00000215447 | ENSG00000157077 | 0.584262283 | 8.21E-14 |
| ENSG00000215447 | ENSG00000163125 | 0.656161094 | 4.29E-18 |
| ENSG00000215447 | ENSG00000197312 | 0.691853096 | 1.10E-20 |
| ENSG00000215447 | ENSG00000166326 | 0.504603489 | 3.76E-10 |
| ENSG00000215447 | ENSG00000130939 | 0.526000141 | 4.83E-11 |
| ENSG00000215447 | ENSG00000072364 | 0.715031805 | 1.41E-22 |
| ENSG00000215447 | ENSG00000147324 | 0.536269822 | 1.71E-11 |
| ENSG00000215447 | ENSG00000160710 | 0.571371597 | 3.75E-13 |
| ENSG00000215447 | ENSG00000119638 | 0.556610439 | 1.98E-12 |
| ENSG00000215447 | ENSG00000116199 | 0.556651837 | 1.97E-12 |
| ENSG00000215447 | ENSG00000141252 | 0.6502253 | 1.07E-17 |
| ENSG00000215447 | ENSG00000187079 | 0.618777515 | 9.90E-16 |
| ENSG00000215447 | ENSG00000188786 | 0.678970213 | 1.04E-19 |
| ENSG00000215908 | ENSG00000125447 | 0.586526914 | 6.24E-14 |
| ENSG00000215908 | ENSG00000142751 | 0.551936201 | 3.29E-12 |
| ENSG00000215908 | ENSG00000166436 | 0.549944352 | 4.08E-12 |
| ENSG00000215908 | ENSG00000177728 | 0.500242266 | 5.61E-10 |
| ENSG00000215908 | ENSG00000078687 | 0.526747999 | 4.48E-11 |
| ENSG00000215908 | ENSG00000163945 | 0.53915621 | 1.27E-11 |
| ENSG00000215908 | ENSG00000162408 | 0.517277506 | 1.13E-10 |
| ENSG00000215908 | ENSG00000106479 | 0.522202038 | 7.02E-11 |
| ENSG00000215908 | ENSG00000176953 | 0.561259815 | 1.18E-12 |
| ENSG00000215908 | ENSG00000144589 | 0.56861374 | 5.15E-13 |
| ENSG00000215908 | ENSG00000169914 | 0.568951039 | 4.96E-13 |
| ENSG00000215908 | ENSG00000133226 | 0.569330201 | 4.75E-13 |
| ENSG00000215908 | ENSG00000148356 | 0.576276724 | 2.12E-13 |
| ENSG00000215908 | ENSG00000059145 | 0.554941687 | 2.37E-12 |
| ENSG00000215908 | ENSG00000074964 | 0.50743655 | 2.89E-10 |
| ENSG00000215908 | ENSG00000146826 | 0.52782064 | 4.03E-11 |
| ENSG00000215908 | ENSG00000266173 | 0.607757913 | 4.30E-15 |
| ENSG00000215908 | ENSG00000160305 | 0.571845086 | 3.55E-13 |
| ENSG00000215908 | ENSG00000169062 | 0.553948682 | 2.64E-12 |
| ENSG00000215908 | ENSG00000187605 | 0.511276549 | 2.01E-10 |
| ENSG00000215908 | ENSG00000134698 | 0.575122345 | 2.43E-13 |
| ENSG00000215908 | ENSG00000197323 | 0.502338262 | 4.63E-10 |
| ENSG00000215908 | ENSG00000137337 | 0.547287224 | 5.41E-12 |
| ENSG00000215908 | ENSG00000070610 | 0.535067747 | 1.94E-11 |
| ENSG00000215908 | ENSG00000141068 | 0.509828807 | 2.31E-10 |
| ENSG00000215908 | ENSG00000213983 | 0.580491053 | 1.29E-13 |
| ENSG00000215908 | ENSG00000132740 | 0.562403907 | 1.04E-12 |
| ENSG00000215908 | ENSG00000108352 | 0.53707975 | 1.58E-11 |
| ENSG00000215908 | ENSG00000111364 | 0.577586551 | 1.82E-13 |
| ENSG00000215908 | ENSG00000065526 | 0.530738993 | 3.01E-11 |
| ENSG00000215908 | ENSG00000143093 | 0.535370143 | 1.88E-11 |
| ENSG00000215908 | ENSG00000108963 | 0.555582319 | 2.21E-12 |
| ENSG00000215908 | ENSG00000011021 | 0.562078981 | 1.08E-12 |
| ENSG00000215908 | ENSG00000275023 | 0.543562396 | 8.03E-12 |
| ENSG00000215908 | ENSG00000139437 | 0.533729622 | 2.22E-11 |
| ENSG00000216863 | ENSG00000136535 | 0.819120257 | 3.79E-34 |
| ENSG00000216863 | ENSG00000164506 | 0.542808765 | 8.69E-12 |
| ENSG00000216863 | ENSG00000100285 | 0.647807788 | 1.54E-17 |
| ENSG00000216863 | ENSG00000053108 | 0.687220129 | 2.50E-20 |
| ENSG00000216863 | ENSG00000109466 | 0.540077471 | 1.16E-11 |
| ENSG00000216863 | ENSG00000128656 | 0.648022271 | 1.50E-17 |
| ENSG00000216863 | ENSG00000156486 | 0.868629576 | 1.07E-42 |
| ENSG00000216863 | ENSG00000106089 | 0.611741234 | 2.55E-15 |
| ENSG00000216863 | ENSG00000107130 | 0.539149279 | 1.27E-11 |
| ENSG00000216863 | ENSG00000060140 | 0.856783959 | 2.30E-40 |
| ENSG00000216863 | ENSG00000171517 | 0.660218506 | 2.27E-18 |
| ENSG00000216863 | ENSG00000149927 | 0.673298839 | 2.71E-19 |
| ENSG00000216863 | ENSG00000135750 | 0.692058462 | 1.06E-20 |
| ENSG00000216863 | ENSG00000153933 | 0.637210986 | 7.42E-17 |
| ENSG00000216863 | ENSG00000119946 | 0.655850053 | 4.50E-18 |
| ENSG00000216863 | ENSG00000139112 | 0.520049133 | 8.67E-11 |
| ENSG00000216863 | ENSG00000154118 | 0.533531828 | 2.27E-11 |
| ENSG00000216863 | ENSG00000171126 | 0.767681233 | 1.13E-27 |
| ENSG00000216863 | ENSG00000176194 | 0.812615556 | 3.20E-33 |
| ENSG00000216863 | ENSG00000197106 | 0.70200075 | 1.72E-21 |
| ENSG00000216863 | ENSG00000163630 | 0.768144199 | 1.01E-27 |
| ENSG00000216863 | ENSG00000107758 | 0.682593647 | 5.61E-20 |
| ENSG00000216863 | ENSG00000091622 | 0.618273386 | 1.06E-15 |
| ENSG00000216863 | ENSG00000183780 | 0.641851324 | 3.76E-17 |
| ENSG00000216863 | ENSG00000147676 | 0.800159075 | 1.53E-31 |
| ENSG00000216863 | ENSG00000165983 | 0.76454747 | 2.48E-27 |
| ENSG00000216863 | ENSG00000065559 | 0.508052086 | 2.73E-10 |
| ENSG00000216863 | ENSG00000215218 | 0.505161453 | 3.57E-10 |
| ENSG00000216863 | ENSG00000115365 | 0.500071821 | 5.70E-10 |
| ENSG00000216863 | ENSG00000180354 | 0.523010332 | 6.49E-11 |
| ENSG00000216863 | ENSG00000050748 | 0.516275637 | 1.25E-10 |
| ENSG00000216863 | ENSG00000156642 | 0.505883696 | 3.34E-10 |
| ENSG00000216863 | ENSG00000133816 | 0.524330756 | 5.70E-11 |
| ENSG00000216863 | ENSG00000129990 | 0.664295068 | 1.18E-18 |
| ENSG00000216863 | ENSG00000136854 | 0.572495872 | 3.30E-13 |
| ENSG00000216863 | ENSG00000137766 | 0.845263675 | 2.74E-38 |
| ENSG00000216863 | ENSG00000135423 | 0.791836123 | 1.74E-30 |
| ENSG00000216863 | ENSG00000065325 | 0.846050805 | 2.00E-38 |
| ENSG00000216863 | ENSG00000135638 | 0.757746444 | 1.30E-26 |
| ENSG00000216863 | ENSG00000138078 | 0.503452356 | 4.18E-10 |
| ENSG00000216863 | ENSG00000189241 | 0.518418243 | 1.02E-10 |
| ENSG00000216863 | ENSG00000131738 | 0.624416414 | 4.56E-16 |
| ENSG00000216863 | ENSG00000084764 | 0.506384539 | 3.18E-10 |
| ENSG00000216863 | ENSG00000183049 | 0.515198905 | 1.38E-10 |
| ENSG00000216863 | ENSG00000156414 | 0.644384999 | 2.58E-17 |
| ENSG00000216863 | ENSG00000140600 | 0.653717588 | 6.26E-18 |
| ENSG00000224046 | ENSG00000184154 | 0.60819041 | 4.06E-15 |
| ENSG00000224046 | ENSG00000085662 | 0.530081677 | 3.21E-11 |
| ENSG00000224743 | ENSG00000155761 | 0.514449926 | 1.49E-10 |
| ENSG00000224743 | ENSG00000146038 | 0.528487692 | 3.77E-11 |
| ENSG00000224743 | ENSG00000141198 | 0.508326317 | 2.66E-10 |
| ENSG00000225675 | ENSG00000138944 | 0.515624043 | 1.33E-10 |
| ENSG00000226711 | ENSG00000135299 | 0.5137423 | 1.59E-10 |
| ENSG00000226711 | ENSG00000139197 | 0.534138911 | 2.13E-11 |
| ENSG00000226711 | ENSG00000167971 | 0.502393961 | 4.61E-10 |
| ENSG00000226711 | ENSG00000088179 | 0.538005762 | 1.43E-11 |
| ENSG00000226711 | ENSG00000148660 | 0.520408014 | 8.37E-11 |
| ENSG00000226711 | ENSG00000173825 | 0.532978049 | 2.40E-11 |
| ENSG00000226816 | ENSG00000160255 | 0.511719944 | 1.93E-10 |
| ENSG00000226816 | ENSG00000115956 | 0.559134516 | 1.50E-12 |
| ENSG00000226816 | ENSG00000095585 | 0.515318358 | 1.37E-10 |
| ENSG00000226816 | ENSG00000113269 | 0.523487543 | 6.19E-11 |
| ENSG00000226816 | ENSG00000167642 | 0.57574663 | 2.26E-13 |
| ENSG00000226816 | ENSG00000100055 | 0.545236192 | 6.73E-12 |
| ENSG00000226816 | ENSG00000140968 | 0.664140666 | 1.21E-18 |
| ENSG00000226816 | ENSG00000147459 | 0.529622788 | 3.36E-11 |
| ENSG00000226816 | ENSG00000129226 | 0.561476015 | 1.15E-12 |
| ENSG00000226816 | ENSG00000167208 | 0.514463215 | 1.49E-10 |
| ENSG00000226816 | ENSG00000169252 | 0.645438656 | 2.21E-17 |
| ENSG00000226816 | ENSG00000092200 | 0.635077905 | 1.01E-16 |
| ENSG00000226816 | ENSG00000185811 | 0.557220181 | 1.85E-12 |
| ENSG00000226816 | ENSG00000110324 | 0.534005544 | 2.16E-11 |
| ENSG00000226816 | ENSG00000142512 | 0.53592937 | 1.77E-11 |
| ENSG00000226816 | ENSG00000137841 | 0.579272559 | 1.49E-13 |
| ENSG00000226816 | ENSG00000108639 | 0.542503063 | 8.97E-12 |
| ENSG00000226816 | ENSG00000111269 | 0.504471483 | 3.80E-10 |
| ENSG00000227372 | ENSG00000070047 | 0.516467466 | 1.23E-10 |
| ENSG00000227372 | ENSG00000187079 | 0.501432191 | 5.03E-10 |
| ENSG00000227372 | ENSG00000100441 | 0.577815626 | 1.77E-13 |
| ENSG00000227372 | ENSG00000146587 | 0.528709438 | 3.69E-11 |
| ENSG00000227372 | ENSG00000198585 | 0.568350828 | 5.31E-13 |
| ENSG00000227630 | ENSG00000113522 | 0.572639175 | 3.24E-13 |
| ENSG00000227630 | ENSG00000113194 | 0.550629276 | 3.79E-12 |
| ENSG00000227695 | ENSG00000111275 | 0.51510998 | 1.40E-10 |
| ENSG00000228408 | ENSG00000021300 | 0.503763769 | 4.06E-10 |
| ENSG00000228408 | ENSG00000111275 | 0.529169243 | 3.52E-11 |
| ENSG00000228408 | ENSG00000114757 | 0.522628639 | 6.74E-11 |
| ENSG00000228408 | ENSG00000180354 | 0.537136132 | 1.57E-11 |
| ENSG00000228434 | ENSG00000196689 | 0.501806244 | 4.86E-10 |
| ENSG00000228544 | ENSG00000160218 | 0.541333214 | 1.01E-11 |
| ENSG00000228544 | ENSG00000178188 | 0.501817043 | 4.86E-10 |
| ENSG00000228544 | ENSG00000072501 | 0.510623948 | 2.14E-10 |
| ENSG00000228544 | ENSG00000113194 | 0.559430545 | 1.45E-12 |
| ENSG00000228544 | ENSG00000152102 | 0.50053662 | 5.46E-10 |
| ENSG00000228544 | ENSG00000086758 | 0.591537197 | 3.38E-14 |
| ENSG00000228544 | ENSG00000065526 | 0.522087622 | 7.10E-11 |
| ENSG00000228544 | ENSG00000166135 | 0.500123188 | 5.67E-10 |
| ENSG00000228544 | ENSG00000149930 | 0.544180989 | 7.52E-12 |
| ENSG00000228544 | ENSG00000274211 | 0.523058479 | 6.46E-11 |
| ENSG00000228544 | ENSG00000115568 | 0.553920025 | 2.65E-12 |
| ENSG00000228544 | ENSG00000166783 | 0.557883674 | 1.72E-12 |
| ENSG00000228544 | ENSG00000160299 | 0.610410627 | 3.04E-15 |
| ENSG00000228544 | ENSG00000166833 | 0.53663531 | 1.65E-11 |
| ENSG00000228544 | ENSG00000131149 | 0.549355126 | 4.34E-12 |
| ENSG00000228544 | ENSG00000166197 | 0.531829729 | 2.69E-11 |
| ENSG00000228544 | ENSG00000174231 | 0.539327062 | 1.25E-11 |
| ENSG00000228544 | ENSG00000136828 | 0.519747752 | 8.93E-11 |
| ENSG00000228544 | ENSG00000214655 | 0.551994602 | 3.27E-12 |
| ENSG00000228544 | ENSG00000133226 | 0.515004739 | 1.41E-10 |
| ENSG00000228544 | ENSG00000149187 | 0.5118389 | 1.91E-10 |
| ENSG00000228630 | ENSG00000120093 | 0.522084558 | 7.11E-11 |
| ENSG00000228630 | ENSG00000144891 | 0.627750115 | 2.87E-16 |
| ENSG00000228630 | ENSG00000180818 | 0.735140394 | 2.22E-24 |
| ENSG00000228971 | ENSG00000102003 | 0.586968382 | 5.92E-14 |
| ENSG00000228971 | ENSG00000183780 | 0.63865822 | 6.01E-17 |
| ENSG00000228971 | ENSG00000104722 | 0.643727233 | 2.85E-17 |
| ENSG00000228971 | ENSG00000198513 | 0.604613638 | 6.47E-15 |
| ENSG00000228971 | ENSG00000139112 | 0.529199496 | 3.51E-11 |
| ENSG00000228971 | ENSG00000137843 | 0.573136959 | 3.06E-13 |
| ENSG00000228971 | ENSG00000140600 | 0.637545244 | 7.07E-17 |
| ENSG00000228971 | ENSG00000167642 | 0.53489995 | 1.97E-11 |
| ENSG00000228971 | ENSG00000091622 | 0.56432639 | 8.38E-13 |
| ENSG00000228971 | ENSG00000172350 | 0.582348461 | 1.03E-13 |
| ENSG00000228971 | ENSG00000125814 | 0.715147212 | 1.37E-22 |
| ENSG00000228971 | ENSG00000158856 | 0.561910438 | 1.10E-12 |
| ENSG00000228971 | ENSG00000181418 | 0.729001523 | 8.19E-24 |
| ENSG00000228971 | ENSG00000171126 | 0.718750237 | 6.71E-23 |
| ENSG00000228971 | ENSG00000100307 | 0.525268576 | 5.19E-11 |
| ENSG00000228971 | ENSG00000116254 | 0.710716946 | 3.27E-22 |
| ENSG00000228971 | ENSG00000215218 | 0.537618962 | 1.49E-11 |
| ENSG00000228971 | ENSG00000100285 | 0.599959897 | 1.18E-14 |
| ENSG00000228971 | ENSG00000136854 | 0.566047581 | 6.90E-13 |
| ENSG00000228971 | ENSG00000060140 | 0.849161446 | 5.69E-39 |
| ENSG00000228971 | ENSG00000163995 | 0.563926988 | 8.76E-13 |
| ENSG00000228971 | ENSG00000189241 | 0.500045981 | 5.71E-10 |
| ENSG00000228971 | ENSG00000147676 | 0.773517295 | 2.55E-28 |
| ENSG00000228971 | ENSG00000177570 | 0.57736704 | 1.87E-13 |
| ENSG00000228971 | ENSG00000114757 | 0.576758997 | 2.00E-13 |
| ENSG00000228971 | ENSG00000154118 | 0.557210561 | 1.85E-12 |
| ENSG00000228971 | ENSG00000078328 | 0.732062081 | 4.29E-24 |
| ENSG00000228971 | ENSG00000127585 | 0.555584284 | 2.21E-12 |
| ENSG00000228971 | ENSG00000123612 | 0.54920184 | 4.41E-12 |
| ENSG00000228971 | ENSG00000050748 | 0.502920768 | 4.39E-10 |
| ENSG00000228971 | ENSG00000132872 | 0.509342549 | 2.41E-10 |
| ENSG00000228971 | ENSG00000123901 | 0.832329337 | 3.76E-36 |
| ENSG00000228971 | ENSG00000147416 | 0.510984096 | 2.07E-10 |
| ENSG00000228971 | ENSG00000129990 | 0.676341367 | 1.63E-19 |
| ENSG00000228971 | ENSG00000084764 | 0.510175704 | 2.23E-10 |
| ENSG00000228971 | ENSG00000072657 | 0.807488247 | 1.63E-32 |
| ENSG00000228971 | ENSG00000176194 | 0.776058938 | 1.31E-28 |
| ENSG00000228971 | ENSG00000053108 | 0.677028196 | 1.45E-19 |
| ENSG00000228971 | ENSG00000116675 | 0.528973051 | 3.59E-11 |
| ENSG00000228971 | ENSG00000166501 | 0.567593125 | 5.79E-13 |
| ENSG00000228971 | ENSG00000198626 | 0.793974944 | 9.42E-31 |
| ENSG00000228971 | ENSG00000135750 | 0.66995077 | 4.72E-19 |
| ENSG00000228971 | ENSG00000165983 | 0.795676108 | 5.75E-31 |
| ENSG00000228971 | ENSG00000142686 | 0.51234213 | 1.82E-10 |
| ENSG00000228971 | ENSG00000007001 | 0.581468612 | 1.15E-13 |
| ENSG00000228971 | ENSG00000183785 | 0.684404418 | 4.10E-20 |
| ENSG00000228971 | ENSG00000107758 | 0.679640489 | 9.31E-20 |
| ENSG00000228971 | ENSG00000135638 | 0.772577019 | 3.25E-28 |
| ENSG00000228971 | ENSG00000101438 | 0.563569534 | 9.12E-13 |
| ENSG00000228971 | ENSG00000158109 | 0.580675594 | 1.26E-13 |
| ENSG00000228971 | ENSG00000153933 | 0.618255213 | 1.06E-15 |
| ENSG00000228971 | ENSG00000011347 | 0.546624004 | 5.81E-12 |
| ENSG00000228971 | ENSG00000197106 | 0.670884909 | 4.04E-19 |
| ENSG00000228971 | ENSG00000004660 | 0.642902597 | 3.22E-17 |
| ENSG00000228971 | ENSG00000149742 | 0.62606242 | 3.63E-16 |
| ENSG00000228971 | ENSG00000110148 | 0.668063046 | 6.42E-19 |
| ENSG00000228971 | ENSG00000133816 | 0.548574976 | 4.72E-12 |
| ENSG00000228971 | ENSG00000065559 | 0.508037854 | 2.73E-10 |
| ENSG00000228971 | ENSG00000163630 | 0.80657722 | 2.16E-32 |
| ENSG00000228971 | ENSG00000183336 | 0.515134656 | 1.39E-10 |
| ENSG00000228971 | ENSG00000175352 | 0.591527408 | 3.38E-14 |
| ENSG00000228971 | ENSG00000164506 | 0.519924495 | 8.77E-11 |
| ENSG00000229124 | ENSG00000086062 | 0.507213389 | 2.95E-10 |
| ENSG00000229124 | ENSG00000145779 | 0.557468907 | 1.80E-12 |
| ENSG00000229152 | ENSG00000196498 | 0.531261173 | 2.85E-11 |
| ENSG00000229152 | ENSG00000123200 | 0.55985284 | 1.38E-12 |
| ENSG00000229152 | ENSG00000162664 | 0.620023265 | 8.35E-16 |
| ENSG00000229152 | ENSG00000152223 | 0.57737677 | 1.86E-13 |
| ENSG00000229152 | ENSG00000078687 | 0.53911773 | 1.28E-11 |
| ENSG00000229152 | ENSG00000106086 | 0.521919076 | 7.22E-11 |
| ENSG00000229152 | ENSG00000104517 | 0.565344868 | 7.47E-13 |
| ENSG00000229152 | ENSG00000088448 | 0.787866011 | 5.35E-30 |
| ENSG00000229152 | ENSG00000066933 | 0.551355393 | 3.50E-12 |
| ENSG00000229152 | ENSG00000065526 | 0.609099077 | 3.61E-15 |
| ENSG00000229152 | ENSG00000038382 | 0.606764531 | 4.90E-15 |
| ENSG00000229152 | ENSG00000171634 | 0.609230894 | 3.55E-15 |
| ENSG00000229152 | ENSG00000166450 | 0.56660399 | 6.48E-13 |
| ENSG00000229152 | ENSG00000152520 | 0.72004825 | 5.17E-23 |
| ENSG00000229152 | ENSG00000197323 | 0.566925423 | 6.24E-13 |
| ENSG00000229152 | ENSG00000011021 | 0.576771118 | 2.00E-13 |
| ENSG00000229152 | ENSG00000213983 | 0.534916157 | 1.97E-11 |
| ENSG00000229152 | ENSG00000132953 | 0.648436984 | 1.40E-17 |
| ENSG00000229152 | ENSG00000068650 | 0.576536877 | 2.06E-13 |
| ENSG00000229152 | ENSG00000139668 | 0.623750122 | 5.00E-16 |
| ENSG00000229152 | ENSG00000182095 | 0.518691394 | 9.89E-11 |
| ENSG00000229152 | ENSG00000133030 | 0.51384227 | 1.58E-10 |
| ENSG00000229152 | ENSG00000176953 | 0.680165925 | 8.51E-20 |
| ENSG00000229152 | ENSG00000166716 | 0.51463966 | 1.46E-10 |
| ENSG00000229152 | ENSG00000108510 | 0.554238851 | 2.56E-12 |
| ENSG00000229152 | ENSG00000110344 | 0.528072095 | 3.93E-11 |
| ENSG00000229152 | ENSG00000169914 | 0.681414855 | 6.87E-20 |
| ENSG00000229152 | ENSG00000066739 | 0.613185437 | 2.10E-15 |
| ENSG00000229152 | ENSG00000008083 | 0.50260707 | 4.52E-10 |
| ENSG00000229152 | ENSG00000106479 | 0.532009465 | 2.64E-11 |
| ENSG00000229152 | ENSG00000084112 | 0.542735461 | 8.76E-12 |
| ENSG00000229152 | ENSG00000115020 | 0.575364543 | 2.36E-13 |
| ENSG00000229152 | ENSG00000275023 | 0.557220544 | 1.85E-12 |
| ENSG00000229152 | ENSG00000108963 | 0.546560998 | 5.85E-12 |
| ENSG00000229152 | ENSG00000164190 | 0.565676791 | 7.19E-13 |
| ENSG00000229152 | ENSG00000166436 | 0.509462896 | 2.39E-10 |
| ENSG00000229152 | ENSG00000099331 | 0.57445306 | 2.63E-13 |
| ENSG00000229152 | ENSG00000127511 | 0.594957 | 2.21E-14 |
| ENSG00000229152 | ENSG00000137776 | 0.564726621 | 8.01E-13 |
| ENSG00000229152 | ENSG00000166783 | 0.519357637 | 9.27E-11 |
| ENSG00000229152 | ENSG00000077044 | 0.561717228 | 1.12E-12 |
| ENSG00000229152 | ENSG00000156030 | 0.630268872 | 2.01E-16 |
| ENSG00000229152 | ENSG00000048028 | 0.518949051 | 9.65E-11 |
| ENSG00000229152 | ENSG00000196914 | 0.501817786 | 4.86E-10 |
| ENSG00000229152 | ENSG00000102606 | 0.619693824 | 8.74E-16 |
| ENSG00000229152 | ENSG00000164164 | 0.583142191 | 9.39E-14 |
| ENSG00000229152 | ENSG00000143776 | 0.504409789 | 3.83E-10 |
| ENSG00000229152 | ENSG00000234616 | 0.575820178 | 2.24E-13 |
| ENSG00000229152 | ENSG00000187605 | 0.637562247 | 7.05E-17 |
| ENSG00000229152 | ENSG00000167522 | 0.51965456 | 9.01E-11 |
| ENSG00000229152 | ENSG00000143093 | 0.523150781 | 6.40E-11 |
| ENSG00000229152 | ENSG00000066427 | 0.58759684 | 5.48E-14 |
| ENSG00000229152 | ENSG00000274211 | 0.504206306 | 3.90E-10 |
| ENSG00000229152 | ENSG00000100354 | 0.592076516 | 3.16E-14 |
| ENSG00000229152 | ENSG00000160305 | 0.692690645 | 9.46E-21 |
| ENSG00000229152 | ENSG00000116539 | 0.509307486 | 2.42E-10 |
| ENSG00000229152 | ENSG00000163939 | 0.501203137 | 5.14E-10 |
| ENSG00000229152 | ENSG00000123066 | 0.54263176 | 8.85E-12 |
| ENSG00000229152 | ENSG00000197283 | 0.582233089 | 1.05E-13 |
| ENSG00000229152 | ENSG00000163516 | 0.612971535 | 2.16E-15 |
| ENSG00000229152 | ENSG00000100201 | 0.67753067 | 1.33E-19 |
| ENSG00000229152 | ENSG00000272886 | 0.532896729 | 2.42E-11 |
| ENSG00000229152 | ENSG00000266028 | 0.536419907 | 1.69E-11 |
| ENSG00000229152 | ENSG00000139990 | 0.540112692 | 1.15E-11 |
| ENSG00000229152 | ENSG00000074755 | 0.60874334 | 3.78E-15 |
| ENSG00000229152 | ENSG00000149187 | 0.574599458 | 2.58E-13 |
| ENSG00000229152 | ENSG00000224470 | 0.515119263 | 1.40E-10 |
| ENSG00000229152 | ENSG00000183955 | 0.508073318 | 2.72E-10 |
| ENSG00000229152 | ENSG00000103657 | 0.572713127 | 3.21E-13 |
| ENSG00000229152 | ENSG00000135365 | 0.590654523 | 3.77E-14 |
| ENSG00000230091 | ENSG00000176714 | 0.570886714 | 3.97E-13 |
| ENSG00000230149 | ENSG00000160218 | 0.545598119 | 6.48E-12 |
| ENSG00000230149 | ENSG00000076108 | 0.594510373 | 2.34E-14 |
| ENSG00000230149 | ENSG00000184787 | 0.571825 | 3.56E-13 |
| ENSG00000230149 | ENSG00000141503 | 0.523414341 | 6.24E-11 |
| ENSG00000230149 | ENSG00000166135 | 0.557438599 | 1.80E-12 |
| ENSG00000230149 | ENSG00000065526 | 0.547552671 | 5.26E-12 |
| ENSG00000230149 | ENSG00000108557 | 0.528309177 | 3.84E-11 |
| ENSG00000230149 | ENSG00000167522 | 0.579131696 | 1.52E-13 |
| ENSG00000230149 | ENSG00000005810 | 0.509338073 | 2.42E-10 |
| ENSG00000230149 | ENSG00000071054 | 0.5282117 | 3.87E-11 |
| ENSG00000230149 | ENSG00000196689 | 0.567618989 | 5.77E-13 |
| ENSG00000230149 | ENSG00000187605 | 0.617550599 | 1.17E-15 |
| ENSG00000230149 | ENSG00000152102 | 0.506332649 | 3.20E-10 |
| ENSG00000230149 | ENSG00000179335 | 0.504618108 | 3.75E-10 |
| ENSG00000230149 | ENSG00000175662 | 0.501779048 | 4.87E-10 |
| ENSG00000230149 | ENSG00000110237 | 0.537202443 | 1.56E-11 |
| ENSG00000230149 | ENSG00000078687 | 0.545648811 | 6.44E-12 |
| ENSG00000230149 | ENSG00000160294 | 0.60885469 | 3.72E-15 |
| ENSG00000230149 | ENSG00000213983 | 0.640954684 | 4.29E-17 |
| ENSG00000230149 | ENSG00000010322 | 0.617434623 | 1.19E-15 |
| ENSG00000230149 | ENSG00000134698 | 0.588531151 | 4.89E-14 |
| ENSG00000230149 | ENSG00000275023 | 0.612759916 | 2.22E-15 |
| ENSG00000230149 | ENSG00000008083 | 0.58837634 | 4.98E-14 |
| ENSG00000230149 | ENSG00000196233 | 0.542761512 | 8.73E-12 |
| ENSG00000230149 | ENSG00000154370 | 0.502714421 | 4.47E-10 |
| ENSG00000230149 | ENSG00000011021 | 0.561684024 | 1.13E-12 |
| ENSG00000230149 | ENSG00000086758 | 0.547118396 | 5.51E-12 |
| ENSG00000230149 | ENSG00000163939 | 0.519696588 | 8.97E-11 |
| ENSG00000230149 | ENSG00000197386 | 0.527526694 | 4.15E-11 |
| ENSG00000230149 | ENSG00000141252 | 0.562084787 | 1.08E-12 |
| ENSG00000230149 | ENSG00000148843 | 0.520636771 | 8.19E-11 |
| ENSG00000230149 | ENSG00000114648 | 0.504310346 | 3.86E-10 |
| ENSG00000230149 | ENSG00000133226 | 0.520806776 | 8.05E-11 |
| ENSG00000230149 | ENSG00000164068 | 0.591855438 | 3.25E-14 |
| ENSG00000230149 | ENSG00000166783 | 0.548469574 | 4.77E-12 |
| ENSG00000230149 | ENSG00000117713 | 0.594986072 | 2.20E-14 |
| ENSG00000230149 | ENSG00000142599 | 0.522336294 | 6.93E-11 |
| ENSG00000230551 | ENSG00000198920 | 0.552270449 | 3.17E-12 |
| ENSG00000230551 | ENSG00000038382 | 0.605762309 | 5.58E-15 |
| ENSG00000230551 | ENSG00000176953 | 0.613007042 | 2.15E-15 |
| ENSG00000230551 | ENSG00000160305 | 0.525590752 | 5.03E-11 |
| ENSG00000230551 | ENSG00000163945 | 0.62299418 | 5.56E-16 |
| ENSG00000230551 | ENSG00000141068 | 0.507805898 | 2.79E-10 |
| ENSG00000230551 | ENSG00000152520 | 0.686955051 | 2.62E-20 |
| ENSG00000230551 | ENSG00000061936 | 0.526381128 | 4.65E-11 |
| ENSG00000230551 | ENSG00000117834 | 0.612829237 | 2.20E-15 |
| ENSG00000230551 | ENSG00000108510 | 0.500535327 | 5.46E-10 |
| ENSG00000230551 | ENSG00000197217 | 0.506919353 | 3.03E-10 |
| ENSG00000230551 | ENSG00000108465 | 0.625869327 | 3.73E-16 |
| ENSG00000230551 | ENSG00000149187 | 0.509657834 | 2.34E-10 |
| ENSG00000230551 | ENSG00000188529 | 0.558227069 | 1.65E-12 |
| ENSG00000230551 | ENSG00000152223 | 0.506687948 | 3.10E-10 |
| ENSG00000230551 | ENSG00000134698 | 0.547547719 | 5.27E-12 |
| ENSG00000230551 | ENSG00000162664 | 0.515659269 | 1.33E-10 |
| ENSG00000230551 | ENSG00000164164 | 0.582199222 | 1.05E-13 |
| ENSG00000230551 | ENSG00000133226 | 0.599290657 | 1.28E-14 |
| ENSG00000230551 | ENSG00000013523 | 0.509620476 | 2.35E-10 |
| ENSG00000230551 | ENSG00000217128 | 0.568179517 | 5.41E-13 |
| ENSG00000230551 | ENSG00000156030 | 0.577016357 | 1.94E-13 |
| ENSG00000230551 | ENSG00000184787 | 0.577249048 | 1.89E-13 |
| ENSG00000230551 | ENSG00000239704 | 0.553634672 | 2.74E-12 |
| ENSG00000230551 | ENSG00000213983 | 0.50786691 | 2.77E-10 |
| ENSG00000230551 | ENSG00000163516 | 0.586455186 | 6.30E-14 |
| ENSG00000230551 | ENSG00000179979 | 0.524953728 | 5.36E-11 |
| ENSG00000230551 | ENSG00000169914 | 0.588241568 | 5.07E-14 |
| ENSG00000230551 | ENSG00000146963 | 0.533535353 | 2.26E-11 |
| ENSG00000230551 | ENSG00000164828 | 0.54264695 | 8.84E-12 |
| ENSG00000230551 | ENSG00000066933 | 0.512429894 | 1.80E-10 |
| ENSG00000230551 | ENSG00000066427 | 0.592511226 | 3.00E-14 |
| ENSG00000230551 | ENSG00000126870 | 0.556989495 | 1.90E-12 |
| ENSG00000230551 | ENSG00000143624 | 0.553780443 | 2.69E-12 |
| ENSG00000230551 | ENSG00000066739 | 0.548773549 | 4.62E-12 |
| ENSG00000230551 | ENSG00000159433 | 0.548762819 | 4.63E-12 |
| ENSG00000230551 | ENSG00000072364 | 0.502985502 | 4.36E-10 |
| ENSG00000230551 | ENSG00000112739 | 0.554907803 | 2.38E-12 |
| ENSG00000230551 | ENSG00000100201 | 0.641839841 | 3.77E-17 |
| ENSG00000230551 | ENSG00000102710 | 0.512123321 | 1.86E-10 |
| ENSG00000230551 | ENSG00000198677 | 0.532131457 | 2.61E-11 |
| ENSG00000230551 | ENSG00000102878 | 0.51649726 | 1.22E-10 |
| ENSG00000230551 | ENSG00000146909 | 0.522271059 | 6.98E-11 |
| ENSG00000230551 | ENSG00000111364 | 0.522752084 | 6.66E-11 |
| ENSG00000230555 | ENSG00000107854 | 0.508242396 | 2.68E-10 |
| ENSG00000230555 | ENSG00000166135 | 0.563329893 | 9.37E-13 |
| ENSG00000230565 | ENSG00000100813 | 0.591783061 | 3.28E-14 |
| ENSG00000230565 | ENSG00000086758 | 0.51870799 | 9.87E-11 |
| ENSG00000230565 | ENSG00000104517 | 0.583087269 | 9.46E-14 |
| ENSG00000230565 | ENSG00000152520 | 0.587504059 | 5.54E-14 |
| ENSG00000230565 | ENSG00000196233 | 0.670108171 | 4.60E-19 |
| ENSG00000230565 | ENSG00000078687 | 0.581820793 | 1.10E-13 |
| ENSG00000230565 | ENSG00000048028 | 0.541793128 | 9.67E-12 |
| ENSG00000230565 | ENSG00000132953 | 0.546921015 | 5.63E-12 |
| ENSG00000230565 | ENSG00000083168 | 0.532315356 | 2.56E-11 |
| ENSG00000230565 | ENSG00000166135 | 0.589769963 | 4.20E-14 |
| ENSG00000230565 | ENSG00000149187 | 0.514699615 | 1.45E-10 |
| ENSG00000230565 | ENSG00000066427 | 0.711219246 | 2.97E-22 |
| ENSG00000230565 | ENSG00000134698 | 0.675137527 | 1.99E-19 |
| ENSG00000230565 | ENSG00000075292 | 0.601624306 | 9.51E-15 |
| ENSG00000230565 | ENSG00000123066 | 0.530622797 | 3.04E-11 |
| ENSG00000230565 | ENSG00000100354 | 0.568751832 | 5.07E-13 |
| ENSG00000230565 | ENSG00000197312 | 0.576841609 | 1.99E-13 |
| ENSG00000230565 | ENSG00000158711 | 0.547273474 | 5.42E-12 |
| ENSG00000230565 | ENSG00000076685 | 0.500319498 | 5.57E-10 |
| ENSG00000230565 | ENSG00000066739 | 0.662729494 | 1.52E-18 |
| ENSG00000230565 | ENSG00000197653 | 0.555363865 | 2.27E-12 |
| ENSG00000230565 | ENSG00000197217 | 0.635751667 | 9.17E-17 |
| ENSG00000230565 | ENSG00000180667 | 0.604883266 | 6.25E-15 |
| ENSG00000230565 | ENSG00000108963 | 0.552735533 | 3.02E-12 |
| ENSG00000230565 | ENSG00000155858 | 0.524878121 | 5.40E-11 |
| ENSG00000230565 | ENSG00000154370 | 0.500130369 | 5.67E-10 |
| ENSG00000230565 | ENSG00000275023 | 0.600323149 | 1.12E-14 |
| ENSG00000230565 | ENSG00000176953 | 0.643042309 | 3.15E-17 |
| ENSG00000230565 | ENSG00000160305 | 0.685645986 | 3.30E-20 |
| ENSG00000230565 | ENSG00000234616 | 0.653811791 | 6.17E-18 |
| ENSG00000230565 | ENSG00000166436 | 0.53576124 | 1.80E-11 |
| ENSG00000230565 | ENSG00000197283 | 0.632177163 | 1.53E-16 |
| ENSG00000230565 | ENSG00000108510 | 0.51141219 | 1.99E-10 |
| ENSG00000230565 | ENSG00000156030 | 0.546385572 | 5.96E-12 |
| ENSG00000230565 | ENSG00000103657 | 0.596709821 | 1.77E-14 |
| ENSG00000230565 | ENSG00000066933 | 0.575129274 | 2.43E-13 |
| ENSG00000230565 | ENSG00000111707 | 0.532875463 | 2.42E-11 |
| ENSG00000230565 | ENSG00000140948 | 0.551768147 | 3.35E-12 |
| ENSG00000230565 | ENSG00000137337 | 0.56018319 | 1.33E-12 |
| ENSG00000230565 | ENSG00000167258 | 0.53124548 | 2.86E-11 |
| ENSG00000230565 | ENSG00000171634 | 0.613992212 | 1.89E-15 |
| ENSG00000230565 | ENSG00000110344 | 0.551118595 | 3.59E-12 |
| ENSG00000230565 | ENSG00000184787 | 0.659696904 | 2.46E-18 |
| ENSG00000230565 | ENSG00000115020 | 0.534036047 | 2.15E-11 |
| ENSG00000230565 | ENSG00000274211 | 0.533286757 | 2.32E-11 |
| ENSG00000230565 | ENSG00000144589 | 0.506433629 | 3.17E-10 |
| ENSG00000230565 | ENSG00000169914 | 0.694459879 | 6.87E-21 |
| ENSG00000230565 | ENSG00000019995 | 0.565841869 | 7.06E-13 |
| ENSG00000230565 | ENSG00000167110 | 0.524066667 | 5.85E-11 |
| ENSG00000230565 | ENSG00000155744 | 0.606986195 | 4.76E-15 |
| ENSG00000230565 | ENSG00000076108 | 0.589156892 | 4.53E-14 |
| ENSG00000230565 | ENSG00000177082 | 0.531156299 | 2.88E-11 |
| ENSG00000230565 | ENSG00000187605 | 0.646428703 | 1.90E-17 |
| ENSG00000230565 | ENSG00000074755 | 0.577436204 | 1.85E-13 |
| ENSG00000230565 | ENSG00000106086 | 0.509268143 | 2.43E-10 |
| ENSG00000230565 | ENSG00000008083 | 0.517752449 | 1.08E-10 |
| ENSG00000230565 | ENSG00000112739 | 0.588512024 | 4.90E-14 |
| ENSG00000230565 | ENSG00000108352 | 0.521925447 | 7.22E-11 |
| ENSG00000230565 | ENSG00000163939 | 0.557162922 | 1.86E-12 |
| ENSG00000230565 | ENSG00000077044 | 0.553213028 | 2.86E-12 |
| ENSG00000230565 | ENSG00000166860 | 0.573692493 | 2.87E-13 |
| ENSG00000231113 | ENSG00000102858 | 0.520601233 | 8.21E-11 |
| ENSG00000231113 | ENSG00000168488 | 0.540942278 | 1.06E-11 |
| ENSG00000231113 | ENSG00000108669 | 0.515343088 | 1.37E-10 |
| ENSG00000231113 | ENSG00000170921 | 0.502667165 | 4.49E-10 |
| ENSG00000231113 | ENSG00000180902 | 0.542337951 | 9.13E-12 |
| ENSG00000231113 | ENSG00000166436 | 0.519524764 | 9.12E-11 |
| ENSG00000231113 | ENSG00000187605 | 0.534522175 | 2.05E-11 |
| ENSG00000231113 | ENSG00000196547 | 0.507188395 | 2.95E-10 |
| ENSG00000231113 | ENSG00000160305 | 0.606155764 | 5.30E-15 |
| ENSG00000231113 | ENSG00000169914 | 0.554815191 | 2.41E-12 |
| ENSG00000231113 | ENSG00000168067 | 0.507970142 | 2.75E-10 |
| ENSG00000231113 | ENSG00000196689 | 0.502635222 | 4.51E-10 |
| ENSG00000231113 | ENSG00000125447 | 0.696300022 | 4.92E-21 |
| ENSG00000231113 | ENSG00000108963 | 0.594506566 | 2.34E-14 |
| ENSG00000231113 | ENSG00000266173 | 0.598557123 | 1.40E-14 |
| ENSG00000231113 | ENSG00000197283 | 0.57978233 | 1.40E-13 |
| ENSG00000231113 | ENSG00000141068 | 0.565752706 | 7.13E-13 |
| ENSG00000231113 | ENSG00000134698 | 0.648437515 | 1.40E-17 |
| ENSG00000231113 | ENSG00000117713 | 0.50874531 | 2.55E-10 |
| ENSG00000231113 | ENSG00000175662 | 0.519077899 | 9.53E-11 |
| ENSG00000231113 | ENSG00000184787 | 0.592465854 | 3.01E-14 |
| ENSG00000231113 | ENSG00000140948 | 0.512436992 | 1.80E-10 |
| ENSG00000231113 | ENSG00000143630 | 0.602375309 | 8.63E-15 |
| ENSG00000231113 | ENSG00000126464 | 0.515399671 | 1.36E-10 |
| ENSG00000231113 | ENSG00000076108 | 0.526639264 | 4.53E-11 |
| ENSG00000231113 | ENSG00000065526 | 0.520247652 | 8.50E-11 |
| ENSG00000231113 | ENSG00000177169 | 0.509391549 | 2.40E-10 |
| ENSG00000231113 | ENSG00000105662 | 0.500282598 | 5.59E-10 |
| ENSG00000231721 | ENSG00000120727 | 0.539437066 | 1.24E-11 |
| ENSG00000231721 | ENSG00000158985 | 0.535277484 | 1.90E-11 |
| ENSG00000231721 | ENSG00000013561 | 0.557974342 | 1.70E-12 |
| ENSG00000231721 | ENSG00000188010 | 0.550455091 | 3.86E-12 |
| ENSG00000231721 | ENSG00000153130 | 0.551630372 | 3.40E-12 |
| ENSG00000232415 | ENSG00000165084 | 0.55684769 | 1.93E-12 |
| ENSG00000232415 | ENSG00000111837 | 0.558789638 | 1.55E-12 |
| ENSG00000232504 | ENSG00000115525 | 0.682599875 | 5.60E-20 |
| ENSG00000233006 | ENSG00000036672 | 0.537677099 | 1.48E-11 |
| ENSG00000233038 | ENSG00000054611 | 0.50576734 | 3.37E-10 |
| ENSG00000233038 | ENSG00000197943 | 0.793763654 | 1.00E-30 |
| ENSG00000233038 | ENSG00000100092 | 0.569047986 | 4.90E-13 |
| ENSG00000233223 | ENSG00000161920 | 0.571257276 | 3.80E-13 |
| ENSG00000233478 | ENSG00000095110 | 0.53867346 | 1.34E-11 |
| ENSG00000233508 | ENSG00000169884 | 0.66904186 | 5.47E-19 |
| ENSG00000233508 | ENSG00000198794 | 0.546657998 | 5.79E-12 |
| ENSG00000233508 | ENSG00000123612 | 0.530919978 | 2.95E-11 |
| ENSG00000233508 | ENSG00000196189 | 0.500057569 | 5.71E-10 |
| ENSG00000233508 | ENSG00000135750 | 0.575080945 | 2.44E-13 |
| ENSG00000233508 | ENSG00000008735 | 0.545920031 | 6.26E-12 |
| ENSG00000233508 | ENSG00000100285 | 0.514889927 | 1.43E-10 |
| ENSG00000233508 | ENSG00000163995 | 0.653540793 | 6.44E-18 |
| ENSG00000233508 | ENSG00000050748 | 0.519292371 | 9.33E-11 |
| ENSG00000233508 | ENSG00000171517 | 0.510614211 | 2.14E-10 |
| ENSG00000233508 | ENSG00000181418 | 0.594645264 | 2.30E-14 |
| ENSG00000233508 | ENSG00000125814 | 0.698468266 | 3.31E-21 |
| ENSG00000233508 | ENSG00000148660 | 0.513703328 | 1.60E-10 |
| ENSG00000233508 | ENSG00000197885 | 0.540352942 | 1.12E-11 |
| ENSG00000233508 | ENSG00000147676 | 0.6270663 | 3.15E-16 |
| ENSG00000233508 | ENSG00000119946 | 0.653246194 | 6.74E-18 |
| ENSG00000233508 | ENSG00000165983 | 0.553112613 | 2.90E-12 |
| ENSG00000233508 | ENSG00000277363 | 0.625648478 | 3.85E-16 |
| ENSG00000233508 | ENSG00000177570 | 0.633306501 | 1.30E-16 |
| ENSG00000233508 | ENSG00000120053 | 0.561799391 | 1.11E-12 |
| ENSG00000233508 | ENSG00000006740 | 0.648325748 | 1.43E-17 |
| ENSG00000233508 | ENSG00000053108 | 0.607749923 | 4.30E-15 |
| ENSG00000233508 | ENSG00000174473 | 0.640163212 | 4.82E-17 |
| ENSG00000233705 | ENSG00000157152 | 0.565107301 | 7.67E-13 |
| ENSG00000233705 | ENSG00000148660 | 0.549756411 | 4.16E-12 |
| ENSG00000233705 | ENSG00000144290 | 0.656368378 | 4.15E-18 |
| ENSG00000233705 | ENSG00000149927 | 0.597836013 | 1.54E-14 |
| ENSG00000233705 | ENSG00000137843 | 0.568668461 | 5.12E-13 |
| ENSG00000233705 | ENSG00000164061 | 0.5290807 | 3.55E-11 |
| ENSG00000233705 | ENSG00000137766 | 0.603082008 | 7.88E-15 |
| ENSG00000233705 | ENSG00000007001 | 0.514557041 | 1.47E-10 |
| ENSG00000233705 | ENSG00000123612 | 0.531031433 | 2.92E-11 |
| ENSG00000233705 | ENSG00000130540 | 0.5896071 | 4.29E-14 |
| ENSG00000233705 | ENSG00000072657 | 0.637401266 | 7.22E-17 |
| ENSG00000233705 | ENSG00000078328 | 0.569325462 | 4.75E-13 |
| ENSG00000233705 | ENSG00000128656 | 0.673717585 | 2.53E-19 |
| ENSG00000233705 | ENSG00000136535 | 0.648008907 | 1.50E-17 |
| ENSG00000233705 | ENSG00000168539 | 0.54367133 | 7.94E-12 |
| ENSG00000233705 | ENSG00000053108 | 0.577977806 | 1.74E-13 |
| ENSG00000233705 | ENSG00000107954 | 0.592451226 | 3.02E-14 |
| ENSG00000233705 | ENSG00000147676 | 0.654330638 | 5.70E-18 |
| ENSG00000233705 | ENSG00000163630 | 0.59183055 | 3.26E-14 |
| ENSG00000233705 | ENSG00000006740 | 0.564732173 | 8.00E-13 |
| ENSG00000233705 | ENSG00000065325 | 0.619900728 | 8.49E-16 |
| ENSG00000233705 | ENSG00000116254 | 0.56060828 | 1.27E-12 |
| ENSG00000233705 | ENSG00000104722 | 0.503943295 | 3.99E-10 |
| ENSG00000233705 | ENSG00000074706 | 0.543920967 | 7.73E-12 |
| ENSG00000233705 | ENSG00000135423 | 0.614838331 | 1.68E-15 |
| ENSG00000233705 | ENSG00000181418 | 0.538609999 | 1.35E-11 |
| ENSG00000233705 | ENSG00000125814 | 0.599680125 | 1.22E-14 |
| ENSG00000233862 | ENSG00000129214 | 0.583085977 | 9.46E-14 |
| ENSG00000233862 | ENSG00000117400 | 0.521985992 | 7.17E-11 |
| ENSG00000233930 | ENSG00000125814 | 0.533457126 | 2.28E-11 |
| ENSG00000233930 | ENSG00000178233 | 0.558080587 | 1.68E-12 |
| ENSG00000233930 | ENSG00000197753 | 0.574550061 | 2.60E-13 |
| ENSG00000234817 | ENSG00000166689 | 0.539717384 | 1.20E-11 |
| ENSG00000234817 | ENSG00000135773 | 0.510336478 | 2.20E-10 |
| ENSG00000234817 | ENSG00000109501 | 0.50287035 | 4.41E-10 |
| ENSG00000235024 | ENSG00000108352 | 0.523879328 | 5.96E-11 |
| ENSG00000235106 | ENSG00000132881 | 0.517068579 | 1.16E-10 |
| ENSG00000235106 | ENSG00000146038 | 0.517181171 | 1.14E-10 |
| ENSG00000235106 | ENSG00000197557 | 0.595766916 | 2.00E-14 |
| ENSG00000235106 | ENSG00000064199 | 0.645719145 | 2.11E-17 |
| ENSG00000235106 | ENSG00000132376 | 0.544173642 | 7.53E-12 |
| ENSG00000235106 | ENSG00000273045 | 0.533076121 | 2.37E-11 |
| ENSG00000235106 | ENSG00000196659 | 0.559605282 | 1.42E-12 |
| ENSG00000235106 | ENSG00000164542 | 0.52953448 | 3.39E-11 |
| ENSG00000235106 | ENSG00000204852 | 0.510993822 | 2.07E-10 |
| ENSG00000235106 | ENSG00000130413 | 0.523264737 | 6.33E-11 |
| ENSG00000235106 | ENSG00000116885 | 0.572940035 | 3.13E-13 |
| ENSG00000235106 | ENSG00000103599 | 0.566200857 | 6.78E-13 |
| ENSG00000235123 | ENSG00000185002 | 0.57393543 | 2.79E-13 |
| ENSG00000235123 | ENSG00000104848 | 0.59774648 | 1.56E-14 |
| ENSG00000235180 | ENSG00000182718 | 0.580409525 | 1.30E-13 |
| ENSG00000235423 | ENSG00000151276 | 0.524383386 | 5.67E-11 |
| ENSG00000235423 | ENSG00000135299 | 0.526521331 | 4.59E-11 |
| ENSG00000235423 | ENSG00000186660 | 0.530708484 | 3.02E-11 |
| ENSG00000235423 | ENSG00000130921 | 0.650094268 | 1.09E-17 |
| ENSG00000235423 | ENSG00000143093 | 0.538740154 | 1.33E-11 |
| ENSG00000235423 | ENSG00000135090 | 0.511837355 | 1.91E-10 |
| ENSG00000235423 | ENSG00000118007 | 0.521116119 | 7.81E-11 |
| ENSG00000235423 | ENSG00000115109 | 0.518808371 | 9.78E-11 |
| ENSG00000235423 | ENSG00000048028 | 0.542659149 | 8.83E-12 |
| ENSG00000235731 | ENSG00000185002 | 0.57842136 | 1.65E-13 |
| ENSG00000235731 | ENSG00000124157 | 0.594293317 | 2.40E-14 |
| ENSG00000235731 | ENSG00000117834 | 0.509421911 | 2.40E-10 |
| ENSG00000235733 | ENSG00000145779 | 0.52701575 | 4.37E-11 |
| ENSG00000236117 | ENSG00000120053 | 0.524596969 | 5.55E-11 |
| ENSG00000236333 | ENSG00000102003 | 0.520545423 | 8.26E-11 |
| ENSG00000236333 | ENSG00000132938 | 0.790192623 | 2.78E-30 |
| ENSG00000236333 | ENSG00000123612 | 0.545229842 | 6.74E-12 |
| ENSG00000236333 | ENSG00000135423 | 0.689308538 | 1.73E-20 |
| ENSG00000236333 | ENSG00000135750 | 0.581651608 | 1.12E-13 |
| ENSG00000236333 | ENSG00000189241 | 0.579926337 | 1.38E-13 |
| ENSG00000236333 | ENSG00000123901 | 0.781486938 | 3.09E-29 |
| ENSG00000236333 | ENSG00000213424 | 0.774318357 | 2.07E-28 |
| ENSG00000236333 | ENSG00000176194 | 0.721731948 | 3.67E-23 |
| ENSG00000236333 | ENSG00000107758 | 0.578813431 | 1.57E-13 |
| ENSG00000236333 | ENSG00000120053 | 0.628455019 | 2.60E-16 |
| ENSG00000236333 | ENSG00000130540 | 0.658891826 | 2.79E-18 |
| ENSG00000236333 | ENSG00000171132 | 0.61331894 | 2.06E-15 |
| ENSG00000236333 | ENSG00000107130 | 0.506579348 | 3.13E-10 |
| ENSG00000236333 | ENSG00000163630 | 0.647384766 | 1.65E-17 |
| ENSG00000236333 | ENSG00000114757 | 0.582197365 | 1.05E-13 |
| ENSG00000236333 | ENSG00000178394 | 0.655799049 | 4.54E-18 |
| ENSG00000236333 | ENSG00000141668 | 0.709763501 | 3.93E-22 |
| ENSG00000236333 | ENSG00000149927 | 0.549928988 | 4.08E-12 |
| ENSG00000236333 | ENSG00000198513 | 0.541062722 | 1.04E-11 |
| ENSG00000236333 | ENSG00000100307 | 0.501591922 | 4.96E-10 |
| ENSG00000236333 | ENSG00000111249 | 0.702502007 | 1.56E-21 |
| ENSG00000236333 | ENSG00000138101 | 0.554546148 | 2.48E-12 |
| ENSG00000236333 | ENSG00000198794 | 0.50000198 | 5.73E-10 |
| ENSG00000236333 | ENSG00000137766 | 0.764784192 | 2.34E-27 |
| ENSG00000236333 | ENSG00000136854 | 0.605299738 | 5.92E-15 |
| ENSG00000236333 | ENSG00000122012 | 0.505733967 | 3.38E-10 |
| ENSG00000236333 | ENSG00000053108 | 0.656823185 | 3.87E-18 |
| ENSG00000236333 | ENSG00000159753 | 0.760838331 | 6.16E-27 |
| ENSG00000236333 | ENSG00000125814 | 0.63279545 | 1.40E-16 |
| ENSG00000236333 | ENSG00000178233 | 0.529764301 | 3.32E-11 |
| ENSG00000236333 | ENSG00000127585 | 0.56926286 | 4.78E-13 |
| ENSG00000236333 | ENSG00000154118 | 0.53629648 | 1.71E-11 |
| ENSG00000236333 | ENSG00000156076 | 0.559891094 | 1.38E-12 |
| ENSG00000236333 | ENSG00000164506 | 0.576697752 | 2.02E-13 |
| ENSG00000236333 | ENSG00000108924 | 0.560933396 | 1.23E-12 |
| ENSG00000236333 | ENSG00000116675 | 0.554897918 | 2.38E-12 |
| ENSG00000236333 | ENSG00000157782 | 0.65199869 | 8.16E-18 |
| ENSG00000236333 | ENSG00000165983 | 0.612192776 | 2.40E-15 |
| ENSG00000236333 | ENSG00000169884 | 0.688861076 | 1.87E-20 |
| ENSG00000236790 | ENSG00000204659 | 0.520947226 | 7.94E-11 |
| ENSG00000236790 | ENSG00000115339 | 0.502953735 | 4.38E-10 |
| ENSG00000236790 | ENSG00000188039 | 0.590128157 | 4.02E-14 |
| ENSG00000237037 | ENSG00000273899 | 0.631025976 | 1.80E-16 |
| ENSG00000237574 | ENSG00000147676 | 0.571910656 | 3.53E-13 |
| ENSG00000237574 | ENSG00000123612 | 0.591495405 | 3.40E-14 |
| ENSG00000237574 | ENSG00000119946 | 0.574370098 | 2.65E-13 |
| ENSG00000237574 | ENSG00000177570 | 0.509251484 | 2.44E-10 |
| ENSG00000237574 | ENSG00000165983 | 0.578752756 | 1.58E-13 |
| ENSG00000237574 | ENSG00000171517 | 0.62664902 | 3.34E-16 |
| ENSG00000237720 | ENSG00000136854 | 0.519973596 | 8.73E-11 |
| ENSG00000237720 | ENSG00000137843 | 0.546244528 | 6.05E-12 |
| ENSG00000237720 | ENSG00000065325 | 0.770377818 | 5.72E-28 |
| ENSG00000237720 | ENSG00000135750 | 0.602116946 | 8.92E-15 |
| ENSG00000237720 | ENSG00000171126 | 0.514768818 | 1.44E-10 |
| ENSG00000237720 | ENSG00000165983 | 0.663155062 | 1.42E-18 |
| ENSG00000237720 | ENSG00000167971 | 0.556375246 | 2.03E-12 |
| ENSG00000237720 | ENSG00000172350 | 0.53218683 | 2.60E-11 |
| ENSG00000237720 | ENSG00000135423 | 0.653953279 | 6.04E-18 |
| ENSG00000237720 | ENSG00000053108 | 0.721129595 | 4.15E-23 |
| ENSG00000244968 | ENSG00000162174 | 0.587690887 | 5.42E-14 |
| ENSG00000244968 | ENSG00000144306 | 0.527049596 | 4.35E-11 |
| ENSG00000244968 | ENSG00000176714 | 0.521523539 | 7.51E-11 |
| ENSG00000244968 | ENSG00000133318 | 0.56003824 | 1.35E-12 |
| ENSG00000244968 | ENSG00000157087 | 0.501249169 | 5.12E-10 |
| ENSG00000244968 | ENSG00000146122 | 0.504891136 | 3.66E-10 |
| ENSG00000244968 | ENSG00000089486 | 0.622379056 | 6.05E-16 |
| ENSG00000244968 | ENSG00000184602 | 0.604663661 | 6.43E-15 |
| ENSG00000244968 | ENSG00000197557 | 0.546068143 | 6.16E-12 |
| ENSG00000245248 | ENSG00000116885 | 0.741977874 | 4.96E-25 |
| ENSG00000245248 | ENSG00000036672 | 0.506180954 | 3.25E-10 |
| ENSG00000245248 | ENSG00000137699 | 0.516710132 | 1.20E-10 |
| ENSG00000245248 | ENSG00000175318 | 0.539760893 | 1.19E-11 |
| ENSG00000245248 | ENSG00000149483 | 0.56409504 | 8.60E-13 |
| ENSG00000245248 | ENSG00000186976 | 0.574682247 | 2.56E-13 |
| ENSG00000245248 | ENSG00000106125 | 0.576440317 | 2.08E-13 |
| ENSG00000245248 | ENSG00000214688 | 0.691709999 | 1.13E-20 |
| ENSG00000245248 | ENSG00000157796 | 0.503320899 | 4.23E-10 |
| ENSG00000245248 | ENSG00000183831 | 0.679793459 | 9.07E-20 |
| ENSG00000245248 | ENSG00000164542 | 0.547710304 | 5.18E-12 |
| ENSG00000245248 | ENSG00000197557 | 0.69857553 | 3.24E-21 |
| ENSG00000245248 | ENSG00000204852 | 0.570427812 | 4.18E-13 |
| ENSG00000245248 | ENSG00000174628 | 0.548771578 | 4.62E-12 |
| ENSG00000245573 | ENSG00000175575 | 0.57310039 | 3.07E-13 |
| ENSG00000245573 | ENSG00000197557 | 0.512868675 | 1.73E-10 |
| ENSG00000245573 | ENSG00000021300 | 0.603077933 | 7.89E-15 |
| ENSG00000246100 | ENSG00000189067 | 0.502919756 | 4.39E-10 |
| ENSG00000246100 | ENSG00000111885 | 0.507618619 | 2.84E-10 |
| ENSG00000246100 | ENSG00000143416 | 0.52523163 | 5.21E-11 |
| ENSG00000246250 | ENSG00000159433 | 0.502401946 | 4.60E-10 |
| ENSG00000246263 | ENSG00000188010 | 0.534234383 | 2.11E-11 |
| ENSG00000246263 | ENSG00000173467 | 0.509546242 | 2.37E-10 |
| ENSG00000246273 | ENSG00000141198 | 0.584252655 | 8.22E-14 |
| ENSG00000246273 | ENSG00000164542 | 0.527019764 | 4.36E-11 |
| ENSG00000246273 | ENSG00000149050 | 0.569525622 | 4.64E-13 |
| ENSG00000246273 | ENSG00000145358 | 0.516780162 | 1.19E-10 |
| ENSG00000246273 | ENSG00000197557 | 0.532311401 | 2.56E-11 |
| ENSG00000246273 | ENSG00000204852 | 0.536840683 | 1.62E-11 |
| ENSG00000246731 | ENSG00000174238 | 0.537927181 | 1.44E-11 |
| ENSG00000246731 | ENSG00000088367 | 0.502507683 | 4.56E-10 |
| ENSG00000246731 | ENSG00000257093 | 0.546127068 | 6.12E-12 |
| ENSG00000246731 | ENSG00000184602 | 0.62905951 | 2.38E-16 |
| ENSG00000246731 | ENSG00000185722 | 0.518582464 | 1.00E-10 |
| ENSG00000246731 | ENSG00000133065 | 0.528699906 | 3.69E-11 |
| ENSG00000246731 | ENSG00000148660 | 0.596361978 | 1.85E-14 |
| ENSG00000246731 | ENSG00000132613 | 0.514930996 | 1.42E-10 |
| ENSG00000246731 | ENSG00000123836 | 0.522991423 | 6.50E-11 |
| ENSG00000246731 | ENSG00000172379 | 0.528607649 | 3.72E-11 |
| ENSG00000246731 | ENSG00000185818 | 0.52136033 | 7.63E-11 |
| ENSG00000246731 | ENSG00000047849 | 0.518880347 | 9.71E-11 |
| ENSG00000247556 | ENSG00000104133 | 0.583286764 | 9.23E-14 |
| ENSG00000247556 | ENSG00000104067 | 0.530236564 | 3.16E-11 |
| ENSG00000247556 | ENSG00000138592 | 0.590568509 | 3.81E-14 |
| ENSG00000247556 | ENSG00000198146 | 0.595691405 | 2.02E-14 |
| ENSG00000247556 | ENSG00000138593 | 0.612549338 | 2.29E-15 |
| ENSG00000247556 | ENSG00000137815 | 0.625634797 | 3.85E-16 |
| ENSG00000247735 | ENSG00000139445 | 0.598695409 | 1.38E-14 |
| ENSG00000247735 | ENSG00000081026 | 0.538517704 | 1.36E-11 |
| ENSG00000247735 | ENSG00000085644 | 0.508507429 | 2.61E-10 |
| ENSG00000247735 | ENSG00000196323 | 0.548377326 | 4.82E-12 |
| ENSG00000247735 | ENSG00000113194 | 0.527409224 | 4.20E-11 |
| ENSG00000247735 | ENSG00000149548 | 0.599951283 | 1.18E-14 |
| ENSG00000247735 | ENSG00000122966 | 0.534753791 | 2.00E-11 |
| ENSG00000247796 | ENSG00000108439 | 0.557254798 | 1.84E-12 |
| ENSG00000247796 | ENSG00000111275 | 0.513939591 | 1.56E-10 |
| ENSG00000247796 | ENSG00000148660 | 0.555817753 | 2.16E-12 |
| ENSG00000247796 | ENSG00000197557 | 0.724064702 | 2.28E-23 |
| ENSG00000247796 | ENSG00000176714 | 0.644521399 | 2.53E-17 |
| ENSG00000247796 | ENSG00000064199 | 0.596566838 | 1.81E-14 |
| ENSG00000247796 | ENSG00000118096 | 0.517312897 | 1.13E-10 |
| ENSG00000247796 | ENSG00000143107 | 0.525783447 | 4.93E-11 |
| ENSG00000247796 | ENSG00000154319 | 0.517608577 | 1.10E-10 |
| ENSG00000247796 | ENSG00000237651 | 0.562282385 | 1.05E-12 |
| ENSG00000247796 | ENSG00000165084 | 0.619265858 | 9.26E-16 |
| ENSG00000247796 | ENSG00000138036 | 0.60558795 | 5.70E-15 |
| ENSG00000247796 | ENSG00000116885 | 0.569186394 | 4.82E-13 |
| ENSG00000247796 | ENSG00000149483 | 0.540620252 | 1.09E-11 |
| ENSG00000247796 | ENSG00000106125 | 0.519863596 | 8.83E-11 |
| ENSG00000247796 | ENSG00000120727 | 0.5049544 | 3.64E-10 |
| ENSG00000247796 | ENSG00000214688 | 0.578632625 | 1.61E-13 |
| ENSG00000247796 | ENSG00000021300 | 0.563375228 | 9.33E-13 |
| ENSG00000247796 | ENSG00000036672 | 0.525413524 | 5.12E-11 |
| ENSG00000247796 | ENSG00000198130 | 0.530711395 | 3.01E-11 |
| ENSG00000247796 | ENSG00000064652 | 0.555665399 | 2.19E-12 |
| ENSG00000248508 | ENSG00000181513 | 0.551120158 | 3.59E-12 |
| ENSG00000248596 | ENSG00000121067 | 0.5128666 | 1.73E-10 |
| ENSG00000248738 | ENSG00000167037 | 0.53955714 | 1.22E-11 |
| ENSG00000248810 | ENSG00000115919 | 0.553806531 | 2.68E-12 |
| ENSG00000248810 | ENSG00000173890 | 0.503865756 | 4.02E-10 |
| ENSG00000248810 | ENSG00000100055 | 0.509567495 | 2.36E-10 |
| ENSG00000248810 | ENSG00000161955 | 0.509844149 | 2.30E-10 |
| ENSG00000248810 | ENSG00000266094 | 0.529383927 | 3.45E-11 |
| ENSG00000248810 | ENSG00000135480 | 0.538559328 | 1.35E-11 |
| ENSG00000248925 | ENSG00000184154 | 0.658917859 | 2.78E-18 |
| ENSG00000248925 | ENSG00000276023 | 0.56580315 | 7.09E-13 |
| ENSG00000249456 | ENSG00000072364 | 0.503650198 | 4.10E-10 |
| ENSG00000249456 | ENSG00000169925 | 0.556006676 | 2.11E-12 |
| ENSG00000249456 | ENSG00000166025 | 0.526810513 | 4.46E-11 |
| ENSG00000249456 | ENSG00000053254 | 0.533536225 | 2.26E-11 |
| ENSG00000249456 | ENSG00000108091 | 0.504914413 | 3.65E-10 |
| ENSG00000249456 | ENSG00000116199 | 0.523193261 | 6.37E-11 |
| ENSG00000249456 | ENSG00000160294 | 0.513457373 | 1.64E-10 |
| ENSG00000249456 | ENSG00000107854 | 0.56720284 | 6.05E-13 |
| ENSG00000249456 | ENSG00000170759 | 0.591929562 | 3.22E-14 |
| ENSG00000249456 | ENSG00000072501 | 0.523419325 | 6.23E-11 |
| ENSG00000249456 | ENSG00000166860 | 0.529612698 | 3.37E-11 |
| ENSG00000249456 | ENSG00000123066 | 0.557319992 | 1.83E-12 |
| ENSG00000249456 | ENSG00000037749 | 0.577767921 | 1.78E-13 |
| ENSG00000249456 | ENSG00000155640 | 0.585104541 | 7.42E-14 |
| ENSG00000249456 | ENSG00000067900 | 0.54101005 | 1.05E-11 |
| ENSG00000249456 | ENSG00000139436 | 0.626831396 | 3.26E-16 |
| ENSG00000249456 | ENSG00000118007 | 0.55977003 | 1.39E-12 |
| ENSG00000249456 | ENSG00000065526 | 0.509428012 | 2.40E-10 |
| ENSG00000249456 | ENSG00000138031 | 0.505999883 | 3.30E-10 |
| ENSG00000249456 | ENSG00000170832 | 0.57280092 | 3.18E-13 |
| ENSG00000249456 | ENSG00000176986 | 0.732222503 | 4.14E-24 |
| ENSG00000249456 | ENSG00000038358 | 0.550519795 | 3.83E-12 |
| ENSG00000249456 | ENSG00000066135 | 0.541671233 | 9.79E-12 |
| ENSG00000249456 | ENSG00000162885 | 0.572366611 | 3.35E-13 |
| ENSG00000249456 | ENSG00000138081 | 0.579014209 | 1.54E-13 |
| ENSG00000249456 | ENSG00000172534 | 0.55068986 | 3.76E-12 |
| ENSG00000249456 | ENSG00000166197 | 0.522188012 | 7.03E-11 |
| ENSG00000249456 | ENSG00000164068 | 0.506890659 | 3.04E-10 |
| ENSG00000249456 | ENSG00000005339 | 0.548419378 | 4.80E-12 |
| ENSG00000249456 | ENSG00000065060 | 0.573756306 | 2.85E-13 |
| ENSG00000249673 | ENSG00000143126 | 0.563349911 | 9.35E-13 |
| ENSG00000249673 | ENSG00000159363 | 0.556782521 | 1.94E-12 |
| ENSG00000249673 | ENSG00000117408 | 0.559020982 | 1.52E-12 |
| ENSG00000249673 | ENSG00000173011 | 0.502465993 | 4.58E-10 |
| ENSG00000249673 | ENSG00000109066 | 0.500871552 | 5.30E-10 |
| ENSG00000249673 | ENSG00000109501 | 0.506745404 | 3.08E-10 |
| ENSG00000249673 | ENSG00000101298 | 0.507124408 | 2.97E-10 |
| ENSG00000249673 | ENSG00000084710 | 0.547661137 | 5.20E-12 |
| ENSG00000249673 | ENSG00000159788 | 0.514325858 | 1.51E-10 |
| ENSG00000249673 | ENSG00000179010 | 0.570727276 | 4.04E-13 |
| ENSG00000249673 | ENSG00000197283 | 0.50111825 | 5.18E-10 |
| ENSG00000249673 | ENSG00000147799 | 0.608929624 | 3.69E-15 |
| ENSG00000249673 | ENSG00000164880 | 0.508991677 | 2.50E-10 |
| ENSG00000249673 | ENSG00000054793 | 0.531143349 | 2.89E-11 |
| ENSG00000249673 | ENSG00000163930 | 0.534364502 | 2.08E-11 |
| ENSG00000249673 | ENSG00000184602 | 0.561395751 | 1.16E-12 |
| ENSG00000249673 | ENSG00000141564 | 0.536658887 | 1.65E-11 |
| ENSG00000249673 | ENSG00000177728 | 0.579862797 | 1.39E-13 |
| ENSG00000249673 | ENSG00000184014 | 0.543591047 | 8.01E-12 |
| ENSG00000249740 | ENSG00000182718 | 0.515146742 | 1.39E-10 |
| ENSG00000249896 | ENSG00000084764 | 0.642370053 | 3.48E-17 |
| ENSG00000249896 | ENSG00000136854 | 0.563775684 | 8.91E-13 |
| ENSG00000249896 | ENSG00000130540 | 0.516734603 | 1.19E-10 |
| ENSG00000250007 | ENSG00000107130 | 0.501370431 | 5.06E-10 |
| ENSG00000250007 | ENSG00000158856 | 0.509135698 | 2.46E-10 |
| ENSG00000250056 | ENSG00000152078 | 0.507746223 | 2.80E-10 |
| ENSG00000250584 | ENSG00000197106 | 0.777889481 | 8.10E-29 |
| ENSG00000250584 | ENSG00000181418 | 0.694310019 | 7.06E-21 |
| ENSG00000250584 | ENSG00000133816 | 0.550469936 | 3.85E-12 |
| ENSG00000250584 | ENSG00000172350 | 0.632183563 | 1.53E-16 |
| ENSG00000250584 | ENSG00000011347 | 0.541257377 | 1.02E-11 |
| ENSG00000250584 | ENSG00000069424 | 0.656517396 | 4.05E-18 |
| ENSG00000250584 | ENSG00000137843 | 0.582534061 | 1.01E-13 |
| ENSG00000250584 | ENSG00000167971 | 0.519332498 | 9.29E-11 |
| ENSG00000250584 | ENSG00000136854 | 0.593662503 | 2.60E-14 |
| ENSG00000250584 | ENSG00000125814 | 0.773794194 | 2.37E-28 |
| ENSG00000250584 | ENSG00000175352 | 0.605618476 | 5.68E-15 |
| ENSG00000250584 | ENSG00000156642 | 0.501779067 | 4.87E-10 |
| ENSG00000250584 | ENSG00000260001 | 0.633454443 | 1.28E-16 |
| ENSG00000250899 | ENSG00000075426 | 0.528079711 | 3.93E-11 |
| ENSG00000250903 | ENSG00000197557 | 0.518582875 | 1.00E-10 |
| ENSG00000251293 | ENSG00000132938 | 0.716409609 | 1.07E-22 |
| ENSG00000251293 | ENSG00000120053 | 0.581516023 | 1.14E-13 |
| ENSG00000251293 | ENSG00000165983 | 0.56792011 | 5.58E-13 |
| ENSG00000251293 | ENSG00000171517 | 0.607555199 | 4.42E-15 |
| ENSG00000251293 | ENSG00000137843 | 0.675530684 | 1.87E-19 |
| ENSG00000251293 | ENSG00000111249 | 0.605245249 | 5.96E-15 |
| ENSG00000251293 | ENSG00000060140 | 0.735243474 | 2.17E-24 |
| ENSG00000251293 | ENSG00000107758 | 0.567748755 | 5.69E-13 |
| ENSG00000251293 | ENSG00000125814 | 0.530734787 | 3.01E-11 |
| ENSG00000251293 | ENSG00000116254 | 0.562772279 | 9.98E-13 |
| ENSG00000251293 | ENSG00000157782 | 0.554758212 | 2.42E-12 |
| ENSG00000251293 | ENSG00000123360 | 0.505603943 | 3.42E-10 |
| ENSG00000251442 | ENSG00000204252 | 0.596358783 | 1.85E-14 |
| ENSG00000251442 | ENSG00000100055 | 0.59623345 | 1.88E-14 |
| ENSG00000251442 | ENSG00000135838 | 0.606076915 | 5.35E-15 |
| ENSG00000251442 | ENSG00000175857 | 0.664748542 | 1.10E-18 |
| ENSG00000251442 | ENSG00000183283 | 0.552749183 | 3.01E-12 |
| ENSG00000251442 | ENSG00000167642 | 0.533193383 | 2.35E-11 |
| ENSG00000251442 | ENSG00000158985 | 0.60458297 | 6.50E-15 |
| ENSG00000251442 | ENSG00000107738 | 0.693125392 | 8.75E-21 |
| ENSG00000251442 | ENSG00000143119 | 0.702930743 | 1.44E-21 |
| ENSG00000251442 | ENSG00000137841 | 0.522408235 | 6.88E-11 |
| ENSG00000251442 | ENSG00000102879 | 0.541235705 | 1.02E-11 |
| ENSG00000251442 | ENSG00000143851 | 0.508017334 | 2.73E-10 |
| ENSG00000251442 | ENSG00000173890 | 0.583445137 | 9.06E-14 |
| ENSG00000251442 | ENSG00000132965 | 0.560533402 | 1.28E-12 |
| ENSG00000251442 | ENSG00000136167 | 0.516390154 | 1.24E-10 |
| ENSG00000251442 | ENSG00000087253 | 0.579642036 | 1.43E-13 |
| ENSG00000251442 | ENSG00000021355 | 0.600246861 | 1.13E-14 |
| ENSG00000253161 | ENSG00000140564 | 0.565943286 | 6.98E-13 |
| ENSG00000253161 | ENSG00000086062 | 0.581456675 | 1.15E-13 |
| ENSG00000253161 | ENSG00000111424 | 0.529102615 | 3.54E-11 |
| ENSG00000253161 | ENSG00000133816 | 0.530085983 | 3.21E-11 |
| ENSG00000253161 | ENSG00000111885 | 0.586764583 | 6.06E-14 |
| ENSG00000253161 | ENSG00000122359 | 0.549344955 | 4.35E-12 |
| ENSG00000253477 | ENSG00000111371 | 0.53029604 | 3.14E-11 |
| ENSG00000253477 | ENSG00000196233 | 0.505632276 | 3.42E-10 |
| ENSG00000253477 | ENSG00000136854 | 0.539953447 | 1.17E-11 |
| ENSG00000253477 | ENSG00000168214 | 0.535175234 | 1.92E-11 |
| ENSG00000254154 | ENSG00000104517 | 0.51143509 | 1.98E-10 |
| ENSG00000254154 | ENSG00000160305 | 0.501117939 | 5.18E-10 |
| ENSG00000254154 | ENSG00000063438 | 0.553735501 | 2.71E-12 |
| ENSG00000254154 | ENSG00000171634 | 0.546142651 | 6.11E-12 |
| ENSG00000254165 | ENSG00000198198 | 0.563722254 | 8.97E-13 |
| ENSG00000254165 | ENSG00000111364 | 0.542305863 | 9.16E-12 |
| ENSG00000254165 | ENSG00000166436 | 0.538287344 | 1.39E-11 |
| ENSG00000254165 | ENSG00000169914 | 0.641625604 | 3.89E-17 |
| ENSG00000254165 | ENSG00000188529 | 0.532774286 | 2.45E-11 |
| ENSG00000254165 | ENSG00000176953 | 0.588951987 | 4.64E-14 |
| ENSG00000254165 | ENSG00000184787 | 0.575303382 | 2.38E-13 |
| ENSG00000254165 | ENSG00000197283 | 0.537575845 | 1.50E-11 |
| ENSG00000254165 | ENSG00000066427 | 0.518776321 | 9.81E-11 |
| ENSG00000254165 | ENSG00000160299 | 0.521721323 | 7.36E-11 |
| ENSG00000254165 | ENSG00000143624 | 0.509897714 | 2.29E-10 |
| ENSG00000254165 | ENSG00000133226 | 0.56452171 | 8.20E-13 |
| ENSG00000254187 | ENSG00000147676 | 0.814669628 | 1.65E-33 |
| ENSG00000254187 | ENSG00000153933 | 0.579688166 | 1.42E-13 |
| ENSG00000254187 | ENSG00000109466 | 0.508805922 | 2.54E-10 |
| ENSG00000254187 | ENSG00000135750 | 0.698995343 | 3.00E-21 |
| ENSG00000254187 | ENSG00000166501 | 0.582090263 | 1.07E-13 |
| ENSG00000254187 | ENSG00000004660 | 0.662301819 | 1.63E-18 |
| ENSG00000254187 | ENSG00000100307 | 0.514585434 | 1.47E-10 |
| ENSG00000254187 | ENSG00000107758 | 0.670258219 | 4.48E-19 |
| ENSG00000254211 | ENSG00000123612 | 0.549670988 | 4.20E-12 |
| ENSG00000254211 | ENSG00000154319 | 0.579999674 | 1.37E-13 |
| ENSG00000254389 | ENSG00000116205 | 0.518392656 | 1.02E-10 |
| ENSG00000254561 | ENSG00000214688 | 0.528485871 | 3.77E-11 |
| ENSG00000254561 | ENSG00000167371 | 0.509818506 | 2.31E-10 |
| ENSG00000254815 | ENSG00000179335 | 0.50828326 | 2.67E-10 |
| ENSG00000254815 | ENSG00000262304 | 0.567582752 | 5.79E-13 |
| ENSG00000254815 | ENSG00000178188 | 0.518602213 | 9.98E-11 |
| ENSG00000254815 | ENSG00000235194 | 0.588431267 | 4.95E-14 |
| ENSG00000254815 | ENSG00000196689 | 0.655612608 | 4.67E-18 |
| ENSG00000254815 | ENSG00000180902 | 0.565632728 | 7.23E-13 |
| ENSG00000254973 | ENSG00000107758 | 0.562063922 | 1.08E-12 |
| ENSG00000254973 | ENSG00000125814 | 0.584183538 | 8.29E-14 |
| ENSG00000254973 | ENSG00000166501 | 0.599703306 | 1.21E-14 |
| ENSG00000254973 | ENSG00000147676 | 0.667157821 | 7.44E-19 |
| ENSG00000255043 | ENSG00000104848 | 0.61459405 | 1.74E-15 |
| ENSG00000255087 | ENSG00000154478 | 0.702350526 | 1.61E-21 |
| ENSG00000255087 | ENSG00000107758 | 0.614370513 | 1.79E-15 |
| ENSG00000255087 | ENSG00000100884 | 0.631068013 | 1.79E-16 |
| ENSG00000255087 | ENSG00000153933 | 0.532589032 | 2.49E-11 |
| ENSG00000255087 | ENSG00000175352 | 0.614904737 | 1.67E-15 |
| ENSG00000255108 | ENSG00000177666 | 0.574824406 | 2.51E-13 |
| ENSG00000255136 | ENSG00000117016 | 0.515106702 | 1.40E-10 |
| ENSG00000255136 | ENSG00000065325 | 0.627192957 | 3.10E-16 |
| ENSG00000255136 | ENSG00000140600 | 0.515487288 | 1.35E-10 |
| ENSG00000255136 | ENSG00000184845 | 0.535814863 | 1.79E-11 |
| ENSG00000255136 | ENSG00000065559 | 0.504626059 | 3.75E-10 |
| ENSG00000255455 | ENSG00000118689 | 0.549957278 | 4.07E-12 |
| ENSG00000255455 | ENSG00000139718 | 0.634023833 | 1.18E-16 |
| ENSG00000255455 | ENSG00000116539 | 0.611587691 | 2.60E-15 |
| ENSG00000255455 | ENSG00000171634 | 0.708232473 | 5.28E-22 |
| ENSG00000255455 | ENSG00000115020 | 0.515834645 | 1.30E-10 |
| ENSG00000255455 | ENSG00000088367 | 0.554525619 | 2.48E-12 |
| ENSG00000255455 | ENSG00000110066 | 0.559212647 | 1.48E-12 |
| ENSG00000255455 | ENSG00000184787 | 0.594133918 | 2.45E-14 |
| ENSG00000255455 | ENSG00000111676 | 0.620610498 | 7.71E-16 |
| ENSG00000255455 | ENSG00000197323 | 0.547280777 | 5.42E-12 |
| ENSG00000255455 | ENSG00000162664 | 0.595483439 | 2.07E-14 |
| ENSG00000255455 | ENSG00000181222 | 0.539825296 | 1.19E-11 |
| ENSG00000255455 | ENSG00000005810 | 0.514381256 | 1.50E-10 |
| ENSG00000255455 | ENSG00000257093 | 0.651669126 | 8.58E-18 |
| ENSG00000255455 | ENSG00000152102 | 0.694206626 | 7.20E-21 |
| ENSG00000255455 | ENSG00000184677 | 0.659170857 | 2.67E-18 |
| ENSG00000255455 | ENSG00000174231 | 0.594103752 | 2.46E-14 |
| ENSG00000255455 | ENSG00000197283 | 0.633842573 | 1.21E-16 |
| ENSG00000255455 | ENSG00000224470 | 0.570587727 | 4.11E-13 |
| ENSG00000255455 | ENSG00000144535 | 0.643313584 | 3.03E-17 |
| ENSG00000255455 | ENSG00000080802 | 0.514130289 | 1.53E-10 |
| ENSG00000255455 | ENSG00000117713 | 0.642667072 | 3.33E-17 |
| ENSG00000255455 | ENSG00000175727 | 0.502774556 | 4.45E-10 |
| ENSG00000255455 | ENSG00000100354 | 0.540475526 | 1.11E-11 |
| ENSG00000255455 | ENSG00000151422 | 0.563055716 | 9.67E-13 |
| ENSG00000255455 | ENSG00000175216 | 0.563163866 | 9.55E-13 |
| ENSG00000255455 | ENSG00000111011 | 0.559922859 | 1.37E-12 |
| ENSG00000255455 | ENSG00000166326 | 0.560793902 | 1.24E-12 |
| ENSG00000255455 | ENSG00000185818 | 0.566646074 | 6.45E-13 |
| ENSG00000255455 | ENSG00000163625 | 0.58464707 | 7.84E-14 |
| ENSG00000255455 | ENSG00000164190 | 0.586992597 | 5.90E-14 |
| ENSG00000255455 | ENSG00000134982 | 0.58699747 | 5.90E-14 |
| ENSG00000255455 | ENSG00000164219 | 0.524014787 | 5.88E-11 |
| ENSG00000255455 | ENSG00000112855 | 0.645119009 | 2.31E-17 |
| ENSG00000255455 | ENSG00000115568 | 0.680240268 | 8.40E-20 |
| ENSG00000255455 | ENSG00000054118 | 0.521366459 | 7.62E-11 |
| ENSG00000255455 | ENSG00000275832 | 0.577150715 | 1.91E-13 |
| ENSG00000255455 | ENSG00000204569 | 0.574027232 | 2.76E-13 |
| ENSG00000255455 | ENSG00000158545 | 0.501934707 | 4.81E-10 |
| ENSG00000255455 | ENSG00000145555 | 0.57462307 | 2.57E-13 |
| ENSG00000255455 | ENSG00000068654 | 0.57731042 | 1.88E-13 |
| ENSG00000255455 | ENSG00000133030 | 0.606191322 | 5.27E-15 |
| ENSG00000255455 | ENSG00000113141 | 0.527544308 | 4.14E-11 |
| ENSG00000255455 | ENSG00000164068 | 0.54214271 | 9.32E-12 |
| ENSG00000255455 | ENSG00000152990 | 0.547165814 | 5.48E-12 |
| ENSG00000255455 | ENSG00000166123 | 0.525426356 | 5.11E-11 |
| ENSG00000255455 | ENSG00000143776 | 0.560486367 | 1.29E-12 |
| ENSG00000255455 | ENSG00000160216 | 0.603178168 | 7.79E-15 |
| ENSG00000255455 | ENSG00000088854 | 0.588950636 | 4.64E-14 |
| ENSG00000255455 | ENSG00000164880 | 0.576971123 | 1.96E-13 |
| ENSG00000255455 | ENSG00000038358 | 0.5055468 | 3.44E-10 |
| ENSG00000255455 | ENSG00000264522 | 0.53722721 | 1.55E-11 |
| ENSG00000255455 | ENSG00000156639 | 0.503738474 | 4.07E-10 |
| ENSG00000255455 | ENSG00000065526 | 0.632016998 | 1.57E-16 |
| ENSG00000255455 | ENSG00000204304 | 0.517349376 | 1.13E-10 |
| ENSG00000255455 | ENSG00000166444 | 0.555953581 | 2.12E-12 |
| ENSG00000255455 | ENSG00000170145 | 0.523299528 | 6.31E-11 |
| ENSG00000255455 | ENSG00000086758 | 0.638978477 | 5.74E-17 |
| ENSG00000255455 | ENSG00000141068 | 0.614401832 | 1.79E-15 |
| ENSG00000255455 | ENSG00000158480 | 0.525225776 | 5.21E-11 |
| ENSG00000255455 | ENSG00000146587 | 0.632828704 | 1.40E-16 |
| ENSG00000255455 | ENSG00000137494 | 0.543458656 | 8.12E-12 |
| ENSG00000255455 | ENSG00000125686 | 0.582097162 | 1.06E-13 |
| ENSG00000255455 | ENSG00000070047 | 0.616512919 | 1.34E-15 |
| ENSG00000255455 | ENSG00000108506 | 0.542838353 | 8.66E-12 |
| ENSG00000255455 | ENSG00000197312 | 0.546964964 | 5.60E-12 |
| ENSG00000255455 | ENSG00000266173 | 0.653990732 | 6.00E-18 |
| ENSG00000255455 | ENSG00000110395 | 0.62521617 | 4.08E-16 |
| ENSG00000255455 | ENSG00000204599 | 0.570447359 | 4.17E-13 |
| ENSG00000255455 | ENSG00000144674 | 0.549195093 | 4.42E-12 |
| ENSG00000255455 | ENSG00000157014 | 0.526421669 | 4.63E-11 |
| ENSG00000255455 | ENSG00000163596 | 0.543455753 | 8.12E-12 |
| ENSG00000255455 | ENSG00000074755 | 0.690583535 | 1.38E-20 |
| ENSG00000255455 | ENSG00000178188 | 0.646975539 | 1.75E-17 |
| ENSG00000255455 | ENSG00000143126 | 0.543023106 | 8.50E-12 |
| ENSG00000255455 | ENSG00000180357 | 0.614542263 | 1.75E-15 |
| ENSG00000255455 | ENSG00000106608 | 0.513360982 | 1.65E-10 |
| ENSG00000255455 | ENSG00000011021 | 0.595068445 | 2.18E-14 |
| ENSG00000255455 | ENSG00000198730 | 0.50413337 | 3.92E-10 |
| ENSG00000255455 | ENSG00000120948 | 0.586846104 | 6.00E-14 |
| ENSG00000255455 | ENSG00000128191 | 0.557614587 | 1.77E-12 |
| ENSG00000255455 | ENSG00000273559 | 0.546883187 | 5.65E-12 |
| ENSG00000255455 | ENSG00000059145 | 0.739512076 | 8.56E-25 |
| ENSG00000255455 | ENSG00000078549 | 0.511359087 | 2.00E-10 |
| ENSG00000255455 | ENSG00000168488 | 0.521733693 | 7.35E-11 |
| ENSG00000255455 | ENSG00000132604 | 0.505500105 | 3.46E-10 |
| ENSG00000255455 | ENSG00000248333 | 0.599590248 | 1.23E-14 |
| ENSG00000255455 | ENSG00000173821 | 0.518060329 | 1.05E-10 |
| ENSG00000255455 | ENSG00000204842 | 0.676305984 | 1.64E-19 |
| ENSG00000255455 | ENSG00000126705 | 0.56730492 | 5.98E-13 |
| ENSG00000255455 | ENSG00000064999 | 0.568912387 | 4.98E-13 |
| ENSG00000255455 | ENSG00000172375 | 0.589659327 | 4.26E-14 |
| ENSG00000255455 | ENSG00000160633 | 0.589489344 | 4.35E-14 |
| ENSG00000255455 | ENSG00000023171 | 0.564222135 | 8.48E-13 |
| ENSG00000255455 | ENSG00000116698 | 0.616305049 | 1.38E-15 |
| ENSG00000255455 | ENSG00000172057 | 0.533839515 | 2.20E-11 |
| ENSG00000255455 | ENSG00000175115 | 0.607624586 | 4.38E-15 |
| ENSG00000255455 | ENSG00000147133 | 0.599029497 | 1.32E-14 |
| ENSG00000255455 | ENSG00000121671 | 0.623467231 | 5.20E-16 |
| ENSG00000255455 | ENSG00000108468 | 0.508459623 | 2.62E-10 |
| ENSG00000255455 | ENSG00000115053 | 0.513447104 | 1.64E-10 |
| ENSG00000255455 | ENSG00000123066 | 0.620207494 | 8.15E-16 |
| ENSG00000255455 | ENSG00000114867 | 0.514414091 | 1.49E-10 |
| ENSG00000255455 | ENSG00000141564 | 0.565086012 | 7.69E-13 |
| ENSG00000255455 | ENSG00000085982 | 0.656227685 | 4.24E-18 |
| ENSG00000255455 | ENSG00000010322 | 0.582920136 | 9.65E-14 |
| ENSG00000255455 | ENSG00000270647 | 0.676880341 | 1.49E-19 |
| ENSG00000255455 | ENSG00000167258 | 0.541650863 | 9.81E-12 |
| ENSG00000255455 | ENSG00000160305 | 0.612186012 | 2.40E-15 |
| ENSG00000255455 | ENSG00000167986 | 0.502493953 | 4.56E-10 |
| ENSG00000255455 | ENSG00000110344 | 0.50955603 | 2.37E-10 |
| ENSG00000255455 | ENSG00000185129 | 0.5102253 | 2.22E-10 |
| ENSG00000255455 | ENSG00000172534 | 0.609992297 | 3.21E-15 |
| ENSG00000255455 | ENSG00000108352 | 0.56637872 | 6.64E-13 |
| ENSG00000255455 | ENSG00000084676 | 0.632255881 | 1.51E-16 |
| ENSG00000255455 | ENSG00000215421 | 0.547427861 | 5.33E-12 |
| ENSG00000255455 | ENSG00000078687 | 0.730607019 | 5.84E-24 |
| ENSG00000255455 | ENSG00000140632 | 0.512727139 | 1.75E-10 |
| ENSG00000255455 | ENSG00000112787 | 0.518285713 | 1.03E-10 |
| ENSG00000255455 | ENSG00000084112 | 0.527689084 | 4.08E-11 |
| ENSG00000255455 | ENSG00000172379 | 0.569738536 | 4.53E-13 |
| ENSG00000255455 | ENSG00000197386 | 0.633827879 | 1.21E-16 |
| ENSG00000255455 | ENSG00000133606 | 0.53025574 | 3.16E-11 |
| ENSG00000255455 | ENSG00000197226 | 0.600346076 | 1.12E-14 |
| ENSG00000255455 | ENSG00000108510 | 0.518280927 | 1.03E-10 |
| ENSG00000255455 | ENSG00000126746 | 0.533629287 | 2.24E-11 |
| ENSG00000255455 | ENSG00000066933 | 0.530778148 | 2.99E-11 |
| ENSG00000255455 | ENSG00000136828 | 0.572706275 | 3.22E-13 |
| ENSG00000255455 | ENSG00000139437 | 0.74265093 | 4.27E-25 |
| ENSG00000255455 | ENSG00000103657 | 0.612775377 | 2.22E-15 |
| ENSG00000255455 | ENSG00000187079 | 0.59188475 | 3.24E-14 |
| ENSG00000255455 | ENSG00000005339 | 0.567049259 | 6.16E-13 |
| ENSG00000255455 | ENSG00000113360 | 0.685814713 | 3.20E-20 |
| ENSG00000255455 | ENSG00000170921 | 0.639606496 | 5.23E-17 |
| ENSG00000255455 | ENSG00000134324 | 0.530398101 | 3.11E-11 |
| ENSG00000255455 | ENSG00000038532 | 0.590051762 | 4.06E-14 |
| ENSG00000255455 | ENSG00000108424 | 0.537183434 | 1.56E-11 |
| ENSG00000255455 | ENSG00000187605 | 0.581201057 | 1.18E-13 |
| ENSG00000255455 | ENSG00000135090 | 0.561500561 | 1.15E-12 |
| ENSG00000255455 | ENSG00000160294 | 0.548855467 | 4.58E-12 |
| ENSG00000255455 | ENSG00000100580 | 0.521586546 | 7.46E-11 |
| ENSG00000255455 | ENSG00000075292 | 0.652055 | 8.09E-18 |
| ENSG00000255455 | ENSG00000145882 | 0.510456063 | 2.17E-10 |
| ENSG00000255455 | ENSG00000089280 | 0.563063272 | 9.66E-13 |
| ENSG00000255455 | ENSG00000087274 | 0.565093075 | 7.68E-13 |
| ENSG00000255455 | ENSG00000135924 | 0.53721106 | 1.55E-11 |
| ENSG00000255455 | ENSG00000149187 | 0.706168748 | 7.83E-22 |
| ENSG00000255455 | ENSG00000160710 | 0.502408242 | 4.60E-10 |
| ENSG00000255455 | ENSG00000175220 | 0.581663805 | 1.12E-13 |
| ENSG00000255455 | ENSG00000141503 | 0.555367917 | 2.26E-12 |
| ENSG00000255455 | ENSG00000114650 | 0.511123614 | 2.04E-10 |
| ENSG00000255455 | ENSG00000274211 | 0.697909308 | 3.66E-21 |
| ENSG00000255455 | ENSG00000151502 | 0.646621408 | 1.85E-17 |
| ENSG00000255455 | ENSG00000118900 | 0.533341059 | 2.31E-11 |
| ENSG00000255455 | ENSG00000146826 | 0.506836625 | 3.05E-10 |
| ENSG00000255455 | ENSG00000119402 | 0.511650644 | 1.94E-10 |
| ENSG00000255455 | ENSG00000182095 | 0.585712621 | 6.89E-14 |
| ENSG00000255455 | ENSG00000135503 | 0.508200732 | 2.69E-10 |
| ENSG00000255455 | ENSG00000198863 | 0.577056789 | 1.94E-13 |
| ENSG00000255455 | ENSG00000061936 | 0.642565074 | 3.38E-17 |
| ENSG00000255455 | ENSG00000187742 | 0.587724086 | 5.40E-14 |
| ENSG00000255455 | ENSG00000196914 | 0.649668115 | 1.16E-17 |
| ENSG00000255455 | ENSG00000076108 | 0.573976569 | 2.78E-13 |
| ENSG00000255455 | ENSG00000133065 | 0.630206441 | 2.03E-16 |
| ENSG00000255455 | ENSG00000183826 | 0.670387987 | 4.39E-19 |
| ENSG00000255455 | ENSG00000075856 | 0.554645988 | 2.45E-12 |
| ENSG00000255455 | ENSG00000082641 | 0.51120061 | 2.03E-10 |
| ENSG00000255455 | ENSG00000138668 | 0.514674965 | 1.46E-10 |
| ENSG00000255455 | ENSG00000142687 | 0.544121074 | 7.57E-12 |
| ENSG00000255455 | ENSG00000087365 | 0.569986826 | 4.40E-13 |
| ENSG00000255455 | ENSG00000067248 | 0.550742718 | 3.74E-12 |
| ENSG00000255455 | ENSG00000197892 | 0.513735816 | 1.59E-10 |
| ENSG00000255455 | ENSG00000123064 | 0.536436765 | 1.68E-11 |
| ENSG00000255455 | ENSG00000196547 | 0.538525079 | 1.36E-11 |
| ENSG00000255455 | ENSG00000164576 | 0.534095517 | 2.14E-11 |
| ENSG00000255455 | ENSG00000008083 | 0.504960782 | 3.63E-10 |
| ENSG00000255455 | ENSG00000109756 | 0.624875064 | 4.28E-16 |
| ENSG00000255455 | ENSG00000169992 | 0.588762153 | 4.75E-14 |
| ENSG00000255455 | ENSG00000173064 | 0.673186526 | 2.76E-19 |
| ENSG00000255455 | ENSG00000133812 | 0.577549121 | 1.83E-13 |
| ENSG00000255455 | ENSG00000143376 | 0.514357026 | 1.50E-10 |
| ENSG00000255455 | ENSG00000122515 | 0.609619791 | 3.37E-15 |
| ENSG00000255455 | ENSG00000140691 | 0.552238595 | 3.18E-12 |
| ENSG00000255794 | ENSG00000164953 | 0.501005233 | 5.23E-10 |
| ENSG00000255980 | ENSG00000169252 | 0.696949537 | 4.37E-21 |
| ENSG00000255980 | ENSG00000198246 | 0.624703568 | 4.39E-16 |
| ENSG00000255980 | ENSG00000140968 | 0.648865274 | 1.32E-17 |
| ENSG00000256028 | ENSG00000059145 | 0.556975512 | 1.90E-12 |
| ENSG00000256028 | ENSG00000119638 | 0.531600378 | 2.76E-11 |
| ENSG00000256028 | ENSG00000108352 | 0.51209646 | 1.86E-10 |
| ENSG00000256028 | ENSG00000204842 | 0.502224087 | 4.68E-10 |
| ENSG00000256028 | ENSG00000177082 | 0.644837774 | 2.41E-17 |
| ENSG00000256028 | ENSG00000010295 | 0.61915925 | 9.40E-16 |
| ENSG00000256028 | ENSG00000125447 | 0.578109313 | 1.71E-13 |
| ENSG00000256028 | ENSG00000160305 | 0.548067499 | 4.98E-12 |
| ENSG00000256028 | ENSG00000204304 | 0.545774246 | 6.36E-12 |
| ENSG00000256028 | ENSG00000160299 | 0.509272182 | 2.43E-10 |
| ENSG00000256028 | ENSG00000176953 | 0.537336245 | 1.53E-11 |
| ENSG00000256028 | ENSG00000135722 | 0.51797848 | 1.06E-10 |
| ENSG00000256028 | ENSG00000161328 | 0.502011232 | 4.77E-10 |
| ENSG00000256028 | ENSG00000127419 | 0.6345209 | 1.10E-16 |
| ENSG00000256028 | ENSG00000169914 | 0.565073273 | 7.70E-13 |
| ENSG00000256028 | ENSG00000159433 | 0.607841316 | 4.25E-15 |
| ENSG00000256028 | ENSG00000070610 | 0.522787231 | 6.63E-11 |
| ENSG00000256028 | ENSG00000275023 | 0.557979349 | 1.70E-12 |
| ENSG00000256028 | ENSG00000124574 | 0.565308935 | 7.50E-13 |
| ENSG00000256028 | ENSG00000139437 | 0.522289592 | 6.96E-11 |
| ENSG00000256028 | ENSG00000108963 | 0.626049907 | 3.64E-16 |
| ENSG00000256028 | ENSG00000168906 | 0.518669383 | 9.91E-11 |
| ENSG00000256028 | ENSG00000126870 | 0.587646341 | 5.45E-14 |
| ENSG00000256028 | ENSG00000133226 | 0.608796281 | 3.75E-15 |
| ENSG00000256028 | ENSG00000135912 | 0.515779942 | 1.31E-10 |
| ENSG00000256028 | ENSG00000160460 | 0.515191884 | 1.39E-10 |
| ENSG00000256028 | ENSG00000266173 | 0.566431556 | 6.60E-13 |
| ENSG00000256028 | ENSG00000197558 | 0.605576134 | 5.71E-15 |
| ENSG00000256028 | ENSG00000177169 | 0.524851128 | 5.41E-11 |
| ENSG00000256028 | ENSG00000061936 | 0.570767857 | 4.02E-13 |
| ENSG00000256028 | ENSG00000164828 | 0.574013346 | 2.76E-13 |
| ENSG00000256028 | ENSG00000254997 | 0.52496758 | 5.35E-11 |
| ENSG00000256028 | ENSG00000146826 | 0.576065707 | 2.17E-13 |
| ENSG00000256028 | ENSG00000184787 | 0.647024716 | 1.74E-17 |
| ENSG00000256028 | ENSG00000183495 | 0.538533809 | 1.36E-11 |
| ENSG00000256028 | ENSG00000074755 | 0.58401377 | 8.46E-14 |
| ENSG00000256028 | ENSG00000214655 | 0.606049808 | 5.37E-15 |
| ENSG00000256085 | ENSG00000185002 | 0.563241233 | 9.47E-13 |
| ENSG00000256193 | ENSG00000158856 | 0.591840582 | 3.25E-14 |
| ENSG00000256193 | ENSG00000181418 | 0.698240111 | 3.45E-21 |
| ENSG00000256193 | ENSG00000136854 | 0.579156105 | 1.51E-13 |
| ENSG00000256193 | ENSG00000065559 | 0.503035295 | 4.34E-10 |
| ENSG00000256193 | ENSG00000140600 | 0.524234371 | 5.75E-11 |
| ENSG00000256193 | ENSG00000166501 | 0.573023678 | 3.10E-13 |
| ENSG00000256193 | ENSG00000183780 | 0.633614714 | 1.25E-16 |
| ENSG00000256193 | ENSG00000120053 | 0.650233134 | 1.07E-17 |
| ENSG00000256193 | ENSG00000156076 | 0.599738402 | 1.21E-14 |
| ENSG00000256193 | ENSG00000156642 | 0.508069066 | 2.72E-10 |
| ENSG00000256193 | ENSG00000172350 | 0.565789152 | 7.10E-13 |
| ENSG00000256193 | ENSG00000128656 | 0.654390706 | 5.64E-18 |
| ENSG00000256193 | ENSG00000060140 | 0.796342514 | 4.73E-31 |
| ENSG00000256193 | ENSG00000125814 | 0.760752597 | 6.29E-27 |
| ENSG00000256193 | ENSG00000123901 | 0.843498996 | 5.51E-38 |
| ENSG00000256193 | ENSG00000147676 | 0.833329716 | 2.61E-36 |
| ENSG00000256193 | ENSG00000107758 | 0.601349932 | 9.84E-15 |
| ENSG00000256193 | ENSG00000164061 | 0.613816572 | 1.93E-15 |
| ENSG00000256193 | ENSG00000135750 | 0.658759839 | 2.85E-18 |
| ENSG00000256193 | ENSG00000100285 | 0.675833851 | 1.77E-19 |
| ENSG00000256193 | ENSG00000069424 | 0.617962309 | 1.11E-15 |
| ENSG00000256193 | ENSG00000123612 | 0.651353279 | 9.01E-18 |
| ENSG00000256193 | ENSG00000137843 | 0.590642822 | 3.77E-14 |
| ENSG00000256193 | ENSG00000171517 | 0.602750253 | 8.23E-15 |
| ENSG00000256193 | ENSG00000165983 | 0.760611876 | 6.51E-27 |
| ENSG00000256193 | ENSG00000154478 | 0.774520849 | 1.96E-28 |
| ENSG00000256193 | ENSG00000153933 | 0.586056461 | 6.61E-14 |
| ENSG00000256193 | ENSG00000197106 | 0.78624327 | 8.41E-30 |
| ENSG00000256193 | ENSG00000198825 | 0.501032134 | 5.22E-10 |
| ENSG00000256193 | ENSG00000169884 | 0.782757171 | 2.19E-29 |
| ENSG00000256193 | ENSG00000177570 | 0.541459519 | 1.00E-11 |
| ENSG00000256193 | ENSG00000260001 | 0.636563424 | 8.15E-17 |
| ENSG00000256193 | ENSG00000164506 | 0.53891043 | 1.30E-11 |
| ENSG00000256193 | ENSG00000119946 | 0.623652483 | 5.07E-16 |
| ENSG00000256894 | ENSG00000225190 | 0.505985492 | 3.30E-10 |
| ENSG00000256894 | ENSG00000083799 | 0.630263635 | 2.01E-16 |
| ENSG00000256894 | ENSG00000154222 | 0.518059261 | 1.05E-10 |
| ENSG00000256894 | ENSG00000121281 | 0.566086087 | 6.87E-13 |
| ENSG00000256894 | ENSG00000136478 | 0.52932483 | 3.47E-11 |
| ENSG00000256894 | ENSG00000147526 | 0.561037752 | 1.21E-12 |
| ENSG00000256894 | ENSG00000164023 | 0.520006901 | 8.70E-11 |
| ENSG00000256894 | ENSG00000169641 | 0.525288193 | 5.18E-11 |
| ENSG00000256894 | ENSG00000159658 | 0.54334313 | 8.22E-12 |
| ENSG00000256894 | ENSG00000152291 | 0.596899713 | 1.73E-14 |
| ENSG00000256894 | ENSG00000123094 | 0.619306733 | 9.21E-16 |
| ENSG00000256894 | ENSG00000158195 | 0.552594686 | 3.06E-12 |
| ENSG00000256894 | ENSG00000138641 | 0.52427278 | 5.73E-11 |
| ENSG00000256894 | ENSG00000152332 | 0.529288685 | 3.48E-11 |
| ENSG00000256894 | ENSG00000185477 | 0.512354018 | 1.82E-10 |
| ENSG00000256894 | ENSG00000137642 | 0.599702229 | 1.21E-14 |
| ENSG00000256894 | ENSG00000166548 | 0.539184169 | 1.27E-11 |
| ENSG00000256894 | ENSG00000188786 | 0.56012673 | 1.34E-12 |
| ENSG00000256894 | ENSG00000068305 | 0.549721417 | 4.17E-12 |
| ENSG00000256894 | ENSG00000141503 | 0.51067127 | 2.13E-10 |
| ENSG00000257337 | ENSG00000119280 | 0.549071811 | 4.48E-12 |
| ENSG00000257524 | ENSG00000163516 | 0.757303178 | 1.45E-26 |
| ENSG00000257524 | ENSG00000204344 | 0.540318046 | 1.13E-11 |
| ENSG00000257524 | ENSG00000239704 | 0.586514671 | 6.25E-14 |
| ENSG00000257524 | ENSG00000169885 | 0.553906028 | 2.66E-12 |
| ENSG00000257524 | ENSG00000179335 | 0.552406494 | 3.13E-12 |
| ENSG00000257524 | ENSG00000112659 | 0.561059615 | 1.21E-12 |
| ENSG00000257524 | ENSG00000197530 | 0.591531739 | 3.38E-14 |
| ENSG00000257524 | ENSG00000075826 | 0.604943091 | 6.20E-15 |
| ENSG00000257524 | ENSG00000163945 | 0.63887995 | 5.82E-17 |
| ENSG00000257524 | ENSG00000180902 | 0.560865885 | 1.23E-12 |
| ENSG00000257524 | ENSG00000133226 | 0.548443475 | 4.79E-12 |
| ENSG00000257524 | ENSG00000213983 | 0.681524128 | 6.74E-20 |
| ENSG00000257524 | ENSG00000109758 | 0.550545243 | 3.82E-12 |
| ENSG00000258301 | ENSG00000108352 | 0.537264801 | 1.55E-11 |
| ENSG00000258301 | ENSG00000013523 | 0.748676298 | 1.09E-25 |
| ENSG00000258301 | ENSG00000134698 | 0.527164854 | 4.30E-11 |
| ENSG00000258301 | ENSG00000152520 | 0.522120093 | 7.08E-11 |
| ENSG00000258301 | ENSG00000162664 | 0.548737114 | 4.64E-12 |
| ENSG00000258301 | ENSG00000100201 | 0.508759961 | 2.55E-10 |
| ENSG00000258634 | ENSG00000066135 | 0.558644163 | 1.58E-12 |
| ENSG00000258634 | ENSG00000141503 | 0.50218267 | 4.70E-10 |
| ENSG00000258634 | ENSG00000187742 | 0.617024847 | 1.26E-15 |
| ENSG00000258634 | ENSG00000149294 | 0.520657153 | 8.17E-11 |
| ENSG00000258634 | ENSG00000151276 | 0.559988635 | 1.36E-12 |
| ENSG00000258634 | ENSG00000025293 | 0.501969947 | 4.79E-10 |
| ENSG00000258634 | ENSG00000137776 | 0.648855126 | 1.32E-17 |
| ENSG00000258634 | ENSG00000135829 | 0.551491108 | 3.45E-12 |
| ENSG00000258634 | ENSG00000005339 | 0.713200355 | 2.01E-22 |
| ENSG00000258634 | ENSG00000164880 | 0.632343528 | 1.50E-16 |
| ENSG00000258634 | ENSG00000115977 | 0.550160183 | 3.98E-12 |
| ENSG00000258634 | ENSG00000105662 | 0.554326361 | 2.54E-12 |
| ENSG00000258634 | ENSG00000197283 | 0.670007501 | 4.67E-19 |
| ENSG00000258634 | ENSG00000070614 | 0.534043428 | 2.15E-11 |
| ENSG00000258634 | ENSG00000076108 | 0.689672358 | 1.62E-20 |
| ENSG00000258634 | ENSG00000132361 | 0.589897397 | 4.14E-14 |
| ENSG00000258634 | ENSG00000114867 | 0.506267641 | 3.22E-10 |
| ENSG00000258634 | ENSG00000115839 | 0.53934177 | 1.25E-11 |
| ENSG00000258634 | ENSG00000164190 | 0.68827415 | 2.08E-20 |
| ENSG00000258634 | ENSG00000196914 | 0.514069203 | 1.54E-10 |
| ENSG00000258634 | ENSG00000204304 | 0.667662143 | 6.86E-19 |
| ENSG00000258634 | ENSG00000130338 | 0.639986951 | 4.95E-17 |
| ENSG00000258634 | ENSG00000164828 | 0.51535889 | 1.36E-10 |
| ENSG00000258634 | ENSG00000008083 | 0.69830525 | 3.41E-21 |
| ENSG00000258634 | ENSG00000133065 | 0.572008829 | 3.49E-13 |
| ENSG00000258634 | ENSG00000055917 | 0.602637336 | 8.35E-15 |
| ENSG00000258634 | ENSG00000163795 | 0.604094246 | 6.92E-15 |
| ENSG00000258634 | ENSG00000132740 | 0.656414548 | 4.12E-18 |
| ENSG00000258634 | ENSG00000173064 | 0.676041717 | 1.71E-19 |
| ENSG00000258634 | ENSG00000181222 | 0.597546666 | 1.60E-14 |
| ENSG00000258634 | ENSG00000171634 | 0.766279445 | 1.61E-27 |
| ENSG00000258634 | ENSG00000160305 | 0.750074848 | 7.91E-26 |
| ENSG00000258634 | ENSG00000014164 | 0.592247961 | 3.09E-14 |
| ENSG00000258634 | ENSG00000124574 | 0.55282214 | 2.99E-12 |
| ENSG00000258634 | ENSG00000140948 | 0.711627338 | 2.74E-22 |
| ENSG00000258634 | ENSG00000134698 | 0.64549385 | 2.19E-17 |
| ENSG00000258634 | ENSG00000112787 | 0.702653875 | 1.52E-21 |
| ENSG00000258634 | ENSG00000131018 | 0.527442492 | 4.18E-11 |
| ENSG00000258634 | ENSG00000112685 | 0.561192837 | 1.19E-12 |
| ENSG00000258634 | ENSG00000074964 | 0.571191203 | 3.83E-13 |
| ENSG00000258634 | ENSG00000089280 | 0.729238367 | 7.79E-24 |
| ENSG00000258634 | ENSG00000140320 | 0.583131785 | 9.41E-14 |
| ENSG00000258634 | ENSG00000170004 | 0.662293799 | 1.63E-18 |
| ENSG00000258634 | ENSG00000170265 | 0.57248839 | 3.30E-13 |
| ENSG00000258634 | ENSG00000169925 | 0.644017452 | 2.73E-17 |
| ENSG00000258634 | ENSG00000130254 | 0.670983822 | 3.98E-19 |
| ENSG00000258634 | ENSG00000196535 | 0.536170134 | 1.73E-11 |
| ENSG00000258634 | ENSG00000158711 | 0.615253627 | 1.59E-15 |
| ENSG00000258634 | ENSG00000204843 | 0.504445104 | 3.81E-10 |
| ENSG00000258634 | ENSG00000162461 | 0.523911826 | 5.94E-11 |
| ENSG00000258634 | ENSG00000108510 | 0.542996354 | 8.52E-12 |
| ENSG00000258634 | ENSG00000140332 | 0.595424885 | 2.08E-14 |
| ENSG00000258634 | ENSG00000204569 | 0.662573721 | 1.56E-18 |
| ENSG00000258634 | ENSG00000187079 | 0.508648919 | 2.58E-10 |
| ENSG00000258634 | ENSG00000149187 | 0.678803599 | 1.07E-19 |
| ENSG00000258634 | ENSG00000163516 | 0.649916646 | 1.12E-17 |
| ENSG00000258634 | ENSG00000144535 | 0.647895368 | 1.52E-17 |
| ENSG00000258634 | ENSG00000119638 | 0.606480946 | 5.08E-15 |
| ENSG00000258634 | ENSG00000196498 | 0.607108955 | 4.68E-15 |
| ENSG00000258634 | ENSG00000068654 | 0.690517516 | 1.40E-20 |
| ENSG00000258634 | ENSG00000168872 | 0.505467925 | 3.47E-10 |
| ENSG00000258634 | ENSG00000187605 | 0.686885225 | 2.65E-20 |
| ENSG00000258634 | ENSG00000115109 | 0.546876975 | 5.66E-12 |
| ENSG00000258634 | ENSG00000164068 | 0.607665332 | 4.35E-15 |
| ENSG00000258634 | ENSG00000157540 | 0.59516167 | 2.15E-14 |
| ENSG00000258634 | ENSG00000158941 | 0.631585934 | 1.67E-16 |
| ENSG00000258634 | ENSG00000108262 | 0.619108214 | 9.46E-16 |
| ENSG00000258634 | ENSG00000196233 | 0.5285495 | 3.75E-11 |
| ENSG00000258634 | ENSG00000204574 | 0.576483804 | 2.07E-13 |
| ENSG00000258634 | ENSG00000158669 | 0.546058278 | 6.17E-12 |
| ENSG00000258634 | ENSG00000064999 | 0.672164272 | 3.27E-19 |
| ENSG00000258634 | ENSG00000152223 | 0.591043906 | 3.59E-14 |
| ENSG00000258634 | ENSG00000083168 | 0.659141732 | 2.69E-18 |
| ENSG00000258634 | ENSG00000156030 | 0.58574738 | 6.86E-14 |
| ENSG00000258634 | ENSG00000108306 | 0.642219065 | 3.56E-17 |
| ENSG00000258634 | ENSG00000111641 | 0.554473653 | 2.50E-12 |
| ENSG00000258634 | ENSG00000138668 | 0.585661576 | 6.93E-14 |
| ENSG00000258634 | ENSG00000204256 | 0.578451441 | 1.64E-13 |
| ENSG00000258634 | ENSG00000126870 | 0.641941355 | 3.71E-17 |
| ENSG00000258634 | ENSG00000214655 | 0.695184673 | 6.03E-21 |
| ENSG00000258634 | ENSG00000074755 | 0.700931415 | 2.10E-21 |
| ENSG00000258634 | ENSG00000187555 | 0.604115701 | 6.90E-15 |
| ENSG00000258634 | ENSG00000166783 | 0.602549569 | 8.44E-15 |
| ENSG00000258634 | ENSG00000123064 | 0.523155739 | 6.40E-11 |
| ENSG00000258634 | ENSG00000148843 | 0.620448687 | 7.88E-16 |
| ENSG00000258634 | ENSG00000143776 | 0.572382712 | 3.34E-13 |
| ENSG00000258634 | ENSG00000125447 | 0.618550768 | 1.02E-15 |
| ENSG00000258634 | ENSG00000108819 | 0.610808724 | 2.88E-15 |
| ENSG00000258634 | ENSG00000179981 | 0.507588037 | 2.85E-10 |
| ENSG00000258634 | ENSG00000075292 | 0.781769074 | 2.86E-29 |
| ENSG00000258634 | ENSG00000100580 | 0.572202426 | 3.41E-13 |
| ENSG00000258634 | ENSG00000077044 | 0.641551794 | 3.93E-17 |
| ENSG00000258634 | ENSG00000197562 | 0.503810209 | 4.04E-10 |
| ENSG00000258634 | ENSG00000266028 | 0.56862396 | 5.15E-13 |
| ENSG00000258634 | ENSG00000124198 | 0.501670378 | 4.92E-10 |
| ENSG00000258634 | ENSG00000166135 | 0.57960849 | 1.43E-13 |
| ENSG00000258634 | ENSG00000170653 | 0.613756631 | 1.95E-15 |
| ENSG00000258634 | ENSG00000025800 | 0.553186621 | 2.87E-12 |
| ENSG00000258634 | ENSG00000170456 | 0.521015905 | 7.89E-11 |
| ENSG00000258634 | ENSG00000169375 | 0.559380379 | 1.46E-12 |
| ENSG00000258634 | ENSG00000100354 | 0.665400991 | 9.90E-19 |
| ENSG00000258634 | ENSG00000183955 | 0.541316374 | 1.02E-11 |
| ENSG00000258634 | ENSG00000141569 | 0.503486406 | 4.17E-10 |
| ENSG00000258634 | ENSG00000084676 | 0.603343875 | 7.62E-15 |
| ENSG00000258634 | ENSG00000170921 | 0.631068788 | 1.79E-16 |
| ENSG00000258634 | ENSG00000110497 | 0.599160512 | 1.30E-14 |
| ENSG00000258634 | ENSG00000184787 | 0.692974191 | 8.99E-21 |
| ENSG00000258634 | ENSG00000159692 | 0.545957712 | 6.24E-12 |
| ENSG00000258634 | ENSG00000124535 | 0.574275054 | 2.68E-13 |
| ENSG00000258634 | ENSG00000136828 | 0.526453947 | 4.62E-11 |
| ENSG00000258634 | ENSG00000141027 | 0.580272818 | 1.32E-13 |
| ENSG00000258634 | ENSG00000160094 | 0.681485953 | 6.79E-20 |
| ENSG00000258634 | ENSG00000275832 | 0.539143708 | 1.27E-11 |
| ENSG00000258634 | ENSG00000112365 | 0.562043816 | 1.08E-12 |
| ENSG00000258634 | ENSG00000143624 | 0.674293944 | 2.30E-19 |
| ENSG00000258634 | ENSG00000121671 | 0.581348738 | 1.16E-13 |
| ENSG00000258634 | ENSG00000183495 | 0.758696773 | 1.03E-26 |
| ENSG00000258634 | ENSG00000204463 | 0.544059697 | 7.62E-12 |
| ENSG00000258634 | ENSG00000116698 | 0.699338729 | 2.82E-21 |
| ENSG00000258634 | ENSG00000126705 | 0.651427109 | 8.91E-18 |
| ENSG00000258634 | ENSG00000174231 | 0.666440397 | 8.36E-19 |
| ENSG00000258634 | ENSG00000104517 | 0.604275307 | 6.76E-15 |
| ENSG00000258634 | ENSG00000125686 | 0.687569425 | 2.35E-20 |
| ENSG00000258634 | ENSG00000132953 | 0.555916076 | 2.13E-12 |
| ENSG00000258634 | ENSG00000139668 | 0.531934948 | 2.66E-11 |
| ENSG00000258634 | ENSG00000126746 | 0.581620714 | 1.13E-13 |
| ENSG00000258634 | ENSG00000146587 | 0.640927723 | 4.31E-17 |
| ENSG00000258634 | ENSG00000077157 | 0.544912136 | 6.97E-12 |
| ENSG00000258634 | ENSG00000176915 | 0.560147703 | 1.34E-12 |
| ENSG00000258634 | ENSG00000168488 | 0.713898256 | 1.76E-22 |
| ENSG00000258634 | ENSG00000087365 | 0.607801937 | 4.28E-15 |
| ENSG00000258634 | ENSG00000134313 | 0.670909767 | 4.03E-19 |
| ENSG00000258634 | ENSG00000059145 | 0.638574012 | 6.09E-17 |
| ENSG00000258634 | ENSG00000130703 | 0.503921646 | 4.00E-10 |
| ENSG00000258634 | ENSG00000180667 | 0.507018574 | 3.00E-10 |
| ENSG00000258634 | ENSG00000186635 | 0.502830295 | 4.43E-10 |
| ENSG00000258634 | ENSG00000008869 | 0.727835449 | 1.05E-23 |
| ENSG00000258634 | ENSG00000197323 | 0.709025728 | 4.53E-22 |
| ENSG00000258634 | ENSG00000116809 | 0.580054862 | 1.36E-13 |
| ENSG00000258634 | ENSG00000160299 | 0.705080757 | 9.62E-22 |
| ENSG00000258634 | ENSG00000273559 | 0.658101767 | 3.16E-18 |
| ENSG00000258634 | ENSG00000248333 | 0.706656418 | 7.13E-22 |
| ENSG00000258634 | ENSG00000169180 | 0.58012862 | 1.35E-13 |
| ENSG00000258634 | ENSG00000163939 | 0.585436021 | 7.12E-14 |
| ENSG00000258634 | ENSG00000065526 | 0.712854877 | 2.16E-22 |
| ENSG00000258634 | ENSG00000197386 | 0.66999016 | 4.69E-19 |
| ENSG00000258634 | ENSG00000122299 | 0.533196912 | 2.34E-11 |
| ENSG00000258634 | ENSG00000106086 | 0.546467943 | 5.91E-12 |
| ENSG00000258634 | ENSG00000234127 | 0.596673099 | 1.78E-14 |
| ENSG00000258634 | ENSG00000130939 | 0.559794509 | 1.39E-12 |
| ENSG00000258634 | ENSG00000136715 | 0.680641605 | 7.84E-20 |
| ENSG00000258634 | ENSG00000153201 | 0.588460646 | 4.93E-14 |
| ENSG00000258634 | ENSG00000153317 | 0.522325584 | 6.94E-11 |
| ENSG00000258634 | ENSG00000148337 | 0.643776753 | 2.83E-17 |
| ENSG00000258634 | ENSG00000002822 | 0.530893604 | 2.96E-11 |
| ENSG00000258634 | ENSG00000110395 | 0.568508368 | 5.21E-13 |
| ENSG00000258634 | ENSG00000139613 | 0.506225901 | 3.23E-10 |
| ENSG00000258634 | ENSG00000166233 | 0.516472963 | 1.23E-10 |
| ENSG00000258634 | ENSG00000117713 | 0.772828932 | 3.05E-28 |
| ENSG00000258634 | ENSG00000175727 | 0.547486594 | 5.30E-12 |
| ENSG00000258634 | ENSG00000185722 | 0.520456985 | 8.33E-11 |
| ENSG00000258634 | ENSG00000038358 | 0.63513364 | 1.00E-16 |
| ENSG00000258634 | ENSG00000160710 | 0.629394328 | 2.27E-16 |
| ENSG00000258634 | ENSG00000108963 | 0.557242294 | 1.84E-12 |
| ENSG00000258634 | ENSG00000157933 | 0.590580402 | 3.80E-14 |
| ENSG00000258634 | ENSG00000075856 | 0.670709175 | 4.16E-19 |
| ENSG00000258634 | ENSG00000183337 | 0.611047303 | 2.79E-15 |
| ENSG00000258634 | ENSG00000112584 | 0.573021723 | 3.10E-13 |
| ENSG00000258634 | ENSG00000100813 | 0.70755952 | 6.00E-22 |
| ENSG00000258634 | ENSG00000118900 | 0.619044608 | 9.54E-16 |
| ENSG00000258634 | ENSG00000175216 | 0.551504642 | 3.45E-12 |
| ENSG00000258634 | ENSG00000224470 | 0.668085494 | 6.40E-19 |
| ENSG00000258634 | ENSG00000116539 | 0.66110664 | 1.97E-18 |
| ENSG00000258634 | ENSG00000108468 | 0.604899854 | 6.24E-15 |
| ENSG00000258634 | ENSG00000166860 | 0.667736628 | 6.78E-19 |
| ENSG00000258634 | ENSG00000159433 | 0.702412288 | 1.59E-21 |
| ENSG00000258634 | ENSG00000158545 | 0.689221358 | 1.76E-20 |
| ENSG00000258634 | ENSG00000065060 | 0.553016108 | 2.93E-12 |
| ENSG00000258634 | ENSG00000140691 | 0.539821495 | 1.19E-11 |
| ENSG00000258634 | ENSG00000005810 | 0.510416797 | 2.18E-10 |
| ENSG00000258634 | ENSG00000076641 | 0.502639343 | 4.50E-10 |
| ENSG00000258634 | ENSG00000215421 | 0.6719199 | 3.41E-19 |
| ENSG00000258634 | ENSG00000168264 | 0.594766422 | 2.26E-14 |
| ENSG00000258634 | ENSG00000139718 | 0.725134345 | 1.83E-23 |
| ENSG00000258634 | ENSG00000123066 | 0.690397571 | 1.43E-20 |
| ENSG00000258634 | ENSG00000163472 | 0.50004929 | 5.71E-10 |
| ENSG00000258634 | ENSG00000054118 | 0.608929621 | 3.69E-15 |
| ENSG00000258634 | ENSG00000103249 | 0.632588836 | 1.44E-16 |
| ENSG00000258634 | ENSG00000089234 | 0.574145784 | 2.72E-13 |
| ENSG00000258634 | ENSG00000143630 | 0.590245403 | 3.96E-14 |
| ENSG00000258634 | ENSG00000180902 | 0.638546678 | 6.11E-17 |
| ENSG00000258634 | ENSG00000151461 | 0.616952721 | 1.27E-15 |
| ENSG00000258634 | ENSG00000071054 | 0.589221958 | 4.49E-14 |
| ENSG00000258634 | ENSG00000106144 | 0.562623879 | 1.01E-12 |
| ENSG00000258634 | ENSG00000172985 | 0.546289538 | 6.02E-12 |
| ENSG00000258634 | ENSG00000204842 | 0.700036309 | 2.47E-21 |
| ENSG00000258634 | ENSG00000149930 | 0.697483359 | 3.96E-21 |
| ENSG00000258634 | ENSG00000141068 | 0.671365438 | 3.74E-19 |
| ENSG00000258634 | ENSG00000179912 | 0.59697636 | 1.71E-14 |
| ENSG00000258634 | ENSG00000090686 | 0.556558754 | 1.99E-12 |
| ENSG00000258634 | ENSG00000157014 | 0.518799216 | 9.79E-11 |
| ENSG00000258634 | ENSG00000163125 | 0.742182209 | 4.74E-25 |
| ENSG00000258634 | ENSG00000162664 | 0.693334736 | 8.42E-21 |
| ENSG00000258634 | ENSG00000118689 | 0.5859523 | 6.69E-14 |
| ENSG00000258634 | ENSG00000168067 | 0.532766072 | 2.45E-11 |
| ENSG00000258634 | ENSG00000072501 | 0.57051739 | 4.14E-13 |
| ENSG00000258634 | ENSG00000147324 | 0.51782226 | 1.08E-10 |
| ENSG00000258634 | ENSG00000137337 | 0.733869487 | 2.92E-24 |
| ENSG00000258634 | ENSG00000127616 | 0.643013133 | 3.17E-17 |
| ENSG00000258634 | ENSG00000180357 | 0.686839974 | 2.68E-20 |
| ENSG00000258634 | ENSG00000175662 | 0.594901262 | 2.22E-14 |
| ENSG00000258634 | ENSG00000119402 | 0.589423083 | 4.38E-14 |
| ENSG00000258634 | ENSG00000010322 | 0.641898665 | 3.73E-17 |
| ENSG00000258634 | ENSG00000266173 | 0.609126953 | 3.59E-15 |
| ENSG00000258634 | ENSG00000122965 | 0.644331789 | 2.60E-17 |
| ENSG00000258634 | ENSG00000133226 | 0.647098326 | 1.72E-17 |
| ENSG00000258634 | ENSG00000112182 | 0.643347633 | 3.01E-17 |
| ENSG00000258634 | ENSG00000152102 | 0.738943776 | 9.70E-25 |
| ENSG00000258634 | ENSG00000155858 | 0.502490002 | 4.57E-10 |
| ENSG00000258634 | ENSG00000270647 | 0.650879266 | 9.69E-18 |
| ENSG00000258634 | ENSG00000141252 | 0.665157564 | 1.03E-18 |
| ENSG00000258634 | ENSG00000143376 | 0.59132911 | 3.47E-14 |
| ENSG00000258634 | ENSG00000124571 | 0.52333711 | 6.28E-11 |
| ENSG00000258634 | ENSG00000080345 | 0.643822296 | 2.81E-17 |
| ENSG00000258634 | ENSG00000086758 | 0.665041736 | 1.05E-18 |
| ENSG00000258634 | ENSG00000181220 | 0.502476165 | 4.57E-10 |
| ENSG00000258634 | ENSG00000066933 | 0.579359578 | 1.47E-13 |
| ENSG00000258634 | ENSG00000053254 | 0.665018908 | 1.05E-18 |
| ENSG00000258634 | ENSG00000167258 | 0.62701983 | 3.18E-16 |
| ENSG00000258634 | ENSG00000108424 | 0.59152364 | 3.38E-14 |
| ENSG00000258634 | ENSG00000170242 | 0.538499913 | 1.36E-11 |
| ENSG00000258634 | ENSG00000108557 | 0.603032394 | 7.93E-15 |
| ENSG00000258634 | ENSG00000077235 | 0.634463579 | 1.10E-16 |
| ENSG00000258634 | ENSG00000057935 | 0.60895522 | 3.68E-15 |
| ENSG00000258634 | ENSG00000274211 | 0.715450143 | 1.29E-22 |
| ENSG00000258634 | ENSG00000134250 | 0.580595822 | 1.27E-13 |
| ENSG00000258634 | ENSG00000170776 | 0.556475422 | 2.01E-12 |
| ENSG00000258634 | ENSG00000102858 | 0.535446356 | 1.86E-11 |
| ENSG00000258634 | ENSG00000061936 | 0.769475008 | 7.19E-28 |
| ENSG00000258634 | ENSG00000132604 | 0.541183235 | 1.03E-11 |
| ENSG00000258634 | ENSG00000204560 | 0.548997834 | 4.51E-12 |
| ENSG00000258634 | ENSG00000138081 | 0.574486583 | 2.62E-13 |
| ENSG00000258634 | ENSG00000110046 | 0.579133664 | 1.51E-13 |
| ENSG00000258634 | ENSG00000111676 | 0.643672385 | 2.87E-17 |
| ENSG00000258634 | ENSG00000183826 | 0.571616324 | 3.65E-13 |
| ENSG00000258634 | ENSG00000144674 | 0.546271799 | 6.03E-12 |
| ENSG00000258634 | ENSG00000139990 | 0.51281 | 1.74E-10 |
| ENSG00000258634 | ENSG00000006459 | 0.504062999 | 3.95E-10 |
| ENSG00000258634 | ENSG00000135912 | 0.530558974 | 3.06E-11 |
| ENSG00000258634 | ENSG00000184677 | 0.773797401 | 2.37E-28 |
| ENSG00000258634 | ENSG00000066739 | 0.722697432 | 3.02E-23 |
| ENSG00000258634 | ENSG00000169914 | 0.67344375 | 2.65E-19 |
| ENSG00000258634 | ENSG00000143093 | 0.69504424 | 6.18E-21 |
| ENSG00000258634 | ENSG00000069275 | 0.57537913 | 2.36E-13 |
| ENSG00000258634 | ENSG00000152990 | 0.586980487 | 5.91E-14 |
| ENSG00000258634 | ENSG00000149657 | 0.649390914 | 1.22E-17 |
| ENSG00000258634 | ENSG00000068308 | 0.529117868 | 3.54E-11 |
| ENSG00000258667 | ENSG00000113083 | 0.571314881 | 3.78E-13 |
| ENSG00000259319 | ENSG00000123612 | 0.553731429 | 2.71E-12 |
| ENSG00000259319 | ENSG00000158109 | 0.557258796 | 1.84E-12 |
| ENSG00000259319 | ENSG00000163393 | 0.538683415 | 1.34E-11 |
| ENSG00000259319 | ENSG00000111269 | 0.510731383 | 2.12E-10 |
| ENSG00000259319 | ENSG00000169252 | 0.562805742 | 9.94E-13 |
| ENSG00000259319 | ENSG00000148660 | 0.542942445 | 8.57E-12 |
| ENSG00000259330 | ENSG00000110324 | 0.558699695 | 1.57E-12 |
| ENSG00000259330 | ENSG00000177885 | 0.522405725 | 6.89E-11 |
| ENSG00000259330 | ENSG00000266094 | 0.538680082 | 1.34E-11 |
| ENSG00000259366 | ENSG00000057935 | 0.532546218 | 2.50E-11 |
| ENSG00000259366 | ENSG00000176915 | 0.503491122 | 4.16E-10 |
| ENSG00000259366 | ENSG00000185722 | 0.608471935 | 3.92E-15 |
| ENSG00000259366 | ENSG00000065613 | 0.550467367 | 3.85E-12 |
| ENSG00000259366 | ENSG00000132953 | 0.57586745 | 2.23E-13 |
| ENSG00000259366 | ENSG00000100354 | 0.686194498 | 3.00E-20 |
| ENSG00000259366 | ENSG00000274211 | 0.677677277 | 1.30E-19 |
| ENSG00000259366 | ENSG00000072736 | 0.672470474 | 3.11E-19 |
| ENSG00000259366 | ENSG00000151276 | 0.512271195 | 1.83E-10 |
| ENSG00000259366 | ENSG00000170921 | 0.660022717 | 2.34E-18 |
| ENSG00000259366 | ENSG00000170776 | 0.547578631 | 5.25E-12 |
| ENSG00000259366 | ENSG00000149187 | 0.640404313 | 4.66E-17 |
| ENSG00000259366 | ENSG00000008083 | 0.624359094 | 4.60E-16 |
| ENSG00000259366 | ENSG00000143322 | 0.521344342 | 7.64E-11 |
| ENSG00000259366 | ENSG00000165097 | 0.53603284 | 1.75E-11 |
| ENSG00000259366 | ENSG00000164164 | 0.613125667 | 2.12E-15 |
| ENSG00000259366 | ENSG00000080345 | 0.602541302 | 8.45E-15 |
| ENSG00000259366 | ENSG00000054793 | 0.58451512 | 7.96E-14 |
| ENSG00000259366 | ENSG00000142599 | 0.538327298 | 1.39E-11 |
| ENSG00000259366 | ENSG00000152767 | 0.502737215 | 4.46E-10 |
| ENSG00000259366 | ENSG00000088854 | 0.583371469 | 9.14E-14 |
| ENSG00000259366 | ENSG00000134250 | 0.65405565 | 5.94E-18 |
| ENSG00000259366 | ENSG00000167258 | 0.744999837 | 2.52E-25 |
| ENSG00000259366 | ENSG00000108506 | 0.591022251 | 3.60E-14 |
| ENSG00000259366 | ENSG00000133226 | 0.536642204 | 1.65E-11 |
| ENSG00000259366 | ENSG00000143970 | 0.652915581 | 7.09E-18 |
| ENSG00000259366 | ENSG00000114648 | 0.610593487 | 2.96E-15 |
| ENSG00000259366 | ENSG00000264522 | 0.620834214 | 7.48E-16 |
| ENSG00000259366 | ENSG00000170871 | 0.575375402 | 2.36E-13 |
| ENSG00000259366 | ENSG00000196233 | 0.56602336 | 6.92E-13 |
| ENSG00000259366 | ENSG00000076108 | 0.724572651 | 2.05E-23 |
| ENSG00000259366 | ENSG00000072364 | 0.699546208 | 2.71E-21 |
| ENSG00000259366 | ENSG00000067596 | 0.530514061 | 3.08E-11 |
| ENSG00000259366 | ENSG00000154114 | 0.565384241 | 7.44E-13 |
| ENSG00000259366 | ENSG00000169375 | 0.504102393 | 3.94E-10 |
| ENSG00000259366 | ENSG00000118007 | 0.592382948 | 3.04E-14 |
| ENSG00000259366 | ENSG00000217128 | 0.500569956 | 5.45E-10 |
| ENSG00000259366 | ENSG00000111266 | 0.512565355 | 1.78E-10 |
| ENSG00000259366 | ENSG00000138081 | 0.71141773 | 2.85E-22 |
| ENSG00000259366 | ENSG00000153201 | 0.613948545 | 1.90E-15 |
| ENSG00000259366 | ENSG00000117713 | 0.707265649 | 6.35E-22 |
| ENSG00000259366 | ENSG00000075292 | 0.630723059 | 1.88E-16 |
| ENSG00000259366 | ENSG00000140948 | 0.692272869 | 1.02E-20 |
| ENSG00000259366 | ENSG00000108819 | 0.544774244 | 7.07E-12 |
| ENSG00000259366 | ENSG00000124782 | 0.565321238 | 7.49E-13 |
| ENSG00000259366 | ENSG00000156030 | 0.636425889 | 8.32E-17 |
| ENSG00000259366 | ENSG00000005810 | 0.571027913 | 3.90E-13 |
| ENSG00000259366 | ENSG00000166860 | 0.637301216 | 7.33E-17 |
| ENSG00000259366 | ENSG00000198836 | 0.575688683 | 2.27E-13 |
| ENSG00000259366 | ENSG00000048740 | 0.503237596 | 4.26E-10 |
| ENSG00000259366 | ENSG00000266173 | 0.501419088 | 5.04E-10 |
| ENSG00000259366 | ENSG00000117036 | 0.52028372 | 8.47E-11 |
| ENSG00000259366 | ENSG00000272886 | 0.558325783 | 1.64E-12 |
| ENSG00000259366 | ENSG00000204256 | 0.507962816 | 2.75E-10 |
| ENSG00000259366 | ENSG00000068305 | 0.536917659 | 1.60E-11 |
| ENSG00000259366 | ENSG00000130779 | 0.530022015 | 3.23E-11 |
| ENSG00000259366 | ENSG00000171634 | 0.723874455 | 2.37E-23 |
| ENSG00000259366 | ENSG00000141252 | 0.630829913 | 1.86E-16 |
| ENSG00000259366 | ENSG00000186660 | 0.53685474 | 1.61E-11 |
| ENSG00000259366 | ENSG00000138246 | 0.65340138 | 6.58E-18 |
| ENSG00000259366 | ENSG00000185129 | 0.534273258 | 2.10E-11 |
| ENSG00000259366 | ENSG00000104517 | 0.693713979 | 7.87E-21 |
| ENSG00000259366 | ENSG00000157540 | 0.502056175 | 4.75E-10 |
| ENSG00000259366 | ENSG00000143376 | 0.643137088 | 3.11E-17 |
| ENSG00000259366 | ENSG00000109787 | 0.505304407 | 3.52E-10 |
| ENSG00000259366 | ENSG00000125686 | 0.679293864 | 9.88E-20 |
| ENSG00000259366 | ENSG00000152102 | 0.66401796 | 1.24E-18 |
| ENSG00000259366 | ENSG00000122299 | 0.595871004 | 1.97E-14 |
| ENSG00000259366 | ENSG00000123066 | 0.739194124 | 9.18E-25 |
| ENSG00000259366 | ENSG00000129595 | 0.507599499 | 2.84E-10 |
| ENSG00000259366 | ENSG00000140332 | 0.54004584 | 1.16E-11 |
| ENSG00000259366 | ENSG00000076513 | 0.514737226 | 1.45E-10 |
| ENSG00000259366 | ENSG00000143624 | 0.692165246 | 1.04E-20 |
| ENSG00000259366 | ENSG00000166326 | 0.555131018 | 2.32E-12 |
| ENSG00000259366 | ENSG00000157933 | 0.570236994 | 4.28E-13 |
| ENSG00000259366 | ENSG00000158195 | 0.532933686 | 2.41E-11 |
| ENSG00000259366 | ENSG00000166450 | 0.522033932 | 7.14E-11 |
| ENSG00000259366 | ENSG00000069275 | 0.518032408 | 1.05E-10 |
| ENSG00000259366 | ENSG00000177303 | 0.531493258 | 2.79E-11 |
| ENSG00000259366 | ENSG00000197081 | 0.586751192 | 6.07E-14 |
| ENSG00000259366 | ENSG00000166197 | 0.58867616 | 4.80E-14 |
| ENSG00000259366 | ENSG00000196535 | 0.650195066 | 1.08E-17 |
| ENSG00000259417 | ENSG00000128872 | 0.520085044 | 8.64E-11 |
| ENSG00000259495 | ENSG00000136448 | 0.557131484 | 1.87E-12 |
| ENSG00000259495 | ENSG00000166689 | 0.553260201 | 2.85E-12 |
| ENSG00000259495 | ENSG00000030419 | 0.575123999 | 2.43E-13 |
| ENSG00000259495 | ENSG00000185129 | 0.529425617 | 3.43E-11 |
| ENSG00000259495 | ENSG00000120451 | 0.616755675 | 1.30E-15 |
| ENSG00000259495 | ENSG00000188786 | 0.567608749 | 5.78E-13 |
| ENSG00000259495 | ENSG00000204659 | 0.547026262 | 5.57E-12 |
| ENSG00000259495 | ENSG00000166444 | 0.581506899 | 1.14E-13 |
| ENSG00000259495 | ENSG00000133065 | 0.565173517 | 7.62E-13 |
| ENSG00000259495 | ENSG00000134982 | 0.525861147 | 4.90E-11 |
| ENSG00000259495 | ENSG00000204104 | 0.550426982 | 3.87E-12 |
| ENSG00000259495 | ENSG00000184602 | 0.547050542 | 5.55E-12 |
| ENSG00000259495 | ENSG00000109501 | 0.516561798 | 1.21E-10 |
| ENSG00000259495 | ENSG00000187726 | 0.520140064 | 8.59E-11 |
| ENSG00000259495 | ENSG00000198198 | 0.504757633 | 3.70E-10 |
| ENSG00000259495 | ENSG00000185722 | 0.602128935 | 8.91E-15 |
| ENSG00000259583 | ENSG00000104722 | 0.573959052 | 2.78E-13 |
| ENSG00000259583 | ENSG00000137875 | 0.530604601 | 3.05E-11 |
| ENSG00000259762 | ENSG00000141068 | 0.523574991 | 6.14E-11 |
| ENSG00000259826 | ENSG00000084674 | 0.504442097 | 3.81E-10 |
| ENSG00000260071 | ENSG00000139537 | 0.518298891 | 1.03E-10 |
| ENSG00000260461 | ENSG00000086758 | 0.517843717 | 1.07E-10 |
| ENSG00000260461 | ENSG00000188786 | 0.513463071 | 1.63E-10 |
| ENSG00000260461 | ENSG00000225190 | 0.535955087 | 1.77E-11 |
| ENSG00000260461 | ENSG00000166135 | 0.551835491 | 3.32E-12 |
| ENSG00000260461 | ENSG00000264522 | 0.513275682 | 1.66E-10 |
| ENSG00000260461 | ENSG00000181222 | 0.503717033 | 4.08E-10 |
| ENSG00000260461 | ENSG00000153201 | 0.501438831 | 5.03E-10 |
| ENSG00000260461 | ENSG00000136448 | 0.569656749 | 4.57E-13 |
| ENSG00000260461 | ENSG00000068323 | 0.538878017 | 1.31E-11 |
| ENSG00000260461 | ENSG00000115839 | 0.565173023 | 7.62E-13 |
| ENSG00000260461 | ENSG00000151461 | 0.554729879 | 2.43E-12 |
| ENSG00000260464 | ENSG00000123612 | 0.569235145 | 4.80E-13 |
| ENSG00000260464 | ENSG00000184602 | 0.502412178 | 4.60E-10 |
| ENSG00000260630 | ENSG00000149927 | 0.514369633 | 1.50E-10 |
| ENSG00000260664 | ENSG00000170921 | 0.541036433 | 1.05E-11 |
| ENSG00000260664 | ENSG00000102606 | 0.513615403 | 1.61E-10 |
| ENSG00000260664 | ENSG00000166454 | 0.532288355 | 2.57E-11 |
| ENSG00000260664 | ENSG00000172379 | 0.533297249 | 2.32E-11 |
| ENSG00000260664 | ENSG00000151276 | 0.610129358 | 3.15E-15 |
| ENSG00000260664 | ENSG00000110066 | 0.508737645 | 2.56E-10 |
| ENSG00000260664 | ENSG00000257093 | 0.536525988 | 1.67E-11 |
| ENSG00000260664 | ENSG00000140948 | 0.558633728 | 1.58E-12 |
| ENSG00000260664 | ENSG00000109756 | 0.543235893 | 8.31E-12 |
| ENSG00000260788 | ENSG00000180354 | 0.695534069 | 5.66E-21 |
| ENSG00000260788 | ENSG00000114757 | 0.803730385 | 5.19E-32 |
| ENSG00000260788 | ENSG00000066468 | 0.56368097 | 9.01E-13 |
| ENSG00000260788 | ENSG00000213424 | 0.507344996 | 2.91E-10 |
| ENSG00000260788 | ENSG00000128645 | 0.696826416 | 4.47E-21 |
| ENSG00000260788 | ENSG00000187764 | 0.591335005 | 3.46E-14 |
| ENSG00000260788 | ENSG00000179630 | 0.524593178 | 5.55E-11 |
| ENSG00000260788 | ENSG00000115365 | 0.61279125 | 2.21E-15 |
| ENSG00000260788 | ENSG00000159753 | 0.559935904 | 1.37E-12 |
| ENSG00000260804 | ENSG00000134982 | 0.595830798 | 1.98E-14 |
| ENSG00000260804 | ENSG00000143126 | 0.541634869 | 9.83E-12 |
| ENSG00000260804 | ENSG00000132846 | 0.6027789 | 8.20E-15 |
| ENSG00000260804 | ENSG00000167986 | 0.533615907 | 2.25E-11 |
| ENSG00000260804 | ENSG00000154229 | 0.510627336 | 2.14E-10 |
| ENSG00000260804 | ENSG00000108306 | 0.649593959 | 1.18E-17 |
| ENSG00000260804 | ENSG00000116117 | 0.515284735 | 1.37E-10 |
| ENSG00000260804 | ENSG00000266173 | 0.601668177 | 9.45E-15 |
| ENSG00000260804 | ENSG00000173064 | 0.671260934 | 3.80E-19 |
| ENSG00000260804 | ENSG00000170456 | 0.57073698 | 4.04E-13 |
| ENSG00000260804 | ENSG00000028310 | 0.557062302 | 1.88E-12 |
| ENSG00000260804 | ENSG00000141564 | 0.54298702 | 8.53E-12 |
| ENSG00000260804 | ENSG00000010322 | 0.515038498 | 1.41E-10 |
| ENSG00000260804 | ENSG00000181222 | 0.510892559 | 2.09E-10 |
| ENSG00000260804 | ENSG00000172379 | 0.612834856 | 2.20E-15 |
| ENSG00000260804 | ENSG00000089234 | 0.545472106 | 6.56E-12 |
| ENSG00000260804 | ENSG00000175216 | 0.572216072 | 3.40E-13 |
| ENSG00000260804 | ENSG00000171634 | 0.657879061 | 3.28E-18 |
| ENSG00000260804 | ENSG00000102606 | 0.518816972 | 9.77E-11 |
| ENSG00000260804 | ENSG00000140632 | 0.531723242 | 2.72E-11 |
| ENSG00000260804 | ENSG00000077157 | 0.522720274 | 6.68E-11 |
| ENSG00000260804 | ENSG00000125447 | 0.51718534 | 1.14E-10 |
| ENSG00000260804 | ENSG00000145555 | 0.632201878 | 1.53E-16 |
| ENSG00000260804 | ENSG00000092421 | 0.560664006 | 1.26E-12 |
| ENSG00000260804 | ENSG00000008869 | 0.623717923 | 5.03E-16 |
| ENSG00000260804 | ENSG00000127616 | 0.608917761 | 3.69E-15 |
| ENSG00000260804 | ENSG00000139437 | 0.608019042 | 4.16E-15 |
| ENSG00000260804 | ENSG00000196914 | 0.567635654 | 5.76E-13 |
| ENSG00000260804 | ENSG00000115977 | 0.511376125 | 1.99E-10 |
| ENSG00000260804 | ENSG00000166783 | 0.563790981 | 8.90E-13 |
| ENSG00000260804 | ENSG00000166188 | 0.563012879 | 9.71E-13 |
| ENSG00000260804 | ENSG00000179981 | 0.510792143 | 2.11E-10 |
| ENSG00000260804 | ENSG00000108468 | 0.521401744 | 7.60E-11 |
| ENSG00000260804 | ENSG00000075856 | 0.591990472 | 3.19E-14 |
| ENSG00000260804 | ENSG00000087274 | 0.604984712 | 6.17E-15 |
| ENSG00000260804 | ENSG00000234616 | 0.598412388 | 1.43E-14 |
| ENSG00000260804 | ENSG00000179295 | 0.552820433 | 2.99E-12 |
| ENSG00000260804 | ENSG00000140718 | 0.591862771 | 3.25E-14 |
| ENSG00000260804 | ENSG00000141068 | 0.590537936 | 3.82E-14 |
| ENSG00000260804 | ENSG00000038532 | 0.563466356 | 9.23E-13 |
| ENSG00000260804 | ENSG00000157014 | 0.56927014 | 4.78E-13 |
| ENSG00000260804 | ENSG00000066933 | 0.507196326 | 2.95E-10 |
| ENSG00000260804 | ENSG00000130254 | 0.537860929 | 1.45E-11 |
| ENSG00000260804 | ENSG00000145348 | 0.502455655 | 4.58E-10 |
| ENSG00000260804 | ENSG00000112584 | 0.512095557 | 1.86E-10 |
| ENSG00000260804 | ENSG00000167522 | 0.504889399 | 3.66E-10 |
| ENSG00000260804 | ENSG00000170242 | 0.565828625 | 7.07E-13 |
| ENSG00000260804 | ENSG00000115109 | 0.586513797 | 6.25E-14 |
| ENSG00000260804 | ENSG00000100354 | 0.568825394 | 5.03E-13 |
| ENSG00000260804 | ENSG00000135090 | 0.500326434 | 5.57E-10 |
| ENSG00000260804 | ENSG00000113360 | 0.635216442 | 9.91E-17 |
| ENSG00000260804 | ENSG00000187189 | 0.515177511 | 1.39E-10 |
| ENSG00000260804 | ENSG00000167258 | 0.511188344 | 2.03E-10 |
| ENSG00000260804 | ENSG00000169180 | 0.531567284 | 2.77E-11 |
| ENSG00000260804 | ENSG00000133030 | 0.573680479 | 2.87E-13 |
| ENSG00000260804 | ENSG00000172057 | 0.511054078 | 2.05E-10 |
| ENSG00000260804 | ENSG00000156931 | 0.565080269 | 7.70E-13 |
| ENSG00000260804 | ENSG00000108424 | 0.519619114 | 9.04E-11 |
| ENSG00000260804 | ENSG00000138668 | 0.546923794 | 5.63E-12 |
| ENSG00000260804 | ENSG00000185818 | 0.509567423 | 2.36E-10 |
| ENSG00000260804 | ENSG00000135299 | 0.529141752 | 3.53E-11 |
| ENSG00000260804 | ENSG00000120948 | 0.579259628 | 1.49E-13 |
| ENSG00000260804 | ENSG00000084676 | 0.531939489 | 2.66E-11 |
| ENSG00000260804 | ENSG00000088367 | 0.512591623 | 1.78E-10 |
| ENSG00000260804 | ENSG00000100105 | 0.560552134 | 1.28E-12 |
| ENSG00000260804 | ENSG00000164190 | 0.560110505 | 1.34E-12 |
| ENSG00000260804 | ENSG00000106479 | 0.50315763 | 4.29E-10 |
| ENSG00000260804 | ENSG00000117713 | 0.582847194 | 9.73E-14 |
| ENSG00000260804 | ENSG00000084710 | 0.512590092 | 1.78E-10 |
| ENSG00000260804 | ENSG00000103657 | 0.601246387 | 9.98E-15 |
| ENSG00000260804 | ENSG00000116698 | 0.547668341 | 5.20E-12 |
| ENSG00000260804 | ENSG00000170921 | 0.701780334 | 1.79E-21 |
| ENSG00000260804 | ENSG00000157087 | 0.503577517 | 4.13E-10 |
| ENSG00000260804 | ENSG00000162664 | 0.582675172 | 9.93E-14 |
| ENSG00000260804 | ENSG00000033627 | 0.610354464 | 3.06E-15 |
| ENSG00000260804 | ENSG00000257923 | 0.540102993 | 1.15E-11 |
| ENSG00000260804 | ENSG00000105429 | 0.5116539 | 1.94E-10 |
| ENSG00000260804 | ENSG00000139613 | 0.549051237 | 4.49E-12 |
| ENSG00000260804 | ENSG00000125686 | 0.616884177 | 1.28E-15 |
| ENSG00000260804 | ENSG00000128872 | 0.522899977 | 6.56E-11 |
| ENSG00000260804 | ENSG00000149657 | 0.574448329 | 2.63E-13 |
| ENSG00000260804 | ENSG00000108262 | 0.538168689 | 1.41E-11 |
| ENSG00000260804 | ENSG00000143376 | 0.590459847 | 3.86E-14 |
| ENSG00000260804 | ENSG00000257093 | 0.533092343 | 2.37E-11 |
| ENSG00000260804 | ENSG00000152102 | 0.662887438 | 1.48E-18 |
| ENSG00000260804 | ENSG00000123200 | 0.543684232 | 7.93E-12 |
| ENSG00000260804 | ENSG00000105662 | 0.559885601 | 1.38E-12 |
| ENSG00000260804 | ENSG00000163125 | 0.509046943 | 2.48E-10 |
| ENSG00000260804 | ENSG00000157077 | 0.527640122 | 4.10E-11 |
| ENSG00000260804 | ENSG00000224470 | 0.531550525 | 2.77E-11 |
| ENSG00000260804 | ENSG00000136715 | 0.509359661 | 2.41E-10 |
| ENSG00000260804 | ENSG00000134313 | 0.650181423 | 1.08E-17 |
| ENSG00000260804 | ENSG00000078687 | 0.692361416 | 1.00E-20 |
| ENSG00000260804 | ENSG00000110066 | 0.59513412 | 2.16E-14 |
| ENSG00000260804 | ENSG00000102858 | 0.51066014 | 2.13E-10 |
| ENSG00000260804 | ENSG00000076641 | 0.567570098 | 5.80E-13 |
| ENSG00000260804 | ENSG00000070778 | 0.511585178 | 1.95E-10 |
| ENSG00000260804 | ENSG00000108819 | 0.523298442 | 6.31E-11 |
| ENSG00000260804 | ENSG00000136828 | 0.583475061 | 9.03E-14 |
| ENSG00000260804 | ENSG00000151276 | 0.640651834 | 4.49E-17 |
| ENSG00000260804 | ENSG00000118689 | 0.540891424 | 1.06E-11 |
| ENSG00000260804 | ENSG00000140948 | 0.635537175 | 9.46E-17 |
| ENSG00000260804 | ENSG00000122965 | 0.546441153 | 5.92E-12 |
| ENSG00000260804 | ENSG00000155858 | 0.507647117 | 2.83E-10 |
| ENSG00000260804 | ENSG00000196391 | 0.552950325 | 2.95E-12 |
| ENSG00000260804 | ENSG00000187605 | 0.578705182 | 1.59E-13 |
| ENSG00000260804 | ENSG00000170004 | 0.525918095 | 4.87E-11 |
| ENSG00000260804 | ENSG00000158941 | 0.523572097 | 6.14E-11 |
| ENSG00000260804 | ENSG00000139718 | 0.542196316 | 9.27E-12 |
| ENSG00000260804 | ENSG00000205336 | 0.528381484 | 3.81E-11 |
| ENSG00000260804 | ENSG00000156639 | 0.511395795 | 1.99E-10 |
| ENSG00000260804 | ENSG00000135503 | 0.548086645 | 4.97E-12 |
| ENSG00000260804 | ENSG00000180357 | 0.57144458 | 3.72E-13 |
| ENSG00000260804 | ENSG00000075292 | 0.533432138 | 2.29E-11 |
| ENSG00000260804 | ENSG00000141027 | 0.642256624 | 3.54E-17 |
| ENSG00000260804 | ENSG00000137337 | 0.541581303 | 9.88E-12 |
| ENSG00000260804 | ENSG00000197323 | 0.54183868 | 9.62E-12 |
| ENSG00000260804 | ENSG00000151422 | 0.619816644 | 8.59E-16 |
| ENSG00000260804 | ENSG00000151502 | 0.598181269 | 1.47E-14 |
| ENSG00000260804 | ENSG00000274211 | 0.70361172 | 1.27E-21 |
| ENSG00000260804 | ENSG00000086758 | 0.551020894 | 3.63E-12 |
| ENSG00000260804 | ENSG00000132604 | 0.574118056 | 2.73E-13 |
| ENSG00000260804 | ENSG00000065526 | 0.607580492 | 4.40E-15 |
| ENSG00000260804 | ENSG00000172534 | 0.590893453 | 3.66E-14 |
| ENSG00000260804 | ENSG00000164576 | 0.505316758 | 3.52E-10 |
| ENSG00000260804 | ENSG00000115568 | 0.618923451 | 9.70E-16 |
| ENSG00000260804 | ENSG00000187555 | 0.55874476 | 1.56E-12 |
| ENSG00000260804 | ENSG00000168259 | 0.535584525 | 1.84E-11 |
| ENSG00000260804 | ENSG00000183495 | 0.633060831 | 1.35E-16 |
| ENSG00000260804 | ENSG00000074755 | 0.579665473 | 1.42E-13 |
| ENSG00000260804 | ENSG00000059145 | 0.56834183 | 5.31E-13 |
| ENSG00000260804 | ENSG00000215421 | 0.553948952 | 2.64E-12 |
| ENSG00000260804 | ENSG00000061936 | 0.569332625 | 4.74E-13 |
| ENSG00000260804 | ENSG00000140992 | 0.532164687 | 2.60E-11 |
| ENSG00000260804 | ENSG00000108175 | 0.56141983 | 1.16E-12 |
| ENSG00000260804 | ENSG00000115839 | 0.537971424 | 1.44E-11 |
| ENSG00000260804 | ENSG00000167971 | 0.530584661 | 3.05E-11 |
| ENSG00000260804 | ENSG00000275832 | 0.557999238 | 1.70E-12 |
| ENSG00000260804 | ENSG00000164402 | 0.588577868 | 4.86E-14 |
| ENSG00000260804 | ENSG00000133812 | 0.531605211 | 2.75E-11 |
| ENSG00000260804 | ENSG00000174231 | 0.553829021 | 2.68E-12 |
| ENSG00000260804 | ENSG00000109756 | 0.636741065 | 7.95E-17 |
| ENSG00000260804 | ENSG00000135365 | 0.503463537 | 4.17E-10 |
| ENSG00000260804 | ENSG00000116539 | 0.516854974 | 1.18E-10 |
| ENSG00000260804 | ENSG00000160633 | 0.548495607 | 4.76E-12 |
| ENSG00000260804 | ENSG00000071054 | 0.566015365 | 6.92E-13 |
| ENSG00000260804 | ENSG00000149187 | 0.543569405 | 8.02E-12 |
| ENSG00000260804 | ENSG00000166123 | 0.579682642 | 1.42E-13 |
| ENSG00000260804 | ENSG00000183826 | 0.615056313 | 1.64E-15 |
| ENSG00000260804 | ENSG00000104067 | 0.515016895 | 1.41E-10 |
| ENSG00000260804 | ENSG00000163625 | 0.567181707 | 6.07E-13 |
| ENSG00000260804 | ENSG00000112365 | 0.510379579 | 2.19E-10 |
| ENSG00000260804 | ENSG00000066739 | 0.509215938 | 2.44E-10 |
| ENSG00000260804 | ENSG00000146587 | 0.509889362 | 2.29E-10 |
| ENSG00000260804 | ENSG00000270647 | 0.587950171 | 5.25E-14 |
| ENSG00000260804 | ENSG00000182095 | 0.523776912 | 6.02E-11 |
| ENSG00000260804 | ENSG00000011021 | 0.545989427 | 6.21E-12 |
| ENSG00000260804 | ENSG00000144535 | 0.600624834 | 1.08E-14 |
| ENSG00000260804 | ENSG00000068654 | 0.583366254 | 9.14E-14 |
| ENSG00000260804 | ENSG00000005339 | 0.551462812 | 3.46E-12 |
| ENSG00000260804 | ENSG00000131018 | 0.524619447 | 5.54E-11 |
| ENSG00000260804 | ENSG00000197386 | 0.60740116 | 4.51E-15 |
| ENSG00000260804 | ENSG00000164880 | 0.50793847 | 2.75E-10 |
| ENSG00000260804 | ENSG00000112787 | 0.514749439 | 1.45E-10 |
| ENSG00000260804 | ENSG00000110395 | 0.601505071 | 9.65E-15 |
| ENSG00000260804 | ENSG00000089280 | 0.558895213 | 1.54E-12 |
| ENSG00000260804 | ENSG00000108510 | 0.550495606 | 3.84E-12 |
| ENSG00000260804 | ENSG00000166860 | 0.55935986 | 1.46E-12 |
| ENSG00000260804 | ENSG00000104731 | 0.527131269 | 4.32E-11 |
| ENSG00000260804 | ENSG00000075340 | 0.56793854 | 5.56E-13 |
| ENSG00000260804 | ENSG00000083168 | 0.519146745 | 9.46E-11 |
| ENSG00000260804 | ENSG00000169925 | 0.511902609 | 1.90E-10 |
| ENSG00000260804 | ENSG00000087365 | 0.505394572 | 3.49E-10 |
| ENSG00000260804 | ENSG00000073910 | 0.502466806 | 4.58E-10 |
| ENSG00000260804 | ENSG00000204842 | 0.572693794 | 3.22E-13 |
| ENSG00000260804 | ENSG00000184014 | 0.520527539 | 8.27E-11 |
| ENSG00000260804 | ENSG00000187391 | 0.538591055 | 1.35E-11 |
| ENSG00000260804 | ENSG00000123066 | 0.569193059 | 4.82E-13 |
| ENSG00000260804 | ENSG00000204569 | 0.587238708 | 5.72E-14 |
| ENSG00000260804 | ENSG00000070614 | 0.672470686 | 3.11E-19 |
| ENSG00000260804 | ENSG00000177728 | 0.655732913 | 4.58E-18 |
| ENSG00000260804 | ENSG00000008083 | 0.511890605 | 1.90E-10 |
| ENSG00000260804 | ENSG00000185722 | 0.604984779 | 6.17E-15 |
| ENSG00000260804 | ENSG00000172915 | 0.504100796 | 3.94E-10 |
| ENSG00000260804 | ENSG00000005810 | 0.510561174 | 2.15E-10 |
| ENSG00000260804 | ENSG00000197283 | 0.512040528 | 1.87E-10 |
| ENSG00000260804 | ENSG00000166716 | 0.521796863 | 7.31E-11 |
| ENSG00000260804 | ENSG00000275023 | 0.66998695 | 4.69E-19 |
| ENSG00000260804 | ENSG00000198863 | 0.566700088 | 6.41E-13 |
| ENSG00000260804 | ENSG00000205269 | 0.531738183 | 2.72E-11 |
| ENSG00000260917 | ENSG00000180357 | 0.623582381 | 5.12E-16 |
| ENSG00000260917 | ENSG00000163939 | 0.547462405 | 5.31E-12 |
| ENSG00000260917 | ENSG00000099204 | 0.502392927 | 4.61E-10 |
| ENSG00000260917 | ENSG00000065060 | 0.511454518 | 1.98E-10 |
| ENSG00000260917 | ENSG00000107829 | 0.614698221 | 1.72E-15 |
| ENSG00000260917 | ENSG00000179981 | 0.522425771 | 6.87E-11 |
| ENSG00000260917 | ENSG00000140320 | 0.536746763 | 1.63E-11 |
| ENSG00000260917 | ENSG00000124198 | 0.561845808 | 1.11E-12 |
| ENSG00000260917 | ENSG00000135503 | 0.518432364 | 1.01E-10 |
| ENSG00000260917 | ENSG00000144674 | 0.527234272 | 4.27E-11 |
| ENSG00000260917 | ENSG00000083168 | 0.518295672 | 1.03E-10 |
| ENSG00000260917 | ENSG00000108306 | 0.672795746 | 2.95E-19 |
| ENSG00000260917 | ENSG00000175727 | 0.628840601 | 2.46E-16 |
| ENSG00000260917 | ENSG00000123066 | 0.580166866 | 1.34E-13 |
| ENSG00000260917 | ENSG00000136872 | 0.525791198 | 4.93E-11 |
| ENSG00000260917 | ENSG00000171634 | 0.640254063 | 4.76E-17 |
| ENSG00000260917 | ENSG00000187742 | 0.524286433 | 5.72E-11 |
| ENSG00000260917 | ENSG00000160299 | 0.622414566 | 6.02E-16 |
| ENSG00000260917 | ENSG00000152223 | 0.572174042 | 3.42E-13 |
| ENSG00000260917 | ENSG00000157216 | 0.548095007 | 4.97E-12 |
| ENSG00000260917 | ENSG00000100285 | 0.572250074 | 3.39E-13 |
| ENSG00000260917 | ENSG00000167280 | 0.515021328 | 1.41E-10 |
| ENSG00000260917 | ENSG00000165424 | 0.50490319 | 3.65E-10 |
| ENSG00000260917 | ENSG00000130939 | 0.54921151 | 4.41E-12 |
| ENSG00000260917 | ENSG00000204842 | 0.585243584 | 7.29E-14 |
| ENSG00000260917 | ENSG00000005339 | 0.573639696 | 2.89E-13 |
| ENSG00000260917 | ENSG00000166860 | 0.506079029 | 3.28E-10 |
| ENSG00000260917 | ENSG00000151461 | 0.631494506 | 1.69E-16 |
| ENSG00000260917 | ENSG00000077157 | 0.587961341 | 5.24E-14 |
| ENSG00000260917 | ENSG00000134313 | 0.61254188 | 2.29E-15 |
| ENSG00000260917 | ENSG00000160460 | 0.508657694 | 2.57E-10 |
| ENSG00000260917 | ENSG00000127616 | 0.540024489 | 1.16E-11 |
| ENSG00000260917 | ENSG00000197283 | 0.610313483 | 3.07E-15 |
| ENSG00000260917 | ENSG00000184787 | 0.529283033 | 3.48E-11 |
| ENSG00000260917 | ENSG00000197323 | 0.519718159 | 8.95E-11 |
| ENSG00000260917 | ENSG00000181222 | 0.580532589 | 1.28E-13 |
| ENSG00000260917 | ENSG00000140948 | 0.659795311 | 2.42E-18 |
| ENSG00000260917 | ENSG00000139998 | 0.542967328 | 8.55E-12 |
| ENSG00000260917 | ENSG00000135365 | 0.503563229 | 4.14E-10 |
| ENSG00000260917 | ENSG00000198825 | 0.501253519 | 5.11E-10 |
| ENSG00000260917 | ENSG00000167291 | 0.578894612 | 1.56E-13 |
| ENSG00000260917 | ENSG00000112787 | 0.638823911 | 5.87E-17 |
| ENSG00000260917 | ENSG00000065526 | 0.609000047 | 3.65E-15 |
| ENSG00000260917 | ENSG00000086758 | 0.652889682 | 7.12E-18 |
| ENSG00000260917 | ENSG00000008086 | 0.741942329 | 5.00E-25 |
| ENSG00000260917 | ENSG00000169925 | 0.53641485 | 1.69E-11 |
| ENSG00000260917 | ENSG00000170325 | 0.501255983 | 5.11E-10 |
| ENSG00000260917 | ENSG00000172985 | 0.557304329 | 1.83E-12 |
| ENSG00000260917 | ENSG00000224470 | 0.581507624 | 1.14E-13 |
| ENSG00000260917 | ENSG00000066933 | 0.548566274 | 4.72E-12 |
| ENSG00000260917 | ENSG00000110395 | 0.571861024 | 3.55E-13 |
| ENSG00000260917 | ENSG00000177169 | 0.662139491 | 1.67E-18 |
| ENSG00000260917 | ENSG00000128191 | 0.546493103 | 5.89E-12 |
| ENSG00000260917 | ENSG00000130338 | 0.760279228 | 7.06E-27 |
| ENSG00000260917 | ENSG00000091157 | 0.520413727 | 8.37E-11 |
| ENSG00000260917 | ENSG00000165125 | 0.573104966 | 3.07E-13 |
| ENSG00000260917 | ENSG00000108175 | 0.736436848 | 1.68E-24 |
| ENSG00000260917 | ENSG00000114648 | 0.500933648 | 5.27E-10 |
| ENSG00000260917 | ENSG00000160294 | 0.555801139 | 2.16E-12 |
| ENSG00000260917 | ENSG00000158545 | 0.52649475 | 4.60E-11 |
| ENSG00000260917 | ENSG00000089280 | 0.513657928 | 1.60E-10 |
| ENSG00000260917 | ENSG00000115977 | 0.778554246 | 6.79E-29 |
| ENSG00000260917 | ENSG00000170145 | 0.542122312 | 9.34E-12 |
| ENSG00000260917 | ENSG00000133030 | 0.610986212 | 2.81E-15 |
| ENSG00000260917 | ENSG00000160218 | 0.572543858 | 3.28E-13 |
| ENSG00000260917 | ENSG00000038358 | 0.568578224 | 5.17E-13 |
| ENSG00000260917 | ENSG00000139112 | 0.507901367 | 2.76E-10 |
| ENSG00000260917 | ENSG00000148337 | 0.523800982 | 6.00E-11 |
| ENSG00000260917 | ENSG00000155858 | 0.524094559 | 5.83E-11 |
| ENSG00000260917 | ENSG00000198794 | 0.509412046 | 2.40E-10 |
| ENSG00000260917 | ENSG00000087157 | 0.586240736 | 6.46E-14 |
| ENSG00000260917 | ENSG00000132740 | 0.579602333 | 1.43E-13 |
| ENSG00000260917 | ENSG00000075292 | 0.650017293 | 1.10E-17 |
| ENSG00000260917 | ENSG00000164506 | 0.606007197 | 5.40E-15 |
| ENSG00000260917 | ENSG00000116698 | 0.573826859 | 2.82E-13 |
| ENSG00000260917 | ENSG00000070614 | 0.612687184 | 2.24E-15 |
| ENSG00000260917 | ENSG00000172915 | 0.520015811 | 8.70E-11 |
| ENSG00000260917 | ENSG00000117016 | 0.586145559 | 6.54E-14 |
| ENSG00000260917 | ENSG00000112365 | 0.55063055 | 3.79E-12 |
| ENSG00000260917 | ENSG00000071054 | 0.629462814 | 2.25E-16 |
| ENSG00000260917 | ENSG00000110400 | 0.668317079 | 6.16E-19 |
| ENSG00000260917 | ENSG00000159433 | 0.596781315 | 1.76E-14 |
| ENSG00000260917 | ENSG00000169180 | 0.571024857 | 3.91E-13 |
| ENSG00000260917 | ENSG00000136828 | 0.597781973 | 1.55E-14 |
| ENSG00000260917 | ENSG00000065613 | 0.503168094 | 4.29E-10 |
| ENSG00000260917 | ENSG00000131018 | 0.603585733 | 7.39E-15 |
| ENSG00000260917 | ENSG00000100077 | 0.534404521 | 2.07E-11 |
| ENSG00000260917 | ENSG00000078687 | 0.741049426 | 6.10E-25 |
| ENSG00000260917 | ENSG00000153201 | 0.501423221 | 5.04E-10 |
| ENSG00000260917 | ENSG00000125447 | 0.625423135 | 3.97E-16 |
| ENSG00000260917 | ENSG00000119946 | 0.559898703 | 1.37E-12 |
| ENSG00000260917 | ENSG00000107560 | 0.501774963 | 4.88E-10 |
| ENSG00000260917 | ENSG00000177570 | 0.593071548 | 2.79E-14 |
| ENSG00000260917 | ENSG00000170921 | 0.61228543 | 2.37E-15 |
| ENSG00000260917 | ENSG00000111249 | 0.578361901 | 1.66E-13 |
| ENSG00000260917 | ENSG00000148356 | 0.576740058 | 2.01E-13 |
| ENSG00000260917 | ENSG00000164068 | 0.602851941 | 8.12E-15 |
| ENSG00000260917 | ENSG00000122965 | 0.586123489 | 6.56E-14 |
| ENSG00000260917 | ENSG00000112584 | 0.506576203 | 3.13E-10 |
| ENSG00000260917 | ENSG00000134698 | 0.642732625 | 3.30E-17 |
| ENSG00000260917 | ENSG00000164236 | 0.572310472 | 3.37E-13 |
| ENSG00000260917 | ENSG00000138944 | 0.522539476 | 6.80E-11 |
| ENSG00000260917 | ENSG00000117713 | 0.590238654 | 3.97E-14 |
| ENSG00000260917 | ENSG00000121671 | 0.528473967 | 3.77E-11 |
| ENSG00000260917 | ENSG00000154118 | 0.571774122 | 3.58E-13 |
| ENSG00000260917 | ENSG00000164190 | 0.556748291 | 1.95E-12 |
| ENSG00000260917 | ENSG00000137337 | 0.543457305 | 8.12E-12 |
| ENSG00000260917 | ENSG00000100354 | 0.558506606 | 1.60E-12 |
| ENSG00000260917 | ENSG00000066739 | 0.674639104 | 2.17E-19 |
| ENSG00000260917 | ENSG00000149187 | 0.540649404 | 1.09E-11 |
| ENSG00000260917 | ENSG00000170456 | 0.515892391 | 1.30E-10 |
| ENSG00000260917 | ENSG00000179832 | 0.576730685 | 2.01E-13 |
| ENSG00000260917 | ENSG00000163125 | 0.547954254 | 5.04E-12 |
| ENSG00000260917 | ENSG00000173064 | 0.797670576 | 3.20E-31 |
| ENSG00000260917 | ENSG00000104731 | 0.530300424 | 3.14E-11 |
| ENSG00000260917 | ENSG00000164880 | 0.507451338 | 2.88E-10 |
| ENSG00000260917 | ENSG00000204469 | 0.54042877 | 1.11E-11 |
| ENSG00000260917 | ENSG00000168488 | 0.550986843 | 3.64E-12 |
| ENSG00000260917 | ENSG00000006740 | 0.575987217 | 2.19E-13 |
| ENSG00000260917 | ENSG00000166135 | 0.752289225 | 4.73E-26 |
| ENSG00000260917 | ENSG00000183337 | 0.512462518 | 1.80E-10 |
| ENSG00000260917 | ENSG00000061936 | 0.641078108 | 4.22E-17 |
| ENSG00000260917 | ENSG00000054793 | 0.570582216 | 4.11E-13 |
| ENSG00000260917 | ENSG00000197312 | 0.54570297 | 6.41E-12 |
| ENSG00000260917 | ENSG00000197386 | 0.696066666 | 5.13E-21 |
| ENSG00000260917 | ENSG00000120256 | 0.525089225 | 5.29E-11 |
| ENSG00000260917 | ENSG00000205726 | 0.572064979 | 3.46E-13 |
| ENSG00000260917 | ENSG00000187605 | 0.681175073 | 7.16E-20 |
| ENSG00000260917 | ENSG00000183495 | 0.639089504 | 5.64E-17 |
| ENSG00000260917 | ENSG00000033627 | 0.674190608 | 2.34E-19 |
| ENSG00000260917 | ENSG00000167258 | 0.505182487 | 3.56E-10 |
| ENSG00000260917 | ENSG00000116539 | 0.560469947 | 1.29E-12 |
| ENSG00000260917 | ENSG00000127585 | 0.563319727 | 9.38E-13 |
| ENSG00000260917 | ENSG00000139182 | 0.604812714 | 6.31E-15 |
| ENSG00000260917 | ENSG00000235194 | 0.520405792 | 8.37E-11 |
| ENSG00000260917 | ENSG00000104722 | 0.514909117 | 1.42E-10 |
| ENSG00000260917 | ENSG00000166197 | 0.612018213 | 2.45E-15 |
| ENSG00000260917 | ENSG00000183826 | 0.670974391 | 3.98E-19 |
| ENSG00000260917 | ENSG00000155744 | 0.506801455 | 3.06E-10 |
| ENSG00000260917 | ENSG00000140548 | 0.512383913 | 1.81E-10 |
| ENSG00000260917 | ENSG00000266173 | 0.515080777 | 1.40E-10 |
| ENSG00000260917 | ENSG00000106089 | 0.508205373 | 2.69E-10 |
| ENSG00000260917 | ENSG00000103150 | 0.527248502 | 4.27E-11 |
| ENSG00000260917 | ENSG00000084710 | 0.56688319 | 6.27E-13 |
| ENSG00000260917 | ENSG00000158669 | 0.501783152 | 4.87E-10 |
| ENSG00000260917 | ENSG00000153815 | 0.57730032 | 1.88E-13 |
| ENSG00000260917 | ENSG00000008083 | 0.610114805 | 3.16E-15 |
| ENSG00000260917 | ENSG00000005810 | 0.663992603 | 1.24E-18 |
| ENSG00000260917 | ENSG00000143376 | 0.575301487 | 2.38E-13 |
| ENSG00000260917 | ENSG00000087274 | 0.508905634 | 2.52E-10 |
| ENSG00000260917 | ENSG00000162104 | 0.501447245 | 5.02E-10 |
| ENSG00000260917 | ENSG00000196547 | 0.549830469 | 4.13E-12 |
| ENSG00000260917 | ENSG00000108557 | 0.557090679 | 1.87E-12 |
| ENSG00000260917 | ENSG00000163625 | 0.503145084 | 4.30E-10 |
| ENSG00000260917 | ENSG00000075856 | 0.501955289 | 4.80E-10 |
| ENSG00000260917 | ENSG00000172534 | 0.612654293 | 2.25E-15 |
| ENSG00000260917 | ENSG00000175662 | 0.648417214 | 1.41E-17 |
| ENSG00000260917 | ENSG00000125686 | 0.54049933 | 1.11E-11 |
| ENSG00000260917 | ENSG00000169914 | 0.603868234 | 7.13E-15 |
| ENSG00000260917 | ENSG00000170871 | 0.533302139 | 2.32E-11 |
| ENSG00000260917 | ENSG00000064999 | 0.630543532 | 1.93E-16 |
| ENSG00000260917 | ENSG00000187555 | 0.564614748 | 8.11E-13 |
| ENSG00000260917 | ENSG00000204843 | 0.617512493 | 1.18E-15 |
| ENSG00000260917 | ENSG00000148843 | 0.608842883 | 3.73E-15 |
| ENSG00000260917 | ENSG00000088854 | 0.685423824 | 3.43E-20 |
| ENSG00000260917 | ENSG00000122966 | 0.608794416 | 3.75E-15 |
| ENSG00000260917 | ENSG00000148541 | 0.562533516 | 1.02E-12 |
| ENSG00000260917 | ENSG00000048471 | 0.539197681 | 1.27E-11 |
| ENSG00000260917 | ENSG00000171132 | 0.618368359 | 1.05E-15 |
| ENSG00000260917 | ENSG00000109756 | 0.661231487 | 1.93E-18 |
| ENSG00000260917 | ENSG00000160305 | 0.688265795 | 2.08E-20 |
| ENSG00000260917 | ENSG00000140332 | 0.535653283 | 1.82E-11 |
| ENSG00000260917 | ENSG00000215421 | 0.597014256 | 1.71E-14 |
| ENSG00000260917 | ENSG00000129473 | 0.643322592 | 3.02E-17 |
| ENSG00000260917 | ENSG00000186635 | 0.508652264 | 2.58E-10 |
| ENSG00000260917 | ENSG00000108669 | 0.669621148 | 4.98E-19 |
| ENSG00000260917 | ENSG00000039523 | 0.614899357 | 1.67E-15 |
| ENSG00000260917 | ENSG00000166716 | 0.513560807 | 1.62E-10 |
| ENSG00000260917 | ENSG00000275832 | 0.62550107 | 3.93E-16 |
| ENSG00000260917 | ENSG00000170004 | 0.640607374 | 4.52E-17 |
| ENSG00000260917 | ENSG00000053254 | 0.513992542 | 1.55E-10 |
| ENSG00000260917 | ENSG00000174231 | 0.503189016 | 4.28E-10 |
| ENSG00000260917 | ENSG00000274211 | 0.723640467 | 2.49E-23 |
| ENSG00000260917 | ENSG00000076513 | 0.572032001 | 3.48E-13 |
| ENSG00000260917 | ENSG00000085733 | 0.587859684 | 5.31E-14 |
| ENSG00000260917 | ENSG00000078328 | 0.52670313 | 4.50E-11 |
| ENSG00000260917 | ENSG00000077235 | 0.509306808 | 2.42E-10 |
| ENSG00000260917 | ENSG00000160633 | 0.540530502 | 1.10E-11 |
| ENSG00000260917 | ENSG00000131242 | 0.622789189 | 5.72E-16 |
| ENSG00000260917 | ENSG00000197562 | 0.532184237 | 2.60E-11 |
| ENSG00000260917 | ENSG00000196233 | 0.600056564 | 1.16E-14 |
| ENSG00000260917 | ENSG00000091622 | 0.523290695 | 6.31E-11 |
| ENSG00000260917 | ENSG00000011347 | 0.549628824 | 4.22E-12 |
| ENSG00000260917 | ENSG00000152484 | 0.50212113 | 4.72E-10 |
| ENSG00000260966 | ENSG00000173064 | 0.541845205 | 9.61E-12 |
| ENSG00000260966 | ENSG00000127585 | 0.525372435 | 5.14E-11 |
| ENSG00000260966 | ENSG00000088179 | 0.547959782 | 5.04E-12 |
| ENSG00000260966 | ENSG00000116675 | 0.509927709 | 2.29E-10 |
| ENSG00000260966 | ENSG00000123200 | 0.505735774 | 3.38E-10 |
| ENSG00000260966 | ENSG00000123901 | 0.541380639 | 1.01E-11 |
| ENSG00000260966 | ENSG00000102606 | 0.529880572 | 3.28E-11 |
| ENSG00000260966 | ENSG00000103657 | 0.50349599 | 4.16E-10 |
| ENSG00000260966 | ENSG00000136828 | 0.506896228 | 3.04E-10 |
| ENSG00000260966 | ENSG00000198794 | 0.592978705 | 2.83E-14 |
| ENSG00000260966 | ENSG00000167861 | 0.526663522 | 4.52E-11 |
| ENSG00000260966 | ENSG00000205269 | 0.537148852 | 1.56E-11 |
| ENSG00000260966 | ENSG00000155744 | 0.53440417 | 2.07E-11 |
| ENSG00000260966 | ENSG00000125814 | 0.615320349 | 1.58E-15 |
| ENSG00000260966 | ENSG00000109756 | 0.547039109 | 5.56E-12 |
| ENSG00000260966 | ENSG00000274211 | 0.509261682 | 2.43E-10 |
| ENSG00000260966 | ENSG00000033627 | 0.517918025 | 1.07E-10 |
| ENSG00000260966 | ENSG00000179295 | 0.501532153 | 4.99E-10 |
| ENSG00000260966 | ENSG00000005810 | 0.59151756 | 3.39E-14 |
| ENSG00000260966 | ENSG00000171132 | 0.562675161 | 1.01E-12 |
| ENSG00000260966 | ENSG00000111911 | 0.54826 | 4.88E-12 |
| ENSG00000260966 | ENSG00000078687 | 0.518874044 | 9.72E-11 |
| ENSG00000260966 | ENSG00000189241 | 0.517186843 | 1.14E-10 |
| ENSG00000260966 | ENSG00000120903 | 0.508048285 | 2.73E-10 |
| ENSG00000260966 | ENSG00000087470 | 0.502238974 | 4.67E-10 |
| ENSG00000260966 | ENSG00000177570 | 0.565785688 | 7.11E-13 |
| ENSG00000260966 | ENSG00000167971 | 0.575938319 | 2.21E-13 |
| ENSG00000260966 | ENSG00000101098 | 0.512322573 | 1.82E-10 |
| ENSG00000260966 | ENSG00000175874 | 0.511371594 | 1.99E-10 |
| ENSG00000260966 | ENSG00000054793 | 0.52963401 | 3.36E-11 |
| ENSG00000260966 | ENSG00000172915 | 0.633783564 | 1.22E-16 |
| ENSG00000260966 | ENSG00000139112 | 0.500170299 | 5.65E-10 |
| ENSG00000260966 | ENSG00000073910 | 0.619157872 | 9.40E-16 |
| ENSG00000260966 | ENSG00000155858 | 0.503695486 | 4.09E-10 |
| ENSG00000260966 | ENSG00000121671 | 0.547523653 | 5.28E-12 |
| ENSG00000260966 | ENSG00000187189 | 0.53611738 | 1.74E-11 |
| ENSG00000260966 | ENSG00000138078 | 0.502053908 | 4.75E-10 |
| ENSG00000260997 | ENSG00000136048 | 0.500723767 | 5.37E-10 |
| ENSG00000260997 | ENSG00000108582 | 0.577943687 | 1.74E-13 |
| ENSG00000260997 | ENSG00000111424 | 0.696980142 | 4.34E-21 |
| ENSG00000260997 | ENSG00000266094 | 0.514293105 | 1.51E-10 |
| ENSG00000260997 | ENSG00000100055 | 0.613675543 | 1.97E-15 |
| ENSG00000260997 | ENSG00000137841 | 0.516379747 | 1.24E-10 |
| ENSG00000260997 | ENSG00000104972 | 0.592156346 | 3.13E-14 |
| ENSG00000260997 | ENSG00000118503 | 0.674785587 | 2.12E-19 |
| ENSG00000260997 | ENSG00000010610 | 0.529044364 | 3.56E-11 |
| ENSG00000260997 | ENSG00000153071 | 0.587730464 | 5.39E-14 |
| ENSG00000260997 | ENSG00000086062 | 0.564028921 | 8.66E-13 |
| ENSG00000261019 | ENSG00000160305 | 0.543138157 | 8.40E-12 |
| ENSG00000261019 | ENSG00000173064 | 0.567944504 | 5.56E-13 |
| ENSG00000261019 | ENSG00000140992 | 0.542803272 | 8.70E-12 |
| ENSG00000261019 | ENSG00000157087 | 0.535181161 | 1.91E-11 |
| ENSG00000261019 | ENSG00000059145 | 0.506103103 | 3.27E-10 |
| ENSG00000261019 | ENSG00000172375 | 0.635673929 | 9.27E-17 |
| ENSG00000261019 | ENSG00000122966 | 0.54444569 | 7.32E-12 |
| ENSG00000261037 | ENSG00000078328 | 0.633614322 | 1.25E-16 |
| ENSG00000261037 | ENSG00000164506 | 0.531443687 | 2.80E-11 |
| ENSG00000261037 | ENSG00000181418 | 0.688249107 | 2.09E-20 |
| ENSG00000261037 | ENSG00000165983 | 0.623587115 | 5.12E-16 |
| ENSG00000261037 | ENSG00000125814 | 0.620068263 | 8.30E-16 |
| ENSG00000261037 | ENSG00000091622 | 0.55840388 | 1.62E-12 |
| ENSG00000261037 | ENSG00000171126 | 0.614970905 | 1.65E-15 |
| ENSG00000261037 | ENSG00000197106 | 0.631170242 | 1.77E-16 |
| ENSG00000261037 | ENSG00000183780 | 0.603328436 | 7.64E-15 |
| ENSG00000261037 | ENSG00000163630 | 0.654872547 | 5.24E-18 |
| ENSG00000261037 | ENSG00000072657 | 0.663282548 | 1.39E-18 |
| ENSG00000261037 | ENSG00000123612 | 0.569504174 | 4.65E-13 |
| ENSG00000261037 | ENSG00000104722 | 0.517955965 | 1.06E-10 |
| ENSG00000261037 | ENSG00000135750 | 0.565450406 | 7.38E-13 |
| ENSG00000261037 | ENSG00000060140 | 0.705489452 | 8.90E-22 |
| ENSG00000261037 | ENSG00000004660 | 0.566065749 | 6.88E-13 |
| ENSG00000261037 | ENSG00000153933 | 0.516989245 | 1.17E-10 |
| ENSG00000261037 | ENSG00000137843 | 0.514104456 | 1.54E-10 |
| ENSG00000261037 | ENSG00000123901 | 0.705295327 | 9.24E-22 |
| ENSG00000261037 | ENSG00000147676 | 0.67979224 | 9.07E-20 |
| ENSG00000261037 | ENSG00000011347 | 0.530036794 | 3.23E-11 |
| ENSG00000261037 | ENSG00000050748 | 0.512647167 | 1.77E-10 |
| ENSG00000261037 | ENSG00000107758 | 0.550714878 | 3.75E-12 |
| ENSG00000261087 | ENSG00000033627 | 0.504819015 | 3.68E-10 |
| ENSG00000261087 | ENSG00000011021 | 0.547144346 | 5.50E-12 |
| ENSG00000261087 | ENSG00000275023 | 0.598939425 | 1.34E-14 |
| ENSG00000261087 | ENSG00000177169 | 0.506469435 | 3.16E-10 |
| ENSG00000261087 | ENSG00000059145 | 0.526155311 | 4.76E-11 |
| ENSG00000261087 | ENSG00000160305 | 0.573761538 | 2.85E-13 |
| ENSG00000261087 | ENSG00000136828 | 0.558938222 | 1.53E-12 |
| ENSG00000261087 | ENSG00000108352 | 0.624144645 | 4.74E-16 |
| ENSG00000261087 | ENSG00000078687 | 0.59599491 | 1.94E-14 |
| ENSG00000261087 | ENSG00000134698 | 0.540789196 | 1.07E-11 |
| ENSG00000261087 | ENSG00000163516 | 0.597481188 | 1.61E-14 |
| ENSG00000261087 | ENSG00000197217 | 0.518043678 | 1.05E-10 |
| ENSG00000261087 | ENSG00000169914 | 0.541876942 | 9.58E-12 |
| ENSG00000261087 | ENSG00000130338 | 0.563211612 | 9.50E-13 |
| ENSG00000261087 | ENSG00000188981 | 0.540660767 | 1.09E-11 |
| ENSG00000261087 | ENSG00000115977 | 0.550060308 | 4.03E-12 |
| ENSG00000261087 | ENSG00000061936 | 0.570922924 | 3.95E-13 |
| ENSG00000261087 | ENSG00000112787 | 0.54143339 | 1.00E-11 |
| ENSG00000261087 | ENSG00000164236 | 0.50010601 | 5.68E-10 |
| ENSG00000261087 | ENSG00000159433 | 0.514000832 | 1.55E-10 |
| ENSG00000261087 | ENSG00000108306 | 0.511733863 | 1.93E-10 |
| ENSG00000261087 | ENSG00000196391 | 0.521749904 | 7.34E-11 |
| ENSG00000261087 | ENSG00000088854 | 0.521363643 | 7.63E-11 |
| ENSG00000261087 | ENSG00000131018 | 0.511057831 | 2.05E-10 |
| ENSG00000261087 | ENSG00000077157 | 0.527936846 | 3.98E-11 |
| ENSG00000261087 | ENSG00000184787 | 0.567579623 | 5.80E-13 |
| ENSG00000261087 | ENSG00000100077 | 0.578480162 | 1.64E-13 |
| ENSG00000261087 | ENSG00000274211 | 0.533556216 | 2.26E-11 |
| ENSG00000261087 | ENSG00000140992 | 0.508408 | 2.64E-10 |
| ENSG00000261087 | ENSG00000173064 | 0.604735025 | 6.37E-15 |
| ENSG00000261286 | ENSG00000059145 | 0.506266955 | 3.22E-10 |
| ENSG00000261286 | ENSG00000075826 | 0.56775977 | 5.68E-13 |
| ENSG00000261286 | ENSG00000172375 | 0.517645649 | 1.09E-10 |
| ENSG00000261286 | ENSG00000140948 | 0.527094241 | 4.33E-11 |
| ENSG00000261286 | ENSG00000108352 | 0.548995575 | 4.51E-12 |
| ENSG00000261340 | ENSG00000158856 | 0.573741949 | 2.85E-13 |
| ENSG00000261340 | ENSG00000116254 | 0.756005299 | 1.97E-26 |
| ENSG00000261340 | ENSG00000114757 | 0.543743801 | 7.88E-12 |
| ENSG00000261340 | ENSG00000154118 | 0.548218568 | 4.90E-12 |
| ENSG00000261340 | ENSG00000123901 | 0.804415548 | 4.21E-32 |
| ENSG00000261340 | ENSG00000004660 | 0.64726285 | 1.68E-17 |
| ENSG00000261340 | ENSG00000107130 | 0.521496637 | 7.53E-11 |
| ENSG00000261340 | ENSG00000147676 | 0.790907675 | 2.27E-30 |
| ENSG00000261340 | ENSG00000102003 | 0.525113775 | 5.27E-11 |
| ENSG00000261340 | ENSG00000171517 | 0.666982411 | 7.66E-19 |
| ENSG00000261340 | ENSG00000123360 | 0.558393489 | 1.62E-12 |
| ENSG00000261340 | ENSG00000100307 | 0.527852716 | 4.02E-11 |
| ENSG00000261340 | ENSG00000171132 | 0.524043113 | 5.86E-11 |
| ENSG00000261340 | ENSG00000171126 | 0.713257224 | 1.99E-22 |
| ENSG00000261340 | ENSG00000120053 | 0.556104858 | 2.09E-12 |
| ENSG00000261340 | ENSG00000060140 | 0.788091693 | 5.02E-30 |
| ENSG00000261340 | ENSG00000127585 | 0.537961512 | 1.44E-11 |
| ENSG00000261340 | ENSG00000132535 | 0.521101718 | 7.82E-11 |
| ENSG00000261340 | ENSG00000165983 | 0.778844893 | 6.28E-29 |
| ENSG00000261578 | ENSG00000142453 | 0.507070381 | 2.99E-10 |
| ENSG00000261578 | ENSG00000107937 | 0.502729285 | 4.47E-10 |
| ENSG00000261578 | ENSG00000090621 | 0.515454858 | 1.35E-10 |
| ENSG00000261578 | ENSG00000112182 | 0.529154956 | 3.53E-11 |
| ENSG00000261781 | ENSG00000174473 | 0.536474413 | 1.68E-11 |
| ENSG00000261781 | ENSG00000123901 | 0.508777451 | 2.55E-10 |
| ENSG00000261781 | ENSG00000070729 | 0.507464884 | 2.88E-10 |
| ENSG00000261795 | ENSG00000138756 | 0.520986889 | 7.91E-11 |
| ENSG00000261795 | ENSG00000175155 | 0.518033172 | 1.05E-10 |
| ENSG00000261795 | ENSG00000197971 | 0.567286942 | 5.99E-13 |
| ENSG00000261799 | ENSG00000198589 | 0.51779714 | 1.08E-10 |
| ENSG00000261799 | ENSG00000134318 | 0.544765751 | 7.07E-12 |
| ENSG00000261799 | ENSG00000108424 | 0.556136614 | 2.08E-12 |
| ENSG00000261799 | ENSG00000137776 | 0.647384175 | 1.65E-17 |
| ENSG00000261799 | ENSG00000197386 | 0.568294056 | 5.34E-13 |
| ENSG00000261799 | ENSG00000188786 | 0.525468815 | 5.09E-11 |
| ENSG00000261799 | ENSG00000132604 | 0.506494423 | 3.15E-10 |
| ENSG00000261799 | ENSG00000134313 | 0.672054793 | 3.33E-19 |
| ENSG00000261799 | ENSG00000138081 | 0.525550494 | 5.05E-11 |
| ENSG00000261799 | ENSG00000187555 | 0.593742116 | 2.57E-14 |
| ENSG00000261799 | ENSG00000083168 | 0.567526493 | 5.83E-13 |
| ENSG00000261799 | ENSG00000100354 | 0.589950896 | 4.11E-14 |
| ENSG00000261799 | ENSG00000187079 | 0.517607342 | 1.10E-10 |
| ENSG00000261799 | ENSG00000166783 | 0.595053527 | 2.18E-14 |
| ENSG00000261799 | ENSG00000166860 | 0.609472985 | 3.43E-15 |
| ENSG00000261799 | ENSG00000143376 | 0.508376568 | 2.64E-10 |
| ENSG00000261799 | ENSG00000071054 | 0.534035018 | 2.15E-11 |
| ENSG00000261799 | ENSG00000115020 | 0.581840691 | 1.10E-13 |
| ENSG00000261799 | ENSG00000275023 | 0.628558754 | 2.56E-16 |
| ENSG00000261799 | ENSG00000197077 | 0.543008647 | 8.51E-12 |
| ENSG00000261799 | ENSG00000116698 | 0.594123214 | 2.45E-14 |
| ENSG00000261799 | ENSG00000092421 | 0.523376731 | 6.26E-11 |
| ENSG00000261799 | ENSG00000204842 | 0.589361255 | 4.42E-14 |
| ENSG00000261799 | ENSG00000167258 | 0.548686502 | 4.66E-12 |
| ENSG00000261799 | ENSG00000140948 | 0.609593893 | 3.38E-15 |
| ENSG00000261799 | ENSG00000086758 | 0.611529834 | 2.62E-15 |
| ENSG00000261799 | ENSG00000055917 | 0.603767686 | 7.22E-15 |
| ENSG00000261799 | ENSG00000164576 | 0.53063506 | 3.04E-11 |
| ENSG00000261799 | ENSG00000166450 | 0.539153316 | 1.27E-11 |
| ENSG00000261799 | ENSG00000143776 | 0.518620048 | 9.96E-11 |
| ENSG00000261799 | ENSG00000151422 | 0.59851351 | 1.41E-14 |
| ENSG00000261799 | ENSG00000162434 | 0.576899968 | 1.97E-13 |
| ENSG00000261799 | ENSG00000065526 | 0.611494969 | 2.63E-15 |
| ENSG00000261799 | ENSG00000139613 | 0.566968148 | 6.21E-13 |
| ENSG00000261799 | ENSG00000185722 | 0.578380971 | 1.66E-13 |
| ENSG00000261799 | ENSG00000149187 | 0.602060424 | 8.99E-15 |
| ENSG00000261799 | ENSG00000134698 | 0.516892868 | 1.18E-10 |
| ENSG00000261799 | ENSG00000126746 | 0.625988709 | 3.67E-16 |
| ENSG00000261799 | ENSG00000140992 | 0.562930507 | 9.80E-13 |
| ENSG00000261799 | ENSG00000123066 | 0.640176004 | 4.81E-17 |
| ENSG00000261799 | ENSG00000151276 | 0.55590583 | 2.14E-12 |
| ENSG00000261799 | ENSG00000115839 | 0.627375866 | 3.02E-16 |
| ENSG00000261799 | ENSG00000157540 | 0.525087288 | 5.29E-11 |
| ENSG00000261799 | ENSG00000187240 | 0.548438052 | 4.79E-12 |
| ENSG00000261799 | ENSG00000141027 | 0.588254889 | 5.06E-14 |
| ENSG00000261799 | ENSG00000087470 | 0.531368641 | 2.82E-11 |
| ENSG00000261799 | ENSG00000076108 | 0.626006974 | 3.66E-16 |
| ENSG00000261799 | ENSG00000153317 | 0.50085917 | 5.30E-10 |
| ENSG00000261799 | ENSG00000099204 | 0.652785232 | 7.23E-18 |
| ENSG00000261799 | ENSG00000116539 | 0.631920002 | 1.59E-16 |
| ENSG00000261799 | ENSG00000224470 | 0.603128071 | 7.84E-15 |
| ENSG00000261799 | ENSG00000134250 | 0.524445878 | 5.63E-11 |
| ENSG00000261799 | ENSG00000184787 | 0.536172513 | 1.73E-11 |
| ENSG00000261799 | ENSG00000131844 | 0.501398433 | 5.05E-10 |
| ENSG00000261799 | ENSG00000196914 | 0.63083647 | 1.85E-16 |
| ENSG00000261799 | ENSG00000118689 | 0.537183362 | 1.56E-11 |
| ENSG00000261799 | ENSG00000103657 | 0.584364678 | 8.11E-14 |
| ENSG00000261799 | ENSG00000164219 | 0.582813357 | 9.77E-14 |
| ENSG00000261799 | ENSG00000179295 | 0.610307727 | 3.08E-15 |
| ENSG00000261799 | ENSG00000072364 | 0.551251326 | 3.54E-12 |
| ENSG00000261799 | ENSG00000177303 | 0.599681445 | 1.22E-14 |
| ENSG00000261799 | ENSG00000125686 | 0.625325944 | 4.02E-16 |
| ENSG00000261799 | ENSG00000164190 | 0.679076172 | 1.02E-19 |
| ENSG00000261799 | ENSG00000270647 | 0.618168314 | 1.08E-15 |
| ENSG00000261799 | ENSG00000152102 | 0.632227617 | 1.52E-16 |
| ENSG00000261799 | ENSG00000117713 | 0.624814256 | 4.32E-16 |
| ENSG00000261799 | ENSG00000133812 | 0.502605675 | 4.52E-10 |
| ENSG00000261799 | ENSG00000166164 | 0.551356863 | 3.50E-12 |
| ENSG00000261799 | ENSG00000136715 | 0.525148658 | 5.25E-11 |
| ENSG00000261799 | ENSG00000123104 | 0.521609776 | 7.44E-11 |
| ENSG00000261799 | ENSG00000120948 | 0.626166626 | 3.58E-16 |
| ENSG00000261799 | ENSG00000104067 | 0.526593673 | 4.55E-11 |
| ENSG00000261799 | ENSG00000110066 | 0.525899378 | 4.88E-11 |
| ENSG00000261799 | ENSG00000066739 | 0.564158561 | 8.54E-13 |
| ENSG00000261799 | ENSG00000109756 | 0.596748094 | 1.76E-14 |
| ENSG00000261799 | ENSG00000067248 | 0.559264944 | 1.47E-12 |
| ENSG00000261799 | ENSG00000145555 | 0.593677225 | 2.59E-14 |
| ENSG00000261799 | ENSG00000183826 | 0.544373903 | 7.37E-12 |
| ENSG00000261799 | ENSG00000069275 | 0.545431787 | 6.59E-12 |
| ENSG00000261799 | ENSG00000175662 | 0.504393975 | 3.83E-10 |
| ENSG00000261799 | ENSG00000146963 | 0.515745677 | 1.31E-10 |
| ENSG00000261799 | ENSG00000215421 | 0.597948846 | 1.52E-14 |
| ENSG00000261799 | ENSG00000104517 | 0.539538789 | 1.22E-11 |
| ENSG00000261799 | ENSG00000068784 | 0.527244514 | 4.27E-11 |
| ENSG00000261799 | ENSG00000170653 | 0.630621791 | 1.91E-16 |
| ENSG00000261799 | ENSG00000234616 | 0.536964153 | 1.59E-11 |
| ENSG00000261799 | ENSG00000141068 | 0.549342961 | 4.35E-12 |
| ENSG00000261799 | ENSG00000073910 | 0.574543027 | 2.60E-13 |
| ENSG00000261799 | ENSG00000154114 | 0.533279653 | 2.32E-11 |
| ENSG00000261799 | ENSG00000213380 | 0.500167391 | 5.65E-10 |
| ENSG00000261799 | ENSG00000131018 | 0.510714576 | 2.12E-10 |
| ENSG00000261799 | ENSG00000204569 | 0.562594733 | 1.02E-12 |
| ENSG00000261799 | ENSG00000170921 | 0.593323946 | 2.71E-14 |
| ENSG00000261799 | ENSG00000130779 | 0.548843945 | 4.59E-12 |
| ENSG00000261799 | ENSG00000170456 | 0.62109963 | 7.21E-16 |
| ENSG00000261799 | ENSG00000011021 | 0.53505714 | 1.94E-11 |
| ENSG00000261799 | ENSG00000143603 | 0.578675731 | 1.60E-13 |
| ENSG00000261799 | ENSG00000110851 | 0.503255781 | 4.26E-10 |
| ENSG00000261799 | ENSG00000054793 | 0.505546822 | 3.44E-10 |
| ENSG00000261799 | ENSG00000100580 | 0.519585944 | 9.07E-11 |
| ENSG00000261799 | ENSG00000111676 | 0.651918705 | 8.26E-18 |
| ENSG00000261799 | ENSG00000175216 | 0.52914881 | 3.53E-11 |
| ENSG00000261799 | ENSG00000139197 | 0.578317894 | 1.67E-13 |
| ENSG00000261799 | ENSG00000166716 | 0.577477754 | 1.84E-13 |
| ENSG00000261799 | ENSG00000115568 | 0.556837959 | 1.93E-12 |
| ENSG00000261799 | ENSG00000084676 | 0.663251546 | 1.40E-18 |
| ENSG00000261799 | ENSG00000122299 | 0.547276636 | 5.42E-12 |
| ENSG00000261916 | ENSG00000128191 | 0.543875366 | 7.77E-12 |
| ENSG00000261916 | ENSG00000106479 | 0.505358817 | 3.50E-10 |
| ENSG00000261916 | ENSG00000108963 | 0.580844087 | 1.24E-13 |
| ENSG00000261916 | ENSG00000168067 | 0.507508784 | 2.87E-10 |
| ENSG00000261916 | ENSG00000166783 | 0.513210241 | 1.67E-10 |
| ENSG00000261916 | ENSG00000074755 | 0.537965795 | 1.44E-11 |
| ENSG00000261916 | ENSG00000166436 | 0.536979342 | 1.59E-11 |
| ENSG00000261916 | ENSG00000119638 | 0.534176098 | 2.12E-11 |
| ENSG00000261916 | ENSG00000197283 | 0.57814303 | 1.70E-13 |
| ENSG00000261916 | ENSG00000175662 | 0.521792985 | 7.31E-11 |
| ENSG00000261916 | ENSG00000213983 | 0.594990099 | 2.20E-14 |
| ENSG00000262879 | ENSG00000183337 | 0.529392899 | 3.44E-11 |
| ENSG00000262879 | ENSG00000177728 | 0.510494515 | 2.17E-10 |
| ENSG00000262879 | ENSG00000109756 | 0.580043205 | 1.36E-13 |
| ENSG00000262879 | ENSG00000196535 | 0.529048175 | 3.56E-11 |
| ENSG00000262879 | ENSG00000108306 | 0.569911265 | 4.44E-13 |
| ENSG00000262879 | ENSG00000224470 | 0.521204442 | 7.74E-11 |
| ENSG00000262879 | ENSG00000163939 | 0.541042394 | 1.05E-11 |
| ENSG00000262879 | ENSG00000085721 | 0.542681067 | 8.81E-12 |
| ENSG00000262879 | ENSG00000170921 | 0.507091646 | 2.98E-10 |
| ENSG00000262879 | ENSG00000274211 | 0.612799565 | 2.21E-15 |
| ENSG00000262879 | ENSG00000140718 | 0.515793412 | 1.31E-10 |
| ENSG00000262879 | ENSG00000180357 | 0.52965958 | 3.35E-11 |
| ENSG00000262879 | ENSG00000187189 | 0.5162559 | 1.25E-10 |
| ENSG00000262879 | ENSG00000185722 | 0.545431628 | 6.59E-12 |
| ENSG00000262879 | ENSG00000215790 | 0.543828505 | 7.81E-12 |
| ENSG00000262879 | ENSG00000139718 | 0.526897183 | 4.42E-11 |
| ENSG00000262879 | ENSG00000130227 | 0.540402716 | 1.12E-11 |
| ENSG00000262879 | ENSG00000266173 | 0.504197805 | 3.90E-10 |
| ENSG00000262879 | ENSG00000086758 | 0.574815216 | 2.52E-13 |
| ENSG00000262879 | ENSG00000125686 | 0.59031744 | 3.93E-14 |
| ENSG00000262879 | ENSG00000188786 | 0.537468204 | 1.51E-11 |
| ENSG00000262879 | ENSG00000129595 | 0.551258837 | 3.54E-12 |
| ENSG00000262879 | ENSG00000112584 | 0.507572495 | 2.85E-10 |
| ENSG00000262879 | ENSG00000135913 | 0.514247806 | 1.52E-10 |
| ENSG00000262879 | ENSG00000257093 | 0.55089693 | 3.68E-12 |
| ENSG00000262879 | ENSG00000113194 | 0.505433938 | 3.48E-10 |
| ENSG00000262879 | ENSG00000197386 | 0.530078493 | 3.21E-11 |
| ENSG00000262879 | ENSG00000048028 | 0.502260862 | 4.66E-10 |
| ENSG00000262879 | ENSG00000164168 | 0.59304493 | 2.80E-14 |
| ENSG00000262879 | ENSG00000123066 | 0.564348429 | 8.36E-13 |
| ENSG00000262879 | ENSG00000198198 | 0.504345247 | 3.85E-10 |
| ENSG00000262879 | ENSG00000166860 | 0.506389407 | 3.18E-10 |
| ENSG00000262879 | ENSG00000008869 | 0.555930217 | 2.13E-12 |
| ENSG00000262879 | ENSG00000083168 | 0.527673683 | 4.09E-11 |
| ENSG00000262879 | ENSG00000138081 | 0.558568101 | 1.59E-12 |
| ENSG00000262879 | ENSG00000122966 | 0.53167506 | 2.74E-11 |
| ENSG00000262879 | ENSG00000170145 | 0.538011172 | 1.43E-11 |
| ENSG00000262903 | ENSG00000166783 | 0.51280955 | 1.74E-10 |
| ENSG00000262903 | ENSG00000175662 | 0.514854533 | 1.43E-10 |
| ENSG00000262903 | ENSG00000179335 | 0.540454965 | 1.11E-11 |
| ENSG00000262903 | ENSG00000170037 | 0.531497397 | 2.78E-11 |
| ENSG00000262903 | ENSG00000160299 | 0.618836702 | 9.82E-16 |
| ENSG00000262903 | ENSG00000165915 | 0.51734523 | 1.13E-10 |
| ENSG00000262903 | ENSG00000198198 | 0.616533906 | 1.34E-15 |
| ENSG00000262903 | ENSG00000162461 | 0.613906176 | 1.91E-15 |
| ENSG00000262903 | ENSG00000166436 | 0.773114962 | 2.83E-28 |
| ENSG00000262903 | ENSG00000066427 | 0.590579918 | 3.80E-14 |
| ENSG00000262903 | ENSG00000197363 | 0.507050424 | 2.99E-10 |
| ENSG00000262903 | ENSG00000235194 | 0.606781679 | 4.88E-15 |
| ENSG00000262903 | ENSG00000143624 | 0.577819057 | 1.77E-13 |
| ENSG00000262903 | ENSG00000185163 | 0.536639378 | 1.65E-11 |
| ENSG00000262903 | ENSG00000176953 | 0.607865536 | 4.24E-15 |
| ENSG00000262903 | ENSG00000183495 | 0.546386069 | 5.96E-12 |
| ENSG00000262903 | ENSG00000214655 | 0.614476904 | 1.77E-15 |
| ENSG00000262903 | ENSG00000197283 | 0.611026623 | 2.80E-15 |
| ENSG00000262903 | ENSG00000104728 | 0.512134921 | 1.85E-10 |
| ENSG00000262903 | ENSG00000146826 | 0.534973241 | 1.96E-11 |
| ENSG00000262903 | ENSG00000197217 | 0.503549885 | 4.14E-10 |
| ENSG00000262903 | ENSG00000144589 | 0.607433773 | 4.49E-15 |
| ENSG00000262903 | ENSG00000266173 | 0.552306189 | 3.16E-12 |
| ENSG00000263069 | ENSG00000100354 | 0.504056248 | 3.95E-10 |
| ENSG00000263069 | ENSG00000184677 | 0.634788287 | 1.05E-16 |
| ENSG00000263069 | ENSG00000168488 | 0.610114907 | 3.16E-15 |
| ENSG00000263069 | ENSG00000160305 | 0.679935364 | 8.85E-20 |
| ENSG00000263069 | ENSG00000108352 | 0.57562163 | 2.29E-13 |
| ENSG00000263069 | ENSG00000197323 | 0.536881869 | 1.61E-11 |
| ENSG00000263069 | ENSG00000215790 | 0.532729673 | 2.46E-11 |
| ENSG00000263069 | ENSG00000170921 | 0.599173191 | 1.30E-14 |
| ENSG00000263069 | ENSG00000070047 | 0.50152224 | 4.99E-10 |
| ENSG00000263069 | ENSG00000070610 | 0.537184431 | 1.56E-11 |
| ENSG00000263069 | ENSG00000187605 | 0.539353545 | 1.25E-11 |
| ENSG00000263069 | ENSG00000198920 | 0.603577225 | 7.40E-15 |
| ENSG00000263069 | ENSG00000038358 | 0.541019233 | 1.05E-11 |
| ENSG00000263069 | ENSG00000133226 | 0.611370421 | 2.67E-15 |
| ENSG00000263069 | ENSG00000178188 | 0.677962357 | 1.24E-19 |
| ENSG00000263069 | ENSG00000152520 | 0.547102062 | 5.52E-12 |
| ENSG00000263069 | ENSG00000048028 | 0.500122846 | 5.67E-10 |
| ENSG00000263069 | ENSG00000164164 | 0.501448324 | 5.02E-10 |
| ENSG00000263069 | ENSG00000089280 | 0.519508271 | 9.14E-11 |
| ENSG00000263069 | ENSG00000274211 | 0.593379009 | 2.69E-14 |
| ENSG00000263069 | ENSG00000102858 | 0.540015471 | 1.16E-11 |
| ENSG00000263069 | ENSG00000144589 | 0.610269412 | 3.09E-15 |
| ENSG00000263069 | ENSG00000168763 | 0.583979185 | 8.49E-14 |
| ENSG00000263069 | ENSG00000176915 | 0.500325916 | 5.57E-10 |
| ENSG00000263069 | ENSG00000075292 | 0.532751131 | 2.45E-11 |
| ENSG00000263069 | ENSG00000143630 | 0.544646992 | 7.16E-12 |
| ENSG00000263843 | ENSG00000078687 | 0.52415973 | 5.79E-11 |
| ENSG00000264853 | ENSG00000075290 | 0.524523451 | 5.59E-11 |
| ENSG00000264853 | ENSG00000185002 | 0.550216901 | 3.96E-12 |
| ENSG00000265148 | ENSG00000116299 | 0.539601821 | 1.21E-11 |
| ENSG00000265148 | ENSG00000187726 | 0.553234662 | 2.86E-12 |
| ENSG00000265148 | ENSG00000146221 | 0.564285874 | 8.42E-13 |
| ENSG00000265393 | ENSG00000014164 | 0.526263463 | 4.70E-11 |
| ENSG00000265393 | ENSG00000132740 | 0.533679008 | 2.23E-11 |
| ENSG00000265393 | ENSG00000158106 | 0.517231314 | 1.14E-10 |
| ENSG00000266844 | ENSG00000169247 | 0.500446468 | 5.51E-10 |
| ENSG00000266844 | ENSG00000163833 | 0.632215783 | 1.52E-16 |
| ENSG00000267034 | ENSG00000154914 | 0.50697076 | 3.02E-10 |
| ENSG00000267532 | ENSG00000120727 | 0.508398907 | 2.64E-10 |
| ENSG00000268549 | ENSG00000196233 | 0.526789674 | 4.47E-11 |
| ENSG00000269821 | ENSG00000124157 | 0.579618763 | 1.43E-13 |
| ENSG00000269821 | ENSG00000215454 | 0.643053424 | 3.15E-17 |
| ENSG00000269821 | ENSG00000113905 | 0.580289559 | 1.32E-13 |
| ENSG00000269821 | ENSG00000180210 | 0.533190717 | 2.35E-11 |
| ENSG00000269821 | ENSG00000100354 | 0.504841962 | 3.68E-10 |
| ENSG00000269821 | ENSG00000173572 | 0.565565213 | 7.29E-13 |
| ENSG00000269821 | ENSG00000278505 | 0.517785453 | 1.08E-10 |
| ENSG00000269821 | ENSG00000187862 | 0.505231739 | 3.54E-10 |
| ENSG00000269821 | ENSG00000054796 | 0.627080399 | 3.15E-16 |
| ENSG00000269821 | ENSG00000131126 | 0.617028646 | 1.25E-15 |
| ENSG00000269821 | ENSG00000117834 | 0.664051234 | 1.23E-18 |
| ENSG00000269821 | ENSG00000159433 | 0.507903237 | 2.76E-10 |
| ENSG00000269821 | ENSG00000185002 | 0.612634452 | 2.26E-15 |
| ENSG00000269821 | ENSG00000137731 | 0.535743603 | 1.81E-11 |
| ENSG00000269945 | ENSG00000107130 | 0.549289397 | 4.37E-12 |
| ENSG00000269945 | ENSG00000107758 | 0.577225258 | 1.90E-13 |
| ENSG00000269945 | ENSG00000111249 | 0.738103121 | 1.17E-24 |
| ENSG00000269945 | ENSG00000140600 | 0.562247994 | 1.06E-12 |
| ENSG00000271204 | ENSG00000113905 | 0.561543123 | 1.14E-12 |
| ENSG00000271204 | ENSG00000185002 | 0.523357902 | 6.27E-11 |
| ENSG00000271754 | ENSG00000075292 | 0.502433647 | 4.59E-10 |
| ENSG00000271754 | ENSG00000132740 | 0.500991038 | 5.24E-10 |
| ENSG00000271754 | ENSG00000116698 | 0.504910543 | 3.65E-10 |
| ENSG00000271754 | ENSG00000120948 | 0.500483379 | 5.49E-10 |
| ENSG00000271754 | ENSG00000149187 | 0.511723503 | 1.93E-10 |
| ENSG00000271892 | ENSG00000136854 | 0.601090184 | 1.02E-14 |
| ENSG00000271892 | ENSG00000157152 | 0.667951131 | 6.54E-19 |
| ENSG00000271892 | ENSG00000107758 | 0.557682938 | 1.76E-12 |
| ENSG00000271892 | ENSG00000176788 | 0.526015996 | 4.82E-11 |
| ENSG00000271892 | ENSG00000135750 | 0.630981307 | 1.82E-16 |
| ENSG00000271892 | ENSG00000119946 | 0.689042162 | 1.81E-20 |
| ENSG00000271892 | ENSG00000154146 | 0.611155133 | 2.75E-15 |
| ENSG00000271892 | ENSG00000196189 | 0.502699699 | 4.48E-10 |
| ENSG00000271892 | ENSG00000166206 | 0.5234343 | 6.22E-11 |
| ENSG00000271892 | ENSG00000153933 | 0.525631326 | 5.01E-11 |
| ENSG00000271892 | ENSG00000138944 | 0.660412466 | 2.20E-18 |
| ENSG00000271892 | ENSG00000117016 | 0.537666214 | 1.48E-11 |
| ENSG00000271892 | ENSG00000100285 | 0.611657194 | 2.57E-15 |
| ENSG00000271892 | ENSG00000171132 | 0.527114991 | 4.32E-11 |
| ENSG00000271892 | ENSG00000069424 | 0.597993673 | 1.51E-14 |
| ENSG00000271936 | ENSG00000149136 | 0.509035533 | 2.49E-10 |
| ENSG00000271936 | ENSG00000115207 | 0.525305325 | 5.17E-11 |
| ENSG00000271936 | ENSG00000112182 | 0.522649125 | 6.72E-11 |
| ENSG00000271936 | ENSG00000106144 | 0.524481834 | 5.61E-11 |
| ENSG00000271936 | ENSG00000089505 | 0.506053439 | 3.28E-10 |
| ENSG00000271936 | ENSG00000136715 | 0.545853175 | 6.31E-12 |
| ENSG00000271936 | ENSG00000172534 | 0.53001248 | 3.23E-11 |
| ENSG00000271936 | ENSG00000168488 | 0.550310705 | 3.92E-12 |
| ENSG00000271936 | ENSG00000072501 | 0.510866607 | 2.09E-10 |
| ENSG00000271936 | ENSG00000080345 | 0.556147687 | 2.08E-12 |
| ENSG00000271936 | ENSG00000139718 | 0.50177273 | 4.88E-10 |
| ENSG00000271936 | ENSG00000160094 | 0.557408081 | 1.81E-12 |
| ENSG00000271936 | ENSG00000149262 | 0.511846326 | 1.91E-10 |
| ENSG00000271936 | ENSG00000166860 | 0.513054186 | 1.70E-10 |
| ENSG00000272163 | ENSG00000181418 | 0.58154208 | 1.14E-13 |
| ENSG00000272163 | ENSG00000104722 | 0.735035374 | 2.27E-24 |
| ENSG00000272163 | ENSG00000174473 | 0.610711485 | 2.92E-15 |
| ENSG00000272163 | ENSG00000151892 | 0.523502509 | 6.18E-11 |
| ENSG00000272163 | ENSG00000165983 | 0.611639406 | 2.58E-15 |
| ENSG00000272163 | ENSG00000171517 | 0.571352898 | 3.76E-13 |
| ENSG00000272163 | ENSG00000164506 | 0.520053869 | 8.66E-11 |
| ENSG00000272163 | ENSG00000120053 | 0.537701492 | 1.48E-11 |
| ENSG00000272163 | ENSG00000116254 | 0.547382553 | 5.36E-12 |
| ENSG00000272163 | ENSG00000006740 | 0.701769368 | 1.79E-21 |
| ENSG00000272163 | ENSG00000166206 | 0.537613773 | 1.49E-11 |
| ENSG00000272163 | ENSG00000123901 | 0.676527985 | 1.58E-19 |
| ENSG00000272163 | ENSG00000120903 | 0.519141167 | 9.47E-11 |
| ENSG00000272323 | ENSG00000185250 | 0.67007611 | 4.62E-19 |
| ENSG00000272323 | ENSG00000116918 | 0.502078415 | 4.74E-10 |
| ENSG00000272323 | ENSG00000158985 | 0.529666907 | 3.35E-11 |
| ENSG00000272323 | ENSG00000120727 | 0.566082318 | 6.87E-13 |
| ENSG00000272323 | ENSG00000184154 | 0.722357264 | 3.23E-23 |
| ENSG00000272323 | ENSG00000110536 | 0.59264944 | 2.94E-14 |
| ENSG00000272323 | ENSG00000013561 | 0.535358713 | 1.88E-11 |
| ENSG00000272323 | ENSG00000168679 | 0.507249013 | 2.94E-10 |
| ENSG00000272323 | ENSG00000149483 | 0.517552306 | 1.10E-10 |
| ENSG00000272323 | ENSG00000176714 | 0.625742582 | 3.80E-16 |
| ENSG00000272341 | ENSG00000187742 | 0.522811331 | 6.62E-11 |
| ENSG00000272420 | ENSG00000127419 | 0.500099447 | 5.68E-10 |
| ENSG00000272515 | ENSG00000123901 | 0.57204609 | 3.47E-13 |
| ENSG00000272515 | ENSG00000149575 | 0.508958591 | 2.50E-10 |
| ENSG00000272620 | ENSG00000130338 | 0.525425956 | 5.11E-11 |
| ENSG00000272620 | ENSG00000225614 | 0.509880421 | 2.30E-10 |
| ENSG00000272884 | ENSG00000197558 | 0.61041621 | 3.03E-15 |
| ENSG00000272899 | ENSG00000130338 | 0.552168317 | 3.21E-12 |
| ENSG00000272899 | ENSG00000149187 | 0.518533829 | 1.00E-10 |
| ENSG00000272899 | ENSG00000132846 | 0.507742246 | 2.81E-10 |
| ENSG00000272899 | ENSG00000122966 | 0.514890687 | 1.43E-10 |
| ENSG00000272899 | ENSG00000011021 | 0.520647361 | 8.18E-11 |
| ENSG00000272899 | ENSG00000136828 | 0.532889856 | 2.42E-11 |
| ENSG00000272899 | ENSG00000152102 | 0.5651141 | 7.67E-13 |
| ENSG00000272899 | ENSG00000197283 | 0.535361616 | 1.88E-11 |
| ENSG00000272899 | ENSG00000117713 | 0.517723311 | 1.09E-10 |
| ENSG00000272899 | ENSG00000115568 | 0.547207308 | 5.46E-12 |
| ENSG00000272899 | ENSG00000064419 | 0.554196644 | 2.57E-12 |
| ENSG00000272899 | ENSG00000197386 | 0.546694465 | 5.77E-12 |
| ENSG00000272899 | ENSG00000103150 | 0.502035492 | 4.76E-10 |
| ENSG00000272918 | ENSG00000128596 | 0.641475701 | 3.98E-17 |
| ENSG00000272918 | ENSG00000155744 | 0.57036757 | 4.21E-13 |
| ENSG00000272918 | ENSG00000274211 | 0.540885724 | 1.06E-11 |
| ENSG00000272918 | ENSG00000171132 | 0.578020342 | 1.73E-13 |
| ENSG00000272918 | ENSG00000130338 | 0.550582291 | 3.81E-12 |
| ENSG00000272918 | ENSG00000172915 | 0.525163346 | 5.25E-11 |
| ENSG00000272918 | ENSG00000162728 | 0.543836815 | 7.80E-12 |
| ENSG00000272918 | ENSG00000125814 | 0.515654952 | 1.33E-10 |
| ENSG00000272918 | ENSG00000157764 | 0.52502379 | 5.32E-11 |
| ENSG00000272918 | ENSG00000169554 | 0.603824177 | 7.17E-15 |
| ENSG00000272918 | ENSG00000136895 | 0.563946024 | 8.75E-13 |
| ENSG00000272918 | ENSG00000136854 | 0.552047152 | 3.25E-12 |
| ENSG00000272918 | ENSG00000162664 | 0.506660117 | 3.10E-10 |
| ENSG00000272918 | ENSG00000110066 | 0.578739815 | 1.59E-13 |
| ENSG00000272918 | ENSG00000102606 | 0.580192018 | 1.34E-13 |
| ENSG00000272918 | ENSG00000205269 | 0.574182391 | 2.71E-13 |
| ENSG00000272918 | ENSG00000073910 | 0.557026899 | 1.89E-12 |
| ENSG00000272918 | ENSG00000197323 | 0.511953629 | 1.89E-10 |
| ENSG00000272918 | ENSG00000005810 | 0.614897592 | 1.67E-15 |
| ENSG00000272918 | ENSG00000183049 | 0.500265796 | 5.60E-10 |
| ENSG00000272918 | ENSG00000187742 | 0.535603642 | 1.83E-11 |
| ENSG00000272918 | ENSG00000275023 | 0.520004772 | 8.71E-11 |
| ENSG00000272918 | ENSG00000066739 | 0.505652552 | 3.41E-10 |
| ENSG00000272918 | ENSG00000109756 | 0.512834761 | 1.74E-10 |
| ENSG00000272918 | ENSG00000134698 | 0.58728809 | 5.69E-14 |
| ENSG00000272918 | ENSG00000128594 | 0.623121699 | 5.46E-16 |
| ENSG00000272918 | ENSG00000078687 | 0.632768188 | 1.41E-16 |
| ENSG00000272918 | ENSG00000164061 | 0.648488663 | 1.39E-17 |
| ENSG00000272918 | ENSG00000151276 | 0.555452197 | 2.24E-12 |
| ENSG00000273001 | ENSG00000108352 | 0.517356259 | 1.13E-10 |
| ENSG00000273033 | ENSG00000071054 | 0.54872116 | 4.65E-12 |
| ENSG00000273033 | ENSG00000077235 | 0.589665921 | 4.25E-14 |
| ENSG00000273033 | ENSG00000074755 | 0.726517246 | 1.37E-23 |
| ENSG00000273033 | ENSG00000120451 | 0.520617753 | 8.20E-11 |
| ENSG00000273033 | ENSG00000160216 | 0.544251082 | 7.47E-12 |
| ENSG00000273033 | ENSG00000076108 | 0.62509909 | 4.15E-16 |
| ENSG00000273033 | ENSG00000038532 | 0.600550751 | 1.09E-14 |
| ENSG00000273033 | ENSG00000105429 | 0.508487578 | 2.62E-10 |
| ENSG00000273033 | ENSG00000143376 | 0.511196804 | 2.03E-10 |
| ENSG00000273033 | ENSG00000197226 | 0.554409585 | 2.51E-12 |
| ENSG00000273033 | ENSG00000089847 | 0.538458064 | 1.37E-11 |
| ENSG00000273033 | ENSG00000197283 | 0.538859429 | 1.31E-11 |
| ENSG00000273033 | ENSG00000108510 | 0.527862217 | 4.01E-11 |
| ENSG00000273033 | ENSG00000196535 | 0.608888037 | 3.71E-15 |
| ENSG00000273033 | ENSG00000128191 | 0.540266983 | 1.13E-11 |
| ENSG00000273033 | ENSG00000197323 | 0.517819737 | 1.08E-10 |
| ENSG00000273033 | ENSG00000085733 | 0.535659717 | 1.82E-11 |
| ENSG00000273033 | ENSG00000160305 | 0.62707679 | 3.15E-16 |
| ENSG00000273033 | ENSG00000136828 | 0.526576984 | 4.56E-11 |
| ENSG00000273033 | ENSG00000109756 | 0.59884341 | 1.35E-14 |
| ENSG00000273033 | ENSG00000084710 | 0.525889795 | 4.88E-11 |
| ENSG00000273033 | ENSG00000164068 | 0.61418566 | 1.84E-15 |
| ENSG00000273033 | ENSG00000099204 | 0.538414289 | 1.37E-11 |
| ENSG00000273033 | ENSG00000068654 | 0.568826483 | 5.03E-13 |
| ENSG00000273033 | ENSG00000070614 | 0.627393998 | 3.01E-16 |
| ENSG00000273033 | ENSG00000125686 | 0.567451264 | 5.88E-13 |
| ENSG00000273033 | ENSG00000172534 | 0.641430833 | 4.00E-17 |
| ENSG00000273033 | ENSG00000164576 | 0.505501938 | 3.46E-10 |
| ENSG00000273033 | ENSG00000164190 | 0.584940079 | 7.57E-14 |
| ENSG00000273033 | ENSG00000139718 | 0.664309079 | 1.18E-18 |
| ENSG00000273033 | ENSG00000197217 | 0.524978103 | 5.34E-11 |
| ENSG00000273033 | ENSG00000159363 | 0.510321448 | 2.20E-10 |
| ENSG00000273033 | ENSG00000185722 | 0.552798465 | 3.00E-12 |
| ENSG00000273033 | ENSG00000084676 | 0.543625315 | 7.98E-12 |
| ENSG00000273033 | ENSG00000077157 | 0.53651113 | 1.67E-11 |
| ENSG00000273033 | ENSG00000135365 | 0.552305166 | 3.16E-12 |
| ENSG00000273033 | ENSG00000131018 | 0.578429549 | 1.65E-13 |
| ENSG00000273033 | ENSG00000119638 | 0.520424 | 8.36E-11 |
| ENSG00000273033 | ENSG00000188786 | 0.632306278 | 1.50E-16 |
| ENSG00000273033 | ENSG00000141027 | 0.519284351 | 9.34E-11 |
| ENSG00000273033 | ENSG00000170145 | 0.566824663 | 6.32E-13 |
| ENSG00000273033 | ENSG00000123066 | 0.64000047 | 4.94E-17 |
| ENSG00000273033 | ENSG00000196914 | 0.575373226 | 2.36E-13 |
| ENSG00000273033 | ENSG00000151461 | 0.558151717 | 1.67E-12 |
| ENSG00000273033 | ENSG00000224470 | 0.630718283 | 1.89E-16 |
| ENSG00000273033 | ENSG00000038358 | 0.527335489 | 4.23E-11 |
| ENSG00000273033 | ENSG00000140548 | 0.523790892 | 6.01E-11 |
| ENSG00000273033 | ENSG00000169914 | 0.585908474 | 6.73E-14 |
| ENSG00000273033 | ENSG00000166860 | 0.537511608 | 1.51E-11 |
| ENSG00000273033 | ENSG00000157014 | 0.502729319 | 4.47E-10 |
| ENSG00000273033 | ENSG00000184677 | 0.684378485 | 4.11E-20 |
| ENSG00000273033 | ENSG00000119402 | 0.533733682 | 2.22E-11 |
| ENSG00000273033 | ENSG00000054793 | 0.564125619 | 8.57E-13 |
| ENSG00000273033 | ENSG00000167258 | 0.609209034 | 3.56E-15 |
| ENSG00000273033 | ENSG00000144674 | 0.589196302 | 4.51E-14 |
| ENSG00000273033 | ENSG00000116539 | 0.60772215 | 4.32E-15 |
| ENSG00000273033 | ENSG00000169925 | 0.517190436 | 1.14E-10 |
| ENSG00000273033 | ENSG00000104728 | 0.579783251 | 1.40E-13 |
| ENSG00000273033 | ENSG00000116698 | 0.565875117 | 7.03E-13 |
| ENSG00000273033 | ENSG00000048471 | 0.634072001 | 1.17E-16 |
| ENSG00000273033 | ENSG00000173821 | 0.571825306 | 3.56E-13 |
| ENSG00000273033 | ENSG00000075292 | 0.597734086 | 1.56E-14 |
| ENSG00000273033 | ENSG00000154767 | 0.607468903 | 4.47E-15 |
| ENSG00000273033 | ENSG00000115977 | 0.627058218 | 3.16E-16 |
| ENSG00000273033 | ENSG00000138246 | 0.555022966 | 2.35E-12 |
| ENSG00000273033 | ENSG00000061936 | 0.572254145 | 3.39E-13 |
| ENSG00000273033 | ENSG00000101350 | 0.566023343 | 6.92E-13 |
| ENSG00000273033 | ENSG00000147133 | 0.598537869 | 1.41E-14 |
| ENSG00000273033 | ENSG00000163625 | 0.56051579 | 1.28E-12 |
| ENSG00000273033 | ENSG00000197312 | 0.60932072 | 3.50E-15 |
| ENSG00000273033 | ENSG00000181222 | 0.581390593 | 1.16E-13 |
| ENSG00000273033 | ENSG00000115568 | 0.627833391 | 2.83E-16 |
| ENSG00000273033 | ENSG00000134698 | 0.61915795 | 9.40E-16 |
| ENSG00000273033 | ENSG00000072364 | 0.559854813 | 1.38E-12 |
| ENSG00000273033 | ENSG00000103222 | 0.57748284 | 1.84E-13 |
| ENSG00000273033 | ENSG00000143776 | 0.508954814 | 2.50E-10 |
| ENSG00000273033 | ENSG00000270647 | 0.548818073 | 4.60E-12 |
| ENSG00000273033 | ENSG00000140948 | 0.612461262 | 2.31E-15 |
| ENSG00000273033 | ENSG00000100580 | 0.555378174 | 2.26E-12 |
| ENSG00000273033 | ENSG00000158711 | 0.545786692 | 6.35E-12 |
| ENSG00000273033 | ENSG00000086758 | 0.691357163 | 1.20E-20 |
| ENSG00000273033 | ENSG00000039523 | 0.508118847 | 2.71E-10 |
| ENSG00000273033 | ENSG00000264522 | 0.526955696 | 4.39E-11 |
| ENSG00000273033 | ENSG00000146587 | 0.551744972 | 3.36E-12 |
| ENSG00000273033 | ENSG00000151422 | 0.575419777 | 2.35E-13 |
| ENSG00000273033 | ENSG00000008083 | 0.51349111 | 1.63E-10 |
| ENSG00000273033 | ENSG00000174231 | 0.613721732 | 1.96E-15 |
| ENSG00000273033 | ENSG00000166783 | 0.654090171 | 5.91E-18 |
| ENSG00000273033 | ENSG00000162104 | 0.577730453 | 1.79E-13 |
| ENSG00000273033 | ENSG00000180357 | 0.539320789 | 1.25E-11 |
| ENSG00000273033 | ENSG00000047849 | 0.506219979 | 3.23E-10 |
| ENSG00000273033 | ENSG00000138002 | 0.623962924 | 4.86E-16 |
| ENSG00000273033 | ENSG00000076513 | 0.525418361 | 5.12E-11 |
| ENSG00000273033 | ENSG00000141034 | 0.595938287 | 1.95E-14 |
| ENSG00000273033 | ENSG00000125447 | 0.623565111 | 5.13E-16 |
| ENSG00000273033 | ENSG00000102921 | 0.529263116 | 3.49E-11 |
| ENSG00000273033 | ENSG00000057935 | 0.557254722 | 1.84E-12 |
| ENSG00000273033 | ENSG00000175115 | 0.501480231 | 5.01E-10 |
| ENSG00000273033 | ENSG00000166197 | 0.502013708 | 4.77E-10 |
| ENSG00000273033 | ENSG00000083799 | 0.538015683 | 1.43E-11 |
| ENSG00000273033 | ENSG00000082641 | 0.523078645 | 6.45E-11 |
| ENSG00000273033 | ENSG00000100354 | 0.517050992 | 1.16E-10 |
| ENSG00000273033 | ENSG00000134250 | 0.548230963 | 4.90E-12 |
| ENSG00000273033 | ENSG00000171634 | 0.681436861 | 6.84E-20 |
| ENSG00000273033 | ENSG00000157796 | 0.520424356 | 8.36E-11 |
| ENSG00000273033 | ENSG00000141068 | 0.553797259 | 2.69E-12 |
| ENSG00000273033 | ENSG00000225190 | 0.604563745 | 6.51E-15 |
| ENSG00000273033 | ENSG00000170242 | 0.514005996 | 1.55E-10 |
| ENSG00000273033 | ENSG00000167291 | 0.536388175 | 1.69E-11 |
| ENSG00000273033 | ENSG00000152223 | 0.647548675 | 1.61E-17 |
| ENSG00000273033 | ENSG00000066739 | 0.591989701 | 3.20E-14 |
| ENSG00000273033 | ENSG00000124198 | 0.540737368 | 1.08E-11 |
| ENSG00000273033 | ENSG00000065526 | 0.608601311 | 3.85E-15 |
| ENSG00000273033 | ENSG00000147799 | 0.507038401 | 3.00E-10 |
| ENSG00000273033 | ENSG00000205726 | 0.546905639 | 5.64E-12 |
| ENSG00000273033 | ENSG00000185818 | 0.51900582 | 9.59E-11 |
| ENSG00000273033 | ENSG00000047578 | 0.613286453 | 2.07E-15 |
| ENSG00000273033 | ENSG00000130338 | 0.552752585 | 3.01E-12 |
| ENSG00000273033 | ENSG00000129595 | 0.503677219 | 4.09E-10 |
| ENSG00000273033 | ENSG00000160633 | 0.517862346 | 1.07E-10 |
| ENSG00000273033 | ENSG00000184787 | 0.558899675 | 1.54E-12 |
| ENSG00000273033 | ENSG00000117713 | 0.612148045 | 2.41E-15 |
| ENSG00000273033 | ENSG00000170921 | 0.613523871 | 2.01E-15 |
| ENSG00000273033 | ENSG00000064999 | 0.602892023 | 8.08E-15 |
| ENSG00000273033 | ENSG00000110237 | 0.541906645 | 9.55E-12 |
| ENSG00000273033 | ENSG00000166135 | 0.604687754 | 6.41E-15 |
| ENSG00000273033 | ENSG00000153201 | 0.569945712 | 4.42E-13 |
| ENSG00000273033 | ENSG00000163125 | 0.557180336 | 1.86E-12 |
| ENSG00000273033 | ENSG00000124120 | 0.501461135 | 5.02E-10 |
| ENSG00000273033 | ENSG00000115839 | 0.531010778 | 2.93E-11 |
| ENSG00000273033 | ENSG00000183495 | 0.656010217 | 4.39E-18 |
| ENSG00000273033 | ENSG00000084112 | 0.537256842 | 1.55E-11 |
| ENSG00000273033 | ENSG00000152102 | 0.656341044 | 4.17E-18 |
| ENSG00000273033 | ENSG00000177169 | 0.60333529 | 7.63E-15 |
| ENSG00000273033 | ENSG00000180902 | 0.582571655 | 1.01E-13 |
| ENSG00000273036 | ENSG00000140600 | 0.701870457 | 1.76E-21 |
| ENSG00000273036 | ENSG00000177238 | 0.64473832 | 2.45E-17 |
| ENSG00000273036 | ENSG00000197971 | 0.644257691 | 2.63E-17 |
| ENSG00000273036 | ENSG00000135750 | 0.504619612 | 3.75E-10 |
| ENSG00000273036 | ENSG00000141524 | 0.524552757 | 5.57E-11 |
| ENSG00000273036 | ENSG00000115365 | 0.574726664 | 2.54E-13 |
| ENSG00000273096 | ENSG00000038532 | 0.526993822 | 4.38E-11 |
| ENSG00000273096 | ENSG00000077044 | 0.600055353 | 1.16E-14 |
| ENSG00000273096 | ENSG00000172932 | 0.54020231 | 1.14E-11 |
| ENSG00000273096 | ENSG00000100354 | 0.563304106 | 9.40E-13 |
| ENSG00000273096 | ENSG00000173064 | 0.566652191 | 6.44E-13 |
| ENSG00000273096 | ENSG00000167971 | 0.510045532 | 2.26E-10 |
| ENSG00000273096 | ENSG00000163945 | 0.589106281 | 4.56E-14 |
| ENSG00000273096 | ENSG00000132535 | 0.51732312 | 1.13E-10 |
| ENSG00000273096 | ENSG00000166833 | 0.525327945 | 5.16E-11 |
| ENSG00000273096 | ENSG00000065526 | 0.512213424 | 1.84E-10 |
| ENSG00000273096 | ENSG00000108963 | 0.523558116 | 6.15E-11 |
| ENSG00000273096 | ENSG00000169914 | 0.607539649 | 4.42E-15 |
| ENSG00000273096 | ENSG00000010322 | 0.567701033 | 5.72E-13 |
| ENSG00000273096 | ENSG00000266173 | 0.527124831 | 4.32E-11 |
| ENSG00000273096 | ENSG00000187605 | 0.525617561 | 5.02E-11 |
| ENSG00000273096 | ENSG00000177169 | 0.559392145 | 1.45E-12 |
| ENSG00000273096 | ENSG00000170004 | 0.511659322 | 1.94E-10 |
| ENSG00000273096 | ENSG00000132740 | 0.506785732 | 3.07E-10 |
| ENSG00000273096 | ENSG00000074755 | 0.586597844 | 6.19E-14 |
| ENSG00000273096 | ENSG00000134698 | 0.588953914 | 4.64E-14 |
| ENSG00000273096 | ENSG00000275023 | 0.50980263 | 2.31E-10 |
| ENSG00000273151 | ENSG00000143624 | 0.687195792 | 2.51E-20 |
| ENSG00000273151 | ENSG00000166436 | 0.632878927 | 1.39E-16 |
| ENSG00000273151 | ENSG00000204659 | 0.629238958 | 2.32E-16 |
| ENSG00000273151 | ENSG00000162461 | 0.573711846 | 2.86E-13 |
| ENSG00000273151 | ENSG00000160294 | 0.634018276 | 1.18E-16 |
| ENSG00000273151 | ENSG00000215790 | 0.580377064 | 1.31E-13 |
| ENSG00000273151 | ENSG00000112739 | 0.523878498 | 5.96E-11 |
| ENSG00000273151 | ENSG00000176953 | 0.625146859 | 4.12E-16 |
| ENSG00000273151 | ENSG00000166135 | 0.50037421 | 5.54E-10 |
| ENSG00000273230 | ENSG00000179832 | 0.508899123 | 2.52E-10 |
| ENSG00000273230 | ENSG00000160216 | 0.561328587 | 1.17E-12 |
| ENSG00000273230 | ENSG00000152102 | 0.597695092 | 1.57E-14 |
| ENSG00000273230 | ENSG00000070614 | 0.547865721 | 5.09E-12 |
| ENSG00000273230 | ENSG00000160703 | 0.562465738 | 1.03E-12 |
| ENSG00000273230 | ENSG00000135924 | 0.511379709 | 1.99E-10 |
| ENSG00000273230 | ENSG00000224470 | 0.575058566 | 2.45E-13 |
| ENSG00000273230 | ENSG00000077044 | 0.517254044 | 1.14E-10 |
| ENSG00000273230 | ENSG00000186350 | 0.646503453 | 1.88E-17 |
| ENSG00000273230 | ENSG00000186635 | 0.521140142 | 7.79E-11 |
| ENSG00000273230 | ENSG00000077235 | 0.576351031 | 2.10E-13 |
| ENSG00000273230 | ENSG00000106263 | 0.631479003 | 1.69E-16 |
| ENSG00000273230 | ENSG00000184677 | 0.531207313 | 2.87E-11 |
| ENSG00000273230 | ENSG00000149115 | 0.631109615 | 1.78E-16 |
| ENSG00000273230 | ENSG00000114867 | 0.577546991 | 1.83E-13 |
| ENSG00000273230 | ENSG00000148356 | 0.526576749 | 4.56E-11 |
| ENSG00000273230 | ENSG00000142751 | 0.509085314 | 2.47E-10 |
| ENSG00000273230 | ENSG00000168056 | 0.533911944 | 2.18E-11 |
| ENSG00000273230 | ENSG00000086758 | 0.608641521 | 3.83E-15 |
| ENSG00000273230 | ENSG00000154767 | 0.607659699 | 4.36E-15 |
| ENSG00000273230 | ENSG00000182095 | 0.653934696 | 6.06E-18 |
| ENSG00000273230 | ENSG00000170265 | 0.644833626 | 2.41E-17 |
| ENSG00000273230 | ENSG00000104728 | 0.572852957 | 3.16E-13 |
| ENSG00000273230 | ENSG00000169871 | 0.729943824 | 6.72E-24 |
| ENSG00000273230 | ENSG00000126012 | 0.525155568 | 5.25E-11 |
| ENSG00000273230 | ENSG00000166326 | 0.515093805 | 1.40E-10 |
| ENSG00000273230 | ENSG00000119402 | 0.532224777 | 2.59E-11 |
| ENSG00000273230 | ENSG00000155034 | 0.513514251 | 1.63E-10 |
| ENSG00000273230 | ENSG00000166833 | 0.519907283 | 8.79E-11 |
| ENSG00000273230 | ENSG00000108424 | 0.523379945 | 6.26E-11 |
| ENSG00000273230 | ENSG00000186591 | 0.551006083 | 3.64E-12 |
| ENSG00000273230 | ENSG00000148337 | 0.530621071 | 3.04E-11 |
| ENSG00000273230 | ENSG00000065526 | 0.523597695 | 6.12E-11 |
| ENSG00000273230 | ENSG00000167258 | 0.512410168 | 1.81E-10 |
| ENSG00000273230 | ENSG00000142186 | 0.512405731 | 1.81E-10 |
| ENSG00000273230 | ENSG00000133619 | 0.600769424 | 1.06E-14 |
| ENSG00000273230 | ENSG00000134250 | 0.514291754 | 1.51E-10 |
| ENSG00000273230 | ENSG00000274211 | 0.521540336 | 7.49E-11 |
| ENSG00000273230 | ENSG00000164828 | 0.562674416 | 1.01E-12 |
| ENSG00000273451 | ENSG00000170325 | 0.508633055 | 2.58E-10 |
| ENSG00000273451 | ENSG00000141068 | 0.51086465 | 2.09E-10 |
| ENSG00000273487 | ENSG00000196189 | 0.50655709 | 3.13E-10 |
| ENSG00000273487 | ENSG00000100307 | 0.52301397 | 6.49E-11 |
| ENSG00000274292 | ENSG00000184677 | 0.50363642 | 4.11E-10 |
| ENSG00000274292 | ENSG00000133226 | 0.5329843 | 2.40E-11 |
| ENSG00000274292 | ENSG00000108963 | 0.570742325 | 4.04E-13 |
| ENSG00000274340 | ENSG00000166436 | 0.612369842 | 2.34E-15 |
| ENSG00000276649 | ENSG00000135951 | 0.515037585 | 1.41E-10 |
| ENSG00000278156 | ENSG00000108963 | 0.511672125 | 1.94E-10 |
| ENSG00000278156 | ENSG00000197283 | 0.52458454 | 5.56E-11 |
| ENSG00000278156 | ENSG00000008869 | 0.546181244 | 6.09E-12 |
| ENSG00000278156 | ENSG00000136854 | 0.518450843 | 1.01E-10 |
| ENSG00000278156 | ENSG00000066739 | 0.521051528 | 7.86E-11 |
| ENSG00000278156 | ENSG00000155744 | 0.615452103 | 1.55E-15 |
| ENSG00000278156 | ENSG00000158528 | 0.507659675 | 2.83E-10 |
| ENSG00000278156 | ENSG00000152520 | 0.61471969 | 1.71E-15 |
| ENSG00000278156 | ENSG00000100201 | 0.542332081 | 9.14E-12 |
| ENSG00000278156 | ENSG00000100027 | 0.501702051 | 4.91E-10 |
| ENSG00000278156 | ENSG00000196233 | 0.504368507 | 3.84E-10 |
| ENSG00000278156 | ENSG00000073910 | 0.636509744 | 8.22E-17 |
| ENSG00000278156 | ENSG00000275023 | 0.606257624 | 5.23E-15 |
| ENSG00000278156 | ENSG00000125851 | 0.512166502 | 1.85E-10 |
| ENSG00000278156 | ENSG00000172915 | 0.632864234 | 1.39E-16 |
| ENSG00000278156 | ENSG00000088179 | 0.558941929 | 1.53E-12 |
| ENSG00000278156 | ENSG00000134698 | 0.659139595 | 2.69E-18 |
| ENSG00000278156 | ENSG00000110066 | 0.571117965 | 3.86E-13 |
| ENSG00000278156 | ENSG00000122299 | 0.503104495 | 4.32E-10 |
| ENSG00000278156 | ENSG00000160716 | 0.583707656 | 8.78E-14 |
| ENSG00000278156 | ENSG00000166405 | 0.606666568 | 4.96E-15 |
| ENSG00000278156 | ENSG00000028310 | 0.516292533 | 1.25E-10 |
| ENSG00000278291 | ENSG00000148660 | 0.596462208 | 1.83E-14 |
| ENSG00000278291 | ENSG00000036672 | 0.613937523 | 1.90E-15 |
| ENSG00000278291 | ENSG00000168710 | 0.506147608 | 3.26E-10 |
| ENSG00000278772 | ENSG00000036672 | 0.515657807 | 1.33E-10 |
| ENSG00000278772 | ENSG00000117222 | 0.59035469 | 3.91E-14 |
| ENSG00000279006 | ENSG00000123594 | 0.72973984 | 7.01E-24 |
| ENSG00000279338 | ENSG00000170921 | 0.50032537 | 5.57E-10 |
| ENSG00000279338 | ENSG00000103657 | 0.525677745 | 4.99E-11 |
| ENSG00000279338 | ENSG00000160305 | 0.506970144 | 3.02E-10 |
| ENSG00000279338 | ENSG00000100234 | 0.524737565 | 5.47E-11 |
| ENSG00000279338 | ENSG00000100354 | 0.56313423 | 9.58E-13 |
| ENSG00000281195 | ENSG00000103657 | 0.51594697 | 1.29E-10 |
| ENSG00000281195 | ENSG00000100354 | 0.6122474 | 2.38E-15 |
| ENSG00000281195 | ENSG00000169914 | 0.511160202 | 2.03E-10 |
| ENSG00000281195 | ENSG00000104848 | 0.715783322 | 1.21E-22 |
| ENSG00000281195 | ENSG00000187605 | 0.522529404 | 6.80E-11 |
| ENSG00000281195 | ENSG00000196233 | 0.503951137 | 3.99E-10 |
| ENSG00000281195 | ENSG00000160305 | 0.530772855 | 3.00E-11 |
| ENSG00000281796 | ENSG00000130338 | 0.522991935 | 6.50E-11 |
