# Supplementary material for: The regulatory pattern of target gene expression by aberrant enhancer methylation in glioblastoma
Source: BMC Bioinformatics. 2021 Sep 5;22:420. doi: 10.1186/s12859-021-04345-8 (PMC8420065; doi:10.1186/s12859-021-04345-8)
Supplement: Supplementary file 6 — Additional file 6. Table S3. GO terms and KEGG pathways. [file 12859_2021_4345_MOESM6_ESM.docx]

Table S3. GO terms and KEGG pathways

**BP**

| ID | Description | p-value | Count |
| --- | --- | --- | --- |
| GO:0016043 | cellular component organization | 6.85E-17 | 455 |
| GO:0071840 | cellular component organization or biogenesis | 6.96E-17 | 465 |
| GO:0006996 | organelle organization | 2.19E-15 | 300 |
| GO:0051641 | cellular localization | 1.33E-14 | 239 |
| GO:0070647 | protein modification by small protein conjugation or removal | 8.20E-14 | 116 |
| GO:0030030 | cell projection organization | 1.81E-13 | 145 |
| GO:0120036 | plasma membrane bounded cell projection organization | 2.58E-13 | 142 |
| GO:0051128 | regulation of cellular component organization | 5.07E-13 | 207 |
| GO:0007399 | nervous system development | 1.46E-12 | 197 |
| GO:0051649 | establishment of localization in cell | 1.36E-11 | 185 |
| GO:0006464 | cellular protein modification process | 1.52E-11 | 300 |
| GO:0036211 | protein modification process | 1.52E-11 | 300 |
| GO:0044087 | regulation of cellular component biogenesis | 2.53E-11 | 98 |
| GO:0022607 | cellular component assembly | 6.13E-11 | 236 |
| GO:0099504 | synaptic vesicle cycle | 6.37E-11 | 35 |
| GO:0044085 | cellular component biogenesis | 1.93E-10 | 248 |
| GO:0060341 | regulation of cellular localization | 2.00E-10 | 92 |
| GO:0043412 | macromolecule modification | 4.88E-10 | 305 |
| GO:0048699 | generation of neurons | 5.23E-10 | 133 |
| GO:0071108 | protein K48-linked deubiquitination | 6.56E-10 | 14 |
| GO:0050794 | regulation of cellular process | 8.11E-10 | 653 |
| GO:0030031 | cell projection assembly | 9.14E-10 | 65 |
| GO:0120039 | plasma membrane bounded cell projection morphogenesis | 1.14E-09 | 72 |
| GO:0060627 | regulation of vesicle-mediated transport | 1.18E-09 | 62 |
| GO:0030182 | neuron differentiation | 1.39E-09 | 121 |
| GO:0048858 | cell projection morphogenesis | 1.49E-09 | 72 |
| GO:0098693 | regulation of synaptic vesicle cycle | 1.83E-09 | 24 |
| GO:0120031 | plasma membrane bounded cell projection assembly | 2.47E-09 | 63 |
| GO:0022008 | neurogenesis | 2.47E-09 | 137 |
| GO:0044260 | cellular macromolecule metabolic process | 3.75E-09 | 515 |
| GO:0032386 | regulation of intracellular transport | 4.33E-09 | 52 |
| GO:0048666 | neuron development | 4.93E-09 | 102 |
| GO:0032990 | cell part morphogenesis | 4.97E-09 | 72 |
| GO:0048812 | neuron projection morphogenesis | 6.20E-09 | 69 |
| GO:0051179 | localization | 8.55E-09 | 428 |
| GO:0046907 | intracellular transport | 9.50E-09 | 149 |
| GO:0031323 | regulation of cellular metabolic process | 1.24E-08 | 404 |
| GO:0017156 | calcium ion regulated exocytosis | 1.82E-08 | 27 |
| GO:0023051 | regulation of signaling | 2.02E-08 | 252 |
| GO:0033043 | regulation of organelle organization | 2.04E-08 | 110 |
| GO:0008104 | protein localization | 2.74E-08 | 203 |
| GO:0016079 | synaptic vesicle exocytosis | 3.15E-08 | 23 |
| GO:0000902 | cell morphogenesis | 3.50E-08 | 94 |
| GO:0033036 | macromolecule localization | 3.80E-08 | 223 |
| GO:0031175 | neuron projection development | 3.99E-08 | 90 |
| GO:0007267 | cell-cell signaling | 4.07E-08 | 134 |
| GO:0044267 | cellular protein metabolic process | 4.10E-08 | 345 |
| GO:0032446 | protein modification by small protein conjugation | 4.15E-08 | 85 |
| GO:1903305 | regulation of regulated secretory pathway | 4.20E-08 | 27 |
| GO:0017158 | regulation of calcium ion-dependent exocytosis | 4.61E-08 | 22 |
| GO:0016567 | protein ubiquitination | 4.65E-08 | 79 |
| GO:0031344 | regulation of cell projection organization | 4.81E-08 | 69 |
| GO:0010646 | regulation of cell communication | 5.24E-08 | 247 |
| GO:0120035 | regulation of plasma membrane bounded cell projection organization | 6.00E-08 | 68 |
| GO:0097479 | synaptic vesicle localization | 6.26E-08 | 27 |
| GO:0007268 | chemical synaptic transmission | 7.19E-08 | 70 |
| GO:0098916 | anterograde trans-synaptic signaling | 7.19E-08 | 70 |
| GO:0099003 | vesicle-mediated transport in synapse | 7.51E-08 | 31 |
| GO:0048522 | positive regulation of cellular process | 7.96E-08 | 355 |
| GO:0099537 | trans-synaptic signaling | 1.12E-07 | 70 |
| GO:0034613 | cellular protein localization | 1.15E-07 | 146 |
| GO:0032502 | developmental process | 1.54E-07 | 403 |
| GO:0099536 | synaptic signaling | 1.54E-07 | 70 |
| GO:0032989 | cellular component morphogenesis | 1.57E-07 | 99 |
| GO:0070727 | cellular macromolecule localization | 1.65E-07 | 146 |
| GO:0007275 | multicellular organism development | 1.72E-07 | 353 |
| GO:0007017 | microtubule-based process | 1.77E-07 | 72 |
| GO:0048667 | cell morphogenesis involved in neuron differentiation | 2.06E-07 | 60 |
| GO:0051130 | positive regulation of cellular component organization | 2.17E-07 | 106 |
| GO:0048856 | anatomical structure development | 2.43E-07 | 379 |
| GO:0016570 | histone modification | 2.87E-07 | 50 |
| GO:0070925 | organelle assembly | 2.98E-07 | 78 |
| GO:0000904 | cell morphogenesis involved in differentiation | 3.00E-07 | 71 |
| GO:0048468 | cell development | 3.20E-07 | 163 |
| GO:2000300 | regulation of synaptic vesicle exocytosis | 3.24E-07 | 17 |
| GO:0044265 | cellular macromolecule catabolic process | 3.40E-07 | 99 |
| GO:0016579 | protein deubiquitination | 4.57E-07 | 36 |
| GO:0007269 | neurotransmitter secretion | 4.89E-07 | 26 |
| GO:0099643 | signal release from synapse | 5.50E-07 | 26 |
| GO:0006325 | chromatin organization | 5.62E-07 | 74 |
| GO:0051640 | organelle localization | 6.19E-07 | 67 |
| GO:0017157 | regulation of exocytosis | 7.09E-07 | 30 |
| GO:0048489 | synaptic vesicle transport | 8.26E-07 | 24 |
| GO:0097480 | establishment of synaptic vesicle localization | 8.26E-07 | 24 |
| GO:0043632 | modification-dependent macromolecule catabolic process | 8.91E-07 | 62 |
| GO:1902803 | regulation of synaptic vesicle transport | 9.89E-07 | 17 |
| GO:0007010 | cytoskeleton organization | 9.97E-07 | 108 |
| GO:0016569 | covalent chromatin modification | 1.05E-06 | 50 |
| GO:0019941 | modification-dependent protein catabolic process | 1.05E-06 | 61 |
| GO:0016358 | dendrite development | 1.08E-06 | 31 |
| GO:0051234 | establishment of localization | 1.20E-06 | 338 |
| GO:0050804 | modulation of chemical synaptic transmission | 1.20E-06 | 47 |
| GO:0009893 | positive regulation of metabolic process | 1.24E-06 | 245 |
| GO:0099177 | regulation of trans-synaptic signaling | 1.28E-06 | 47 |
| GO:0051276 | chromosome organization | 1.41E-06 | 101 |
| GO:0006511 | ubiquitin-dependent protein catabolic process | 1.62E-06 | 60 |
| GO:0070646 | protein modification by small protein removal | 1.72E-06 | 36 |
| GO:0006810 | transport | 1.81E-06 | 330 |
| GO:0031325 | positive regulation of cellular metabolic process | 2.00E-06 | 226 |
| GO:0065007 | biological regulation | 2.04E-06 | 718 |
| GO:0051129 | negative regulation of cellular component organization | 2.16E-06 | 68 |
| GO:0060271 | cilium assembly | 2.18E-06 | 41 |
| GO:0048731 | system development | 2.65E-06 | 314 |
| GO:0051648 | vesicle localization | 2.95E-06 | 36 |
| GO:0044782 | cilium organization | 3.05E-06 | 42 |
| GO:0048813 | dendrite morphogenesis | 3.64E-06 | 22 |
| GO:0070536 | protein K63-linked deubiquitination | 3.72E-06 | 10 |
| GO:0080090 | regulation of primary metabolic process | 3.90E-06 | 384 |
| GO:0016192 | vesicle-mediated transport | 3.99E-06 | 153 |
| GO:0050789 | regulation of biological process | 4.02E-06 | 683 |
| GO:0060284 | regulation of cell development | 4.64E-06 | 81 |
| GO:0046928 | regulation of neurotransmitter secretion | 5.12E-06 | 18 |
| GO:0051171 | regulation of nitrogen compound metabolic process | 5.20E-06 | 374 |
| GO:0051656 | establishment of organelle localization | 6.04E-06 | 50 |
| GO:0048791 | calcium ion-regulated exocytosis of neurotransmitter | 6.87E-06 | 7 |
| GO:1905114 | cell surface receptor signaling pathway involved in cell-cell signaling | 7.56E-06 | 58 |
| GO:0048518 | positive regulation of biological process | 7.62E-06 | 383 |
| GO:0043254 | regulation of protein complex assembly | 7.84E-06 | 47 |
| GO:0006836 | neurotransmitter transport | 8.20E-06 | 32 |
| GO:0044257 | cellular protein catabolic process | 9.06E-06 | 68 |
| GO:0007018 | microtubule-based movement | 9.11E-06 | 33 |
| GO:0051603 | proteolysis involved in cellular protein catabolic process | 1.01E-05 | 64 |
| GO:0035556 | intracellular signal transduction | 1.07E-05 | 200 |
| GO:0042073 | intraciliary transport | 1.15E-05 | 12 |
| GO:0023061 | signal release | 1.29E-05 | 46 |
| GO:0000226 | microtubule cytoskeleton organization | 1.32E-05 | 52 |
| GO:0010604 | positive regulation of macromolecule metabolic process | 1.32E-05 | 223 |
| GO:0019219 | regulation of nucleobase-containing compound metabolic process | 1.49E-05 | 266 |
| GO:0009057 | macromolecule catabolic process | 1.50E-05 | 107 |
| GO:0051650 | establishment of vesicle localization | 1.52E-05 | 33 |
| GO:0048869 | cellular developmental process | 1.53E-05 | 282 |
| GO:0019538 | protein metabolic process | 1.73E-05 | 366 |
| GO:0030154 | cell differentiation | 1.75E-05 | 271 |
| GO:0051049 | regulation of transport | 1.76E-05 | 136 |
| GO:0050767 | regulation of neurogenesis | 1.97E-05 | 70 |
| GO:0022604 | regulation of cell morphogenesis | 1.99E-05 | 47 |
| GO:0044248 | cellular catabolic process | 2.01E-05 | 162 |
| GO:0048523 | negative regulation of cellular process | 2.02E-05 | 307 |
| GO:1902115 | regulation of organelle assembly | 2.03E-05 | 25 |
| GO:0010975 | regulation of neuron projection development | 2.08E-05 | 48 |
| GO:0051252 | regulation of RNA metabolic process | 2.31E-05 | 247 |
| GO:0035735 | intraciliary transport involved in cilium assembly | 2.46E-05 | 10 |
| GO:0043547 | positive regulation of GTPase activity | 2.57E-05 | 41 |
| GO:0009966 | regulation of signal transduction | 2.60E-05 | 210 |
| GO:0051588 | regulation of neurotransmitter transport | 2.73E-05 | 20 |
| GO:0035640 | exploration behavior | 3.14E-05 | 8 |
| GO:0051960 | regulation of nervous system development | 3.19E-05 | 76 |
| GO:0044093 | positive regulation of molecular function | 3.44E-05 | 128 |
| GO:0006366 | transcription by RNA polymerase II | 3.66E-05 | 189 |
| GO:0030163 | protein catabolic process | 3.69E-05 | 76 |
| GO:0044090 | positive regulation of vacuole organization | 3.74E-05 | 6 |
| GO:0044089 | positive regulation of cellular component biogenesis | 3.84E-05 | 50 |
| GO:0009894 | regulation of catabolic process | 3.84E-05 | 79 |
| GO:0036465 | synaptic vesicle recycling | 4.10E-05 | 13 |
| GO:0006887 | exocytosis | 4.12E-05 | 74 |
| GO:0007264 | small GTPase mediated signal transduction | 4.19E-05 | 52 |
| GO:0061024 | membrane organization | 4.85E-05 | 73 |
| GO:0043085 | positive regulation of catalytic activity | 5.48E-05 | 106 |
| GO:0051056 | regulation of small GTPase mediated signal transduction | 6.23E-05 | 35 |
| GO:0023052 | signaling | 6.75E-05 | 395 |
| GO:0031329 | regulation of cellular catabolic process | 6.97E-05 | 71 |
| GO:0035520 | monoubiquitinated protein deubiquitination | 7.07E-05 | 5 |
| GO:0035871 | protein K11-linked deubiquitination | 7.07E-05 | 5 |
| GO:0032879 | regulation of localization | 7.45E-05 | 189 |
| GO:0010769 | regulation of cell morphogenesis involved in differentiation | 7.71E-05 | 32 |
| GO:2000112 | regulation of cellular macromolecule biosynthetic process | 7.81E-05 | 253 |
| GO:0006906 | vesicle fusion | 8.17E-05 | 15 |
| GO:0031324 | negative regulation of cellular metabolic process | 8.62E-05 | 177 |
| GO:0097659 | nucleic acid-templated transcription | 8.78E-05 | 237 |
| GO:0120032 | regulation of plasma membrane bounded cell projection assembly | 9.07E-05 | 22 |
| GO:0051173 | positive regulation of nitrogen compound metabolic process | 9.23E-05 | 208 |
| GO:0010556 | regulation of macromolecule biosynthetic process | 9.74E-05 | 260 |
| GO:0006351 | transcription, DNA-templated | 9.75E-05 | 234 |
| GO:0007154 | cell communication | 9.96E-05 | 395 |
| GO:0048488 | synaptic vesicle endocytosis | 0.000106989 | 11 |
| GO:0140238 | presynaptic endocytosis | 0.000106989 | 11 |
| GO:0060491 | regulation of cell projection assembly | 0.000107436 | 22 |
| GO:0032774 | RNA biosynthetic process | 0.000117983 | 237 |
| GO:2000786 | positive regulation of autophagosome assembly | 0.000124221 | 5 |
| GO:0045664 | regulation of neuron differentiation | 0.000124732 | 56 |
| GO:0031338 | regulation of vesicle fusion | 0.000126146 | 8 |
| GO:0000209 | protein polyubiquitination | 0.000134148 | 32 |
| GO:0016246 | RNA interference | 0.000134861 | 6 |
| GO:0030705 | cytoskeleton-dependent intracellular transport | 0.000137696 | 22 |
| GO:1903827 | regulation of cellular protein localization | 0.000138859 | 47 |
| GO:0050806 | positive regulation of synaptic transmission | 0.000140901 | 21 |
| GO:0010639 | negative regulation of organelle organization | 0.000146775 | 38 |
| GO:0019222 | regulation of metabolic process | 0.000147225 | 428 |
| GO:0001505 | regulation of neurotransmitter levels | 0.000156215 | 35 |
| GO:0018210 | peptidyl-threonine modification | 0.00016664 | 18 |
| GO:0090174 | organelle membrane fusion | 0.000167218 | 15 |
| GO:0048583 | regulation of response to stimulus | 0.000185769 | 269 |
| GO:0046777 | protein autophosphorylation | 0.000186362 | 26 |
| GO:0048284 | organelle fusion | 0.000195253 | 17 |
| GO:0016188 | synaptic vesicle maturation | 0.000203976 | 5 |
| GO:0051716 | cellular response to stimulus | 0.000210204 | 443 |
| GO:0010807 | regulation of synaptic vesicle priming | 0.000211132 | 4 |
| GO:0071947 | protein deubiquitination involved in ubiquitin-dependent protein catabolic process | 0.000211132 | 4 |
| GO:0032268 | regulation of cellular protein metabolic process | 0.000215365 | 176 |
| GO:0010970 | transport along microtubule | 0.000218881 | 20 |
| GO:0099111 | microtubule-based transport | 0.000218881 | 20 |
| GO:0050684 | regulation of mRNA processing | 0.000220905 | 18 |
| GO:0050773 | regulation of dendrite development | 0.000221764 | 19 |
| GO:0033157 | regulation of intracellular protein transport | 0.000230039 | 25 |
| GO:0006357 | regulation of transcription by RNA polymerase II | 0.000232348 | 176 |
| GO:0007265 | Ras protein signal transduction | 0.000239058 | 41 |
| GO:0048167 | regulation of synaptic plasticity | 0.000239326 | 22 |
| GO:0097581 | lamellipodium organization | 0.000256038 | 13 |
| GO:0006476 | protein deacetylation | 0.000262832 | 14 |
| GO:0050803 | regulation of synapse structure or activity | 0.000263759 | 25 |
| GO:0048519 | negative regulation of biological process | 0.000267533 | 350 |
| GO:1903311 | regulation of mRNA metabolic process | 0.000299471 | 32 |
| GO:0007409 | axonogenesis | 0.000305516 | 42 |
| GO:0045934 | negative regulation of nucleobase-containing compound metabolic process | 0.000310275 | 106 |
| GO:0051172 | negative regulation of nitrogen compound metabolic process | 0.000314873 | 164 |
| GO:0051253 | negative regulation of RNA metabolic process | 0.00033169 | 98 |
| GO:0009056 | catabolic process | 0.000338895 | 171 |
| GO:0060998 | regulation of dendritic spine development | 0.000353215 | 12 |
| GO:0009889 | regulation of biosynthetic process | 0.000362545 | 270 |
| GO:1903506 | regulation of nucleic acid-templated transcription | 0.000371056 | 224 |
| GO:0071705 | nitrogen compound transport | 0.000372008 | 157 |
| GO:0099565 | chemical synaptic transmission, postsynaptic | 0.000395084 | 15 |
| GO:0060996 | dendritic spine development | 0.000410057 | 14 |
| GO:1903363 | negative regulation of cellular protein catabolic process | 0.000413672 | 13 |
| GO:2000463 | positive regulation of excitatory postsynaptic potential | 0.000414481 | 7 |
| GO:0001701 | in utero embryonic development | 0.000420795 | 35 |
| GO:2001141 | regulation of RNA biosynthetic process | 0.00042886 | 224 |
| GO:0043009 | chordate embryonic development | 0.000429332 | 52 |
| GO:0006355 | regulation of transcription, DNA-templated | 0.000445215 | 220 |
| GO:0009792 | embryo development ending in birth or egg hatching | 0.000455294 | 53 |
| GO:0021953 | central nervous system neuron differentiation | 0.000459972 | 21 |
| GO:0031399 | regulation of protein modification process | 0.000490151 | 127 |
| GO:1902017 | regulation of cilium assembly | 0.000491182 | 10 |
| GO:0043087 | regulation of GTPase activity | 0.000493987 | 42 |
| GO:0034654 | nucleobase-containing compound biosynthetic process | 0.000494325 | 262 |
| GO:0031330 | negative regulation of cellular catabolic process | 0.000502621 | 27 |
| GO:0061502 | early endosome to recycling endosome transport | 0.000514933 | 3 |
| GO:1901537 | positive regulation of DNA demethylation | 0.000514933 | 3 |
| GO:1990168 | protein K33-linked deubiquitination | 0.000514933 | 3 |
| GO:0060765 | regulation of androgen receptor signaling pathway | 0.000522438 | 7 |
| GO:0046578 | regulation of Ras protein signal transduction | 0.000539189 | 25 |
| GO:0071407 | cellular response to organic cyclic compound | 0.000554522 | 46 |
| GO:0050808 | synapse organization | 0.000560473 | 37 |
| GO:0060079 | excitatory postsynaptic potential | 0.000562188 | 14 |
| GO:0000122 | negative regulation of transcription by RNA polymerase II | 0.000564962 | 65 |
| GO:0031326 | regulation of cellular biosynthetic process | 0.000574315 | 264 |
| GO:0030100 | regulation of endocytosis | 0.00060497 | 28 |
| GO:0048511 | rhythmic process | 0.000607204 | 29 |
| GO:0061564 | axon development | 0.000607317 | 44 |
| GO:0051254 | positive regulation of RNA metabolic process | 0.000614174 | 117 |
| GO:0045595 | regulation of cell differentiation | 0.000617282 | 127 |
| GO:0060255 | regulation of macromolecule metabolic process | 0.000628556 | 395 |
| GO:0022411 | cellular component disassembly | 0.000648442 | 46 |
| GO:0045935 | positive regulation of nucleobase-containing compound metabolic process | 0.000666785 | 128 |
| GO:0016050 | vesicle organization | 0.000667469 | 31 |
| GO:0031346 | positive regulation of cell projection organization | 0.000680833 | 35 |
| GO:0071310 | cellular response to organic substance | 0.000682545 | 174 |
| GO:0018107 | peptidyl-threonine phosphorylation | 0.00070845 | 16 |
| GO:0023056 | positive regulation of signaling | 0.000720827 | 125 |
| GO:0045184 | establishment of protein localization | 0.000746376 | 140 |
| GO:1990090 | cellular response to nerve growth factor stimulus | 0.0007528 | 9 |
| GO:0035601 | protein deacylation | 0.000759771 | 14 |
| GO:0071495 | cellular response to endogenous stimulus | 0.000763579 | 98 |
| GO:0070988 | demethylation | 0.0008092 | 11 |
| GO:0009653 | anatomical structure morphogenesis | 0.000813713 | 175 |
| GO:0019438 | aromatic compound biosynthetic process | 0.000832517 | 264 |
| GO:0065008 | regulation of biological quality | 0.000833665 | 248 |
| GO:1901565 | organonitrogen compound catabolic process | 0.00083657 | 92 |
| GO:0098732 | macromolecule deacylation | 0.000837462 | 14 |
| GO:1901699 | cellular response to nitrogen compound | 0.000910616 | 52 |
| GO:0061025 | membrane fusion | 0.000918789 | 18 |
| GO:0018130 | heterocycle biosynthetic process | 0.000936741 | 263 |
| GO:0030397 | membrane disassembly | 0.000947532 | 5 |
| GO:0051081 | nuclear envelope disassembly | 0.000947532 | 5 |
| GO:1900242 | regulation of synaptic vesicle endocytosis | 0.000947532 | 5 |
| GO:0030036 | actin cytoskeleton organization | 0.000985256 | 53 |
| GO:0030521 | androgen receptor signaling pathway | 0.000987415 | 10 |
| GO:0045055 | regulated exocytosis | 0.00101651 | 61 |
| GO:0031050 | dsRNA processing | 0.001017288 | 9 |
| GO:0070918 | production of small RNA involved in gene silencing by RNA | 0.001017288 | 9 |
| GO:1903421 | regulation of synaptic vesicle recycling | 0.001077925 | 6 |
| GO:0048168 | regulation of neuronal synaptic plasticity | 0.00117524 | 9 |
| GO:1990089 | response to nerve growth factor | 0.00117524 | 9 |
| GO:0030518 | intracellular steroid hormone receptor signaling pathway | 0.001175282 | 16 |
| GO:0042177 | negative regulation of protein catabolic process | 0.001175282 | 16 |
| GO:0009895 | negative regulation of catabolic process | 0.001198771 | 29 |
| GO:0015833 | peptide transport | 0.001220467 | 134 |
| GO:0001661 | conditioned taste aversion | 0.00123817 | 3 |
| GO:0035523 | protein K29-linked deubiquitination | 0.00123817 | 3 |
| GO:0006468 | protein phosphorylation | 0.001252927 | 131 |
| GO:0010647 | positive regulation of cell communication | 0.001257493 | 123 |
| GO:1903508 | positive regulation of nucleic acid-templated transcription | 0.001294695 | 110 |
| GO:0032880 | regulation of protein localization | 0.001294736 | 74 |
| GO:1902680 | positive regulation of RNA biosynthetic process | 0.001322013 | 110 |
| GO:0016055 | Wnt signaling pathway | 0.001331934 | 43 |
| GO:0051345 | positive regulation of hydrolase activity | 0.001340398 | 59 |
| GO:1903307 | positive regulation of regulated secretory pathway | 0.001352415 | 9 |
| GO:0045956 | positive regulation of calcium ion-dependent exocytosis | 0.00135753 | 6 |
| GO:0006897 | endocytosis | 0.001367394 | 62 |
| GO:0045892 | negative regulation of transcription, DNA-templated | 0.00140761 | 86 |
| GO:0198738 | cell-cell signaling by wnt | 0.001434761 | 43 |
| GO:0030032 | lamellipodium assembly | 0.001444402 | 10 |
| GO:1902117 | positive regulation of organelle assembly | 0.001452201 | 11 |
| GO:0046903 | secretion | 0.001453284 | 112 |
| GO:0007417 | central nervous system development | 0.001457285 | 73 |
| GO:0032940 | secretion by cell | 0.001465573 | 104 |
| GO:1903507 | negative regulation of nucleic acid-templated transcription | 0.00149435 | 89 |
| GO:0007165 | signal transduction | 0.001518267 | 358 |
| GO:0071702 | organic substance transport | 0.001528007 | 176 |
| GO:2000113 | negative regulation of cellular macromolecule biosynthetic process | 0.001536463 | 102 |
| GO:0071417 | cellular response to organonitrogen compound | 0.001537297 | 47 |
| GO:0030042 | actin filament depolymerization | 0.001550473 | 9 |
| GO:0051246 | regulation of protein metabolic process | 0.001560153 | 184 |
| GO:1902679 | negative regulation of RNA biosynthetic process | 0.001565316 | 89 |
| GO:0042886 | amide transport | 0.001568127 | 135 |
| GO:0060999 | positive regulation of dendritic spine development | 0.001571419 | 8 |
| GO:0010558 | negative regulation of macromolecule biosynthetic process | 0.001612528 | 107 |
| GO:0000380 | alternative mRNA splicing, via spliceosome | 0.00162102 | 11 |
| GO:0015031 | protein transport | 0.001660206 | 131 |
| GO:1901564 | organonitrogen compound metabolic process | 0.001696225 | 404 |
| GO:0018193 | peptidyl-amino acid modification | 0.00171191 | 88 |
| GO:0045921 | positive regulation of exocytosis | 0.001717485 | 12 |
| GO:0016071 | mRNA metabolic process | 0.001729231 | 65 |
| GO:0099173 | postsynapse organization | 0.001763158 | 18 |
| GO:0009890 | negative regulation of biosynthetic process | 0.001778962 | 112 |
| GO:0016575 | histone deacetylation | 0.001805536 | 11 |
| GO:0007420 | brain development | 0.001823779 | 56 |
| GO:0007416 | synapse assembly | 0.001857969 | 19 |
| GO:0050807 | regulation of synapse organization | 0.001893626 | 22 |
| GO:0010638 | positive regulation of organelle organization | 0.001916781 | 49 |
| GO:0021954 | central nervous system neuron development | 0.002006815 | 11 |
| GO:0010256 | endomembrane system organization | 0.002008537 | 37 |
| GO:1905515 | non-motile cilium assembly | 0.002016249 | 9 |
| GO:0009896 | positive regulation of catabolic process | 0.002022265 | 36 |
| GO:0003091 | renal water homeostasis | 0.002044646 | 7 |
| GO:0042752 | regulation of circadian rhythm | 0.002055201 | 14 |
| GO:0035196 | production of miRNAs involved in gene silencing by miRNA | 0.002113935 | 8 |
| GO:0007049 | cell cycle | 0.002128233 | 122 |
| GO:0000278 | mitotic cell cycle | 0.002142375 | 73 |
| GO:0009790 | embryo development | 0.002142375 | 73 |
| GO:0060078 | regulation of postsynaptic membrane potential | 0.002180775 | 16 |
| GO:0051493 | regulation of cytoskeleton organization | 0.002210368 | 43 |
| GO:0016082 | synaptic vesicle priming | 0.002219112 | 5 |
| GO:1901701 | cellular response to oxygen-containing compound | 0.002250128 | 80 |
| GO:0060560 | developmental growth involved in morphogenesis | 0.002253648 | 23 |
| GO:1901362 | organic cyclic compound biosynthetic process | 0.002269509 | 268 |
| GO:0048814 | regulation of dendrite morphogenesis | 0.002294257 | 12 |
| GO:0031339 | negative regulation of vesicle fusion | 0.00238202 | 3 |
| GO:0099011 | neuronal dense core vesicle exocytosis | 0.00238202 | 3 |
| GO:0099525 | presynaptic dense core vesicle exocytosis | 0.00238202 | 3 |
| GO:1902903 | regulation of supramolecular fiber organization | 0.002391267 | 31 |
| GO:0007077 | mitotic nuclear envelope disassembly | 0.002429119 | 4 |
| GO:0016081 | synaptic vesicle docking | 0.002429119 | 4 |
| GO:0038180 | nerve growth factor signaling pathway | 0.002429119 | 4 |
| GO:1904862 | inhibitory synapse assembly | 0.002429119 | 4 |
| GO:0030029 | actin filament-based process | 0.002439521 | 57 |
| GO:0031669 | cellular response to nutrient levels | 0.002511496 | 23 |
| GO:0034332 | adherens junction organization | 0.002521688 | 16 |
| GO:0070076 | histone lysine demethylation | 0.002532932 | 6 |
| GO:0019045 | latent virus replication | 0.002619364 | 2 |
| GO:0019046 | release from viral latency | 0.002619364 | 2 |
| GO:0035408 | histone H3-T6 phosphorylation | 0.002619364 | 2 |
| GO:0070560 | protein secretion by platelet | 0.002619364 | 2 |
| GO:0110061 | regulation of angiotensin-activated signaling pathway | 0.002619364 | 2 |
| GO:1901187 | regulation of ephrin receptor signaling pathway | 0.002619364 | 2 |
| GO:1902954 | regulation of early endosome to recycling endosome transport | 0.002619364 | 2 |
| GO:1904327 | protein localization to cytosolic proteasome complex | 0.002619364 | 2 |
| GO:1904379 | protein localization to cytosolic proteasome complex involved in ERAD pathway | 0.002619364 | 2 |
| GO:1905696 | regulation of polysome binding | 0.002619364 | 2 |
| GO:1905704 | positive regulation of inhibitory synapse assembly | 0.002619364 | 2 |
| GO:2000474 | regulation of opioid receptor signaling pathway | 0.002619364 | 2 |
| GO:0044237 | cellular metabolic process | 0.00263735 | 620 |
| GO:0065003 | protein-containing complex assembly | 0.002642982 | 128 |
| GO:0065009 | regulation of molecular function | 0.002651948 | 185 |
| GO:0010976 | positive regulation of neuron projection development | 0.002663409 | 26 |
| GO:0007045 | cell-substrate adherens junction assembly | 0.002722443 | 11 |
| GO:0048041 | focal adhesion assembly | 0.002722443 | 11 |
| GO:0007389 | pattern specification process | 0.002732542 | 37 |
| GO:0030099 | myeloid cell differentiation | 0.002794674 | 35 |
| GO:0007051 | spindle organization | 0.002806522 | 18 |
| GO:0098657 | import into cell | 0.002830013 | 68 |
| GO:0042053 | regulation of dopamine metabolic process | 0.00283547 | 5 |
| GO:0042069 | regulation of catecholamine metabolic process | 0.00283547 | 5 |
| GO:0043401 | steroid hormone mediated signaling pathway | 0.002890677 | 19 |
| GO:0010506 | regulation of autophagy | 0.002933433 | 29 |
| GO:0032870 | cellular response to hormone stimulus | 0.00296585 | 53 |
| GO:0016577 | histone demethylation | 0.003057409 | 6 |
| GO:1900181 | negative regulation of protein localization to nucleus | 0.003057409 | 6 |
| GO:0016482 | cytosolic transport | 0.003068476 | 17 |
| GO:0031327 | negative regulation of cellular biosynthetic process | 0.003074125 | 109 |
| GO:0031647 | regulation of protein stability | 0.003076309 | 26 |
| GO:0006913 | nucleocytoplasmic transport | 0.003082694 | 30 |
| GO:0032956 | regulation of actin cytoskeleton organization | 0.003082694 | 30 |
| GO:0006914 | autophagy | 0.003112814 | 40 |
| GO:0061919 | process utilizing autophagic mechanism | 0.003112814 | 40 |
| GO:0050793 | regulation of developmental process | 0.003258836 | 168 |
| GO:1903530 | regulation of secretion by cell | 0.003264587 | 56 |
| GO:0071277 | cellular response to calcium ion | 0.003304575 | 11 |
| GO:0034645 | cellular macromolecule biosynthetic process | 0.003365726 | 291 |
| GO:0001845 | phagolysosome assembly | 0.003367612 | 4 |
| GO:0035641 | locomotory exploration behavior | 0.003367612 | 4 |
| GO:0051169 | nuclear transport | 0.003501277 | 30 |
| GO:0001956 | positive regulation of neurotransmitter secretion | 0.00356663 | 5 |
| GO:2000810 | regulation of bicellular tight junction assembly | 0.00356663 | 5 |
| GO:0043933 | protein-containing complex subunit organization | 0.003581878 | 145 |
| GO:0010586 | miRNA metabolic process | 0.003658854 | 6 |
| GO:0033365 | protein localization to organelle | 0.003659716 | 66 |
| GO:0050790 | regulation of catalytic activity | 0.003717422 | 147 |
| GO:1903047 | mitotic cell cycle process | 0.003733007 | 63 |
| GO:0009719 | response to endogenous stimulus | 0.00378175 | 109 |
| GO:0051592 | response to calcium ion | 0.003819572 | 16 |
| GO:1903320 | regulation of protein modification by small protein conjugation or removal | 0.003865159 | 22 |
| GO:0043484 | regulation of RNA splicing | 0.003868598 | 15 |
| GO:0060322 | head development | 0.003921548 | 57 |
| GO:0007044 | cell-substrate junction assembly | 0.003921647 | 12 |
| GO:0044773 | mitotic DNA damage checkpoint | 0.003921647 | 12 |
| GO:0010628 | positive regulation of gene expression | 0.003962452 | 129 |
| GO:0036302 | atrioventricular canal development | 0.004010186 | 3 |
| GO:1901535 | regulation of DNA demethylation | 0.004010186 | 3 |
| GO:0034330 | cell junction organization | 0.004066209 | 26 |
| GO:0110053 | regulation of actin filament organization | 0.004111521 | 24 |
| GO:0030834 | regulation of actin filament depolymerization | 0.004119502 | 8 |
| GO:1900006 | positive regulation of dendrite development | 0.004328117 | 10 |
| GO:0009059 | macromolecule biosynthetic process | 0.004330536 | 298 |
| GO:0006482 | protein demethylation | 0.004343805 | 6 |
| GO:0008214 | protein dealkylation | 0.004343805 | 6 |
| GO:0018205 | peptidyl-lysine modification | 0.004363845 | 33 |
| GO:0098815 | modulation of excitatory postsynaptic potential | 0.004410939 | 7 |
| GO:0002091 | negative regulation of receptor internalization | 0.004525398 | 4 |
| GO:0038166 | angiotensin-activated signaling pathway | 0.004525398 | 4 |
| GO:0010243 | response to organonitrogen compound | 0.004560491 | 70 |
| GO:0043112 | receptor metabolic process | 0.004618789 | 19 |
| GO:0006998 | nuclear envelope organization | 0.004654823 | 8 |
| GO:1903828 | negative regulation of cellular protein localization | 0.004772391 | 13 |
| GO:0044271 | cellular nitrogen compound biosynthetic process | 0.004787858 | 293 |
| GO:0045944 | positive regulation of transcription by RNA polymerase II | 0.004827223 | 82 |
| GO:0030177 | positive regulation of Wnt signaling pathway | 0.004859945 | 18 |
| GO:0016310 | phosphorylation | 0.004870871 | 151 |
| GO:0006397 | mRNA processing | 0.004887966 | 42 |
| GO:0022402 | cell cycle process | 0.004921628 | 93 |
| GO:0031057 | negative regulation of histone modification | 0.005064031 | 7 |
| GO:0044088 | regulation of vacuole organization | 0.005064031 | 7 |
| GO:0016571 | histone methylation | 0.005087242 | 15 |
| GO:0045893 | positive regulation of transcription, DNA-templated | 0.005133561 | 101 |
| GO:0007623 | circadian rhythm | 0.005135136 | 20 |
| GO:1901888 | regulation of cell junction assembly | 0.005202597 | 11 |
| GO:0000422 | autophagy of mitochondrion | 0.005247769 | 10 |
| GO:0061726 | mitochondrion disassembly | 0.005247769 | 10 |
| GO:0031503 | protein-containing complex localization | 0.005271761 | 25 |
| GO:1903008 | organelle disassembly | 0.005447165 | 12 |
| GO:0034968 | histone lysine methylation | 0.005545118 | 13 |
| GO:1901575 | organic substance catabolic process | 0.005560674 | 137 |
| GO:0010770 | positive regulation of cell morphogenesis involved in differentiation | 0.005621525 | 16 |
| GO:0000381 | regulation of alternative mRNA splicing, via spliceosome | 0.005651936 | 9 |
| GO:0031668 | cellular response to extracellular stimulus | 0.005706949 | 24 |
| GO:0035050 | embryonic heart tube development | 0.005761535 | 10 |
| GO:0006904 | vesicle docking involved in exocytosis | 0.005787155 | 7 |
| GO:0030866 | cortical actin cytoskeleton organization | 0.005787155 | 7 |
| GO:0071364 | cellular response to epidermal growth factor stimulus | 0.005787155 | 7 |
| GO:0035635 | entry of bacterium into host cell | 0.005923751 | 4 |
| GO:0048172 | regulation of short-term neuronal synaptic plasticity | 0.005923751 | 4 |
| GO:0060766 | negative regulation of androgen receptor signaling pathway | 0.005923751 | 4 |
| GO:0016049 | cell growth | 0.006020308 | 38 |
| GO:0034333 | adherens junction assembly | 0.006167909 | 11 |
| GO:0034720 | histone H3-K4 demethylation | 0.006173222 | 3 |
| GO:0060896 | neural plate pattern specification | 0.006173222 | 3 |
| GO:0061669 | spontaneous neurotransmitter secretion | 0.006173222 | 3 |
| GO:0090385 | phagosome-lysosome fusion | 0.006173222 | 3 |
| GO:0008589 | regulation of smoothened signaling pathway | 0.0063137 | 10 |
| GO:0030433 | ubiquitin-dependent ERAD pathway | 0.0063137 | 10 |
| GO:0033143 | regulation of intracellular steroid hormone receptor signaling pathway | 0.0063137 | 10 |
| GO:0048259 | regulation of receptor-mediated endocytosis | 0.00636925 | 12 |
| GO:0031331 | positive regulation of cellular catabolic process | 0.00638887 | 30 |
| GO:1900078 | positive regulation of cellular response to insulin stimulus | 0.006560074 | 5 |
| GO:0014047 | glutamate secretion | 0.006584726 | 7 |
| GO:0034105 | positive regulation of tissue remodeling | 0.006584726 | 7 |
| GO:0061001 | regulation of dendritic spine morphogenesis | 0.006584726 | 7 |
| GO:1902667 | regulation of axon guidance | 0.006584726 | 7 |
| GO:0006997 | nucleus organization | 0.006785689 | 14 |
| GO:0060401 | cytosolic calcium ion transport | 0.00681893 | 17 |
| GO:0010508 | positive regulation of autophagy | 0.006888408 | 13 |
| GO:0060402 | calcium ion transport into cytosol | 0.007166842 | 16 |
| GO:1902531 | regulation of intracellular signal transduction | 0.007351915 | 124 |
| GO:0032456 | endocytic recycling | 0.007461177 | 7 |
| GO:1902743 | regulation of lamellipodium organization | 0.007461177 | 7 |
| GO:0000077 | DNA damage checkpoint | 0.007480907 | 15 |
| GO:0007612 | learning | 0.007480907 | 15 |
| GO:0140029 | exocytic process | 0.0075407 | 10 |
| GO:0042176 | regulation of protein catabolic process | 0.007576634 | 31 |
| GO:2000369 | regulation of clathrin-dependent endocytosis | 0.00758249 | 4 |
| GO:2001224 | positive regulation of neuron migration | 0.00758249 | 4 |
| GO:0003383 | apical constriction | 0.007590375 | 2 |
| GO:0032804 | negative regulation of low-density lipoprotein particle receptor catabolic process | 0.007590375 | 2 |
| GO:0033693 | neurofilament bundle assembly | 0.007590375 | 2 |
| GO:0035668 | TRAM-dependent toll-like receptor signaling pathway | 0.007590375 | 2 |
| GO:0035669 | TRAM-dependent toll-like receptor 4 signaling pathway | 0.007590375 | 2 |
| GO:0036369 | transcription factor catabolic process | 0.007590375 | 2 |
| GO:0043578 | nuclear matrix organization | 0.007590375 | 2 |
| GO:0051595 | response to methylglyoxal | 0.007590375 | 2 |
| GO:0090292 | nuclear matrix anchoring at nuclear membrane | 0.007590375 | 2 |
| GO:1902856 | negative regulation of non-motile cilium assembly | 0.007590375 | 2 |
| GO:1903294 | regulation of glutamate secretion, neurotransmission | 0.007590375 | 2 |
| GO:1903296 | positive regulation of glutamate secretion, neurotransmission | 0.007590375 | 2 |
| GO:1904048 | regulation of spontaneous neurotransmitter secretion | 0.007590375 | 2 |
| GO:1904158 | axonemal central apparatus assembly | 0.007590375 | 2 |
| GO:1905702 | regulation of inhibitory synapse assembly | 0.007590375 | 2 |
| GO:1990108 | protein linear deubiquitination | 0.007590375 | 2 |
| GO:1990167 | protein K27-linked deubiquitination | 0.007590375 | 2 |
| GO:1990926 | short-term synaptic potentiation | 0.007590375 | 2 |
| GO:2001160 | regulation of histone H3-K79 methylation | 0.007590375 | 2 |
| GO:0016239 | positive regulation of macroautophagy | 0.007607065 | 9 |
| GO:0045806 | negative regulation of endocytosis | 0.007607065 | 9 |
| GO:0071242 | cellular response to ammonium ion | 0.007607065 | 9 |
| GO:1901698 | response to nitrogen compound | 0.007629289 | 74 |
| GO:0021952 | central nervous system projection neuron axonogenesis | 0.00786071 | 5 |
| GO:0007052 | mitotic spindle organization | 0.007978854 | 12 |
| GO:0044774 | mitotic DNA integrity checkpoint | 0.007978854 | 12 |
| GO:0001881 | receptor recycling | 0.00805055 | 6 |
| GO:0021955 | central nervous system neuron axonogenesis | 0.00805055 | 6 |
| GO:0098751 | bone cell development | 0.00805055 | 6 |
| GO:2000785 | regulation of autophagosome assembly | 0.00805055 | 6 |
| GO:0071241 | cellular response to inorganic substance | 0.008122715 | 20 |
| GO:0007224 | smoothened signaling pathway | 0.008256561 | 14 |
| GO:0010557 | positive regulation of macromolecule biosynthetic process | 0.008340814 | 119 |
| GO:1903362 | regulation of cellular protein catabolic process | 0.008398175 | 22 |
| GO:0032480 | negative regulation of type I interferon production | 0.008420939 | 7 |
| GO:0034504 | protein localization to nucleus | 0.008460148 | 23 |
| GO:0032271 | regulation of protein polymerization | 0.008527426 | 20 |
| GO:0071248 | cellular response to metal ion | 0.008897263 | 18 |
| GO:0071496 | cellular response to external stimulus | 0.00889996 | 28 |
| GO:0001188 | RNA polymerase I preinitiation complex assembly | 0.008909993 | 3 |
| GO:0048312 | intracellular distribution of mitochondria | 0.008909993 | 3 |
| GO:0070922 | small RNA loading onto RISC | 0.008909993 | 3 |
| GO:0097011 | cellular response to granulocyte macrophage colony-stimulating factor stimulus | 0.008909993 | 3 |
| GO:0097012 | response to granulocyte macrophage colony-stimulating factor | 0.008909993 | 3 |
| GO:1990504 | dense core granule exocytosis | 0.008909993 | 3 |
| GO:0060997 | dendritic spine morphogenesis | 0.00906435 | 8 |
| GO:0048588 | developmental cell growth | 0.009096522 | 21 |
| GO:0045666 | positive regulation of neuron differentiation | 0.009251223 | 30 |
| GO:0048169 | regulation of long-term neuronal synaptic plasticity | 0.00933011 | 5 |
| GO:0080111 | DNA demethylation | 0.00933011 | 5 |
| GO:1904353 | regulation of telomere capping | 0.00933011 | 5 |
| GO:0030010 | establishment of cell polarity | 0.009369783 | 14 |
| GO:0070849 | response to epidermal growth factor | 0.009468423 | 7 |
| GO:0090304 | nucleic acid metabolic process | 0.009492254 | 327 |
| GO:0048311 | mitochondrion distribution | 0.009519827 | 4 |
| GO:0008277 | regulation of G protein-coupled receptor signaling pathway | 0.009524111 | 15 |
| GO:0051046 | regulation of secretion | 0.009527084 | 57 |
| GO:0007033 | vacuole organization | 0.009559547 | 16 |
| GO:0043161 | proteasome-mediated ubiquitin-dependent protein catabolic process | 0.009570702 | 33 |
| GO:0030522 | intracellular receptor signaling pathway | 0.009618649 | 24 |
| GO:0032970 | regulation of actin filament-based process | 0.00971623 | 31 |
| GO:0033554 | cellular response to stress | 0.009811819 | 129 |
| GO:0051261 | protein depolymerization | 0.009890612 | 12 |
| GO:1905037 | autophagosome organization | 0.00992429 | 11 |
| GO:0006886 | intracellular protein transport | 0.009934826 | 75 |

**CC**

| ID | Description | p-value | Count |
| --- | --- | --- | --- |
| GO:0005765 | lysosomal membrane | 0.006796484 | 29 |
| GO:0005635 | nuclear envelope | 0.00659977 | 36 |
| GO:0031234 | extrinsic component of cytoplasmic side of plasma membrane | 0.005814039 | 11 |
| GO:0010494 | cytoplasmic stress granule | 0.005521195 | 9 |
| GO:0035869 | ciliary transition zone | 0.005521195 | 9 |
| GO:0030863 | cortical cytoskeleton | 0.005473095 | 13 |
| GO:0005819 | spindle | 0.005180061 | 29 |
| GO:0070160 | tight junction | 0.005012954 | 14 |
| GO:0032839 | dendrite cytoplasm | 0.004655069 | 6 |
| GO:0070603 | SWI/SNF superfamily-type complex | 0.004582674 | 10 |
| GO:0099738 | cell cortex region | 0.00455388 | 7 |
| GO:0034708 | methyltransferase complex | 0.00438011 | 13 |
| GO:0000151 | ubiquitin ligase complex | 0.004328044 | 25 |
| GO:0099523 | presynaptic cytosol | 0.004218628 | 4 |
| GO:0030286 | dynein complex | 0.003660689 | 8 |
| GO:0005923 | bicellular tight junction | 0.00350027 | 14 |
| GO:0055037 | recycling endosome | 0.003325482 | 18 |
| GO:0034451 | centriolar satellite | 0.003321822 | 6 |
| GO:0016580 | Sin3 complex | 0.003136837 | 4 |
| GO:0005769 | early endosome | 0.003134659 | 30 |
| GO:0098982 | GABA-ergic synapse | 0.002775199 | 10 |
| GO:0090568 | nuclear transcriptional repressor complex | 0.002773463 | 6 |
| GO:0030904 | retromer complex | 0.002603954 | 5 |
| GO:0071782 | endoplasmic reticulum tubular network | 0.002603954 | 5 |
| GO:0043025 | neuronal cell body | 0.002305179 | 40 |
| GO:0032039 | integrator complex | 0.002295773 | 6 |
| GO:0043296 | apical junction complex | 0.002233061 | 16 |
| GO:0005874 | microtubule | 0.002043769 | 35 |
| GO:0030992 | intraciliary transport particle B | 0.002036248 | 5 |
| GO:0031514 | motile cilium | 0.001699313 | 19 |
| GO:0035097 | histone methyltransferase complex | 0.001189388 | 12 |
| GO:0097060 | synaptic membrane | 0.001128645 | 37 |
| GO:0072686 | mitotic spindle | 0.001109322 | 14 |
| GO:0044309 | neuron spine | 0.000988931 | 19 |
| GO:0044305 | calyx of Held | 0.000973714 | 6 |
| GO:0019898 | extrinsic component of membrane | 0.000959541 | 28 |
| GO:0070822 | Sin3-type complex | 0.000867298 | 5 |
| GO:0098831 | presynaptic active zone cytoplasmic component | 0.000867298 | 5 |
| GO:0043197 | dendritic spine | 0.000857738 | 19 |
| GO:0060076 | excitatory synapse | 0.000763504 | 9 |
| GO:0030027 | lamellipodium | 0.000724913 | 21 |
| GO:0044447 | axoneme part | 0.00071921 | 8 |
| GO:0010008 | endosome membrane | 0.000634828 | 41 |
| GO:0044441 | ciliary part | 0.000588442 | 40 |
| GO:0098563 | intrinsic component of synaptic vesicle membrane | 0.000475159 | 9 |
| GO:0001726 | ruffle | 0.000410795 | 20 |
| GO:0098978 | glutamatergic synapse | 0.000379373 | 33 |
| GO:0035770 | ribonucleoprotein granule | 0.000369169 | 24 |
| GO:0031252 | cell leading edge | 0.000308699 | 37 |
| GO:0043679 | axon terminus | 0.000300747 | 16 |
| GO:0030990 | intraciliary transport particle | 0.000288635 | 7 |
| GO:0017053 | transcriptional repressor complex | 0.00027107 | 13 |
| GO:0005774 | vacuolar membrane | 0.000235077 | 38 |
| GO:0036464 | cytoplasmic ribonucleoprotein granule | 0.000172612 | 24 |
| GO:0042734 | presynaptic membrane | 0.000169695 | 20 |
| GO:0005881 | cytoplasmic microtubule | 0.000149745 | 12 |
| GO:0044448 | cell cortex part | 0.000144413 | 22 |
| GO:0150034 | distal axon | 0.00010877 | 30 |
| GO:0030133 | transport vesicle | 8.47E-05 | 38 |
| GO:0044306 | neuron projection terminus | 6.14E-05 | 19 |
| GO:0033267 | axon part | 4.88E-05 | 38 |
| GO:0030285 | integral component of synaptic vesicle membrane | 3.29E-05 | 9 |
| GO:0005802 | trans-Golgi network | 2.21E-05 | 28 |
| GO:0000790 | nuclear chromatin | 1.62E-05 | 39 |
| GO:0070382 | exocytic vesicle | 1.58E-05 | 26 |
| GO:0005938 | cell cortex | 1.45E-05 | 34 |
| GO:0097542 | ciliary tip | 1.02E-05 | 11 |
| GO:0048786 | presynaptic active zone | 9.76E-06 | 14 |
| GO:0030658 | transport vesicle membrane | 5.91E-06 | 27 |
| GO:0000118 | histone deacetylase complex | 4.57E-06 | 13 |
| GO:0008021 | synaptic vesicle | 3.64E-06 | 26 |
| GO:0097014 | ciliary plasm | 9.09E-07 | 21 |
| GO:0005930 | axoneme | 7.92E-07 | 21 |
| GO:0016607 | nuclear speck | 7.53E-07 | 44 |
| GO:0014069 | postsynaptic density | 4.00E-07 | 39 |
| GO:0099572 | postsynaptic specialization | 3.61E-07 | 41 |
| GO:0032279 | asymmetric synapse | 2.01E-07 | 40 |
| GO:0098984 | neuron to neuron synapse | 5.71E-08 | 43 |
| GO:0030672 | synaptic vesicle membrane | 4.05E-08 | 21 |
| GO:0099501 | exocytic vesicle membrane | 4.05E-08 | 21 |
| GO:0098793 | presynapse | 2.18E-08 | 55 |
| GO:0032838 | plasma membrane bounded cell projection cytoplasm | 1.54E-08 | 32 |

**MF**

| ID | Description | p-value | Count |
| --- | --- | --- | --- |
| GO:0008569 | ATP-dependent microtubule motor activity, minus-end-directed | 0.002367739 | 5 |
| GO:0005049 | nuclear export signal receptor activity | 0.00221999 | 4 |
| GO:0046965 | retinoid X receptor binding | 0.001790555 | 5 |
| GO:0004386 | helicase activity | 0.001650264 | 19 |
| GO:0003713 | transcription coactivator activity | 0.001608287 | 31 |
| GO:0035591 | signaling adaptor activity | 0.001388781 | 12 |
| GO:0046332 | SMAD binding | 0.001388781 | 12 |
| GO:0042393 | histone binding | 0.001307219 | 22 |
| GO:0140142 | nucleocytoplasmic carrier activity | 0.001243915 | 7 |
| GO:0000149 | SNARE binding | 0.001029675 | 15 |
| GO:0019905 | syntaxin binding | 0.000774943 | 12 |
| GO:0042162 | telomeric DNA binding | 0.000767365 | 8 |
| GO:0005085 | guanyl-nucleotide exchange factor activity | 0.000760522 | 24 |
| GO:0016922 | nuclear receptor binding | 0.000550385 | 6 |
| GO:0017048 | Rho GTPase binding | 0.000304482 | 22 |
| GO:0061630 | ubiquitin protein ligase activity | 0.000221202 | 26 |
| GO:1901981 | phosphatidylinositol phosphate binding | 0.000212487 | 21 |
| GO:0005516 | calmodulin binding | 0.000111434 | 25 |
| GO:0060589 | nucleoside-triphosphatase regulator activity | 8.05E-05 | 37 |
| GO:0061659 | ubiquitin-like protein ligase activity | 7.11E-05 | 28 |
| GO:0017075 | syntaxin-1 binding | 5.33E-05 | 8 |
| GO:0060090 | molecular adaptor activity | 4.84E-05 | 29 |
| GO:0004674 | protein serine/threonine kinase activity | 4.66E-05 | 45 |
| GO:0017137 | Rab GTPase binding | 3.43E-05 | 24 |
| GO:0030695 | GTPase regulator activity | 3.10E-05 | 35 |
| GO:0061578 | Lys63-specific deubiquitinase activity | 1.92E-05 | 6 |
| GO:0140030 | modification-dependent protein binding | 1.32E-05 | 22 |
| GO:0070577 | lysine-acetylated histone binding | 3.64E-06 | 8 |
| GO:0140033 | acetylation-dependent protein binding | 3.64E-06 | 8 |
| GO:0005096 | GTPase activator activity | 2.92E-06 | 35 |
| GO:0005543 | phospholipid binding | 2.20E-06 | 48 |
| GO:0035091 | phosphatidylinositol binding | 1.88E-06 | 33 |
| GO:0004842 | ubiquitin-protein transferase activity | 1.29E-06 | 45 |
| GO:0008234 | cysteine-type peptidase activity | 9.93E-07 | 28 |
| GO:0019787 | ubiquitin-like protein transferase activity | 5.53E-07 | 48 |
| GO:1990380 | Lys48-specific deubiquitinase activity | 3.78E-07 | 8 |
| GO:0019783 | ubiquitin-like protein-specific protease activity | 2.52E-09 | 26 |
| GO:0004843 | thiol-dependent ubiquitin-specific protease activity | 2.41E-09 | 24 |
| GO:0036459 | thiol-dependent ubiquitinyl hydrolase activity | 4.42E-10 | 26 |
| GO:0101005 | ubiquitinyl hydrolase activity | 4.42E-10 | 26 |
| GO:0017016 | Ras GTPase binding | 7.62E-11 | 59 |
| GO:0031267 | small GTPase binding | 1.29E-12 | 64 |

**KEGG**

| ID | Description | p-value | Count |
| --- | --- | --- | --- |
| hsa05017 | Spinocerebellar ataxia | 0.000103 | 18 |
| hsa04919 | Thyroid hormone signaling pathway | 0.000139 | 16 |
| hsa05022 | Pathways of neurodegeneration - multiple diseases | 0.000161 | 40 |
| hsa04020 | Calcium signaling pathway | 0.000403 | 21 |
| hsa04072 | Phospholipase D signaling pathway | 0.000481 | 17 |
| hsa04012 | ErbB signaling pathway | 0.000514 | 12 |
| hsa04925 | Aldosterone synthesis and secretion | 0.000562 | 13 |
| hsa04911 | Insulin secretion | 0.000573 | 12 |
| hsa04750 | Inflammatory mediator regulation of TRP channels | 0.000684 | 13 |
| hsa04360 | Axon guidance | 0.000712 | 19 |
| hsa04921 | Oxytocin signaling pathway | 0.000762 | 17 |
| hsa04912 | GnRH signaling pathway | 0.001166 | 12 |
| hsa04970 | Salivary secretion | 0.001166 | 12 |
| hsa04270 | Vascular smooth muscle contraction | 0.001436 | 15 |
| hsa05223 | Non-small cell lung cancer | 0.001686 | 10 |
| hsa04022 | cGMP-PKG signaling pathway | 0.001875 | 17 |
| hsa04724 | Glutamatergic synapse | 0.002321 | 13 |
| hsa04540 | Gap junction | 0.002395 | 11 |
| hsa04971 | Gastric acid secretion | 0.002546 | 10 |
| hsa05205 | Proteoglycans in cancer | 0.003076 | 19 |
| hsa05032 | Morphine addiction | 0.003129 | 11 |
| hsa04144 | Endocytosis | 0.003168 | 22 |
| hsa04720 | Long-term potentiation | 0.00358 | 9 |
| hsa04928 | Parathyroid hormone synthesis, secretion and action | 0.003586 | 12 |
| hsa04070 | Phosphatidylinositol signaling system | 0.005134 | 11 |
| hsa04713 | Circadian entrainment | 0.005134 | 11 |
| hsa04024 | cAMP signaling pathway | 0.00545 | 19 |
| hsa00561 | Glycerolipid metabolism | 0.006837 | 8 |
| hsa04916 | Melanogenesis | 0.006958 | 11 |
| hsa04310 | Wnt signaling pathway | 0.007316 | 15 |
| hsa05110 | Vibrio cholerae infection | 0.007829 | 7 |
| hsa04727 | GABAergic synapse | 0.007966 | 10 |
| hsa04722 | Neurotrophin signaling pathway | 0.009026 | 12 |
| hsa04935 | Growth hormone synthesis, secretion and action | 0.009026 | 12 |
