# Supplementary material for: The regulatory pattern of target gene expression by aberrant enhancer methylation in glioblastoma
Source: BMC Bioinformatics. 2021 Sep 5;22:420. doi: 10.1186/s12859-021-04345-8 (PMC8420065; doi:10.1186/s12859-021-04345-8)
Supplement: Supplementary file 7 — Additional file 7. Table S4. Prognostic modules regulated by hypomethylation enhancer regions in GBM. [file 12859_2021_4345_MOESM7_ESM.docx]

Table S4. Prognostic modules regulated by hypomethylation enhancer regions in GBM

| lncRNA | mRNA |
| --- | --- |
| ANKRD10-IT1 | ANKZF1 |
| ANKRD10-IT1 | AP1G2 |
| ANKRD10-IT1 | ATG2B |
| ANKRD10-IT1 | ATXN3 |
| ANKRD10-IT1 | CELF1 |
| ANKRD10-IT1 | DDX17 |
| ANKRD10-IT1 | DIP2A |
| ANKRD10-IT1 | ELMSAN1 |
| ANKRD10-IT1 | EPG5 |
| ANKRD10-IT1 | MED13 |
| ANKRD10-IT1 | MYO9A |
| ANKRD10-IT1 | NFATC2IP |
| ANKRD10-IT1 | OTUD3 |
| ANKRD10-IT1 | OTUD4 |
| ANKRD10-IT1 | PAN3 |
| ANKRD10-IT1 | TRIO |
| ANKRD10-IT1 | ZNF326 |
| CTB-89H12.4 | ANKZF1 |
| CTB-89H12.4 | AP1G2 |
| CTB-89H12.4 | ATG2B |
| CTB-89H12.4 | ATXN3 |
| CTB-89H12.4 | CELF1 |
| CTB-89H12.4 | DDX17 |
| CTB-89H12.4 | DIP2A |
| CTB-89H12.4 | ELMSAN1 |
| CTB-89H12.4 | EPG5 |
| CTB-89H12.4 | MED13 |
| CTB-89H12.4 | MYO9A |
| CTB-89H12.4 | NFATC2IP |
| CTB-89H12.4 | OTUD3 |
| CTB-89H12.4 | OTUD4 |
| CTB-89H12.4 | PAN3 |
| CTB-89H12.4 | TRIO |
| CTB-89H12.4 | ZNF326 |
|  |  |
| RP11-57H14.4 | ATP6V0A1 |
| RP11-57H14.4 | ATP9A |
| RP11-57H14.4 | CRY2 |
| RP11-57H14.4 | FAM126B |
| RP11-57H14.4 | FBXL16 |
| RP11-57H14.4 | GABARAPL1 |
| RP11-57H14.4 | HECTD4 |
| RP11-57H14.4 | LSM11 |
| RP11-57H14.4 | MYCBP2 |
| RP11-57H14.4 | NBEA |
| RP11-57H14.4 | PRKCE |
| RP11-57H14.4 | RALGPS1 |
| RP11-57H14.4 | RAPGEF2 |
| RP11-57H14.4 | SAMD12 |
| RP11-57H14.4 | SCAMP5 |
| RP11-57H14.4 | SOCS7 |
| RP11-57H14.4 | TNRC6C |
| RP11-690D19.3 | ATP6V0A1 |
| RP11-690D19.3 | ATP9A |
| RP11-690D19.3 | CRY2 |
| RP11-690D19.3 | FAM126B |
| RP11-690D19.3 | FBXL16 |
| RP11-690D19.3 | GABARAPL1 |
| RP11-690D19.3 | HECTD4 |
| RP11-690D19.3 | LSM11 |
| RP11-690D19.3 | MYCBP2 |
| RP11-690D19.3 | NBEA |
| RP11-690D19.3 | PRKCE |
| RP11-690D19.3 | RALGPS1 |
| RP11-690D19.3 | RAPGEF2 |
| RP11-690D19.3 | SAMD12 |
| RP11-690D19.3 | SCAMP5 |
| RP11-690D19.3 | SOCS7 |
| RP11-690D19.3 | TNRC6C |
|  |  |
| CTB-152G17.6 | ARHGEF7 |
| CTB-152G17.6 | FAM126B |
| RP1-257A7.4 | ARHGEF7 |
| RP1-257A7.4 | FAM126B |
| RP11-690D19.3 | ARHGEF7 |
| RP11-690D19.3 | FAM126B |
|  |  |
| CTB-152G17.6 | ARHGEF7 |
| CTB-152G17.6 | FAM126B |
| CTB-152G17.6 | TULP4 |
| RP1-257A7.4 | ARHGEF7 |
| RP1-257A7.4 | FAM126B |
| RP1-257A7.4 | TULP4 |
|  |  |
| KB-1460A1.5 | UBE2G2 |
| RP11-197N18.2 | RAPGEFL1 |
| RP11-197N18.2 | UBE2G2 |
| RP11-66N24.4 | RAPGEFL1 |
| RP11-66N24.4 | UBE2G2 |
| RP11-890B15.3 | RAPGEFL1 |
| RP11-890B15.3 | UBE2G2 |
| ZNF32-AS2 | RAPGEFL1 |
| ZNF32-AS2 | UBE2G2 |
|  |  |
| KB-1460A1.5 | DIP2A |
| KB-1460A1.5 | RAPGEFL1 |
| KB-1460A1.5 | UBE2G2 |
| RP11-197N18.2 | DIP2A |
| RP11-197N18.2 | RAPGEFL1 |
| RP11-197N18.2 | UBE2G2 |
| RP11-890B15.3 | DIP2A |
| RP11-890B15.3 | RAPGEFL1 |
| RP11-890B15.3 | UBE2G2 |
| ZNF32-AS2 | DIP2A |
| ZNF32-AS2 | RAPGEFL1 |
| ZNF32-AS2 | UBE2G2 |
|  |  |
| RP11-197N18.2 | DIP2A |
| RP11-197N18.2 | RAPGEFL1 |
| RP11-197N18.2 | UBE2G2 |
| RP11-197N18.2 | ZZEF1 |
| RP11-890B15.3 | DIP2A |
| RP11-890B15.3 | RAPGEFL1 |
| RP11-890B15.3 | UBE2G2 |
| RP11-890B15.3 | ZZEF1 |
| ZNF32-AS2 | DIP2A |
| ZNF32-AS2 | RAPGEFL1 |
| ZNF32-AS2 | UBE2G2 |
| ZNF32-AS2 | ZZEF1 |
|  |  |
| BX322557.10 | DIP2A |
| BX322557.10 | EP400 |
| BX322557.10 | GGA3 |
| BX322557.10 | OTUD3 |
| BX322557.10 | UBE2G2 |
| BX322557.10 | ULK1 |
| RP11-197N18.2 | DIP2A |
| RP11-197N18.2 | EP400 |
| RP11-197N18.2 | GGA3 |
| RP11-197N18.2 | OTUD3 |
| RP11-197N18.2 | UBE2G2 |
| RP11-197N18.2 | ULK1 |
| RP11-57H14.4 | DIP2A |
| RP11-57H14.4 | EP400 |
| RP11-57H14.4 | GGA3 |
| RP11-57H14.4 | OTUD3 |
| RP11-57H14.4 | UBE2G2 |
| RP11-57H14.4 | ULK1 |
| RP11-67L2.2 | DIP2A |
| RP11-67L2.2 | EP400 |
| RP11-67L2.2 | GGA3 |
| RP11-67L2.2 | OTUD3 |
| RP11-67L2.2 | UBE2G2 |
| RP11-67L2.2 | ULK1 |
|  |  |
| LY86-AS1 | CIDEA |
| LY86-AS1 | FSTL4 |
| LY86-AS1 | JPH3 |
| LY86-AS1 | KCNK1 |
| LY86-AS1 | PPP3CB |
| LY86-AS1 | PTER |
| LY86-AS1 | STXBP1 |
| LY86-AS1 | STXBP5 |
| LY86-AS1 | SYNPR |
| LY86-AS1 | TSPYL1 |
| RP11-286B14.1 | CIDEA |
| RP11-286B14.1 | FSTL4 |
| RP11-286B14.1 | JPH3 |
| RP11-286B14.1 | KCNK1 |
| RP11-286B14.1 | PPP3CB |
| RP11-286B14.1 | PTER |
| RP11-286B14.1 | STXBP1 |
| RP11-286B14.1 | STXBP5 |
| RP11-286B14.1 | SYNPR |
| RP11-286B14.1 | TSPYL1 |
| TRHDE-AS1 | CIDEA |
| TRHDE-AS1 | FSTL4 |
| TRHDE-AS1 | JPH3 |
| TRHDE-AS1 | KCNK1 |
| TRHDE-AS1 | PPP3CB |
| TRHDE-AS1 | PTER |
| TRHDE-AS1 | STXBP1 |
| TRHDE-AS1 | STXBP5 |
| TRHDE-AS1 | SYNPR |
| TRHDE-AS1 | TSPYL1 |
|  |  |
| LINC00507 | SLC6A17 |
| LINC00507 | STXBP1 |
| LINC01511 | SLC6A17 |
| LINC01511 | STXBP1 |
| LY86-AS1 | SLC6A17 |
| LY86-AS1 | STXBP1 |
| RP11-286B14.1 | SLC6A17 |
| RP11-286B14.1 | STXBP1 |
|  |  |
| LY86-AS1 | GABARAPL1 |
| LY86-AS1 | PREPL |
| LY86-AS1 | TSPYL1 |
| RP11-690D19.3 | GABARAPL1 |
| RP11-690D19.3 | PREPL |
| RP11-690D19.3 | TSPYL1 |
|  |  |
| LINC00507 | DMTN |
| LINC00507 | GOT1 |
| LINC00507 | GPR83 |
| LINC00507 | LPAR3 |
| LINC00507 | MAL2 |
| LINC00507 | PTER |
| LINC00507 | STYK1 |
| RP11-215H22.1 | DMTN |
| RP11-215H22.1 | GOT1 |
| RP11-215H22.1 | GPR83 |
| RP11-215H22.1 | LPAR3 |
| RP11-215H22.1 | MAL2 |
| RP11-215H22.1 | PTER |
| RP11-215H22.1 | STYK1 |
|  |  |
| BX322557.10 | ENTPD4 |
| BX322557.10 | NFATC2IP |
| BX322557.10 | SYNGAP1 |
| RP11-235E17.6 | ENTPD4 |
| RP11-235E17.6 | NFATC2IP |
| RP11-235E17.6 | SYNGAP1 |
| ZNF32-AS2 | ENTPD4 |
| ZNF32-AS2 | NFATC2IP |
| ZNF32-AS2 | SYNGAP1 |
|  |  |
| RP1-257A7.4 | JARID2 |
| RP1-257A7.4 | LCOR |
| RP1-257A7.4 | PBRM1 |
| RP11-57H14.4 | JARID2 |
| RP11-57H14.4 | LCOR |
| RP11-57H14.4 | PBRM1 |
| RP3-508I15.19 | JARID2 |
| RP3-508I15.19 | LCOR |
| RP3-508I15.19 | PBRM1 |
| RP4-773N10.4 | JARID2 |
| RP4-773N10.4 | LCOR |
| RP4-773N10.4 | PBRM1 |
| ZNF32-AS2 | JARID2 |
| ZNF32-AS2 | LCOR |
| ZNF32-AS2 | PBRM1 |
|  |  |
| AC004158.3 | ARHGEF7 |
| AC004158.3 | ZCCHC14 |
| PKI55 | ARHGEF7 |
| PKI55 | ZCCHC14 |
| RP1-257A7.4 | ARHGEF7 |
| RP1-257A7.4 | ZCCHC14 |
|  |  |
| BX322557.10 | NEK9 |
| BX322557.10 | ZZEF1 |
| RP11-197N18.2 | NEK9 |
| RP11-197N18.2 | ZZEF1 |
| RP11-235E17.4 | NEK9 |
| RP11-235E17.4 | ZZEF1 |
| RP11-67L2.2 | NEK9 |
| RP11-67L2.2 | ZZEF1 |
| RP4-773N10.4 | NEK9 |
| RP4-773N10.4 | ZZEF1 |
|  |  |
| ANKRD10-IT1 | MLLT6 |
| ANKRD10-IT1 | ZZEF1 |
| BX322557.10 | MLLT6 |
| BX322557.10 | ZZEF1 |
| PKI55 | MLLT6 |
| PKI55 | ZZEF1 |
| RP11-197N18.2 | MLLT6 |
| RP11-197N18.2 | ZZEF1 |
| RP3-508I15.20 | MLLT6 |
| RP3-508I15.20 | ZZEF1 |
| ZNF32-AS2 | MLLT6 |
| ZNF32-AS2 | ZZEF1 |
|  |  |
| BX322557.10 | OTUD3 |
| BX322557.10 | ULK1 |
| KB-1460A1.5 | OTUD3 |
| KB-1460A1.5 | ULK1 |
| RP11-197N18.2 | OTUD3 |
| RP11-197N18.2 | ULK1 |
| RP11-57H14.4 | OTUD3 |
| RP11-57H14.4 | ULK1 |
| RP11-67L2.2 | OTUD3 |
| RP11-67L2.2 | ULK1 |
| RP3-475N16.1 | OTUD3 |
| RP3-475N16.1 | ULK1 |
| RP3-508I15.20 | OTUD3 |
| RP3-508I15.20 | ULK1 |
|  |  |
| BX322557.10 | ATG2B |
| BX322557.10 | MLLT6 |
| BX322557.10 | SUV420H1 |
| CTB-152G17.6 | ATG2B |
| CTB-152G17.6 | MLLT6 |
| CTB-152G17.6 | SUV420H1 |
| PKI55 | ATG2B |
| PKI55 | MLLT6 |
| PKI55 | SUV420H1 |
| RP11-283I3.6 | ATG2B |
| RP11-283I3.6 | MLLT6 |
| RP11-283I3.6 | SUV420H1 |
| TSC22D1-AS1 | ATG2B |
| TSC22D1-AS1 | MLLT6 |
| TSC22D1-AS1 | SUV420H1 |
|  |  |
| ANKRD10-IT1 | TET3 |
| ANKRD10-IT1 | TNRC6C |
| BX322557.10 | TET3 |
| BX322557.10 | TNRC6C |
| CROCCP2 | TET3 |
| CROCCP2 | TNRC6C |
| PKI55 | TET3 |
| PKI55 | TNRC6C |
| RP11-57H14.4 | TET3 |
| RP11-57H14.4 | TNRC6C |
| RP11-890B15.3 | TET3 |
| RP11-890B15.3 | TNRC6C |
| RP3-508I15.19 | TET3 |
| RP3-508I15.19 | TNRC6C |
| ZNF32-AS2 | TET3 |
| ZNF32-AS2 | TNRC6C |
